# Abstracts from HIVR4P 2024, the 5th HIV Research for Prevention Conference, 6 – 10 October, Lima, Peru & Virtual

**DOI:** 10.1002/jia2.26351

**Published:** 2024-10-07

## ORAL ABSTRACT

### OA0102 Infrequent new HIV acquisition among returning pre‐exposure prophylaxis clients in PEPFAR, 2021−2023

E.E. Akom ^1,2^, G. Djomand^3^, T. Lucas^3^, R. Bhatia^3^, A. Arunmozhi^1,2^, E. Dorward^4^, L. Martindale^4^, R. Eakle^5,6^

^1^Walter Reed Army Institute of Research, U.S. Military HIV Research Program (MHRP), Silver Spring, United States, ^2^Henry M. Jackson Foundation for the Advancement of Military Medicine, Bethesda, United States, ^3^U.S. Centers for Disease Control and Prevention, Division of Global HIV and TB, Atlanta, United States, ^4^United States Agency for International Development, Washington, United States, ^5^Bureau of Global Health Security and Diplomacy, Washington, United States, ^6^London School of Hygiene and Tropical Medicine, Department of Global Health and Development, London, United Kingdom

**Background: **The U.S. President's Emergency Plan for AIDS Relief (PEPFAR) has provided pre‐exposure prophylaxis (PrEP) since 2017 and, from October 2021 to September 2023, PEPFAR supported 3,380,468 new initiations. To assess whether large‐scale PrEP implementation retains its HIV prevention benefits, we conducted a review of HIV infections among PrEP users in PEPFAR.

**Methods: **We analysed cross‐sectional, country‐level HIV test result data among PEPFAR‐supported PrEP clients who returned for visits after initiation. Quarterly data were abstracted from the PEPFAR Monitoring, Evaluation, and Reporting (MER) database for 24 countries and three geographic regions from October 2021 to September 2023. The PrEP_CT indicator is defined as number of clients returning to the PrEP service site at least once during the quarter, including either continuous PrEP users or re‐initiating clients. PrEP_CT captures HIV testing data including Positive, Negative and Other results. PrEP_CT does not differentiate between daily and event‐driven oral PrEP and does not allow for aggregation across reporting periods since one individual could be represented in sequential quarters. We calculated the proportion of those testing Positive among those with a Positive or Negative test result.

**Results: **Over the 2‐year review period, there were 4415 Positive tests among the 3,089,834 Positive or Negative HIV tests, for positivity of 0.14%−0.20% by quarter. At individual country level, quarterly positivity was mostly under 1%, ranging from 0% to 0.70% with only two instances of a country reporting ≥1% positivity (1.60% and 1.91%), each in only one quarter. “Other” results were recorded for 19% of all returning PrEP visits overall, ranging across countries from zero to over 80% per quarter.

**Conclusions: **New HIV diagnoses among previously HIV‐negative PrEP clients (prerequisite for initiation on PrEP) were infrequent across PEPFAR PrEP programming, suggesting that most returning clients use PrEP effectively. The findings, together with UNAIDS epidemiological data, indicate that scaling up PrEP is a highly effective prevention option critical to the UNAIDS goal to end HIV as a public health threat by 2030. Tracking longitudinal client‐level data and investigating “Other” results would be beneficial to better understand the dynamics of seroconversion and the contribution of PrEP to achieving epidemic control.

### OA0103 Advancing global HIV prevention: Trends in CDC‐supported pre‐exposure prophylaxis (PrEP) initiation in 37 countries, 2017–2023

M. Peck^1^, S. Davis^1^, C. Toledo ^1^, H.B. Demeke^1^, E. Odoyo‐June^2^, E. Oyugi^3^, T. Hoang^4^, M. Canda^5^, J. Seleme^6^, M. Bock^6^, L. Ndeikemona^7^, S. Dladla^8^, R. Machava^8^, N. Nyagonde^9^, A. Mashauri^10^, A.C. Awor^11^, S. Alamo^11^, O. Chituwo^12^, T. Chisenga^13^, R. Malaba^14^, M. Mutseta^15^, C. Angumua^16^, K.T. Nkwoh^17^, J. Ricketts^18^, K.‐A. Gordon‐Johnson^18^, V. Adamu^19^, S. Adamu‐Oyegun^19^, J. Mondi^20^, S. Bunga^20^, N. Farach^21^, C. Castaneda^22^, R. Bhatia^1^

^1^Centers for Diseases Control and Prevention, Division of Global HIV & TB, Atlanta, United States, ^2^U.S. Centers for Disease Control and Prevention, Division of Global HIV and Tuberculosis, Kisumu, Kenya, ^3^Ministry of Health, Nairobi, Kenya, ^4^U.S. Centers for Disease Control and Prevention, Division of Global HIV and Tuberculosis, Hanoi, Viet Nam, ^5^U.S. Centers for Disease Control and Prevention, Division of Global HIV and Tuberculosis, Maputo, Mozambique, ^6^U.S. Centers for Disease Control and Prevention, Division of Global HIV and Tuberculosis, Windhoek, Namibia, ^7^Ministry of Health and Social Services, Windhoek, Namibia, ^8^U.S. Centers for Disease Control and Prevention, Division of Global HIV and Tuberculosis, Pretoria, South Africa, ^9^U.S. Centers for Disease Control and Prevention, Division of Global HIV and Tuberculosis, Dar es Salaam, the United Republic of Tanzania, ^10^Ministry of Health and Social Welfare, Dodoma, the United Republic of Tanzania, ^11^U.S. Centers for Disease Control and Prevention, Division of Global HIV and Tuberculosis, Kampala, Uganda, ^12^U.S. Centers for Disease Control and Prevention, Division of Global HIV and Tuberculosis, Lusaka, Zambia, ^13^Ministry of Health, Lusaka, Zambia, ^14^U.S. Centers for Disease Control and Prevention, Division of Global HIV and Tuberculosis, Harare, Zimbabwe, ^15^Ministry of Health and Child Care, Harare, Zimbabwe, ^16^U.S. Centers for Disease Control and Prevention, Division of Global HIV and Tuberculosis, Yaoundé, Cameroon, ^17^S. Centers for Disease Control and Prevention, Division of Global HIV and Tuberculosis, Yaoundé, Cameroon, ^18^U.S. Centers for Disease Control and Prevention, Division of Global HIV and Tuberculosis, Kingston, Jamaica, ^19^U.S. Centers for Disease Control and Prevention, Division of Global HIV and Tuberculosis, Abuja, Nigeria, ^20^U.S. Centers for Disease Control and Prevention, Division of Global HIV and Tuberculosis, Juba, South Sudan, ^21^U.S. Centers for Disease Control and Prevention, Division of Global HIV and Tuberculosis, Tegucigalpa, Honduras, ^22^U.S. Centers for Disease Control and Prevention, Division of Global HIV and Tuberculosis, San Salvador, El Salvador

**Background: **Oral pre‐exposure prophylaxis (PrEP) reduces sexual HIV transmission risk by 99% when used correctly. Since 2016, the U.S. Centers for Disease Control and Prevention (CDC), with support from the U.S. President's Emergency Plan for AIDS Relief (PEPFAR), joined country governments in implementing PrEP, targeting populations at highest risk for HIV including adolescent girls and young women (AGYW) aged 15−24 years, and key populations (KPs: men who have sex with men [MSM], transgender people, female sex workers [FSWs], people who inject drugs and people in closed settings).

**Methods: **To contribute to a more thorough understanding of CDC's support towards PrEP scale‐up, PEPFAR Monitoring, Evaluation and Reporting (MER) data were analysed from 2017 to 2023 to describe annual and cumulative PrEP initiations by region and achievement of PrEP targets. Initiations were disaggregated by sex, age and KP.

**Results: **From 2017 to 2023, CDC supported 2,274,396 PrEP initiations in 37 countries (Table), with the majority (96.0%) in sub‐Saharan Africa. Annual PrEP initiations steadily increased from 8800 to 856,238 in 2023, with 37.6% of all initiations occurring in 2023. Targets increased annually, with the highest target in 2023 exceeded by 130.7%. Overall, most PrEP users were 15−24 years old (52.4%) and female (64.0%). Over one‐third of initiations was among KPs (35.4%), which varied by region: 63.4% of KP initiations was among FSW in sub‐Saharan Africa, in the other regions, MSM predominated (69.2% European Region; 77.2% Region of the Americas; 93.0% South East Asia).

**Conclusions: **From 2017 to 2023, CDC supported over 2.2 million PrEP initiation, with the greatest uptake among AGYW in sub‐Saharan Africa. The largest uptake among KPs was FSWs in sub‐Saharan Africa and MSM in South East Asia, European Region and Region of the Americas. Robust PrEP programming and uptake among those at highest risk remains critical to achieving the UNAIDS 2025 goal for 10 million PrEP users.[Table jia226351-tbl-0001]

**Table jia226351-tbl-0001:** OA0103

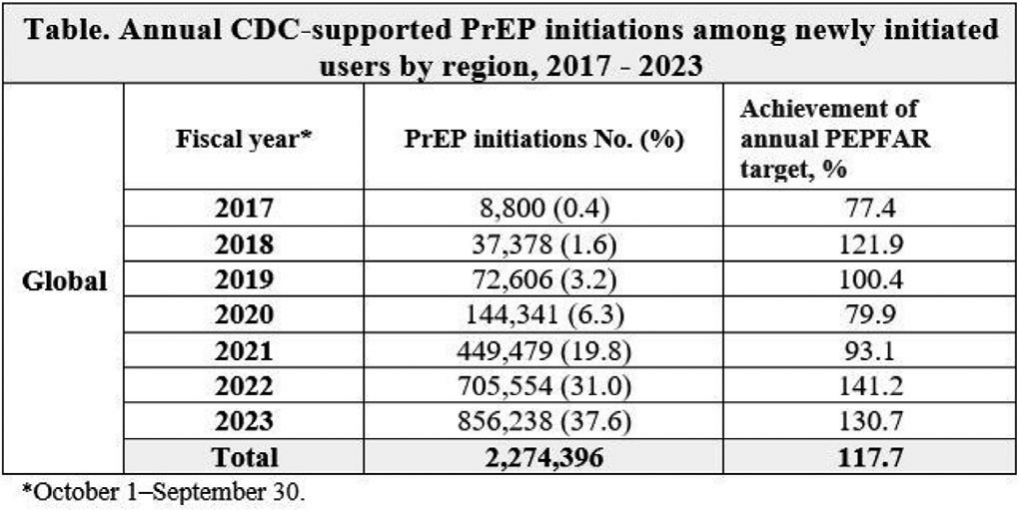

### OA0104 Identifying global typologies of HIV PrEP implementation: An analysis of global data using PrEP‐to‐need ratios and PrEP distribution volumes

C. Verde Hashim ^1^, J. Tailor^2^, M. Prochazka^3^, W. Nyagah^4^, M. Warren^2^, M. Rodolph^3^, R. Baggaley^3^

^1^AVAC, London, United Kingdom, ^2^AVAC, New York, United States, ^3^WHO, Geneva, Switzerland, ^4^AVAC, Nairobi, Kenya

**Background: **To reach 10 million people in high incidence groups with PrEP by 2025, it is vital to assess the global need for and capacity to distribute PrEP using programmatic data. The PrEP‐to‐need ratio (PNR) is the number of annual PrEP initiations relative to HIV incidence, and together with cumulative distribution volumes, it can support identifying implementation gaps.

**Methods: **This retrospective analysis of global data explores PrEP implementation in countries with available data using two metrics (PNRs and volume of PrEP distributed) to identify six typologies of national implementation. PNRs were calculated as the ratio of 2023 PrEP initiations collected from AVAC's Global PrEP Tracker to estimated 2022 UNAIDS HIV incidence per 1000 people, using World Bank's country population estimates. Volumes distributed were proxied by the number of cumulative PrEP initiations to 2023 (Global PrEP Tracker). Both metrics were summarized using medians and interquartile ranges (IQR). PNRs were used to determine countries leading (>5.00), growing (1.00−4.99) or emerging (<1.00) in meeting PrEP need. Volumes were categorized using 100,000 initiations as cut‐off for programme size. Countries were assigned to one of six typologies based on these metrics.

**Results: **Eighty‐four countries had available data for both metrics. The median PNR was 0.81 (IQR: 0.12−3.45). Fifty‐two percent (*n* = 44), 31% (*n* = 26) and 17% (*n* = 14) of countries were classified as emerging, growing or leading in meeting PrEP need, respectively. The median volume of cumulative PrEP distribution was 2918 (IQR: 464−20,734). Eighty‐eight percent (*n* = 74) countries had distribution volumes <100,000, whereas 12% (*n* = 10) had distribution volumes >100,000. Most countries (52%, *n* = 44) had low met need for PrEP and <100,000 PrEP distribution, and four countries (5%) were leading on met need with >100,000 distribution volumes (Table 1).

**Conclusions: **Most PrEP programmes globally are not sufficiently scaled‐up, with countries in Eastern and Southern Africa leading on large‐scale implementation. Focused efforts in Latin America and the Caribbean, Southeast Asia, and Eastern Europe and Central Asia are needed to achieve global goals. Typifying implementation scenarios may facilitate knowledge sharing, global programming and advocacy.[Table jia226351-tbl-0002]

**Table jia226351-tbl-0002:** OA0104

Metric	Distribution volumes >100,000	Distribution volumes <100,000
**PrEP‐to‐need ratio >5 (“Leading”)**	Kenya, Lesotho, Zambia, Zimbabwe	Belgium, Eswatini, France, Guatemala, Iceland, Liberia, Luxembourg, Malta, New Zealand, Portugal
**PrEP‐to‐need ratio <5 and >1 (“Growing”)**	Brazil, Malawi, Mozambique, South Africa, Tanzania, Uganda	Barbados, Botswana, Burundi, Cambodia, Cote d'Ivoire, Croatia, Czechia, DR Congo, El Salvador, Ethiopia, Haiti, Honduras, Italy, Namibia, Nepal, Rwanda, Senegal, Sierra Leone, Thailand, Vietnam
**PrEP‐to‐need ratio <1 (“Emerging”)**	*NA*	Argentina, Armenia, Azerbaijan, Bahamas, Belarus, Belize, Benin, Burkina Faso, Cameroon, Chile, Colombia, Costa Rica, Cuba, Dominican Republic, Ecuador, Eritrea, Gambia, Georgia, Ghana, Grenada, Guyana, India, Indonesia, Iran, Jamaica, Kyrgyzstan, Lao PDR, Lithuania, Madagascar, Malaysia, Mali, Mexico, Moldova, Mongolia, Morocco, Myanmar, North Macedonia, Panama, Papua New Guinea, Peru, Philippines, South Sudan, Tajikistan, Togo

### OA0105 Evaluating overlap between condomless sex and prevention‐effective oral pre‐exposure prophylaxis (PrEP) use throughout pregnancy and postpartum in Cape Town, South Africa

K. Bheemraj ^1^, R. Mvududu^1^, N. Wara^2^, L. Myer^1^, T.J. Coates^2^, D.L.J. Davey^1,2,3^

^1^University of Cape Town, Department of Epidemiology and Biostatistics, Cape Town, South Africa, ^2^University of California Los Angeles, David Geffen School of Medicine, Los Angeles, United States, ^3^University of California Los Angeles, Fielding School of Public Health, Los Angeles, United States

**Background: **Understanding the patterns of sexual behaviour and effective oral PrEP use is crucial for improving effective PrEP use in pregnant and postpartum women.

**Methods: **We offered oral PrEP with HIV risk counselling to 1195 HIV‐negative pregnant women aged >15 years between Aug’19 and Oct’21. We collected data on sexual behaviour and PrEP use during quarterly study visits. We used logistic regression to evaluate the relationship between condomless sex and PrEP use (prevention‐effective use) adjusted for *a priori* confounders: age, education, gestational age in pregnancy and postpartum time. We calculated HIV incidence using number of new HIV diagnoses and cumulative person‐years for each risk category through 12 m postpartum.

**Results: **Among 1195 participants (median age 26 years; IQR: 23−31, median gestational age 21 weeks; IQR: 15−31), 72% (*n* = 864) participants reported recent condomless sex in the past 3 months, with 84% (*n* = 731) initiating PrEP. In the third‐trimester of pregnancy, fewer participants reported recent condomless sex (25%); those reporting condomless sex were 83% less likely to use PrEP compared to those reporting consistent condom use (aOR = 0.17; 95% CI = 0.12−0.24). Overall, 69% (*n* = 784) reported condomless sex in their first postpartum visit, of whom 89% (*n* = 699) reported no PrEP use in the last month. During early postpartum (9−22 weeks), participants reporting condomless sex were 91% less likely to use PrEP (aOR = 0.09; 95% CI = 0.05−0.16). Participants reporting condomless sex and PrEP use decreased to 5% (*n* = 54/1109) through 12 months postpartum. Overall, 16 incident HIV infections were reported among 1195 participants, the overall HIV incident rate was 0.96 per 100 person‐years (95% CI = 0.49−1.42), highest among those reporting condomless sex and no PrEP use (1.18; 95% CI = 0.66−1.96).[Fig jia226351-fig-0001]

**Conclusions: **There was an alignment in condomless sex and effective PrEP use in pregnancy; however, this alignment declines from birth through the postpartum period (through 12 months), when HIV incidence was elevated. There is a critical need for targeted strategies to improve effective PrEP use in postpartum.

**Figure 1 jia226351-fig-0001:**
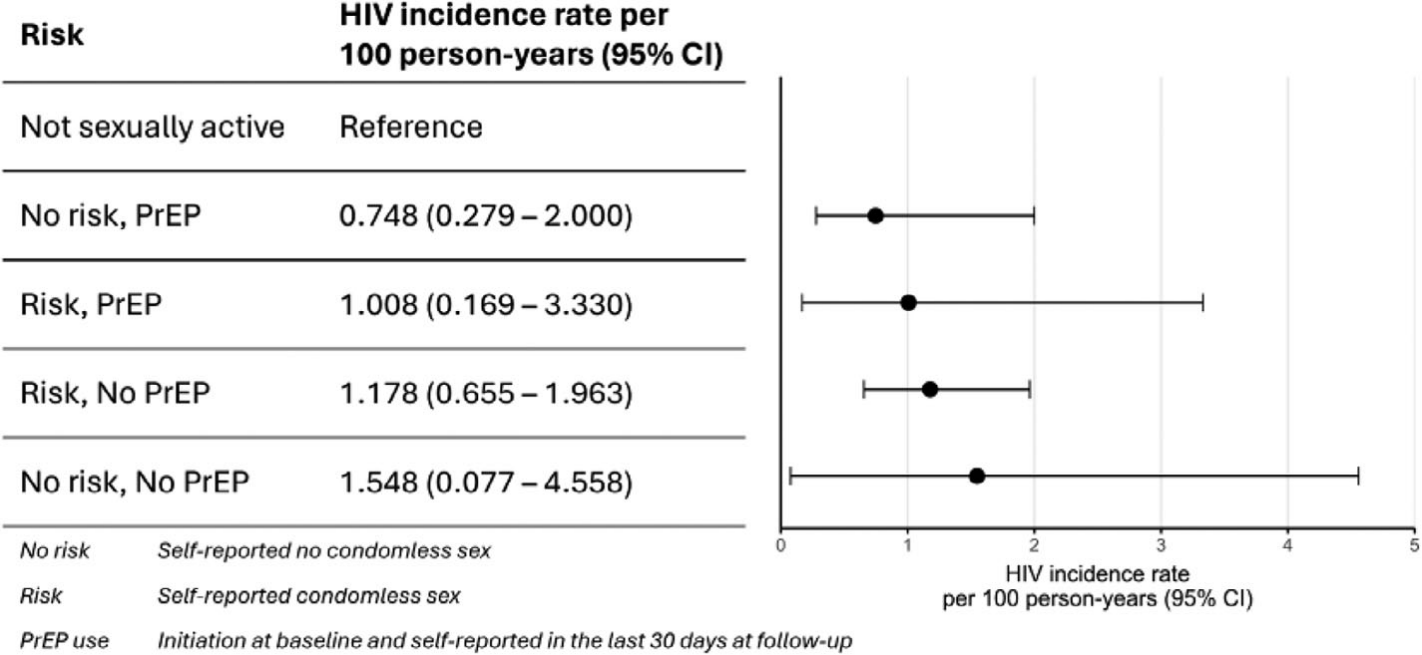
**OA0105**

### OA0106 The choice of indicators influences who is identified as priority populations for HIV epidemic response: a combined analysis of 15 mathematical models from 10 African countries

R. Silhol ^1,2^, R.D. Booton^1^, K.M. Mitchell^1,3^, J. Stannah^1,4^, O. Stevens^1^, D. Dimitrov^5^, A. Bershteyn^6^, L.F. Johnson^7^, S.L. Kelly^8^, H.‐Y. Kim^6^, M. Maheu‐Giroux^4^, R. Martin‐Hughes^8^, S. Mishra^9^, J. Stone^10^, J. Stover^11^, P. Vickerman^10^, S. Baral^12^, D.P. Wilson^13^, J.W. Imai‐Eaton^1,14^, M.‐C. Boily^1,2^

^1^Imperial College London, London, United Kingdom, ^2^HPTN modelling Centre, London, United Kingdom, ^3^Glasgow Caledonian University, London, United Kingdom, ^4^McGill University, Montreal (QC), Canada, ^5^Fred Hutchinson Cancer Center, Seattle (WA), United States, ^6^New York University Grossman School of Medicine, New York (NY), United States, ^7^University of Cape Town, Cape Town, South Africa, ^8^Burnet Institute, Melbourne (VIC), Australia, ^9^University of Toronto, Toronto (ON), Canada, ^10^University of Bristol, Bristol, United Kingdom, ^11^Avenir Health, Glastonbury (CT), United States, ^12^Johns Hopkins University, Baltimore (MD), United States, ^13^Bill & Melinda Gates Foundation, Seattle (WA), United States, ^14^Harvard T. H. Chan School of Public Health, Boston (MA), United States

**Background: **Improving the HIV response necessitates better understanding of the contribution of different groups to new infections (stemming from prevention and treatment gaps), whose levels vary widely across studies, settings and indicators used. We assessed to which extent the choice of indicators affects the estimated contribution of different groups to all new infections.

**Methods: **We compared estimates from 15 African models of the fractions of new infections: Ind1) acquired by a specific group in 2020, Ind2) attributable to direct transmissions from a group in 2020 and Ind3) which could be averted over 2020−2029 if transmission from a group was removed completely (tPAF). We focused on the following key populations (KPs): female sex workers (FSWs), their clients (CFSWs) and men who have sex with men (MSM).

**Results: **Ind2 attributed higher proportion of all new infections than Ind1 to KPs but lower to most non‐KPs, especially in Western Africa (Figure A), meaning that most non‐KPs acquired more infections than they transmitted. Ind3 consistently attributed higher proportion of all new infections than Ind2 to all groups, up to two‐fold among FSWs, meaning that improving treatment as prevention services for FSW have substantial long‐term effects on all new infections (Figure B). Among the 10 models providing estimates for non‐KP by age, eight identified non‐KP women aged 25+ years as acquiring the largest proportion of new HIV infections (Ind1), but only one model estimated that this group transmitted the most infections (Ind2). Conversely, one model identified non‐KP men 25+ years old as acquiring the most infections (Ind1), while six identified them as transmitting the most infections (Ind2).

**Conclusions: **Estimated contributions of groups to all new HIV infections substantially varied across indicators, especially for FSWs and their clients. Future studies should report indicators accounting for long‐term effects on transmission chains alongside the fractions of infections acquired by the group.[Fig jia226351-fig-0002]

**Figure 1 jia226351-fig-0002:**
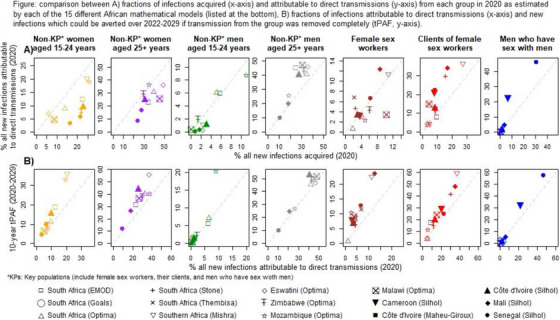
**OA0106**

### OA0202 Safety and tolerability of oral Islatravir (ISL) once‐monthly (QM) as pre‐exposure prophylaxis (PrEP) in cisgender women at elevated risk for acquiring HIV‐1

S. Delany‐Moretlwe ^1^, K. Ahmed^2,3^, P. Kotze^4^, L. Naidoo^5^, C. Louw^6^, N. Mwelase^7^, P. Hwang^8^, N. Joshi^8^, P. Castronuovo^8^, B. Evans^8^, C. Celum^9^, R.A. Heffron^10^, M.N. Robertson^8^, R.M. Plank^8^, IMPOWER‐22 Study Group

^1^University of the Witwatersrand, Johannesburg, South Africa, ^2^Setshaba Research Centre, Soshanguve, South Africa, ^3^University of Pretoria, Faculty of Health Sciences, Department of Medical Microbiology, Pretoria, South Africa, ^4^Qhakaza Mbokodo Research Clinic, Ladysmith, South Africa, ^5^SA Medical Research Council, Chatsworth, South Africa, ^6^Madibeng Centre for Research, Brits, South Africa, ^7^Helen Joseph Hospital, Johannesburg, South Africa, ^8^Merck & Co., Inc., Rahway, United States, ^9^University of Washington, Seattle, United States, ^10^University of Alabama, Birmingham, United States

**Background: **Once‐monthly oral pre‐exposure prophylaxis (PrEP) options are needed to address challenges with adherence to daily regimens and the complexity of injectable PrEP options and its barriers to scale‐up, and to meet research‐identified ideal product profiles. Islatravir (ISL) is a nucleoside reverse transcriptase translocation inhibitor (NRTTI) with nanomolar potency against wild‐type HIV and NRTI‐resistant variants (e.g. M184I/V). Here, we present safety findings from a phase 3 study of oral ISL 60 mg QM compared to daily oral tenofovir‐based PrEP in cisgender women at elevated risk for acquiring HIV‐1 (NCT04644029).

**Methods: **Participants were randomized (1:1) to receive double‐blind ISL QM or emtricitabine/tenofovir disoproxil fumerate (FTC/TDF) once daily. Due to dose/exposure‐related decreases in total lymphocyte and CD4 counts observed across the ISL programme, enrolment and blinded intervention were stopped after ∼1 year. All participants were offered open‐label FTC/TDF for HIV PrEP with continued safety monitoring.

**Results: **Of 1145 participants screened, 730 were randomized and 727 were treated (362 with ISL; 365 with FTC/TDF) and included in the analysis. Most participants were black or African American (92.4%); median age was 25 (range 18−46) years. 94.6% of participants (343 and 345, respectively) entered the open‐label phase. No participant acquired HIV‐1 during the blinded phase. Adverse events (AEs) were experienced by 54.7% of participants in the ISL group and 69.9% of those in the FTC/TDF group, with <2% rated as severe (DAIDS Grade 3 or 4). The most common AEs were headache (11.6%) in the ISL group and bacterial vaginosis (15.3%), headache (14.5%) and nausea (11.2%) in the FTC/TDF group. Infection AEs (MeDRA terms) were reported in 22.4% of ISL participants and 40.5% of FTC/TDF participants. At Month 3, the mean percent change from baseline in total lymphocyte count was −20.8% (95% CI: −24.0, −17.6; *n* = 159) in the ISL group and −8.9% (−11.6, −6.2; *n* = 280) in the FTC/TDF group, with a trend of recovery observed at open‐label Month 12 (−6.3% [−8.7, −3.9; *n* = 322] and −5.3% [−8.0, −2.7; *n* = 295], respectively).

**Conclusions: **In cisgender women at elevated risk of acquiring HIV‐1, decreases in total lymphocyte counts observed with ISL 60 mg QM were not associated with increased infection AEs and were followed by a trend of recovery after drug discontinuation.

### OA0203 Safety and tolerability of oral Islatravir (ISL) once‐monthly (QM) as pre‐exposure prophylaxis (PrEP) in cisgender men and transgender women at elevated risk for acquiring HIV‐1

R.J. Landovitz ^1^, Y. Pinedo^2^, F. Hinestrosa^3^, G. Crofoot^4^, C. Brinson^5^, R. Kaplan^6^, P. Hwang^7^, J. Du^7^, B. Jackson^7^, B. Homony^7^, B. Evans^7^, M.N. Robertson^7^, R.M. Plank^7^, IMPOWER‐24 Study Group

^1^UCLA Center for Clinical AIDS Research and Education, Los Angeles, United States, ^2^Via Libre, Lima, Peru, ^3^Orlando Immunology Center, Orlando, United States, ^4^The Crofoot Research Center, Houston, United States, ^5^Central Texas Clinical Research, Austin, United States, ^6^Desmond Tutu Health Foundation, Cape Town, South Africa, ^7^Merck & Co., Inc., Rahway, United States

**Background: **Challenges to daily oral adherence, operational complexity and insurance barriers to scale‐up of injectable pre‐exposure prophylaxis (PrEP) options, as well as ideal product profiles described by at‐risk populations, support the development of once‐monthly oral PrEP options. Islatravir (ISL) is a nucleoside reverse transcriptase translocation inhibitor (NRTTI) with nanomolar potency against wild‐type HIV and NRTI‐resistant variants (e.g. M184I/V). We report the safety findings from a phase 3 study of oral ISL 60 mg QM compared to daily oral tenofovir‐based PrEP in cisgender men and transgender women at elevated risk for acquiring HIV‐1 (NCT04652700).

**Methods: **Participants were randomized (2:1) to receive double‐blind ISL QM or active control (emtricitabine [FTC] with tenofovir disoproxil [TDF] or tenofovir alafenamide [TAF]) once daily. Due to dose/exposure‐related decreases in total lymphocyte and CD4 counts observed across the ISL programme, enrolment and blinded intervention were stopped after ∼1 year; all participants were offered open‐label FTC/TDF or FTC/TAF for HIV PrEP with continued safety monitoring.

**Results: **Of 695 participants screened, 494 were randomized, treated (328 ISL, 166 control) and included in the analysis (4.5% were transgender, 41.7% white, 25.1% black/African American, 20.4% Asian); median age 27 (range 18−76) years. 93.1% of participants (306 and 154, respectively) entered the open‐label phase. No participant acquired HIV‐1 during the blinded phase. Adverse events (AEs) were reported by 64.3% of the ISL group and 77.1% of the control group and were mild or moderate in most cases (>96%). Infection AEs (MeDRA terms) were reported in 37.2% of ISL participants and 50.0% of control participants; COVID‐19 was the most common AE (11.0% and 13.3%, respectively). Mean percent change from baseline in total lymphocyte count was −7.4% (95% CI: −10.6, −4.1; *n* = 200) in the ISL group and −2.7% (−6.9, 1.4; *n* = 133) in the control group at double‐blind Month 3, −17.4% (‐23.4, ‐11.4; *n* = 53) versus 0.4% (−6.1, 6.9; *n* = 50) at double‐blind Month 9, and ‐0.9% (−3.9, 2.1; *n* = 263) versus −0.4% (−5.2, 4.5; *n* = 124) at open‐label Month 10.

**Conclusions: **In cisgender men and transgender women at elevated risk of acquiring HIV‐1, decreases in total lymphocyte counts observed with ISL 60 mg QM were not associated with increased infection AEs and were followed by a trend of recovery after drug discontinuation.

### OA0204 Acceptability of an on‐demand, single‐dose tenofovir rectal douche for HIV pre‐exposure prophylaxis in young men (ATN 163)

J. Bauermeister^1^, C. Hendrix^2^, W. Lin^1^, J. Webster^1^, A. Agwu^2^, T. Anderson^2^, J. Coleman Lewis^2^, L. Hightow‐Weidman^3^, R. Arrington‐Sanders ^4^

^1^University of Pennsylvania, Philadelphia, United States, ^2^Johns Hopkins University, Baltimore, United States, ^3^Florida State University, Tallahassee, United States, ^4^Children's Hospital of Philadelphia, Philadelphia, United States

**Background: **A behaviourally congruent, on‐demand rectal tenofovir (TFV) douche as pre‐exposure prophylaxis (PrEP) to prevent HIV acquisition via receptive anal intercourse (RAI) would fill a critical gap in PrEP product availability. As part of ATN 163, we examined young men who have sex with men's experiences after participating in a phase I trial, examining the safety, pharmacokinetics and pharmacodynamics, and acceptability of a single‐dose PrEP douche candidate.

**Methods: **We enrolled eight men (62.5% White and 37.5% Black; median age: 21 years; range: 18−24) at a single site between January and August 2022. One 125 ml douche containing 660 mg TFV was rectally administered. Participants completed a product experience survey and in‐depth interview following dosing. We triangulated quantitative and qualitative assessments of the acceptability of rectal douche.

**Results: **Participants rated high overall acceptability on a scale of 1–10, with a mean of 9 (SD = 1.35), with all stating they would recommend use to others. Six (75%) of participants reported ever douching prior to enrolling in the trial. Participants familiar with traditional douching practices appreciated the behavioural congruence of a PrEP douche by aligning HIV prevention with their sexual preparation routines. When asked to select a future HIV protection as a receptive partner, the majority of participants (*n* = 6; 80%) stated that they would prefer the TFV douche over daily oral PrEP, with the remaining two participants (20%) valuing both the douche and daily oral PrEP equally. Participants offered recommendations regarding its design and sustainability, especially among those engaging in receptive anal intercourse, noting it would have consumer appeal among young men if it had comparable effectiveness to oral PrEP.

**Conclusions: **The high acceptability and behavioural congruence of a TFV douche shows promise as an on‐demand PrEP product to prevent HIV among young men. Given its potential as an on‐demand PrEP modality, future clinical development is warranted.

### OA0205 Is the U = U status maintained after switching to a dual regimen? The answer from the Icona cohort study

A. De Vito ^1,2^, G. Madeddu^1^, C. Marelli^3^, A. Cingolani^4^, N. Gianotti^5^, R. Gagliardini^6^, G. Marchetti^7^, M.M. Santoro^8^, F. Maggiolo^9^, A. Cozzi‐Lepri^10^, E. Girardi^11^, A. d'Arminio Monforte^12^, A. Antinori^6^, ICONA Foundation Study

^1^University of Sassari, Unit of Infectious Diseases, Department of Medicine, Surgery, and Pharmacy, Sassari, Italy, ^2^University of Sassari, PhD School in Biomedical Science, Biomedical Science Department, Sassari, Italy, ^3^IRCCS Ospedale Policlinico San Martino, UO Clinica Malattie Infettive, Genoa, Italy, ^4^Fondazione Policlinico Universitario A. Gemelli ‐ Università Cattolica Del Sacro Cuore, Infectious Diseases Unit, Rome, Italy, ^5^IRCCS San Raffaele Scientific Institute, Unit of Infectious and Tropical Diseases, Milan, Italy, ^6^National Institute for Infectious Diseases Lazzaro Spallanzani IRCCS, HIV/AIDS Clinical Unit, Rome, Italy, ^7^ASST Santi Paolo e Carlo, University of Milan, Clinic of Infectious and Tropical Diseases, Department of Health Sciences, Milan, Italy, ^8^University of Rome Tor Vergata, Department of Experimental Medicine, Rome, Italy, ^9^ASST Papa Giovanni XXIII, Unit of Infectious Diseases, Bergamo, Italy, ^10^Institute for Global Health UCL, Centre for Clinical Research, Epidemiology, Modelling and Evaluation (CREME), London, United Kingdom, ^11^National Institute for Infectious Diseases Lazzaro Spallanzani IRCCS, Clinical Epidemiology Unit, Rome, Italy, ^12^ICONA Foundation, Milan, Italy

**Background: **Observational studies found no linked HIV transmissions in sero‐discordant couples when the partner's viral load (VL) was<200 copies/ml (U = U‐status). Data on the risk of losing the U = U status after switching to a dual‐regimen (2DR) when VL was≤200 copies/ml are lacking.

**Methods: **We included PWH in the ICONA cohort who had reached a U = U status as of January 2014 while on triple therapy and were subsequently switched to dolutegravir (DTG)+lamivudine (3TC), DTG+rilpivirine (RPV) or darunavir/boosted (DRV/b)+3TC therapy. The number of person‐months of follow‐up (PMFU) spent with a U = U status has been calculated. The main outcome was the proportion of PWH who spent >10% of their PMFU with a VL>200 cp/ml. A logistic regression model was used to evaluate the association between the use of DTG+3TC versus other recommended 2DR as well as other key exposures and risk of losing the U = U status, after adjusting for confounding.

**Results: **Overall, 3205 PWH were included. Of these, 2509 (78.3%) were switched to DTG+3TC, 696 (21.7%) to other dual. Five hundred and sixty‐nine (17.8%) were females. Overall, only 70 (2.2%) participants spent >10% of their PMFU with a VL>200 copies/ml, and this proportion remained stable over time (*p* = 0.984). The overall median time with VL>200 copies/ml was 4.3 (IQR: 1.7−10.5) PMFU in subjects treated with DTG+3TC and 5.4 (IQR: 2.9−10.9) in subjects treated with other 2DR (*p* = 0.17) and there was evidence for a difference in the proportion of PWH with>10% of time off U = U status (1.9% vs. 3.2%, *p* = 0.046). DTG+3TC therapy showed a lower risk of losing the U = U‐status in the unadjusted analysis but not after controlling for confounding factors (aOR: 0.84; 95% CI: 0.46−1.54; *p* = 0.57) (Figure 1A). Figure 1B shows that female sex at birth, being born outside Italy and history of failure were confirmed risk factors for losing the U = U status in this setting.

**Conclusions: **Our findings confirm a low risk of losing U = U after switching to 2DR, regardless of the type of therapy used.[Fig jia226351-fig-0003]

**Figure 2 jia226351-fig-0003:**
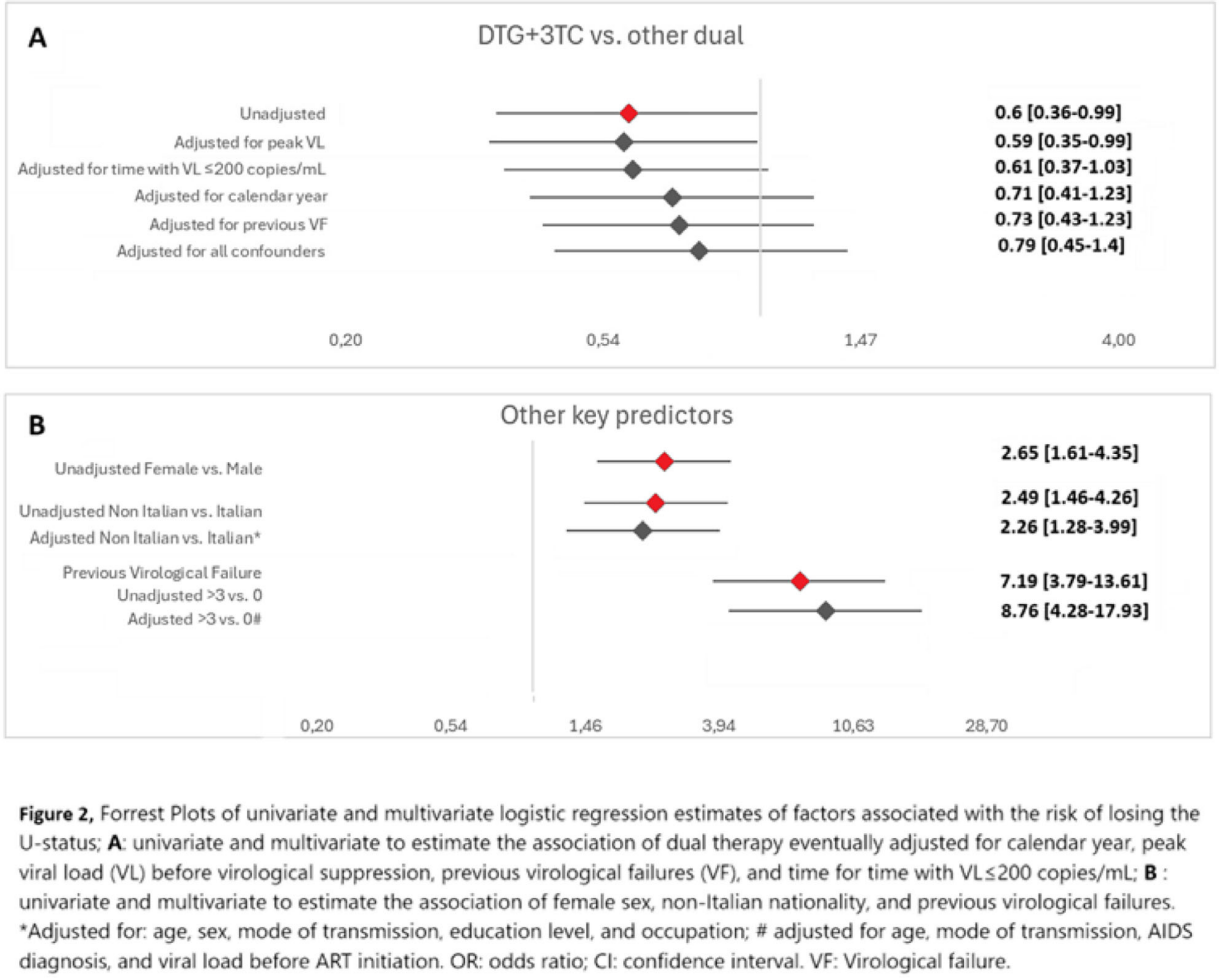
**OA0205**

### OA0206 Development of a long‐acting biodegradable hydrogel injectable system for single or multi‐purpose prevention of HIV and/or pregnancy

M. Peet ^1^, V. Agrahari^1^, N. Chandra^1^, A.‐P. Forsback^2^, M. Akieh‐Pirkanniemi^2^, L. Leino^2^, G. Kaushal^3^, G. Doncel^1^, M. Clark^1^

^1^CONRAD/Eastern Virginia Medical School, OB/GYN, Norfolk, United States, ^2^DelSiTech, Turku, Finland, ^3^Thomas Jefferson University, Pharmaceutical Sciences, Philadelphia, United States

**Background: **There is a scarcity of long‐acting (LA) injectable drug delivery systems capable of providing HIV prevention alone or as multipurpose‐prevention technology (MPT) for simultaneous protection against HIV and unplanned pregnancies. Cabotegravir (CAB) nanosuspension (200 mg/ml) is currently the only approved formulation for LA HIV prevention, but requires a 3 ml intramuscular injection every 2 months. Herein, we report on an LA subcutaneous injectable hydrogel depot platform that is biodegradable, provides lower injection volume and higher drug loading capacity, and may co‐deliver two drugs with discretely controlled release profiles. Preclinical development of hydrogel injectable formulations containing CAB or dolutegravir (DTG) alone and combined with levonorgestrel (LNG) are reported.

**Methods: **Silica‐based hydrogel formulations were optimized using micronized drugs to maximize drug loading and control burst release for enhanced duration. LNG was co‐formulated after encapsulation in silica (Si) microparticles to create a two‐compartment hydrogel matrix design for independently controlled release. Optimization efforts also included improvements on formulation injectability, homogeneity and stability. Lead formulations were administered (0.1 ml) to rats to assess pharmacokinetics, drug−drug interactions (DDIs) and injection site reactions for up to 3 months.

**Results: **Lead formulations containing 515 or 616 mg/ml of micronized CAB, and 359 mg/ml of micronized DTG and 67 mg/ml LNG were selected for 3‐month rat testing after meeting *in vitro* physicochemical targets including injectability through 21G needle. Both CAB formulations showed high and sustained plasma concentrations (>10,000 ng/ml), while the CAB‐616 formulation had a slightly lower C_max_ and more drug remaining in retrieved depots. DTG and LNG plasma levels did not show DDIs and were above 1000 and 0.1 ng/ml, respectively. There were no observed safety signals in any formulations.

**Conclusions: **We have developed a silica‐based hydrogel injectable platform loaded with high concentrations of CAB or DTG/LNG, supporting subcutaneous administration and drug release for at least 3 months for protection against HIV and/or unplanned pregnancy. Additional studies are underway in non‐human primates to better assess protective target drug concentrations and injection intervals. With the successful advancement of LA CAB injectable for HIV prevention, the development of a more clinically and regulatory congruent MPT combining CAB and LNG in this hydrogel platform is ongoing.

### OA0302 HIV therapeutic vaccine induces CD8^+^ T‐cell responses targeting highly networked epitopes in a subset of participants

A. Alrubayyi ^1^, K. Chew^2^, L. Humeau^3^, D. Weiner^4^, S.G. Deeks^5^, R.L. Rutishauser^5^, G.D. Gaiha^1^

^1^Ragon Institute of Mass General, MIT and Harvard, Cambridge, United States, ^2^David Geffen School of Medicine at the University of California, Los Angeles, United States, ^3^Inovio Pharmaceuticals, Inc., San Diego, United States, ^4^Vaccine & Immunotherapy Center, The Wistar Institute, Philadelphia, United States, ^5^University of California San Francisco, San Francisco, United States

**Background: **Functional cytotoxic CD8^+^ T‐lymphocytes (CTL) targeting epitopes derived from structurally constrained regions of the viral proteome (i.e. highly networked) are associated with HIV‐1 control. These epitopes are comprised of amino acid residues involved in important non‐covalent interactions within the protein and, therefore, have reduced mutational tolerance due to putative impact on viral fitness. We previously showed that CTL responses targeting highly networked epitopes can distinguish individuals who naturally control HIV, even in the absence of protective HLA alleles. However, the extent to which a T cell‐based therapeutic vaccine can elicit functional responses towards highly networked epitopes is unknown. Here, we examine the magnitude and proliferative capacity of highly networked CTL responses in individuals enrolled in an HIV DNA therapeutic vaccine trial (PENNVAX).

**Methods: **Forty‐eight participants enrolled in the PENNVAX therapeutic vaccine trial were randomized 1:1:1 to receive DNA vaccination with multiclade Gag/Pol+IL‐12, Gag/Pol/Env+IL‐12 or placebo. Vaccine or placebo was administered by intramuscular electroporation at weeks 0, 4, 8 and 12. T‐cell responses were evaluated at baseline and 2 weeks after the last dose (14 weeks). *Ex vivo* T‐cell reactivity was measured by IFN‐γ ELISpot and proliferative capacity through a 6‐day CFSE‐based assay with overlapping Gag, Pol and Env peptide pools (clade B) and individual optimal clade B epitopes (202 epitopes) matched to each participant's HLA haplotype.

**Results: **DNA vaccination using consensus HIV immunogens induced higher T‐cell reactivity and proliferation, with 45% and 36% of participants showing a two‐fold increase in Gag‐ and Pol‐specific responses, respectively (in Gag/Pol arm). However, these responses were mostly boosted responses from pre‐existing T cells to known immunodominant epitopes. Interestingly, CD8^+^ T‐cell responses to 27 highly networked epitopes were identified in 23% of participants (*n* = 8/37), although with only 11% of participants (*n* = 4/37) having generated a new response. Interestingly, these *de novo* responses were observed in participants without protective HLA alleles.

**Conclusions: **DNA vaccination using full protein immunogens induced new responses towards highly networked epitopes, although in a low frequency of participants (∼11%). These data warrant further investigation to understand the potential of these responses and immune‐focusing vaccines to effectively control viral rebound after treatment interruption.

### OA0303 Structural characterization of the affinity maturations of an intermediate VRC01‐class bnAb elicited by immunizations in VRC01 precursor transgenic mice

A. Galkin ^1^, L. Lei^2^, T. Ota^3^, X. Zhao^2^, S. O'Dell^4^, J. Guenaga^3^, M. Ota^3^, X. Shang^1^, C.‐I Chiang^2^, R. Wyatt^3^, N. Doria‐Rose^4^, J. Mascola^4^, E. Pozharski^5^, D. Nemazee^3^, Y. Li^1^

^1^Institute for Bioscience and Biotechnology Research, University of Maryland, Department of Microbiology and Immunology, University of Maryland School of Medicine, Rockville, United States, ^2^Institute for Bioscience and Biotechnology Research, University of Maryland, Rockville, United States, ^3^Department of Immunology and Microbiology, Scripps Research Institute, La Jolla, United States, ^4^Vaccine Research Center, NIAID, NIH, Bethesda, United States, ^5^Institute for Bioscience and Biotechnology Research, University of Maryland, Department of Biochemistry and Molecular Biology, University of Maryland School of Medicine, Rockville, United States

**Background: **Recent advances in structural biology and immunogen design have led to the identification of conserved epitopes on HIV‐1 Envs that could serve as vaccine targets. However, the elicitation of VRC01‐class bNAbs to overcome the steric hindrance imposed by N276 glycan in diverse Env trimers remains challenging, despite the progresses in germline‐targeting immunogen designs and the development of sequential immunization strategies.

**Methods: **We used Cryo‐electron microscopy (CryoEM) to investigate the interaction between an immunization‐elicited intermediate VRC01‐class bnAb, capable of neutralizing HIV‐1 virus bearing N276 glycan and the cognate Env trimer.

**Results: **From transgenic mice expressing VRC01‐germline heavy/light chain (HC/LC), which were immunized with Env‐CD4i fusion protein presented on nanoparticles and a modified Env trimer, 45_01dG5 bearing N276 glycan, we have cloned a panel of VRC01 lineage mAbs to characterize the affinity maturation driven by the immunization. We observed clustered VRC01‐class somatic hypermutations (SHMs) in the HC/LC variable regions of these mAbs. Selected mAbs could neutralize a small panel of viruses with Envs bearing N276 glycan, one of the critical roadblocks impeding VRC01‐class mAb affinity maturation. To examine the structural basis of how the affinity maturations confer Env trimer recognition, we obtained high‐resolution CryoEM reconstructions of representative antibody (bc24) in complex with 45_01dG5 Env trimers with N276 glycan (bc24‐dG5_276gl+). Around 68% of the interaction surface between bc24 and dG5_276gl+ trimer involves bc24 CDRH2, where multiple SHM mutations have been accumulated. The SHM in the LC is critical to accommodate N276 glycan: three out of four mutated residues in the bc24 CDRL1 interact with the 234/276 glycan complex. Comparison of dG5_276gl+ trimers with and without bc24 reveals significant N276 glycan movement upon bc24 binding to avoid clashes with bc24 LC: with 79° of angle rotation, and 14 Å of beta‐D‐mannopyranose moiety shift.

**Conclusions: **Our sequential immunization strategy facilitates the elicitation of antibodies, such as bc24, which lack deletion in the CDRL1 observed in the bnAb VRC01. This subclass of antibodies is able to accommodate the N276 glycan by reorienting it, a critical step for achieving neutralization breadth.

### OA0304 Identification of non‐neutralizing antibodies that compete with CD4 binding site‐neutralizing antibodies in HIV infection

M.L. Rodriguez de la Concepción ^1^, N. Predreño‐López^1^, J. Carabelli^1^, E. Ainsua‐Enrich^1^, C. Rovirosa^1^, C. Ávila‐Nieto^1^, C. Aguilar‐Gurrieri^1^, F. Cunyat^1^, E. Pradenas^1^, S. Marfil^1^, L.M. Molinos‐Albert^1^, F. Tarrés‐Freixas^1^, B. Clotet^1,2,3,4,5^, J. Blanco^1,3,4,5^, J. Carrillo^1,3,5^

^1^IrsiCaixa, Badalona, Spain, ^2^Fight Infections Foundation, Germans Trias i Pujol Hospital, Badalona, Spain, ^3^Germans Trias i Pujol Research Institute (IGTP), Can Ruti Campus, Badalonaspainspain, Spain, ^4^Center for Health and Social Care Research (CESS), Faculty of Medicine, University of Vic – Central University of Catalonia (UVic – UCC), Vic, Spain, ^5^CIBERINFEC. ISCIII, Madrid, Spain

**Background: ** Non‐neutralizing antibodies (non‐NAbs) are elicited earlier than neutralizing antibodies (NAbs) and predominate in the humoral immune response to human immunodeficiency virus (HIV) during infection. However, their role in the pathogenesis of HIV infection remains poorly understood. Since the number of antibodies that can simultaneously bind to the envelope glycoprotein (Env) is limited due to steric hindrance, non‐NAbs might impede the access of NAbs to Env, reducing their anti‐viral efficacy, and delaying their *de novo* generation by competitive mechanisms. Since this information is crucial to improve NAbs‐based immunotherapies and refining HIV immunogens, we aimed here to define whether neutralization‐interfering antibodies (NiAbs) are generated during natural HIV acquisition. Particularly, we focused on CD4‐binding site (CD4bs) antibodies as they are frequently elicited during HIV infection.

**Methods**: Plasma samples from 19 people living with HIV (PLWH) with viral load higher than 50 copies/ml and low neutralizing activity against the HIV BaL isolate (reciprocal IC50<500) were screened for the presence of CD4bs b12‐blocking antibodies by ELISA, flow cytometry and neutralization assays. Eight HIV‐1‐uninfected individuals were included as controls. Env‐specific antibodies were generated by RT‐PCR from memory B cells isolated by FACS or single‐cell microcultures.

**Results**: IgGs from PLWH blocked b12 binding to Env, which correlated with the levels of Env‐binding antibodies. Eight clonally unrelated Env‐specific monoclonal antibodies were generated from a selected individual. These antibodies showed limited (*n* = 3/8) or non‐neutralizing activity (*n* = 5/8) and targeted g120 (*n* = 5/8), or gp41 (*n* = 3/8). All of them bound to purified recombinant Env trimers by ELISA or the native Env expressed on the surface of chronically infected MOLT cells. Three anti‐gp120 competed with CD4bs NAbs for Env‐binding. Interestingly, two antibodies, one targeting gp41 and the other recognizing gp120, also reduced the neutralizing activity of the CD4bs NAbs b12 and VRC01.

**Conclusions**: Non‐NAbs targeting different epitopes within Env reduced the binding and neutralizing activity of CD4bs antibodies (b12 and VRC01). The generation of NiAbs may represent a poorly described HIV immune escape mechanism that can hamper the development and function of NAbs. The identification of Env regions inducing NiAbs might be crucial for the design of successful HIV immunogens.

### OA0305 Early neutrophil recruitment after heterologous late boost with and without new adjuvant in RV546 potentially contributes to vaccine‐specific antibody responses

A. Schuetz ^1,2,3^, S. Jongrakthaitae^1^, B. Slike^2,3^, N. Tragonlugsana^1^, B. Keawboon^1^, S. Sukhumvittaya^1^, S. Vimonpatranon^1^, Y. Phuang‐Ngern^1^, S. Krebs^2^, E. Serti Chrisos^2,3^, S. Akapirat^1^, J. Dhitavat^4^, P. Pitisuttithum^4^, S. Nitayaphan^5^, J.A. Ake^2^, J. Cowden^1^, S. Vasan^2,3^, RV546 Study Group

^1^WRAIR‐Armed Forces Research Institute of Medical Sciences, Bangkok, Thailand, ^2^U.S. Military HIV Research Program, Silver Springs, United States, ^3^Henry M. Jackson Foundation for the Advancement of Military Medicine, Bethesda, United States, ^4^Mahidol University, Vaccine Trial Center, Bangkok, Thailand, ^5^Royal Thai Army Clinical Research Center, Armed Forces Research Institute of Medical Sciences, Bangkok, Thailand

**Background: **Neutrophils contribute to vaccine adjuvant effects by creating an inflammatory environment through cytokine and DNA release and may impact vaccine‐induced antibody responses through B‐cell activation, but are often overlooked due to loss during processing. Here, we assess fresh neutrophil responses upon heterologous booster vaccination with and without the Army Liposomal Formulation with QS21 (ALFQ) adjuvant.

**Methods: **RV546 enrolled participants receiving RV144 ALVAC‐HIV/AIDSVAX B/E prime/boost vaccine regimen followed by late boosts (RV306) who then received an additional heterologous boost 6−8 years later with gp120‐CD4 IHV01 and gp120 A244 with or without ALFQ adjuvant. Neutrophil activation and function (neutrophil extracellular nets‐NETs) was assessed using flowcytometry at baseline, day(d) 1, 7, 14 and 168 post‐vaccination. Binding antibodies (BAb) were measured by Luminex at d14. Interim blinded data include placebo recipients.

**Results: **The frequency of CD66b^+^CD16^+^FcαRI^+^ neutrophils increased after vaccination and remained elevated throughout d168 with and without ALFQ (one‐way ANOVA: *p*<0.0001 and *p* = 0.04, respectively). Participants receiving ALFQ had increased frequencies of CD64^+^ activated neutrophils at d1 and d7 compared to baseline (baseline: 1.68% vs. d1: 2.28% and d7: 2.51%; *p* = 0.04 and *p* = 0.04, respectively), normalizing to pre‐vaccination levels at d14. Similar trends were observed without ALFQ. Neutrophil expression of B‐cell activating factor (BAFF) increased at d1 and peaked at d14 post‐vaccination with and without ALFQ (one‐way ANOVA: *p* = 0.0002 and *p* = 0.003, respectively). However, only in participants receiving ALFQ, a transient increase of IL‐21^+^ neutrophils at d1 was observed (baseline: 0.98% vs. d1: 1.42%; *p* = 0.02). Formation of NETs increased at d1 in both groups (with ALFQ: baseline: 4.39% vs. d1: 9.96%, *p* = 0.0007; without AFLQ: baseline: 2.91% vs. d1: 3.30%, *p* = 0.003) and remained elevated throughout d14 before normalizing to pre‐vaccination levels at d168. Interestingly, in participants receiving ALFQ, BAb levels against IHV01 at d14 correlated with the frequency of IL‐21^+^ neutrophils at d1 (*r* = 0.53, *p* = 0.05) and CD64^+^ neutrophils at d7 (*r* = 0.56, *p* = 0.04).

**Conclusions: **Overall, neutrophils are recruited early after vaccination potentially contributing to a transient inflammatory environment and providing B cell help, both required for induction of vaccine‐specific antibody responses, further adjuvanted by ALFQ. These results warrant additional interrogations into the role of neutrophils during initial vaccine responses.

### OA0306 Broadest binding antibody and cellular responses seen with a matched prime‐boost polyvalent DNA plasmid vaccine in a comparative analysis of 13 HIV vaccine trials conducted worldwide

Z. Moodie ^1^, S.S. Li^1^, E.E. Giorgi^1^, L.D. Williams^2^, O. Dintwe^3^, L.N. Carpp^1^, S. Chen^1^, K.E. Seaton^2^, S.S. Sawant^2^, L. Zhang^2^, J. Heptinstall^2^, S. Liu^4^, N. Grunenberg^1^, S. Rerks‐Ngarm^5^, P. Pitisuttithum^6^, S. Nitayaphan^7^, J.A. Ake^8^, S. Vasan^8^, G. Pantaleo^9^, I. Frank^10^, L.R. Baden^11^, P.A. Goepfert^12^, M. Keefer^13^, Z.M. Chirenje^14^, M.C. Hosseinipour^15^, K. Mngadi^16^, F. Laher^17^, N. Garrett^18^, L.‐G. Bekker^19^, S. De Rosa^1^, E. Andersen‐Nissen^3^, J.D. Kublin^1^, S. Lu^4^, P.B. Gilbert^1^, G.E. Gray^17^, L. Corey^1^, M.J. McElrath^1^, G.D. Tomaras^2^

^1^Fred Hutchinson Cancer Center, Seattle, United States, ^2^Duke University School of Medicine, Durham, United States, ^3^Hutchinson Centre Research Institute of South Africa, Cape Town HVTN Immunology Laboratory, Cape Town, South Africa, ^4^University of Massachusetts, Chan Medical School, Worcester, United States, ^5^Thai Ministry of Public Health, Nonthaburi, Thailand, ^6^Mahidol University, Vaccine Trials Center, Faculty of Tropical Medicine, Bangkok, Thailand, ^7^Royal Thai Army, Armed Forces Research Institute of Medical Sciences, Bangkok, Thailand, ^8^Walter Reed Army Institute of Research, U.S. Military HIV Research Program,, Silver Spring, United States, ^9^Service of Immunology and Allergy, Lausanne University Hospital and University of Lausanne, Lausanne, Switzerland, ^10^Perelman School of Medicine, University of Pennsylvania, Philadelphia, United States, ^11^Department of Medicine, Brigham and Women's Hospital, Boston, United States, ^12^University of Alabama at Birmingham Heersink School of Medicine, Birmingham, United States, ^13^University of Rochester, Department of Medicine, Rochester, United States, ^14^University of Zimbabwe College of Health Sciences Clinical Trials Research Centre, Harare, Zimbabwe, ^15^UNC‐Project Malawi, Lilongwe, Malawi, ^16^Aurum Institute, Johannesburg, South Africa, ^17^Perinatal HIV Research Unit (PHRU), Wits Health Consortium, Soweto, Johannesburg, South Africa, ^18^School of Nursing and Public Health, University of KwaZulu‐Natal, Durban, South Africa, ^19^Desmond Tutu HIV Centre, University of Cape Town, Cape Town, South Africa

**Background: **The HIV Vaccine Trials Network (HVTN) conducted numerous individual phase 1/2/3 trials following RV144 to assess delivery systems, immunogen sequences, doses, schedules and adjuvants. We compared immune responses across these trials to characterize regimens that elicited the strongest responses.[Fig jia226351-fig-0004]

**Figure 1 jia226351-fig-0004:**
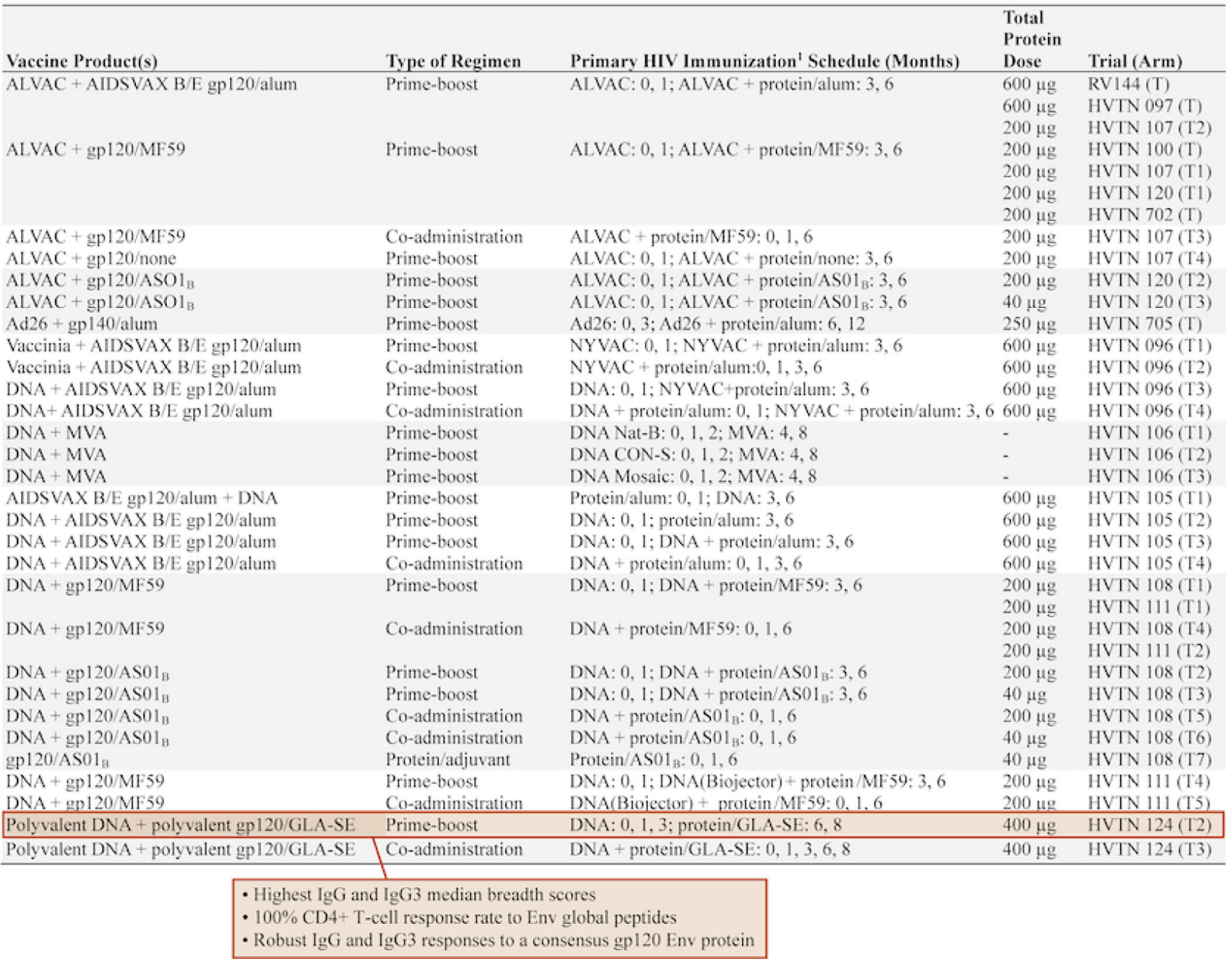
**OA0306**

**Methods: **Validated assays were used to measure immune responses to 36 vaccine regimens from 13 clinical trials, conducted across four continents between 2003 and 2018, that enrolled healthy adults without HIV. HIV‐specific CD4^+^ T‐cell (IFN‐g and/or IL‐2) and binding antibody (IgG, IgG3) responses were measured 2 weeks post‐last primary HIV immunization. Analysis included all participants who received the protocol‐specified primary HIV vaccinations, excluding those diagnosed with HIV before immunogenicity assessment (*n* = 1688). IgG and IgG3 breadth scores were the geometric mean of binding antibody responses to the three heterologous V1V2 antigens with the highest median responses among all participants by trial and vaccine regimen. CD4^+^ T‐cell magnitude to any Env was the maximum over all Env pools tested. Positive response rates and breadth scores were compared between regimen pairs by Barnard's exact test and Wilcoxon rank‐sum test, respectively.

**Results: **Highest median IgG breadth scores were elicited by the prime‐boost and co‐administrated regimens of polyvalent DNA with matched polyvalent protein adjuvanted with GLA‐SE (HVTN 124) and the B/E ALVAC+gp120/alum prime‐boost regimen (HVTN 097, RV144). The prime‐boost HVTN 124 regimen induced significantly higher IgG3 breadth scores than all other regimens (median score>5.9‐fold higher, all *p*<0.001) with robust IgG and IgG3 responses to a consensus gp120 Env protein (Con 6 gp120/B) and 100% CD4^+^ T‐cell response rate to Env global peptides.

**Conclusions: **DNA plasmids provided an immunogenic priming platform when followed by adjuvanted Env protein. Greatest binding antibody breadth and cellular responses were seen with the prime‐boost polyvalent DNA‐matched polyvalent protein/GLA‐SE regimen.

### OA0402 SPrEP—online PrEP and PEP: the first online platform for access to HIV prophylaxes in Brazil

M.C. Abbate ^1^, A.Q. Silva^1^, C.M. Matos^1^, S.F.M. Rodrigues^1^, L. Pinheiro^1^, M.A. Barbosa^1^, R.F. Camargo^1^, G.M. Rodrigues^1^, M.L.F. Camargo^1^, B.L. Macedo^1^

^1^Sao Paulo Municipal Health Department, STI/Aids Coordination, São Paulo, Brazil

**Background: **In the city of São Paulo, the highest concentration of new HIV cases is among men and young people aged 15−29. Pre‐exposure prophylaxis (PrEP) and post‐exposure prophylaxis (PrEP) for HIV have been decisive in reducing the number of new infections. The STI/Aids Coordination of São Paulo has been diversifying strategies to break down access barriers to PrEP and PEP.

**Methods: **Through teleconsultations, SPrEP—online PrEP and PEP is the first online service of its kind in Brazil. It operates with an alternative schedule in comparison to traditional services, from 6 PM to 10 PM, every day, including holidays and weekends. To access SPrEP, the user must be registered in the e‐saúdeSP app. The PrEP request can be triggered by uploading an image or PDF file, which can also be an HIV self‐test, with a negative result for up to 7 consecutive days. In the case of PEP, an HIV test is not required. An appointment request is generated and the user is video called within a few minutes. Medication prescriptions can be sent by email, SMS or WhatsApp and can be collected in 45 units, 17 of which are available 24 hours a day.

**Results: **From June/2023 to April/2024, SPrEP had 622,000 accesses, with 1232 appointments, 288 for PrEP, 365 for PEP, 126 follow‐up appointments and 403 for other appointments. Of the total number of appointments, 33% were for people aged 15−29; 57.2% for white people and 36.3% for black people. Regarding medication pick up, 65.7% did so in 24‐hour units and 34.3% in units with regular hours.

**Conclusions: **With more than 40,000 PrEP registrations since 2018, the city of São Paulo has the largest distribution hub for this prophylaxis in Brazil. SPrEP is another option for the population to have access to prophylaxes in alternative times and at various medication pick‐up points at any time of the day in São Paulo, at any time via teleconsultation, which offers access to a medical prescription and to online medical examination requests.

### OA0403 Examining the impact of stigma on the PrEP cascade among indigenous gay and bisexual men in Guatemala

C. Purnell ^1^, J. Yac^2^, B. Saucedo Mérida^3^, L. Weyer Johnson^1^, D.M. Elias Rodas^4^, E.R. Orellana^5^, D.A. Davis^1^

^1^Duke Global Health Institute, Durham, United States, ^2^Asociación IDEI, Quezaltenango, Guatemala, ^3^Trabajando Unidos, Huehuetenango, Guatemala, ^4^Universidad del Valle de Guatemala, Guatemala City, Guatemala, ^5^University of Washington, Seattle, Seattle, United States

**Background: **Indigenous gay and bisexual men (GBM) in Guatemala experience intersectional stigma based on their sexual orientation (SO) and Indigenous identity, which likely negatively impacts their engagement with HIV prevention services. We aimed to assess the relationship between different forms of stigma and the PrEP cascade among Indigenous GBM to inform PrEP programming.

**Methods: **Between June 2023 and March 2024, we administered a cross‐sectional questionnaire to Indigenous GBM (*n* = 348) in collaboration with two community‐based organizations. We estimated prevalence of enacted SO stigma, internalized SO stigma, interpersonal racism, internalized racism, and systemic racism and the PrEP cascade (awareness, intent‐to‐use, uptake, adherence). We examined the relationship between stigma and the PrEP cascade using logistic regression.

**Results: **We found a high burden of SO stigma and Indigenous identity stigma: 93% of participants reported hearing that their SO was not normal; 53% reported losing a relationship with a family member; and 44% suffered physical violence for being gay/bisexual. Indigenous GBM reported high levels of interpersonal racism (71%) and moderate or high systemic racism (67%); 15% reported high internalized racism. Seventy‐three percent participants were aware of PrEP and a third (31%) had initiated PrEP. Among non‐users, less than half (45%) found PrEP acceptable and 28% intended to use PrEP. Among PrEP users, 66% reported perfect 7‐day adherence. In multivariable analyses, we found that for each one unit increase in internalized racism, participants were nearly twice as likely to be aware of PrEP (OR: 1.86, 95% CI: 1.04−3.36, *p* = 0.04). For every one unit increase in enacted SO stigma (OR: 2.43; 95% CI: 1.20−4.98; *p* = 0.01), systemic racism (OR: 2.04, 95% CI: 1.30−3.26, *p*<0.01) and internalized racism (OR: 2.30, 95% CI: 1.29−4.17, *p*<0.01), participants were over twice as likely to use PrEP. Internalized SO stigma and interpersonal racism were not significantly associated with any PrEP outcomes.

**Conclusions: **Indigenous GBM experience high rates of stigma based on their SO and Indigenous identity which may contribute to greater awareness of their HIV vulnerability, leading to an increased use of PrEP. Future research should explore the role of self‐assessed HIV risk on the relationship between stigma and PrEP use.

### OA0404 Strategic alliance to reduce barriers to treatment of migrants with HIV and/or TB in Peru

A.L. Boccardi Vidarte^1^, P. Giusti^2^, M. Castillo ^3^, P. Bracamonte^4^, E. Nepo^5^

^1^UNAIDS Andean Countries, Director, Lima, Peru, ^2^MPH, MD and Pediatrician, Chief of Party LHSS for Peru, Lima, Peru, ^3^SI DA VIDA, Journalist ‐ Communications Area Sí, da Vida / General Producer at Conexión Vida / Coordinator of GIVAR, Lima, Peru, ^4^UNAIDS Andean Countries, Strategic Information Adviser, Lima, Peru, ^5^Universidad Peruana de Ciencias Aplicadas (UPC), Profesor de Salud Publica de la Escuela de Medicina de la UPC, Lima, Peru

**Background: **At the end of 2023, 1.6 million Venezuelan migrants were living in Peru. The Peruvian's Ministry of Health reports that TB cases among migrants are exponentially on the rise since 2016. The HIV prevalence rate among Venezuelan migrants is 0.6–0.7%, double than the general population (0.3%). Between 2018 and 2022, there were 4043 migrants living with HIV on antiretroviral treatment (ART) but the total number of migrants with HIV is estimated in 8000. It is required that people with TB and HIV take medical tests before initiating treatment. For migrants in irregular situation, the out‐of‐pocket expense of these is very high and cannot initiate treatment. This limits cure or control, increases risk of transmission and AIDS‐related deaths.

**Methods: **An Steering Group was established in 2022 to develop a bill that incorporates migrants with HIV and/or TB into the National Health Insurance (SIS) while they obtain regular migration status. The group is comprised of USAID, UNAIDS, LHSS, VENEACTIVA, GIVAR, IOM, OPEMS UPCH, UNHCR, Social Observatory of TB of Peru and Partners in Health. The bill is evidence‐based, states public health reasons and a cost‐benefit analysis for the measure. The group held advocacy meetings with national authorities and legislators, an awareness raising event with the Ombudsman's Office, trained spokespersons from PLHIV and migrants groups, carried out political advocacy, and information material was distributed.

**Results: **In June 2023, the bill (5253) entitled “*Law amending Legislative Decree 1164 for the health protection of refugees and migrants with HIV/TB”* was submitted to the Congress, in January 2024 was unanimously approved by the Health and Population Committee and in March 2024, the Budget and General Account Committee of the Congress established an opinion in favour. The driving group is optimistic that it will be approved by the plenary before the end of the current legislature.

**Conclusions: **The strategic alliance between UN agencies, bilateral cooperation, academia, CSOs, PLHIV and migrants has been key for the development of the bill and an advocacy strategy to close the gap in access to HIV and/or TB treatment by migrants, and thus reduce the risk of transmission and AIDS‐related deaths.

### OA0405 Venezuelan migrant cisgender women sex workers in Lima, Peru: is time increased in Lima associated with less HIV‐related vulnerability?

K. Konda ^1,2^, A. Silva‐Santisteban^2^, D. Apedaile^3^, A. Villon^4^, M. Castro‐Arteaga^3^, D.F. De Jesus Leon Morris^2^, A. Perez‐Brumer^2,3^

^1^Keck School of Medicine, University of Southern California, Department of Population and Public Health Sciences, Los Angeles, United States, ^2^Center for Interdisciplinary Research in Sexuality, AIDS and Society. Universidad Peruana Cayetano Heredia, Lima, Peru, ^3^Dalla School of Public Health, University of Toronto, Toronto, Canada, ^4^Asociacion de Trabajadoras Sexuales Miluska Vida y Dignidad, Lima, Peru

**Background: **Peru is home to the second‐largest Venezuelan migrant population. Lima hosts over 1 million Venezuelans, half of whom are women, with many engaging in sex work to earn income. To inform HIV prevention and care interventions tailored to migrant women vulnerable to HIV, such as sex workers, we explored how time since arrival in Lima, Peru was associated with HIV‐related vulnerabilities.

**Methods: **Between February and March 2024, 198 Venezuelan migrant cisgender women sex workers in Lima participated in a bio‐behavioural survey including testing for HIV, syphilis, gonorrhoea and chlamydia. We compared participants reporting living in Lima for ≥4 years versus those in Lima <4 years, using chi‐square and Fisher's exact tests to explore if less time living in Lima was associated with increased experience of HIV‐related vulnerabilities.

**Results: **Among participants (median age 32 years, IQR 26−39), most (72.2%) had permanent or temporary residency, only 23% had health insurance and 76.3% reported earning less than half of Peru's monthly minimum wage. Migrants in Lima for <4 years were significantly more likely to report recent difficulty finding a safe place to sleep at night (48% vs. 23%, *p* = 0.001), difficulty paying rent this month (62.1% vs. 38.2%, *p* = 0.001) and sex work as their main source of income (80.0% vs. 63.3%, *p* = 0.039), compared to migrants in Lima for ≥4 years. STI prevalence (HIV, syphilis, gonorrhoea or chlamydia) was similar between the two groups (9.2% vs. 7.7%) as were other types of vulnerabilities including food/housing insecurity, mental health and violence.

**Conclusions: **Increased time in Lima was associated with decreased reliance on sex work for income and increased autonomy in sex work practices. Though HIV prevalence was low, experiences of food and housing insecurity, intimate‐partner violence and mental health remained disproportionality high, which could increase their vulnerability to STIs, necessitating comprehensive interventions for this population.[Table jia226351-tbl-0007]

**Table jia226351-tbl-0007:** OA0405

**Relationship between time in Lima among Venezuelan migrant cisgender women sex workers**			

	Arrived in Lima <4 years ago *N* = 87	Arrived in Lima ≥4 years ago *N* = 110	*p*‐value
Difficulty finding a safe place to sleep (past 6 months)	36 (48.0%)	22 (23.2%)	0.001
Worried about not having enough food to eat (past 12 months)	78 (96.3%)	100 (96.2%)	0.767
Sex work is main source of income	56 (80.0%)	50 (63.3%)	0.038
Able to always choose own sex work clients	45 (66.1%)	64 (83.1%)	0.030
Any HIV/STI	8 (9.2%)	8 (7.7%)	0.911
Has health insurance	17 (20.5%)	27 (25.5%)	0.527
Intimate‐partner violence (past year)	20 (27.0%)	32 (33.0%)	0.502
Mental Health:			
Anxiety Depression Severe psychological distress	35 (45.5%) 32 (43.8%) 16 (22.2%)	37 (37.8%) 39 (39.0%) 17 (18.1%)	0.383 0.630 0.641

### OA0406 Needed policy reform and programmatic efforts to make Peruvian HIV prevention and care accessible for Venezuelan sexual and gender minority migrants: qualitative exploration

A. Silva‐Santisteban ^1^, J.C. Ramirez^1^, K. Solari^1^, A. Perez‐Brumer^2^

^1^Universidad Peruana Cayetano Heredia, Center for Interdisciplinary Research in Sexuality, AIDS and Society, Lima, Peru, ^2^University of Toronto, Dalla Lana School of Public Health, Toronto, Canada

**Background: **Latin America is currently facing its largest recorded mass migration due to the displacement of more than 7.03 million Venezuelans. Peru is the second largest recipient of Venezuelans, yet limited research has assessed health policy and responsiveness. Multi‐level, systemic inequities perpetuate vulnerability to HIV and limit care, especially for Venezuelan sexual and gender minorities (SGM) migrants. There exists an urgent need to monitor SGM needs and strengthen HIV health systems to improve quality and accessibility.

**Methods: **Between January and March 2023, we conducted interviews with key informants from public institutions, international cooperation agencies, civil society organizations, and grassroots organizations working with migrants and refugees (*n* = 16), and Venezuelan SGM migrants living in Lima, Trujillo and Piura (*n* = 26). Interviews explored health system access, HIV prevention and care, legal and policy barriers. Inductive and deductive analysis were informed by the Action Framework of the Working Group on Refugees and Migrants (GTRM) in Latin America.

**Results: **SGM participants had a median age of 39 years (IQR 26−37). Four barriers to accessing HIV prevention and care were described that affect Venezuelan SGM: (1) Health system constraints including underfunded and overburdened public systems that are ill‐equipped to handle the influx of migrants and insufficient training on SGM needs. Confusion among SGM migrants regarding if SGM services are available and where to access them; (2) Economic hardships experienced by SGM migrants increase vulnerability to HIV (e.g. sex work, housing instability, exploitation), juxtaposed against needed HIV prevention and care not prioritized due lack of insurance and out of pocket expenses; (3) Social stigma and structural violence, including the multiplicative impact of xenophobia, homophobia, transphobia, limits trust in public health systems; (4) Shifting migration policies and heterogenous processes within health establishments limits access to healthcare and disproportionality impacting SGM migrants.

**Conclusions: **Several needs of SGM migrants are not addressed by the Peruvian health system, due to legal, procedural and implementation barriers, leaving aside groups with high vulnerability to HIV. Considerable policy reform and programmatic efforts need to focus on the integration of this Venezuelan SGM into existing HIV prevention and care services and on the design of tailored interventions.

### OA0502 Overcoming challenges integrating injectable PrEP into care: Key strategies from the PILLAR study

T. Khan ^1^, A. Liu^2^, S. Perlotto^3^, K.L. Nelson^4^, L. Stassek^5^, J. Fang^5^, W.R. Lenderking^5^, D. Merrill^4^, D. Warren^6^, R. Moodley^7^, H. Swygard^4^, P. Budnik^7^, A. de Ruiter^7^, M. Czarnogorski^4^, N. Pilgrim^4^

^1^Fenway Health, Boston, United States, ^2^San Francisco Department of Public Health, San Francisco, United States, ^3^Yale School of Public Health, New Haven, United States, ^4^ViiV Healthcare, Durham, United States, ^5^Evidera, Bethesda, United States, ^6^GSK, Collegeville, United States, ^7^ViiV Healthcare, Brentford, United Kingdom

**Background: **Long‐acting cabotegravir (CAB LA) for PrEP offers a new HIV prevention option to people who could benefit from PrEP. CAB LA has appealing features (e.g. less frequent dosing), but there may be challenges to introducing an injectable option into real‐world settings. PILLAR is a phase IV implementation science study evaluating strategies for integrating CAB LA into care at existing PrEP clinics across the United States. We present results on facilitators and strategies to address challenges across seven implementation journey (IJ) stages of integrating CAB LA into care.

**Methods: **Seventeen clinics were randomized 2:1 into dynamic implementation (DI) (treatment) and routine implementation (control). DI clinics (*n* = 11) participated in monthly 1:1 implementation facilitation calls (FCs) and quarterly group FCs focused on exchanging knowledge, sharing implementation challenges and identifying solutions across stages of the IJ to support CAB LA integration. Sixty‐two monthly 1:1 FCs and six quarterly FCs were coded using the rapid qualitative analysis process described by Hamilton (2013) into five themes (barriers, facilitators, strategies, recommendations and support provided).

**Results: **Clinics spent the most effort in the following stages: patient selection, benefits verification, injection visits and continuation (Table). Challenges across the IJ included insufficient staffing, increased clinic‐pharmacy coordination, variable insurance coverage, CAB LA scheduling integration and required labs. Facilitators across the IJ included prior injectables experience, pharmacy expertise with HIV‐related medications, ease of physical storage, clinic effort to simplify scheduling, clinic‐patient rapport and patient enthusiasm to avoid pills. Helpful strategies across the IJ included clinic‐specific adaptations to resource management (e.g. tracking logs), visits (e.g. transportation, integrated care), injection scheduling (e.g. injection days, drop‐in appointments) and outreach through multiple mediums.

**Conclusions: **Findings suggest the initial challenges with CAB LA integration into standard of care are surmountable. Flexible injection scheduling, resource management and coordination, and regular clinic discussion of implementation learnings are important strategies in CAB LA delivery.[Table jia226351-tbl-0013]

**Table jia226351-tbl-0013:** OA0502

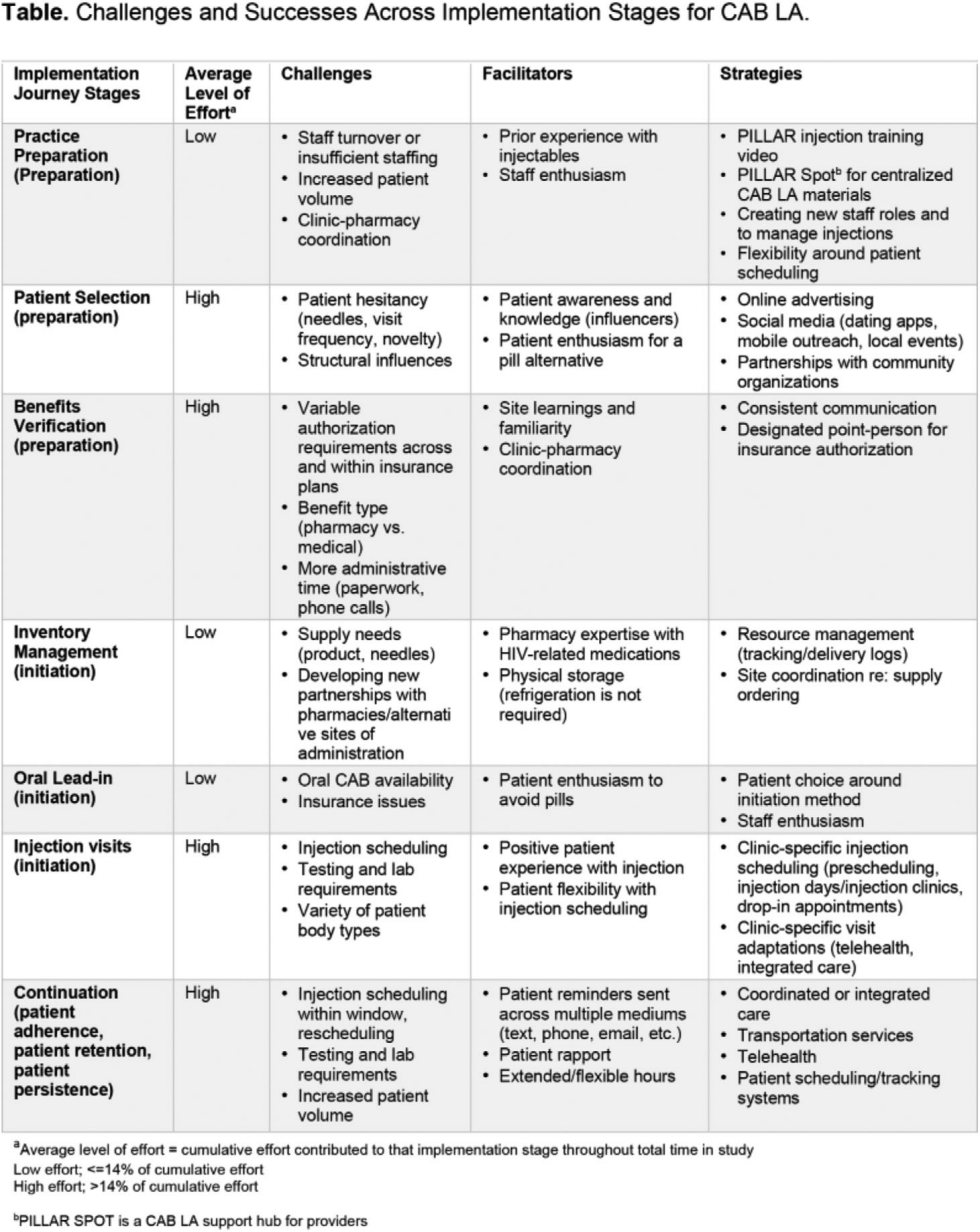

### OA0503 Low‐barrier, rapid Cabotegravir PrEP initiation and retention within a U.S. municipal health system

M.J. Heise ^1^, C. Camp^2^, J. Oskarsson^3^, M. Shiels^3^, F. Mayorga‐Munoz^1^, A. Chimezie^3^, J.Q. Nguyen^1^, K. Sassaman^1^, M. Gandhi^1^, M.A. Spinelli^1^

^1^University of California, San Francisco, Division of HIV, Infectious Disease and Global Medicine, San Francisco, United States, ^2^University of California, San Francisco, Division of Prevention Science, San Francisco, United States, ^3^Zuckerberg San Francisco General Hospital, Ward 86, San Francisco, United States

**Background: **Despite its potential to expand PrEP uptake, long‐acting cabotegravir (CAB‐LA) rollout needs to expand among underserved populations. The present study examines CAB‐LA implementation in a clinic system serving publicly insured patients.

**Methods: **The San Francisco Department of Public Health Primary Care Clinics is an integrated, municipal health system of 15 clinics. CAB‐LA was offered on a drop‐in basis, on the same day with a negative rapid HIV test and pending HIV viral load. On‐time injections were defined as +−7 days of target date. Logistic and Cox regression examined predictors of on‐time injection and discontinuation, respectively.

**Results: **Ninety‐six participants (mean = 37.6 years) taking CAB as PrEP from March 2022 to March 2024 were included in analyses; 64% cisgender male, 16% cisgender female, 10% nonbinary gender, 10% transgender female; 33% were Hispanic; 38% White, 14% Black, 7% Asian; 50% initiating PrEP for the first time. Most CAB‐LA injections (85%) were on‐time, and there were no differences in proportion of on‐time injections by age, gender or race (*p*s>0.116). CAB‐LA retention was 84.8% for the first 6 months (95% CI 80.9−88.9%), and mean time on CAB was 252 days (95% CI 210−293), Figure 1. In Cox regression, older age (scaled 10 years) was associated with retention (HR = 0.73, 95% CI 0.60−0.89, *p* = 0.002); cisgender men had higher retention than transgender women (HR = 0.46, 95% CI 0.23−0.94, *p* = 0.032), and nonbinary people had higher retention than both cisgender women (HR = 0.34, 95% CI 0.15−0.79, *p* = 0.012) and transgender women (HR = 0.29, 95% CI 0.11−0.78, *p* = 0.014). In addition, 8% of participants were also prescribed doxycycline post‐exposure prophylaxis (doxy‐PEP) for sexual health. No HIV seroconversions occurred.

**Conclusions: **Results provide evidence of the potential of CAB‐LA to expand PrEP uptake for diverse participant populations in a low‐barrier setting, with a high proportion initiating PrEP for the first time. Additional support may be needed for cisgender and transgender women CAB‐LA users to ensure retention, such as navigation or case management.[Fig jia226351-fig-0005]

**Figure 1 jia226351-fig-0005:**
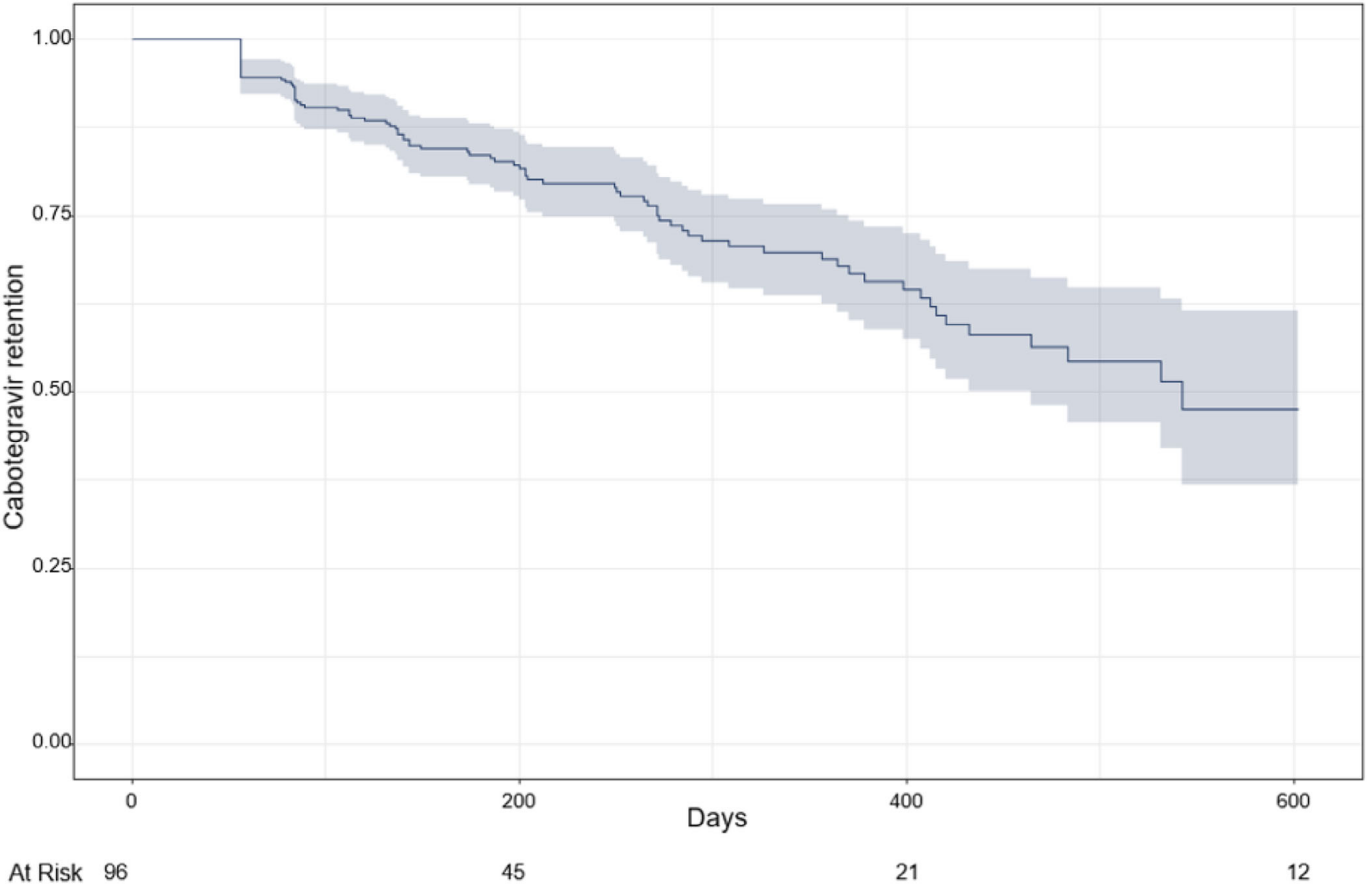
**OA0503**

### OA0504 Long‐acting injectable Cabotegravir (CAB‐LA) pilot implementation at primary healthcare level in resource‐limited settings: Early real‐world evidence from the USAID DISCOVER‐Health Project in Zambia

A.P. Ndhlovu ^1^, L. Kawanga^1^, D. Phiri^1^, M. Nyumbu^2^, M. Musonda^3^

^1^JSI USAID DISCOVER‐Health Project, Technical Services, Lusaka, Zambia, ^2^JSI USAID DISCOVER‐Health Project, Lusaka, Zambia, ^3^USAID/Zambia Mission, Health, Lusaka, Zambia

**Background: **Achieving the AIDS 2025 targets of less than 370,000 global annual HIV acquisitions from the baseline of 1.5 million in 2021 requires expanding combination HIV prevention choices beyond the currently existing arsenal. Long‐acting injectable Cabotegravir for HIV pre‐exposure prophylaxis (PrEP) has the potential to reduce the persistently high HIV incidence, although data on widespread implementation are scanty. John Snow Inc. through the DISCOVER‐Health Project supports the Zambian ministry of health in implementing the PEPFAR CAB‐LA pilot.

**Methods: **Following training of supervisors, health workers and community‐based volunteers, health facility preparation for CAB‐LA implementation and demand generation, CAB‐LA was provided to eligible individuals in line with the national Implementation plan, in two health facilities participating in the demonstration from 9th February 2024. Aggregates of client‐level data were extracted from the electronic database housing clients records, and de‐identified data by variables such as age, sex, population category for clients injected CAB‐LA or discontinued (by reasons) were analysed using WINPEPI.

**Results: **By 5th April 2024, 609 clients accessed CAB‐LA (median age = 24.4 years), with 425 (70%) of them being PrEP‐naïve and 184 (30%) transitioning from oral PrEP. Females were more likely to be PrEP‐naïve than males (OR = 1.36; 95% CI  = 0.94−1.95; *p = *0.051). Of all clients, 340 (55.8%) were females, 198 (32.5%) were adolescent girls and young women, 131 (21.5%) were adolescent boys and young men, 42 (6.9%) identified as key populations and 238 (39.1%) were other high‐risk populations. Additionally, 406/609 (67%) were due for second initiation injection and 371 (91%) were reinjected, 35 (9%) were pending reviews, 24 (3.9%) discontinued CAB‐LA, 22 (92%) of which were switched to oral PrEP, while 2 (8.3%) commenced antiretroviral therapy after acquiring HIV. Discontinuation reasons included hepatitis B virus 20 (83.3%), pregnancy 2 (8.3%), severe rash 1 (4.2%) and severe injection‐site pain 1 (4.1%).

**Conclusions: **CAB‐LA has the potential to prevent HIV acquisition through expanding PrEP uptake and persistence among females, adolescents and young people and other high‐risk populations. Robust laboratory support for eligibility screening is required in low‐resource settings for smooth implementation.

### OA0505 HIV pre‐exposure prophylaxis (PrEP) access preference among men who have sex with men in China: A discrete choice experiment

W. Huang ^1^, D. Stegmueller^2^, J.M. Sales^1^, G. Mi^3^, F. Yu^3^, Y. Liu^4^, W.S. Rice^1^, P.S. Sullivan^5^, J.J. Ong^6^, A.J. Siegler^1,5^

^1^Emory University, Department of Behavioral, Social, and Health Education Sciences, Atlanta, United States, ^2^Duke University, Department of Political Science, Durham, United States, ^3^Danlan Public Welfare, Beijing, China, ^4^China CDC, National Center for AIDS/STD Control and Prevention, Beijing, China, ^5^Emory University, Department of Epidemiology, Atlanta, United States, ^6^Monash University, Central Clinical School, Melbourne, Australia

**Background: **HIV pre‐exposure prophylaxis (PrEP) is highly effective but not widely used by men who have sex with men (MSM) in China. This study explores PrEP access preferences among Chinese MSM.

**Methods: **An online cross‐sectional survey with a discrete choice experiment (DCE) was distributed to MSM residing in Beijing and Chengdu in June 2023. Eligible study participants were above 18 years old, HIV negative or unknown status and were PrEP‐eligible based on criteria from a Chinese consensus statement for PrEP. The DCE explored attributes of PrEP modality (daily pill, on‐demand pill, injections, implants), clinical care model (same‐day, 2‐visit, telehealth prescription), medication pick‐up (clinics, pharmacy, home delivery), enhanced support (self‐management, smartphone app, text reminder) and cost. Data were analysed using mixed logit and latent class models.

**Results: **A total of 1013 MSM completed the survey with an average age of 31 years and a quarter (25%, 249/1013) had used PrEP. The most influential attributes were cost (relative importance = 65%), PrEP modality (28%), medication pickup (4%), enhanced support (4%) and clinical care model (0.2%). The most preferred way to access PrEP was no‐cost, on‐demand pill, medication home delivery, self‐management and telehealth. Four latent classes were identified. Men in Class 1 (“Modality matters,” 30% of participants) were more likely to be influenced by PrEP modality, followed by cost. Men in Class 2 (“All about cost,” 29%) were more likely to be influenced by cost. Men in Class 3 (“PrEP hesitant,” 19%) were more likely to prefer not using PrEP. Men in Class 4 (“Everything matters,” 23%) were more likely to value all attributes and to prefer long‐acting injectable PrEP. Men in Class 2 were more likely to report lower income level (coefficient = −0.54, *p*<0.05) compared to referent men (Class 4).

**Conclusions: **MSM in China have strong and unique preferences regarding PrEP: cost is a critical variable, especially important because the medication and clinical care are currently entirely unsubsidized in China. Preferences for on‐demand PrEP and home delivery indicate methods the healthcare system can utilize to best meet the needs of MSM, and factors that should be incorporated into future interventions.

### OA0506 Using stated preference methods to design gender‐affirming long‐acting PrEP programmes among transgender and nonbinary adults

A. Restar ^1^, M. Wilson‐Barthes^2^, E. Dusic^1^, D. Operario^3^, O. Galarraga^4^

^1^University of Washington, Department of Epidemiology, Seattle, United States, ^2^Brown University School of Public Health, Department of Epidemiology, Providence, United States, ^3^Emory University Rollins School of Public Health, Department of Behavioral, Social, and Health Education Sciences, Atlanta, United States, ^4^Brown University School of Public Health, Department of Health Services, Policy and Practice, Providence, United States

**Background: **Integrating gender‐affirming care with HIV biomedical prevention could help address the disproportionate HIV risk experienced by transgender and nonbinary (trans) adults. This discrete choice experiment (DCE) assesses and identifies the most important programming factors influencing the decisions of trans adults to use injectable long‐acting HIV pre‐exposure prophylaxes (LA‐PrEP).[Fig jia226351-fig-0006]

**Figure 1 jia226351-fig-0006:**
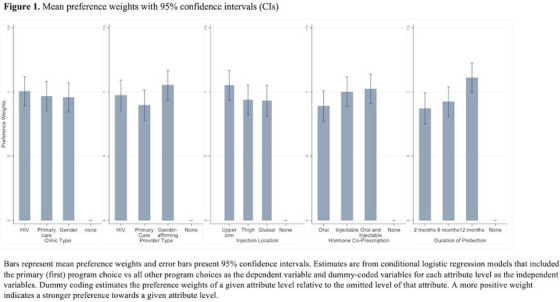
**OA0506**

**Methods: **From March to April 2023, *n* = 366 trans adults in Washington state chose between different four choice profiles that presented hypothetical programmes (each comprised of five attributes with four levels). We analysed ranked choice responses using a mixed rank‐ordered logit model.

**Results: **Respondents preferred to receive LA‐PrEP from a gender‐affirming care provider and a co‐prescription for oral and injectable hormones. Trans adults strongly favoured 12‐month protection and injection in the upper arm. No strong preferences emerged surrounding the type of health facility offering the gender‐affirming LA‐PrEP programme.

**Conclusions: **Our findings show that integrating and leveraging gender‐affirming health systems, particularly medical services such as hormone therapy, with HIV biomedical products like LA‐PrEP is strongly preferred and influential to trans adults’ decision to use LA‐PrEP. Leveraging choice‐based design experiments provides informative results that hold promise for optimizing gender‐affirming LA‐PrEP programming for trans adults.

### OA0602 *In vitro* characterization of mRNA‐mediated delivery of multispecific bNAbs for HIV‐1 immunoprophylaxis

C.J. O'Connor^1^, A. Ely^2^, M.A. Papathanasopoulos^1^, M.A. Killick ^1^

^1^University of the Witwatersrand, HIV Pathogenesis Research Unit, Johannesburg, South Africa, ^2^University of the Witwatersrand, Antiviral Gene Therapy Unit, Johannesburg, South Africa

**Background: **HIV‐1 broadly neutralizing antibodies (bNAbs) show great promise at both reducing viraemia and preventing HIV‐1 acquisition; however, their application may ultimately be limited due to high manufacturing costs and the requirement for combination‐based therapies. Here, we describe the development of *in vitro* transcribed (IVT)‐mRNA delivery of multispecific antibodies as a potential cost‐effective, passive immunisation strategy for prevention of HIV‐1 acquisition.

**Methods: **Previously described tandem single chain variable fragment (scFv) bispecifics (Bi‐scFv and Bi‐NAb) and heterologous heavy chain (knob‐into‐hole mutations) assembled tri‐specific (Tri‐NAb) antibodies combining VRC01/PGT121 and VRC01/PGT121/10e08 paratopes, respectively (>95% neutralization coverage *in vitro* [208 pseudovirus panel], with geometric mean IC50 titres <0.4 µg/ml), were selected for development. Antibody‐encoding DNA plasmid constructs, including a novel single open reading frame (sORF) polycistronic Tri‐NAb construct for comparison to Bi‐NAb/10e8 co‐transfection (Co‐T) strategy, engineered with T7‐IVT mRNA transcription compatibility were generated. Parental (PGT121, VRC01 and 10e08) and multispecific antibodies were expressed from DNA constructs by transient transfection in 293F cells, purified and biochemically characterized. Multispecific antibody‐encoding mRNA transcripts were IVT from linearized DNA, purified of dsRNA, enzymatically capped (5’ Cap 1 structure) and transfected into 293F cells. The functionality of the purified antibodies and mRNA‐transfected cell culture supernatants were assessed *in vitro* against a panel of 17 HIV‐1 tier 2 pseudoviruses with appropriate VRC01/PGT1231 sensitivity/resistance profiles.

**Results: **Expression and conformational assembly of purified multispecific antibodies from DNA constructs were confirmed and demonstrated improved neutralization coverage >70% coverage at IC80 <1 µg/ml, compared to the parental monoclonal antibodies PGT121 (59%), VRC01 and 10e8 (24% coverage at IC80 <1 µg/ml), as expected. Encouragingly, unpurified mRNA‐expressed multispecific antibodies matched the neutralization coverage of purified antibodies (94%), with sufficient multispecific antibody titres to generate inhibitory dilution factors conferring 80% neutralization (median ID80) >150: Bi‐scFv (1 427), Bi‐NAb (655), Tri‐NAb Co‐T (327) and Tri‐NAb sORF (166). Our data suggest that smaller, less complicated tandem scFv multispecific conformations (Bi‐scFv and Bi‐NAb) may be preferred for IVT‐mRNA delivery.

**Conclusions: **These data support the preclinical advancement of IVT‐mRNA encoding multispecific antibodies as a possible passive immunization strategy against HIV‐1 acquisition and require empirical determination of whether therapeutic titres are attainable *in vivo*.

### OA0603 Biodistribution of intra‐biliary injected Cu64 labelled HIV bNAbs in a non‐human primate model

M. Araínga ^1^, D.L. Hasselschwert^1^, D.E. Ferrell^1^, A.M. Carias^2^, Y. Thomas^2^, M.D. McRaven^2^, E. Allen^2^, K.A. Rogers^1^, N. Smith^1^, M. Louis^1^, J.A. Fontenot^1^, J.E. Goetzmann^1^, F.J. Villinger^1^, T.J. Hope^2^

^1^New Iberia Research Center (NIRC), New Iberia, United States, ^2^Northwestern University, Chicago, United States

**Background: **Copper‐64 (Cu64) is being used for positron emission tomography (PET) imaging, as diagnostic or targeting tool in cancer. HIV broadly neutralizing antibodies (bNAbs) are currently being evaluated as therapeutics in patients. Yet, in humans, pharmacokinetics of therapeutic Ab is mostly followed in blood with limited insight into actual distribution. Using the rhesus macaque model, we interrogated the distribution of isotope labelled HIV bNAbs using PET/CT. Surprisingly, we found rapid homing of specific HIV bNAbs to the liver and primarily the gall bladder following intravenous injection. Monitoring of faeces did, however, not show rapid elimination via the gastrointestinal tract. This prompted us to Cu64 labelled bNAbs directly into the biliary vesicle and follow their distribution over time.

**Methods: **Uninfected rhesus macaques were used for intra‐biliary delivery of Cu64‐DOTA or fluorescently tagged Ab via ultrasound‐guided location of the biliary vesicle. After injection of antibodies VRC01 and VRC07‐LS, several PET/CT scans were acquired between 5 minutes and 48 hours post injection. Plasma, urine and faeces were collected at the time of scans and radiation determined by scintillation. CT scans without contrast were used for anatomic localization and attenuation correction. MIM software was used to determine Cu64 signals. Tissues were also taken from macaques administered fluorescently tagged bNAbs and examined with deconvolution fluorescent microscopy.

**Results: **Intra‐biliary delivery of Cu64‐DOTA probes was successfully performed and radiation signal to evaluate biodistribution was determined. While the immediate signals were concentrated in the biliary vesicle, later time points showed transfer to the gut and uptake of the labelled bNAbs in small and large intestine walls. This was followed with rapid dissemination via lymphatic ducts at 4 hours post administration and beyond over time. Excretion via faeces or mucosal surfaces was limited over time. These data were corroborated with fluorescently tagged antibodies at the tissue level.

**Conclusions: **Our delivery system of Cu64 via biliary vesicle using NHP models was successful and illustrate a novel mechanism of antibody recirculation and persistence. This delivery approach can be used for a variety of targets in the field of infectious diseases and cancer and will facilitate furthering the HIV therapeutics field.

### OA0604 *In vivo* protection by a combination of engineered bnAbs against repeated high‐dose mixed‐SHIV challenges

R. Rouzeau ^1^, T. Silva De Assis^1^, I. Burton^2^, L. Shahin^1^, K. McKenney^1^, E. Chin^1^, H. Li^3^, S. Wang^3^, T. Sincomb^4^, K. Weisgrau^5^, J. Furlott^5^, M. Oakes^6^, N. Doria‐Rose^7^, A. Pegu^8^, B. Murrell^9^, A. Hessell^10^, E. Rakasz^5^, E. Landais^1^, G. Shaw^3^, J.H. Lee^1^, J. Jardine^11^, D. Sok^1^

^1^International AIDS Vaccine Initiative (IAVI), Neutralizing Antibody Center (NAC), San Diego, United States, ^2^Scripps Research, Immunology ‐ Burton Laboratory, San Diego, United States, ^3^University of Pennsylvania (UPenn), Division of Hematology/Oncology, G. Shaw Laboratory, Philadelphia, United States, ^4^International AIDS Vaccine Initiative (IAVI), San Diego, United States, ^5^University of Wisconsin‐Madison, Wisconsin National Primate Research Center, Madison, United States, ^6^University of California Irvine (UCI), Center for Complex Biological System, Irvine, United States, ^7^Vaccine Research Center (VRC), Bethesda, United States, ^8^Vaccine Research Center, Bethesda, United States, ^9^Karolinska Institutet, C1 Mikrobiologi, tumör‐ och cellbiologi, C1 Virology and Immunology, Solna, Sweden, ^10^Oregon Health and Science University, Portland, United States, ^11^Scripps Research, Immunology Department ‐ Jardine Laboratory, San Diego, United States

**Background: **The HVTN703/HPTN081 and HVTN704/HPTN085 trials demonstrated that broadly neutralizing antibodies (bnAbs) can protect against HIV‐1 infection but only if the infecting virus is neutralization sensitive to the antibody. Indeed, the clinical trials failed to show protective efficacy because of the high frequency of neutralization‐resistant isolates at the trial sites. Thus, a combination of bnAbs providing broader neutralization coverage is required to provide protection against the diversity of global isolates.

**Methods: **We sought to study the protection conferred by a cocktail of three enhanced bnAbs (ebnAbs) in rhesus macaques (RMs) from exposure to diverse simian‐human immunodeficiency viruses (SHIVs). We selected ePGT121v1, ePGDM1400v9 and VRC01.23J1 that were engineered for increased half‐life and improved neutralization breadth and potency. Three SHIVs were selected such that each ebnAb neutralizes 2/3 SHIVs, requiring all three mAbs for protection against a mix of all SHIVs. Starting at 5 days post‐infusion, RMs were given a high‐dose intrarectal challenge every 3 weeks, consisting of one or all three SHIVs. Animals were bled longitudinally to monitor infection and measure serum antibody concentrations, determined by ELISA. Long‐reads sequencing was employed to identify breakthrough viruses and the acquisition of resistance mutations.

**Results: **The first group received one ebnAb and was challenged with one neutralization‐sensitive SHIV. All animals except one were protected from 2 to 7 challenges. A second group of animals received one ebnAb but was challenged with all three SHIVs. In these animals, infection was established after one challenge in 75% animals, and seeded by the neutralization‐resistant SHIV in the mixture. A third group of RMs received all three ebnAbs and challenged with all three SHIVs. Overall, animals receiving one ebnAb became more rapidly infected when challenged with multiple SHIVs than to one SHIV, but ebnAb cocktails delay infection from exposure to diverse isolates. We are currently analysing breakthrough infections and ebnAb serum protective titres for the ebnAbs in cocktail.

**Conclusions: **The triple ebnAb cocktail protects against repeated high‐dose mixed SHIV challenges. Whether the concentration required for protection would be different for individual ebnAbs than for the cocktail is under assessment. These results will help inform future approaches to antibody‐mediated protection strategies.

### OA0605 Analytical treatment interruption (ATI) in Peru among MSM, trans and gender non‐conforming (GNC) individuals with early ART initiation +/− VRC01 proximate to HIV acquisition: Stakeholder engagement and early clinical data

S. Karuna^1^, J. Gallardo‐Cartagena ^2^, R. De La Grecca^1^, D. Grove^1^, J. MacRae^3^, J. Lama^3^, J. Montenegro‐Idrogo^2^, J. Hidalgo^4^, J. Hinojosa^5^, G. Broder^1^, J. Lucas^6^, P. Andrew^6^, L. Gama^7^, R. Tressler^8^, L. Soto‐Torres^9^, M. Cohen^10^, J. Currier^11^, J. Eron^12^, L. Corey^1^, J. Sanchez^2^, A. DeCamp^1^, K. Bar^13^, HVTN 804/HPTN 095/A5390 Study Team

^1^Fred Hutchinson Cancer Center, Vaccine and Infectious Disease Division, Seattle, United States, ^2^Universidad Nacional Mayor de San Marcos, Centro de Investigaciones Tecnológicas, Biomédicas y Medioambientales, Lima, Peru, ^3^Asociación Civil Impacta Salud y Educación, Lima, Peru, ^4^Asociación Civil Via Libre, Lima, Peru, ^5^Asociación Civil Selva Amazónica, Iquitos, Peru, ^6^FHI 360, Durham, United States, ^7^National Institute of Allergy and Infectious Diseases, National Institutes of Health, Vaccine Research Center, Bethesda, United States, ^8^National Institute of Allergy and Infectious Diseases, National Institutes of Health, HIV Research Branch, Division of AIDS, Bethesda, United States, ^9^National Institute of Allergy and Infectious Diseases, National Institutes of Health, HIV Research Branch, Division of AIDS, Rockville, United States, ^10^The University of North Carolina at Chapel Hill, Institute for Global Health and Infectious Diseases, Chapel Hill, United States, ^11^University of California, Department of Medicine, Division of Infectious Diseases, Los Angeles, United States, ^12^University of North Carolina at Chapel Hill, Department of Health Behavior, Chapel Hill, United States, ^13^University of Pennsylvania, Perelman School of Medicine, Philadelphia, United States

**Background: **ART prevents and treats but does not eradicate HIV; viraemia rebounds rapidly in most PWH upon ART cessation. Early ART initiation is associated with later ART‐free virologic control, as observed in 10−15% of males with Clade B HIV who initiated ART in early and acute infection. Broadly neutralizing anti‐HIV‐1 antibodies (bnAbs) may modulate immune responses to HIV. Our ATI was designed based on international consensus recommendations and in partnership with Peruvian community, investigators, healthcare providers and other stakeholders, to evaluate whether early ART +/− the VRC01 bnAb, present at the time of HIV acquisition, is associated with post‐treatment virologic control.[Fig jia226351-fig-0007]

**Figure 1 jia226351-fig-0007:**
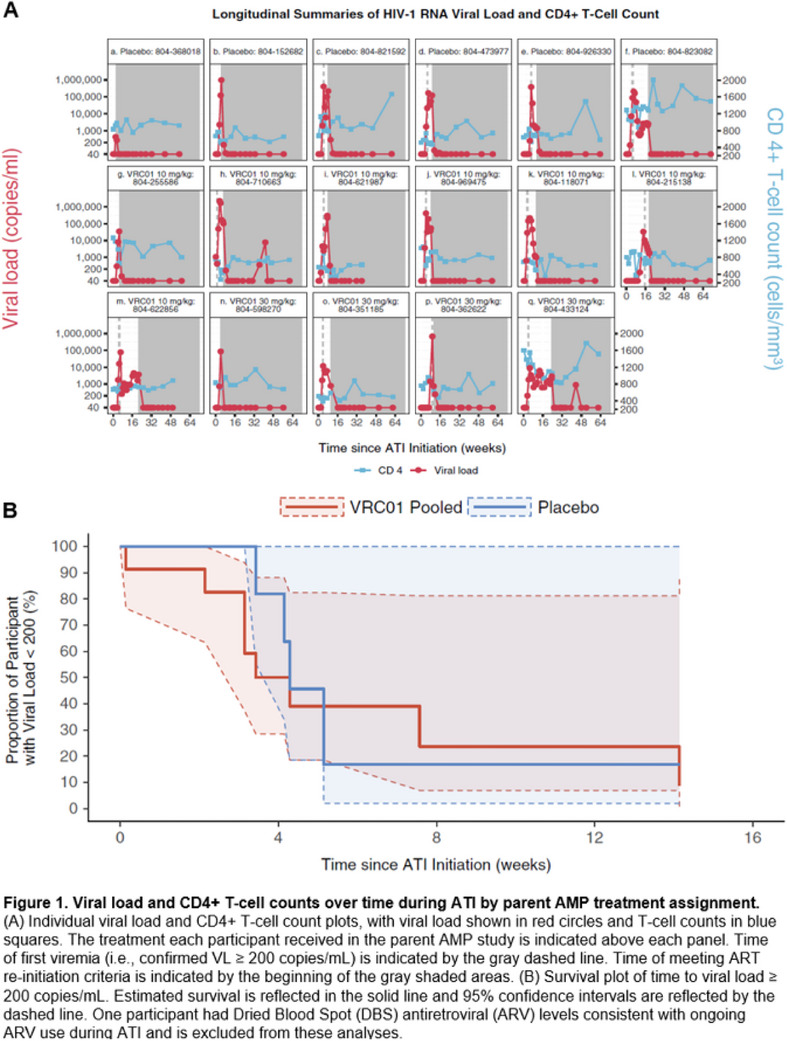
**OA0605**

**Methods: **ATI eligibility includes former HVTN 704/HPTN 085 Peru participants with estimated HIV acquisition within 8 weeks of receiving VRC01/placebo and early suppressive ART initiation for ≥1 year. Participants stopped ART and received frequent viral load (VL), T cell and clinical monitoring. ART re‐initiation criteria include confirmed CD4<250, VL>1000 for 4 weeks (wks) without 0.5log_10_ decline, [A1] [A2] HIV‐related syndrome or participant/clinician request.

**Results**: Fourteen MSM, one GNC and three transwomen enrolled in Peru. No HIV transmissions or ATI‐related SAEs or ≥Grade 3 AEs were observed. One ATI‐related AE was reported, Grade 2 acute retroviral syndrome; the participant's VL rose rapidly from 1450 to 679,000 and they immediately reinitiated ART. Nine STIs were diagnosed in eight participants during ATI. One participant had tenofovir levels consistent with use during ATI. Among the remaining 17 participants, ART re‐initiation criteria met included virologic (*n* = 12), clinician request (*n* = 1), participant request (*n* = 4) and HIV‐related syndrome (*n* = 1). Median time to confirmed VL>200 was 4.1 wks (range 0.1−14.1); median time to meeting ART re‐initiation criteria was 7.9 wks (range 2.7−23); these did not differ by VRC01 versus placebo receipt (Figure).

**Conclusions: **An ATI developed with local stakeholder engagement in Peru, following international consensus recommendations, was safe; however, participants did not exhibit post‐viral control.

### OA0606 Pharmacokinetic interaction assessment of the HIV broadly neutralizing monoclonal antibody VRC07‐523LS: A cross‐protocol analysis of three phase 1 HIV prevention trials HVTN127/HPTN087, HVTN130/HPTN089 and HVTN136/HPTN092

T.D. Chawana ^1^, S.R. Walsh^2^, L. Stranix‐Chibanda^1,3^, Z.M. Chirenje^1,4^, C. Yu^5^, L. Zhang^5^, K. Seaton^6^, T. Gamble^7^, S.T. Karuna^5^, P. Andrew^7^, M.E. Sobieszczyk^8^, S. Edupuganti^9^, C.L. Gay^10^, S.B. Mannheimer^8^, C.B. Hurt^10^, K.E. Stephenson^2^, L.L. Polakowski^11^, M. Yacovone^11^, S. Regenold^11^, C. Yen^11^, L. Gama^12^, D.H. Barouch^2^, R. Koup^12^, G.D. Tomaras^6^, O. Hyrien^5^, A.C. Roxby^5^, Y. Huang^5,13^

^1^University of Zimbabwe Clinical Trials Research Centre (UZ‐CTRC), Harare, Zimbabwe, ^2^Harvard Medical School, Boston, MA, United States, ^3^University of Zimbabwe, Faculty of Medicine and Health Sciences, Harare, Zimbabwe, ^4^University of California San Francisco, Bixby Centre for Global Reproductive Health, California, San Francisco, United States, ^5^Fred Hutchinson Cancer Centre, Vaccine and Infectious Disease Division, Seattle, United States, ^6^Duke University, Durham, NC, United States, ^7^FHI 360, Durham, NC, United States, ^8^Columbia University, New York, NY, United States, ^9^Emory University, School of Medicine, Atlanta, Georgia, United States, ^10^University of North Carolina at Chapel Hill, Institute for Global Health and Infectious Diseases, Chapel Hill, NC, United States, ^11^National Institutes of Health, Division of AIDS, National Institute of Allergy and Infectious Diseases, Bethesda, MD, United States, ^12^Vaccine Research Centre, National Institute of Allergy and Infectious Diseases, Bethesda, MD, United States, ^13^University of Washington, Department of Global Health, Seattle, WA, United States

**Background: **VRC07‐523LS is a safe, well‐tolerated broadly neutralizing monoclonal antibody (mAb) developed for HIV prevention. Within individual trials, pharmacokinetic (PK) features of VRC07‐523LS were not significantly different when administered alone or in combination with other mAbs. We combined data from three trials to increase the power to evaluate PK interactions and hypothesized that overall concentrations of VRC07‐523LS would be similar when administered combined or alone.[Table jia226351-tbl-0009]

**Table 1 jia226351-tbl-0009:** OA0606: Targeted maximum likelihood estimation (TMLE)‐adjusted pharmacokinetic features of VRC07‐523LS

PK features	Description	Single: Mean (95% CI)	Combination: Mean (95% CI)	Combination/Single: Ratio (95% CI)	Two‐sided raw *p*‐value	Two‐sided adjusted *p*‐value
CL (L/day)	Clearance from the central compartment	0.12 (0.11, 0.13)	0.13 (0.12, 0.13)	1.06 (1.00, 1.13)	0.056	0.17
Vc (L)	Volume of the central compartment	3.74 (3.26, 4.29)	4.66 (4.07, 5.34)	1.25 (1.14, 1.37)	<0.001	<0.001
Q (L/day)	Inter‐compartmental distribution clearance	0.30 (0.22, 0.41)	0.30 (0.22, 0.41)	1.00 (0.91, 1.11)	0.97	0.97
Vp (L)	Volume of the peripheral compartment	3.51 (3.07, 4.01)	3.89 (3.45, 4.38)	1.11 (1.04, 1.18)	<0.001	0.005
Distribution half‐life (day)	Length of time for serum concentration of the mAb to decrease by half in the distribution phase	3.71 (2.75, 5.01)	4.23 (3.15, 5.69)	1.14 (1.03, 1.27)	0.014	0.06
Elimination half‐life (day)	Length of time for serum concentration of the mAb to decrease by half in the elimination phase	47.67 (45.46, 49.98)	52.84 (50.17, 55.66)	1.11 (1.05, 1.17)	<0.001	0.002
Dose normalized steady‐state AUC (day/L)	Dose‐normalized area under the curve assuming a single IV administration	8.42 (7.96, 8.9)	7.94 (7.52, 8.39)	0.94 (0.89, 1)	0.056	0.17

**Methods: **This retrospective cross‐protocol analysis assessed three phase 1, randomized, multicentre trials of participants without HIV aged 18–50 years in the United States and Switzerland. We included participants receiving intravenous or subcutaneous VRC07‐523LS alone (HVTN127/HPTN087, *n* = 100), combined with PGT121, PGDM1400 or 10‐1074 (HVTN130/HPTN089, *n* = 26), or combined with PGT121.414.LS (HVTN136/HPTN092, *n* = 20). An open, two‐compartment population PK model was used to describe serum concentrations of VRC07‐523LS. We compared PK parameters estimated using the targeted maximum likelihood estimation method to account for potential differences in participants’ characteristics between groups (combination vs. single), including age, sex‐at‐birth, body weight and creatinine clearance.

**Results: **Median age was both 28 years for the combination and single groups, with 52% and 61% reporting female sex‐at‐birth, median body weight of 71 and 76 kg, and median creatinine clearance of 120 and 122 ml/minute, respectively. No significant differences in VRC07‐523LS clearance rate, inter‐compartmental clearance, distribution half‐life or area under the concentration curve were observed between combination and single groups. However, the mean covariate‐adjusted central volume of distribution (Vc) was 1.25 times larger (4.66 vs. 3.74 L, adjusted‐*p*<0.001), peripheral volume of distribution (Vp) was 1.11 times higher (3.89 vs. 3.51 L, adjusted‐*p* = 0.005) and elimination half‐life was 1.11 times longer (53 vs. 48 days, adjusted‐*p* = 0.002) for combination versus single administration.

**Conclusions: **Biodistribution of VRC07‐523LS differed when administered combined with other mAbs versus alone, but overall concentration‐over‐time was not impacted. This is important for planning future trials of VRC07‐523LS with new mAb formulations.

### OA0702 Dysregulation of intestinal CX3CR1^+^ macrophages homeostasis contributes to inflammation and viral persistence in SIV‐infected cynomolgus macaques

S. Hua^1^, K. Benmeziane^1^, D. Desjardins^1^, N. Dimant^1^, N. Dereuddre‐Bosquet^1^, F. Relouzat^1^, V. Avettand‐Fenoël^2^, A. Sáez‐Cirión^3^, R. Le Grand^1^, M. Cavarelli ^1^

^1^Université Paris‐Saclay, Inserm, CEA, Center for Immunology of Viral, Auto‐immune, Hematological and Bacterial diseases (IMVA‐HB/IDMIT), Fontenay‐aux‐Roses & Le Kremlin‐Bicêtre, France, ^2^Université Paris Cité; INSERM, U1016; CNRS, UMR8104, Paris, France, ^3^Institut Pasteur, Université Paris Cité, Viral Reservoirs and Immune Control Unit, Paris, France

**Background: **Chronic HIV‐1 and SIV infections are marked by persistent immune activation and inflammation, despite antiretroviral therapy (ART). In this study, we aimed to elucidate the contribution of intestinal CX3CR1^+^ macrophages (MΦs) to the immune dysregulation observed during chronic SIV infection and its implications for HIV‐1 persistence following antiretroviral treatment interruption (ATI).

**Methods: **We investigated the dynamics of mucosal CX3CR1^+^ MΦs and T cell subsets in a cohort of Cynomolgus macaques during chronic SIV infection and subsequent ATI.

**Results: **Analysis of mucosal specimens revealed a notable accumulation of pro‐inflammatory MΦs in infected animals, which positively correlated with viral load levels. Interestingly, animals classified as post‐treatment controllers (PTCs) exhibited restoration of MΦ homeostasis, suggesting a potential role in viral control post‐therapy.

Furthermore, our study unveiled significant alterations in mucosal T‐cell populations during chronic SIV infection. Total CD4^+^ T cells, particularly Treg cells, were depleted in the colonic mucosa of infected animals, with partial restoration observed in PTCs but not in non‐controller animals. Conversely, Th1 CD4^+^ cells showed an increase in SIV‐infected animals, while a trend towards reduction in Th17 cells was observed. Of interest, the accumulation of pro‐inflammatory MΦs correlated with the depletion of Th17 cells. Additionally, activated phenotypes were evident in CD4^+^ T cells from infected and non‐controller animals, as indicated by the upregulation of PD‐1, HLA‐DR and Ki67 markers. Neutrophils, recognized as key mediators of inflammation, also exhibited activation in SIV‐infected animals, with a notable increase in CD66^+^CD32a^+^ cells. These activated neutrophils were positively associated with the accumulation of pro‐inflammatory MΦs and faecal calprotectin levels, indicative of intestinal epithelial damage.

**Conclusions: **Overall, our findings provide novel insights into the complex interplay between mucosal immune cell populations during chronic SIV infection, shedding light on the mechanisms underlying immune dysfunction and gut epithelial damage observed in progressive HIV/SIV infections and their contribution to viral rebound at ATI. As such, strategies able to preserve and/or regenerate the functions of CX3CR1^+^ macrophages are critically needed in future HIV cure research.

### OA0703 Single‐cell analyses reveal that monocyte gene expression impacts HIV‐1 reservoir size in acutely treated cohorts

P. Ehrenberg^1^, A. Geretz^1,2^, T. Izumi^1,2,3^, M. Volcic^4^, L. Yum^1,2^, D. Paquin‐Proulx^1,2^, M. Creegan^1,2^, C. Sacdalan^5^, N. Phanuphak^5^, R. Apps^6^, E. Daar^7^, J. Mellors^8^, S. Vasan^1,2^, N. Michael^9^, F. Kirchhoff^4^, R. Thomas ^1^

^1^Walter Reed Army Institute of Research, MHRP, Silver Spring, United States, ^2^HJF, Bethesda, United States, ^3^American University, Washington, DC, United States, ^4^Ulm University Medical Center, Ulm, Germany, ^5^Institute of HIV Research and Innovation, Bangkok, Thailand, ^6^National Institutes of Health, Bethesda, United States, ^7^Lundquist Institute at Harbor‐UCLA Medical Center, Torrance, United States, ^8^University of Pittsburgh, Pittsburgh, United States, ^9^Walter Reed Army Institute of Research, CIDR, Silver Spring, United States

**Background: **Host genetic variation impacts HIV‐1 susceptibility and disease progression in antiretroviral therapy (ART)‐naïve individuals. Similarly, HIV reservoir sizes can vary considerably between ART‐treated people living with HIV (PLWH). Here, using unbiased single‐cell transcriptomics, we investigated if host gene expression differences in PBMC cell subsets from PLWH influence total reservoir size during suppressive ART.

**Methods: **Samples from 14 PLWH, diagnosed at Fiebig stage 3, from an acutely treated cohort in Thailand demonstrated effective viral suppression and significantly different HIV‐1 DNA reservoir sizes at 48 weeks after ART initiation. PBMCs from week 48 samples were analysed by single‐cell RNA sequencing (scRNA‐seq) to identify differentially expressed genes (DEGs) that associated with reservoir size phenotype. Significant findings were validated in an independent acutely treated cohort, comprised of 38 participants from the United States with different genetic backgrounds and HIV‐1 subtype. Host factor effects on HIV‐1 infectivity, proviral transcription and memory CD4^+^ T‐cell population frequencies were studied using *in vitro* functional assays.

**Results: **DEGs and enriched pathways demonstrated increased monocyte activity in participants with lower HIV‐1 cell‐associated DNA levels. *IL1B* expression in CD14^+^ monocytes showed the greatest fold‐difference associating with smaller reservoir in independent cohorts. Modelling interactions with cell population frequencies showed that monocyte *IL1B* expression correlated inversely with reservoir size in the context of higher frequencies of central memory CD4^+^ T cells suggesting an indirect IL1B effect via modulation of the cell population preferentially used for reservoir establishment. Our *in vitro* results suggest that IL1B may contribute to decreased reservoir size *in vivo* during AHI via NF‐kB activation: (1) induction of proviral transcription to “flush‐out” the reservoir via natural latency reversing activity; (2) reduction of virus spread through activation of antiviral responses; and (3) reduction of the frequencies of specific CD4^+^ T‐cell memory populations.

**Conclusions: **Collectively, unbiased high‐throughput scRNA‐seq analyses identified an effect of monocyte transcriptomic variation on HIV‐1 reservoirs in individuals initiating ART during AHI. Specifically, *IL1B* expression in monocytes associated with lower HIV‐1 reservoir size and this could be via activation of NF‐kB. Unbiased single‐cell omics approaches can identify novel pathways that reduce the reservoir and have therapeutic implications for HIV‐1 cure.

### OA0704 Association of innate cells activation and mucosal homing potential with HIV acquisition in Thai HIV‐exposed individuals

K. Machmach Leggat ^1,2^, K.F. N'Guessan^1,2^, D. Kim^1,2^, I. Swafford^1,2^, B. Slike^3^, L.A. Eller^1,2^, J. Ake^1^, M. Robb^1,2^, S.J. Krebs^1^, D. Paquin‐Proulx^1,2^

^1^U.S. Military HIV Research Program, Walter Reed Army Institute of Research, Silver Spring, United States, ^2^Henry M. Jackson Foundation for the Advancement of Military Medicine, Bethesda, United States, ^3^Henry Jackson Foundation for the Advancement of Military Medicine/Walter Reed Army Institute of Research, Walter Reed Army Institute of Research, Silver Spring, United States

**Background: **HIV‐exposed seronegative individuals (HESN) are a unique model to study immune factors impacting HIV‐1 acquisition. Increased NK cell activity has been correlated with reduced HIV acquisition, suggesting the involvement of NK cells in decreasing the risk of HIV‐1 acquisition. Other studies have identified markers of HIV susceptibility such as the gut‐homing integrin α4β7, whose expression in memory CD4^+^ T cells is associated with increased susceptibility to HIV acquisition. Here, we compare circulating innate and CD4 T cells phenotype in HESN and HIV‐exposed seroconverted (HESC) participants prior to HIV‐1 acquisition.

**Methods: **Cryopreserved PBMCs from Thai participants in the Early Capture HIV Cohort (RV217) were used to characterize innate and CD4 T cells in 25 HESC and 75 HESN participants. HESC pre‐acquisition samples were matched for collection time, age, gender and risk behaviour with three HESN. Two flow cytometry panels were developed to investigate NK and T‐cell subsets.

**Results: **NK cells from HESN were less activated (HLA‐DR+%, *p* = 0.003), showed a higher potential to migrate to the gut (α4β7+%, *p* = 0.010) and lower expression of the inhibitory receptor ILT‐2. Moreover, we observed an increased frequency of α4β7 expressing CD4^+^ memory T cells (*p* = 0.003) and α4β7 expressing invariant natural killer T (iNKT) cells in HESC (*p* = 0.003), more specifically on the CD4^+^ subset. iNKT cells from HESC also had increased expression of T‐bet. Random forest analysis showed that HLA‐DR and α4β7 expression by NK cells have the strongest association in predicting acquisition status. Moreover, logistic regression model analysis including four variables: HLA‐DR+ NK, CD16^+^ NK, α4β7^hi^ memory CD4^+^ T and T‐bet+ iNKT cells, showed a sensitivity of 64%, a specificity of 95.83% and an accuracy of 87.63% in identifying HESC and HESN.

**Conclusions: **Our data suggest that in addition to an increased frequency of HIV target cells such as CD4^+^ memory T and CD4^+^ iNKT cells expressing α4β7, NK cells from HESC participants might have an exhausted and dysfunctional phenotype with a decreased ability to migrate to the gut mucosa. It is possible that this dysfunctional phenotype increases the risk of HIV‐1 acquisition relative to a more favourable quiescent NK cell profile as observed in the HESN participants.

### OA0705 Single‐cell RNA sequencing analysis of human milk myeloid cells as potential vehicles of and defenders against vertical transmission of HIV

R. Powell ^1^, X. Yang^1^, D. Chowhan^1^, S. Shroff^1^, A. Fox^1^, K. Beaumont^1^

^1^Icahn School of Medicine at Mount Sinai, Medicine, New York, United States

**Background: **Approximately 150,000 HIV transmission events occur annually via human milk; yet, the risk of HIV infection via human milk‐feeding is <15%, suggesting milk itself is partially protective. Infants ingest ∼10^5^–10^8^ milk leukocytes daily, and our recent studies demonstrated that milk leukocytes, including unclassifiable CD14^‐^ myeloid cells, perform antibody‐dependent cellular phagocytosis (ADCP) of HIV, calling for their comprehensive analysis in the context of HIV transmission.

**Methods: **Cells were purified from milk pumped within the previous 12 hours by nine HIV‐negative donors in NYC. Live CD45+DRAQ5+ cells were sorted for scRNAseq. Single cells were encapsulated and cDNA prepared on a10x Genomics Chromium instrument. Barcoded cDNA was amplified, fragmented and subjected to end‐repair, poly A‐tailing, adapter ligation, and 10x‐specific sample‐indexing. Data were processed using the v6.1 Cell Ranger Software Suite and v4.0 Loupe Cell Browser, prior to analytics using the Seurat single‐cell analysis R package (v4.0).

**Results: **Two thousand one hundred and seventy‐nine milk myeloid cells were analysed, and 10 populations were defined based on differential gene expression. Clusters were then categorically annotated, which indicated five distinct non‐granulocyte myeloid subsets. Importantly, annotation with markers derived from PBMCs indicated that milk myeloid cells are largely unique and do not align with those found in blood. Myeloid subsets were found to express CD4 (25%−75% expression by all five subsets) with highly dominant CXCR4 (50%−100% expression) compared to CCR5. Notably, Fc‐gamma receptor 2A was expressed by ∼100% of all subsets, alongside varying levels of FcGR 1A/2B/3A. Two subsets exhibited 50%−75% expression of Fc‐alpha receptor, suggesting these cells may be elicited to perform Fc‐mediated anti‐viral functions not only by IgG isotypes, but also by monomeric (serum‐derived) and secretory (mucosal) IgA (which comprises ∼90% of milk antibody).

**Conclusions: **These data indicate that milk myeloid cells are unique and highlight the need for their comprehensive analysis among donors living with HIV, including the assessment of HIV susceptibility, Env expression and Fc‐mediated functionality. This will facilitate the design of a therapeutic vaccine aimed to eliminate HIV transmission via human milk‐feeding that would effectively target infected milk myeloid cells (the vehicles of transmission), while eliciting antibody isotypes/subclasses that augment Fc‐mediated function of protective milk myeloid cells (the defenders against transmission).

### OA0902 Field performance evaluation of dual rapid HIV and syphilis assays among pregnant women attending antenatal care at selected health facilities in Uganda

J. Kyokushaba ^1^, V. Kisone^1^, A. Namakula^1^, L. Kisaakye^2^

^1^Ministry of Health, Laboratory, Kampala, Uganda, ^2^Ministry of Health, PMTCT, Kampala, Uganda

**Background: **The Global Plan Towards Elimination of New HIV Infections in Children and keeping their mothers alive was launched in 2009 setting a series of ambitious targets with unprecedented investments in the prevention of mother to child transmission (PMTCT) of HIV. Uganda developed the triple elimination strategy to reduce mother‐to‐child transmission (MTCT) of HIV, syphilis, and hepatitis B and innovative new approaches to service delivery that included triple testing for these infections. A novel point‐of‐care immunochromatographic test for dual diagnosis of both HIV and syphilis as well as hepatitis B became a facilitator for this approach. This paper evaluated the performance of the SD Bioline HIV/syphilis Duo and Standard Q HIV/syphilis Comb in 30 health facilities in Uganda.

**Methods: **This was a cross‐sectional study involving 18,924 pregnant women with a mean age of 25.8 years at 30 health facilities who were tested with the Standard Q HIV/syphilis combo to assess its field performance in comparison with the SD Bioline HIV/syphilis Duo in antenatal clinics. Sensitivity and specificity for HIV and syphilis were determined in comparison with the National HIV testing algorithm (Determine, Statpack and SD Bioline), and the Treponema pallidum particle agglutination assay for HIV and syphilis, respectively, as the reference standards. Acceptability, ease of use and feasibility were also assessed using self‐reported questionnaires.

**Results: **For Standard Q, the HIV sensitivity was 96.1% (95% CI: 86.5−99.5) and the specificity was 100%. Syphilis sensitivity was 100%, and specificity was 99.5% (95% CI: 98.6−99.9). For SD Bioline, HIV sensitivity was 89.4% (95% CI: 86.1−92.0) and specificity was 96.3% (95% CI: 95.3−97.1); syphilis sensitivity was 66.2% (95% CI: 59.4−72.4) and specificity was 97.2% (95% CI: 96.4−97.9). Therefore, Standard Q meets the diagnostic expectations with even better performance characteristics than the SD Bioline kits. Both rapid diagnostic tests were assessed as highly acceptable and feasible by health workers.

**Conclusions: **The excellent performance of the Duo Kits has facilitated the integration of syphilis testing and treatment into the already established HIV prevention programme contributing to the dual HIV and syphilis elimination goal. This will enable more women to be diagnosed with HIV and syphilis so that they can access treatment and prevent transmission to their children.

### OA0903 Same day testing and treatment for STIs in adolescents: Results of a pilot randomized controlled trial in South Africa

D. Travill ^1^, D. Machemedze^1^, P. Zwane^1^, T. Mokhele^1^, L. Makhale^1^, Z. Sokhela^1^, M. Maraba^1^, S. Delany‐Moretlwe^1^

^1^Wits RHI, University of Witwatersrand, Research Team, Johannesburg, South Africa

**Background: **Laboratory‐based testing for sexually transmitted infections (STIs) is not routine in South Africa, but studies suggest that clients would prefer point‐of‐care (POC) STI testing, and that same day testing may lead to better treatment completion and reduce STI transmission. We conducted a pilot randomized trial to evaluate whether same day testing and treatment for positive tests (SD) improved treatment completion rates.

**Methods: **The study was conducted in an adolescent‐friendly primary healthcare facility in Johannesburg from August 2023 to February 2024. Participants 18 years or older, sexually active in the past 3 months, at elevated risk for STIs and able to provide a means of contact were included. Randomization was by day 1:1 to either SD or standard laboratory testing with results return and treatment in 24−48 hours. Urine samples were tested for *C. trachomatis* (CT) and *N. gonorrhoeae* (NG) using GeneXpert™. The primary outcome of treatment completion within 30 days was assessed by study arm using chi‐squared tests. Time to treatment by study arm was assessed by log‐rank test. Cox proportional hazards models assessed factors associated with time to treatment.

**Results: **Of 355 screened, 350 were enrolled, and 348 with valid tests were analysed. Median age was 21 years (IQR, 20−23), and 10% (33/348) had STI symptoms at that visit. Overall, 40% (138/348) had confirmed CT/NG; 79% (109/138) were asymptomatic. In the ITT analysis, 97% (65/67) in the SD group compared to 92% (65/71) in the standard group completed treatment within 30 days (risk ratio [RR] 1.06, 95% CI 0.98−1.15). In the per‐protocol analysis, treatment completion rates were higher in the SD group (98% [63/67] SD vs. 92% [49/71] standard; RR 1.36, 95% CI 1.15−1.61). Median time to treatment completion was significantly longer in the standard group (0 [SD] vs. 3 [standard] days, *p*<0.0001). Being asymptomatic prolonged time to treatment (adjusted hazard ratio 2.38; 95% CI 1.48−3.85).

**Conclusions: **Curable STIs were high and frequently asymptomatic or unrecognized in this adolescent population. With adequate counselling and follow up, treatment completion rates are high but can be improved with same day testing and treatment.

### OA0904 The impact of expedited partner therapy on repeat STI positivity among adolescent girls and young women using oral PrEP in Johannesburg, South Africa

T. Palanee‐Phillips ^1^, R. Howett^2^, K. Reddy^1^, N. Ndlovu^1^, L. Kew^1^, H. Ishmail^1^, R. Kgoa^1^, N. Sigcu^1^, N. Dladla^1^, J. Ross^2^, J. Velloza^3^, J. Balkus^2^

^1^University of the Witwatersrand, Wits RHI, Johannesburg, South Africa, ^2^University of Washington, Department of Epidemiology, Seattle, United States, ^3^University of California, San Francisco, Division of Global Health and Infectious Disease Epidemiology, Department of Epidemiology & Biostatistics, San Francisco, United States

**Background: **Adolescent girls and young women (AGYW) using pre‐exposure prophylaxis (PrEP) for HIV prevention are at risk for sexually transmitted co‐infections (STIs). In alignment with South African national STI treatment guidelines, syndromic STI management is implemented at PrEP visits. Asymptomatic AGYW miss treatment via this approach. The ARISE prospective cohort study is assessing point‐of‐care (POC) diagnostics, treatment and expedited partner therapy (EPT) for STI management among AGYW using PrEP in Johannesburg.

**Methods: **Between February 2022 and December 2023, HIV‐negative, non‐pregnant, sexually active AGYW aged 18−25 years interested in or already using PrEP underwent POC STI testing for *C. trachomatis* (CT) and *N. gonorrhoeae* (GC) by GeneXpert; *T. vaginalis* (TV) by OSOM. Those testing STI+ received treatment and were eligible to enrol. Participants were offered EPT paired with counselling to support delivery. Decliners were offered partner referral cards. Participants returned for a test‐of‐cure (TOC) visit 1 month later. We summarize STI concordance (+ for the same STI at enrolment and TOC) among those who delivered EPT versus those who declined or were not able to deliver EPT by partnership type (primary vs. non‐primary).

**Results: **Among 305 AGYW enrolled, 271 (89%) had a primary or non‐primary partner at enrolment and returned for the TOC visit. Median age was 21 years and baseline STI prevalence was 79% for CT, 19% for GC and 19% for TV, with 35% reporting STI‐related symptoms. Among participants with a primary sexual partner, 226/256 (88%) accepted and delivered EPT. Among participants with a non‐primary partner, 39/69 (57%) delivered EPT. Concordant STI+ results by EPT delivery status and partnership type are presented in the Table.

**Conclusions: **Among AGYW using PrEP and who received POC STI testing, EPT acceptance and successful delivery to primary partners was high; few were concordant STI+. Diagnostic STI testing and EPT should be considered as part of STI prevention packages for AGYW.[Table jia226351-tbl-0006]

**Table jia226351-tbl-0006:** OA0904

Positive STI result at enrolment	Concordant STI result at test of cure			
	EPT for primary partner (*N* = 256)		EPT for non‐primary partner(s)^a^ (*N* = 69)	
	Accepted and delivered	Did not accept, or accepted but did not deliver	Accepted and delivered	Did not accept, or accepted but did not deliver
	(*N* = 226)	(*N* = 30)	(*N* = 39)	(*N* = 30)
*Chlamydia trachomatis*, *n*/*N* (%)	19/179 (11%)	3/22 (14%)	4/26 (15%)	3/30 (10%)
*Neisseria gonorrhoeae*, *n*/*N* (%)	3/43 (7%)	0/4 (0%)	0/13 (0%)	0/3 (0%)
*Trichomonas vaginalis*, *n*/*N* (%)	4/46 (9%)	1/6 (17%)	1/10 (10%)	1/4 (25%)

^a^For the non‐primary partner analysis, acceptance was defined as accepted EPT with ≥1 non‐primary partner(s) as intended recipient(s), and delivery was defined as delivered EPT to ≥1 non‐primary partner(s). Participants who accepted and/or delivered EPT according to this definition, might not have accepted and/or delivered EPT to all their non‐primary partners.

Abbreviations: EPT, expedited partner therapy; STI, sexually transmitted infection.

### OA0905 PrEP use and associations with STI incidence among young cisgender men and transgender women who sell sex in Bangkok and Pattaya, Thailand

V.H. Vu ^1^, B.W. Weir^1,2^, A.L. Wirtz^1^, J.P. Ngo^1^, P. Sullivan^3^, C. Beyrer^1,4^, the Combination Prevention Effectiveness (COPE) Study Team

^1^Johns Hopkins Bloomberg School of Public Health, Center for Public Health and Human Rights, Department of Epidemiology, Baltimore, United States, ^2^Johns Hopkins Bloomberg School of Public Health, Department of Health, Behavior & Society, Baltimore, United States, ^3^Emory University Rollins School of Public Health, Department of Epidemiology, Atlanta, United States, ^4^Duke University, Duke Global Health Institute, Durham, United States

**Background: **Sexually transmitted infections (STIs) are an important consideration when it comes to implementing pre‐exposure prophylaxis (PrEP), especially in young men who have sex with men (MSM) and transgender women (TGW). STI incidence has been associated with declines in condom use while on PrEP. Our analysis explores the relationship between STIs and these interventions among sex workers.

**Methods: **The Combination Prevention Effectiveness (COPE) study, an open‐label combination HIV‐prevention implementation trial, evaluated the effectiveness of PrEP in 846 participants between 2017 and 2020, accumulating 867.1 person‐years of follow‐up. Participants had the option to start, stop and restart TDF/FTC PrEP (Truvada®, Gilead Sciences, Inc.) at any point during follow‐up. Routine testing was done for rectal *Neisseria gonorrhoeae* and *Chlamydia trachomatis* by nucleic acid amplification from rectal swab samples at study enrolment, 6‐month‐interval visits and additionally at 3‐month visits if optional testing was requested. Demographic and behavioural variables from the survey responses at each study visit were assessed for association with incidence of STI. Cox proportional hazards models were used to assess the association between variables and the time to first incidence of STI after baseline.

**Results: **At enrolment, 151 (17.1%) of the study participants were positive for at least one STI. The incidence rate of chlamydia and gonorrhoea was 22.76 per 100 person‐years (95% CI: 19.29−26.66) and 8.47 (95% CI: 6.42−10.98), respectively. Participants who reported PrEP use had a higher incidence of STIs, while condom use showed a protective effect against incidents of STIs.

**Conclusions: **Our findings indicate high STI incidence and prevalence in Thai gender‐diverse populations involved in commercial sex. Although PrEP use might suggest behavioural disinhibition, it is important to remember that STI risk behaviours is an indicator for PrEP use. Our data underline the need for a comprehensive intervention package that includes targeted testing, safer‐sex education, free condoms and PrEP provision.[Fig jia226351-fig-0008]

**Figure 1 jia226351-fig-0008:**
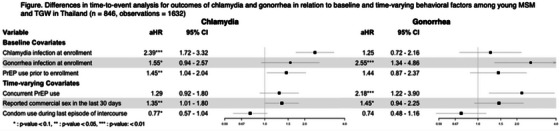
**OA0905**

### OA0906 Willingness and uptake of mpox vaccine among sexual minority men (SMM) in Latin America: Examining differences by HIV serostatus

C. Hong ^1^, A. Garner^2^, A. Bautista^3^, I. Holloway^4^

^1^University of Connecticut, School of Social Work, Hartford, United States, ^2^MPact Global Action, Los Angeles, United States, ^3^MPact Global Action, Mexico City, Mexico, ^4^University of California Los Angeles, Department of Social Welfare, Los Angeles, United States

**Background: **Sexual minority men (SMM) were disproportionately affected by the 2022 global mpox outbreak, and there is a growing concern about a possible increase in cases during summer 2024. Lessons learned from the recent outbreak highlighted the importance of vaccination strategies to mitigate the spread of the virus among at‐risk populations, especially among subgroups with intersecting vulnerabilities, such as those living with HIV. The current study aims to describe differences in willingness and uptake of the mpox vaccine by HIV status among an online sample of SMM in Latin America.

**Methods: **Data were collected through Hornet, a geosocial networking application in late 2023. SMM residing in Latin American countries were invited to complete a self‐report survey regarding the impact of the recent mpox outbreak. Descriptive statistics were used to present the rates of mpox diagnoses and willingness and uptake of the mpox vaccine. We conducted bivariate analyses to detect differences in these outcomes by self‐reported HIV serostatus.

**Results: **Among the 5141 SMM from 25 Latin American countries, the average age was 33.1 years (SD: 10.8), and 21.9% were living with HIV. A small percentage of study participants (3.8%) reported being diagnosed with mpox; 7.5% said they had been vaccinated for mpox. The majority (83.7%) of participants expressed willingness to be vaccinated if offered the vaccine. SMM living with HIV were more likely to report being diagnosed with mpox (10.7% vs. 2.4%, *p* < 0.001) and having been vaccinated (12.6% vs. 6.4%, *p* < 0.001). They were also more willing to receive the mpox vaccine (88.3% vs. 82.7%, *p* < 0.001). Lastly, those living with HIV were more likely to report perceived or experienced mpox stigma (14.4% vs. 7.9%, *p* < 0.001).

**Conclusions: **Our study reveals disparities in mpox diagnoses by HIV serostatus and highlights critical gaps in vaccine uptake among a high‐risk population in Latin America. The findings emphasize the urgent need for tailored vaccination strategies and targeted efforts to improve vaccine accessibility, especially among SMM living with HIV. Addressing these gaps is essential to meet the high willingness for vaccination and to promote global vaccine equity.

### OA1002 Pre‐exposure prophylaxis use, discontinuation and HIV seroconversion in a cohort of men who have sex with men in a Buenos Aires‐based non‐governmental organization

R.B. Antivero Battistelli ^1^, Á. Lopez Malizia^1^, A. Adamczyk^1^, M.V. Iannantuono^2^, C. Cartasso^2^, M. Meijomil^1^, N. Morando^1^, A. Arévalo^3^, D. Serantes^3^, R. Marone^2^, M.Á. Pando^1^

^1^Instituto de Investigaciones Biomédicas en Retrovirus y SIDA (INBIRS UBA‐CONICET), CABA, Argentina, ^2^Nexo Asociación Civil, CABA, Argentina, ^3^Coordinación Salud Sexual, VIH e Infecciones de Transmisión Sexual, CABA, Argentina

**Background: **HIV pre‐exposure prophylaxis (PrEP) is recommended as an effective biomedical intervention to address the HIV pandemic. This study aims to examine the use of PrEP and HIV seroconversion during the first 30 months of PrEP administration among men who have sex with men (MSM) from a non‐governmental organization (NGO).

**Methods: **This retrospective cohort included MSM who initiated PrEP (TDF/FTC) from July 2021 to December 2023 in Buenos Aires, Argentina. Treatment discontinuation was defined as users going over 2 months without medication. Data were analysed using SPSS and R‐Studio.

**Results: **During the cohort period, 2442 MSM underwent rapid HIV testing, with 78 being reactive (3.2%). Among non‐reactive individuals (*n* = 2364), 1043 (44.1%) initiated PrEP. Younger MSM and those who tested positive for syphilis were more likely to initiate PrEP (*p*‐values<0.01). Of the 610 individuals on PrEP during the first 18 months, 83.6% (510) remained active after 6 months, and 69.3% (423) after 12 months. Adherence to PrEP for 6 months was higher among older individuals (median age: 34 vs. 32, *p*‐value: 0.027) and those who tested positive for syphilis at baseline (92.6% vs. 75.3%, *p*‐value: 0.006). Before the second month on PrEP, 38.3% (210/549) reported adverse effects associated with lower adherence at 6 months (*p*‐value: 0.008). Although all individuals had a negative rapid test before initiating PrEP, four users seroconverted during prophylaxis: one after 1 month and three after 2 months. Only one of these individuals underwent confirmatory HIV testing, which revealed the presence of the M184V mutation associated with resistance to FTC.

**Conclusions: **A positive syphilis diagnosis was found to be correlated with higher adherence, likely due to increased risk perception. This underscores the importance of screening for other STIs, thus raising awareness among PrEP users. The lower adherence in young adults is consistent with other studies, reinforcing the need to develop strategies tailored to this group. Additionally, seroconversion highlights the need to re‐evaluate screening methods before starting PrEP and to monitor resistance in HIV‐positive cases.

### OA1003 Unravelling PrEP persistence: mental health and substance use among South African adolescent girls and young women enrolled in a PrEP implementation programme

D. Giovenco ^1,2^, E. Rousseau^1^, M. Wallace^1^, N. Mathola^1^, B. Leonard^1^, L.‐G. Bekker^1^

^1^Desmond Tutu Health Foundation, Cape Town, South Africa, ^2^Emory University, Epidemiology, Atlanta, United States

**Background: **PrEP implementation among South African AGYW is essential to ending the HIV epidemic. However, mental health and substance use disorders may hinder PrEP engagement. We explored how these factors relate to the persistence of AGYW in a district‐wide PrEP programme.

**Methods: **We used data from FastPrEP, an ongoing youth‐focused, decentralized PrEP implementation programme with peer‐navigator support. From April 2022, PrEP was offered to young people at four community‐based mobile clinics and 12 health facilities near Cape Town. Females aged 15−29 years who received a 1‐month PrEP supply at enrolment were included. Log‐binomial regression was used to estimate associations between moderate‐severe depression/anxiety symptoms (PHQ‐4 ≥6) and disordered drinking (AUDIT‐C ≥3) at enrolment and persistence (1) in the programme (i.e. retention) and (2) on PrEP at the first follow‐up visit, adjusting for mobile versus facility enrolment. Findings were stratified by age.

**Results: **Through 2 April 2024, 7317 AGYW had PrEP dispensed through the FastPrEP programme. The median age was 21 years (IQR = 18−25) and most (78%) were enrolled at a mobile clinic. Further, 8% (316/4008) reported moderate‐severe depression/anxiety symptoms, which did not differ by age group (*X* ^2^ *p* = .069), and 49% (1984/4015) reported disordered alcohol use, which varied by age (15−19 years = 44%; 20−24 = 54%; 25−29 = 51%, *p*<.001). Among all AGYW, 31% (2264/7317) were retained and 24% (1741/7317) had PrEP dispensed at their first follow‐up visit. Moderate‐severe depression/anxiety symptoms and disordered alcohol use were associated with a greater probability of persistence. When findings were stratified by age, associations were only present for AGYW aged 15−19 years. Specifically, those with moderate‐severe depression/anxiety symptoms were more likely to be persistent in the programme (adjusted prevalence ratio [aPR] = 1.45, 1.15−1.82) and on PrEP (aPR = 1.45, 1.10−1.93), and those who reported disordered alcohol use were also more likely to be persistent in the programme (aPR = 1.22, 1.04−1.43) and on PrEP (aPR = 1.26, 1.04−1.53), at their first follow‐up visit.

**Conclusions: **Our results reveal an unexpected relationship between mental health, substance use and PrEP persistence among younger AGYW. Those facing these challenges may have found support within the PrEP programme, enhancing their likelihood of continued engagement. Further mixed‐methods investigations are warranted to elucidate these findings.

### OA1004 A mixed methods implementation study of peer‐led depression screening among transgender women with and without HIV in Thailand

K. Sitikornvorakul ^1^, R. Janamnuaysook^1^, K. Sinchai^1^, J. Ross^2^, N. Phanuphak^1^

^1^Institute of HIV Research and Innovation, Bangkok, Thailand, ^2^TREAT Asia, amfAR, Bangkok, Thailand

**Background: **Transgender women (TGW) experience high levels of depression, but access to mental health services remains limited. We assessed depressive symptoms and associated factors among TGW clients at the Tangerine Clinic, a transgender‐led sexual health clinic in Bangkok, Thailand, and feasibility, acceptability, facilitators and barriers to peer‐led depression screening.

**Methods: **Peer‐led depression screening was implemented between October 2021 and March 2022. TGW, aged >18 attending routine visits, answering “yes” to either or both Patient Health Questionnaire‐2 (PHQ‐2) questions were classified as positive, and further screened with PHQ‐9. Factors associated with positive PHQ‐2, and self‐harm and suicidal ideation were analysed using logistic regression. Self‐harm and suicidal ideation were defined as those endorsing the PHQ‐9 self‐harm and suicidal ideation item.

TGW declining PHQ‐2 screening (*n* = 2), PHQ‐9 score <7 (*n* = 5) and PHQ‐9 score >7 (*n* = 5) were purposively recruited for in‐depth interviews (IDIs) exploring feasibility, acceptability, facilitators and barriers to peer‐led depression screening. Healthcare providers (*n* = 12) were also interviewed. Two focus group discussions (FGDs) with TGW with different HIV status (*n* = 16) were conducted. Qualitative data were analysed using the Consolidated Framework for Implementation Research (CFIR) and Dedoose.

**Results: **A total of 317 TGW were screened, of which 167 (53%) had a positive PHQ‐2 screening. Of these, 12 (7%) were with HIV, and 86 (51%) were taking HIV pre‐exposure prophylaxis (PrEP). On PHQ‐9 screening, 41 (25%) had mild to moderate depressive symptoms (PHQ‐9 score 7−18), and 3 (2%) severe depressive symptoms (PHQ‐9 score≥19). In IDIs, rejection by family and friends, romance scams, financial hardship, school‐based bullying and multi‐level intersectional stigma were believed to be associated with depressive symptoms in TGW.

FGD and IDI participants agreed that implementing peer‐led depression screening at the Tangerine Clinic was feasible and acceptable. Key facilitators included a peer‐led service delivery model, stigma‐free clinic setting with associated psychiatric treatment costs identified as a key barrier.

**Conclusions: **The prevalence of depression symptoms among TGW was substantial. Implementing peer‐led depression screening using PHQ‐2/PHQ‐9 was feasible and acceptable to both TGW and healthcare providers. Multi‐level barriers to peer‐led depression screening were identified and should be addressed to facilitate improved access to mental healthcare services among TGW.

### OA1005 Reducing HIV acquisition among clients on oral pre‐exposure prophylaxis (PrEP) through the PrEP case‐manager model: Real‐world evidence from the DISCOVER‐Health Project PrEP programme in Zambia

A.P. Ndhlovu ^1^, L. Kawanga^1^, D. Phiri^1^, M. Musonda^2^, M. Nyumbu^3^

^1^JSI USAID DISCOVER‐Health Project, Technical Services, Lusaka, Zambia, ^2^USAID/Zambia Mission, Health, Lusaka, Zambia, ^3^JSI USAID DISCOVER‐Health Project, Lusaka, Zambia

**Background: **Oral tenofovir‐based HIV pre‐exposure prophylaxis (PrEP) is an effective modality that was approved for use by the WHO in 2015 to prevent HIV acquisition in high‐risk individuals. However, taking the daily oral pill is a significant challenge to adherence among clients on PrEP, explaining the 3% HIV incidence. To improve PrEP adherence and persistence, the DISCOVER‐Health Project developed a case‐manager model where clients on PrEP were assigned a trained community‐based volunteer (CBV).

**Methods: **CBVs working in Project‐supported health facilities were oriented in PrEP case‐management using a training curriculum adapted from national HIV treatment guidelines. At each health facility, trained CBVs were assigned a defined number of clients on PrEP for adherence support through appointment reminders via text messages, phone calls or home visits. A list of clients due for PrEP follow‐up visits was generated from the PrEP management system database every week for reminders within 3 days of the appointment visit. Individuals on PrEP were followed up for a year and de‐identified client‐level data were generated from the database for analysis using WINPEPI.

**Results: **Between 01/10/2022 and 30/09/2023, 55,940 clients were initiated on PrEP representing 55,940 person‐years. Of these, 52% were males, 47% were adolescents and young adults aged 15−24 years and 23% identified as key populations. In the same period, new HIV acquisitions were recorded in seven individuals on PrEP ranging 18−52 years old (mean 32.6 years; median 37 years) for the incidence rate of 0.013 (95% CI: 0.005−0.026) cases per 100 person‐years and mean survival time of 372 days (95% CI: 14.2−730). Two individuals were identified within 1 month of PrEP initiation. The risk of HIV acquisition was over 10 times higher in individuals in sero‐discordant relationships than in the general population (odds ratio 11.87; 95% CI: 2.66−53.04; *p*<0.0001)

**Conclusions: **Reinforcing adherence among individuals using PrEP through approaches like the case‐manager model has the potential to improve oral PrEP persistence and reduce HIV acquisition. Adoption of such models in real‐world settings may help to contribute towards the reduction of new HIV acquisitions towards HIV epidemic control.

### OA1006 HIV pre‐exposure prophylaxis (PrEP) efficacy, adherence and persistence in an Italian multicentric access programme (Sep2017−Nov2023): ItaPrEP study

V. Mazzotta ^1^, D. Calzavara^2^, A. Tavelli^3^, S. Lanini^4^, R. Esvan^5^, A. De Bona^6^, E. Caruso^2^, S. Mattioli^7^, F. De Zottis^5^, D. Tesoro^6^, L. Badia^7^, S. Nozza^8^, A. Cingolani^9^, A. Bianchi^2^, R. Bellagamba^5^, M. Pedone^7^, N. Frattini^2^, A. Oliva^5^, R. Repossi^2^, R. Rossotti^10^, C. Mastroianni^11^, C. Torti^9^, G. Marchetti^6^, A. Castagna^8^, A. d'Arminio Monforte^3^, M. Cernuschi^2^, A. Antinori^5^

^1^National Institute for Infectious Diseases Lazzaro Spallanzani IRCCS, Clinic, Rome, Italy, ^2^Milano Checkpoint, Milan, Italy, ^3^Fondazione Icona, Milan, Italy, ^4^Clinica Malattie Infettive, Università di Udine, Udine, Italy, ^5^National Institute for Infectious Diseases Lazzaro Spallanzani IRCCS, Rome, Italy, ^6^ASST Santi Paolo e Carlo, University of Milano, Milan, Italy, ^7^PLUS aps (BLQ Checkpoint), Bologna, Bologna, Italy, ^8^HSR San Raffaele Scientific Institute, Milano, Milan, Italy, ^9^Fondazione Policlinico A. Gemelli, Catholic University, Roma, Rome, Italy, ^10^9ASST Niguarda Hospital, Milan, Italy, ^11^Sapienza University, Roma, Rome, Italy

**Background: **PrEP implementation faced challenges until its reimbursement in 2023. National data are lacking. Aim is to report incidence rates (IRs) of HIV, poor adherence and discontinuation (outcomes), along with their predictors.

**Methods: **PrEP programme partially provided free drug supply. IRs were expressed as the number of new events/PYFU on PrEP. Poor adherence was defined as an incorrect intake for on‐demand, temporary stop for daily PrEP, or reported sex without PrEP or a condom, and PrEP discontinuation as a definitive stop of or loss to FU for at least 1 year. Kaplan‐Meier curves were fitted to estimate outcomes probability. A mixed‐effect logistic model with a random intercept on the centre was used to explore the association between risk factors and outcomes.

**Results: **One thousand seven hundred and fifty‐eight PrUs with at least one FU visit were included. Ninety‐eight percent men, 92% MSM, 88% Caucasian with a median age of 36 years (31−44). Sixty‐six percent had university degrees, and 19% used chemsex. Six hundred and fifty‐five PrUs (38%) chose daily, 619 (36%) on‐demand schedule and 464 (27%) switched. Nine hundred and sixty‐eight (55%) never had free supply. Six HIV seroconversions were observed over 2673 PYFU (IR 0.22/100 PYFU [95% CI: 0.08−0.49]). IR and 2‐year probability for outcomes were 44/100PYFU (40.8−47.2) and 57.9% (54.8−61.0) for poor adherence and 22.1/100PYFU (20.2−24.2) and 37.1% (34.3−40.1) for discontinuation. Chemsex (OR 1.56; 95% CI 1.11−2.18) and switching schedule (3.21; 2.38−4.33) were associated with poor adherence, unlike a high educational level (0.70; 0.54−0.91).[Fig jia226351-fig-0009]

**Figure 1 jia226351-fig-0009:**
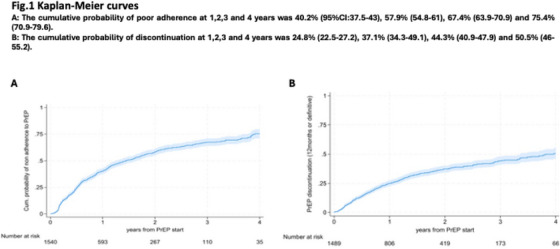
**OA1006**

An age >40y (0.68; 0.53−0.86), free drug supplies (0.73; 0.54−0.99) and laboratory monitoring (0.40; 0.29−0.53) were associated with a lower risk of discontinuation, while chemsex with a higher risk (1.80; 1.30−2.48) (Figure 1).

**Conclusions: **This Italian PrEP programme, prior to reimbursability, showed an HIV seroconversion rate lower than in RCT control arms in high‐risk populations. Younger age, low educational level and chemsex, and barriers such as lack of free drugs and monitoring are key to targeting strategies to improve PrEP implementation.

### OA1102 Antibodies to HIV‐1 CD4‐induced epitope may evolve from B lymphocytes primed by antigens from human microbiome or infectious agents

S. Biswas ^1^, T. Kuwata^1^, S. Yamauchi^2^, K. Okazaki^2^, Y. Kaku^1^, Z. Hasan^1^, H. Morioka^2^, S. Matsushita^1^

^1^Joint Research Center for Human Retrovirus Infection, Kumamoto University, Division of Clinical Retrovirology, Kumamoto, Japan, ^2^Faculty of Medical and Pharmaceutical Sciences, Kumamoto University, Department of Analytical and Biophysical Chemistry, Kumamoto, Japan

**Background: **Antibodies against the conformational CD4‐induced (CD4i) epitope are frequently found in HIV‐1 infected subjects. However, the mechanism of development of anti‐CD4i Abs is unclear. We used anti‐idiotypic (aID) monoclonal Abs (mAbs) of anti‐CD4i mAbs as bait to isolate anti‐CD4i mAbs from infected subjects and tracked the antigens that could have induced such Abs. Our preliminary results suggested that anti‐CD4i Abs may evolve from B cells primed by microorganisms.

**Methods: **Anti‐aID mAbs were isolated from HIV‐1‐infected subjects (following up regularly at Kumamoto University Hospital, Kumamoto, Japan) using aID mAbs against anti‐CD4i mAbs, 916B2 and 4E9C. Critical amino acid sequences for the binding of anti‐aID mAbs, with shared idiotope to anti‐CD4i mAbs, were analysed by phage display. Peptides synthesized based on the identified sequences were used to examine reactivity to anti‐aID mAbs and anti‐CD4i mAbs by AlphaLISA. Germline‐reverted anti‐aID mAbs and anti‐CD4i mAbs were constructed to estimate the ability of the peptides to prime these antibodies.

**Results: **Anti‐aID mAbs, including that with the characteristics of anti‐CD4i Abs, were isolated from HIV‐1 infected subjects. Three amino acid sequences were selected from the phage library by anti‐aID mAbs. The identified amino acid sequences showed similarity to proteins from members of human microbiota (*Caudoviricetes sp*) and infectious agents (*Vibrio vulnificus*). Peptides synthesized based on the identified sequences were reactive to most anti‐aIDs and some anti‐CD4i mAbs. These results suggest that anti‐CD4i Abs may evolve from B cells primed by microorganisms. To confirm this hypothesis, we tested binding activity of the germline reverted anti‐aID mAbs and anti‐CD4i mAbs to the peptides from *Caudoviricetes sp* and *Vibrio vulnificus*. Comparison with the original mAbs revealed that the binding activity to the peptides was significantly stronger in most of the germline reverted mAbs than original mAbs. Moreover, the germline reverted mAbs are being examined for their recognition amino acid sequences by phage display.

**Conclusions: **The hypothesis of antibody evolution from B cells primed by human microbiome may be attributed to antibody development and evolution against other viral antigens. Moreover, our data may provide important information for HIV‐1 vaccine design strategies to induce Abs against conformational epitopes.

### OA1103 The vaginal microbiome pre‐vaccination associates with local and systemic HIV vaccine immune responses

L. Noel‐Romas^1^, A. Borgognone^2^, F. Catala‐Moll^2^, R. Eckert^1^, S. Bailey^1^, M. Parera^2^, A. Yoon^1^, S. Azzam^1^, S. Knodel^1^, O. Taylor^1^, S. Akapirat^3^, S. Rittiroongrad^3^, J. Ake^4^, S. Vasan^4^, C. Herrera^5^, R. Paredes^2^, A. Schuetz^3^, A. Burgener ^1^, RV306 Study Group

^1^Case Western Reserve University, Cleveland, United States, ^2^IrsiCaixa AIDS Research Institute, Badalona, Spain, ^3^WRAIR‐Armed Forces Research Institute of Medical Sciences, Bangkok, Thailand, ^4^WRAIR‐US Military HIV Research Program, Silver Spring, United States, ^5^CONRAD‐Eastern Virginia Medical School, Norfolk, United States

**Background: **New vaccine strategies against HIV should induce protective mucosal immune response in the female genital tract which is a first site of HIV acquisition. The microbiome has been implicated in altered mucosal immunity, but its impact on vaccine‐induced mucosal responses in the genital tract is not well understood. Here, we assess the relationship between the vaginal microbiome and vaccine‐induced binding antibody (BAb) responses.

**Methods: **Cervicovaginal mucus samples were collected from participants (*n* = 95) receiving the RV144 ALVAC‐HIV/AIDSVAX B/E prime/boost vaccine regimen at baseline and 2 weeks post‐RV144 prime/boost regimen. Vaginal microbiome data were generated by label‐free tandem mass spectrometry. Mucosal and serum BAb levels were measured using ELISA, and differences between microbiome groups were determined by *t*‐tests.

**Results: **A total of 24 unique genus‐ and species‐level taxa were identified. Participants clustered into three microbiome groups: *Lactobacillus* (*L*.) *crispatus* dominant (*n* = 20, 21.1%), *L. iners* (*n* = 40, 42.1%) and a polymicrobial group (PM, *n* = 36.8,36.8%). Relative to *L. crispatus* group, women with a PM microbiome showed increased activation of pathways involved in B‐ and T‐cell receptor signalling, MHC class I/II antigen presentation and neutrophil degranulation (Z score>5; *p*<0.0001). A subset of women had a consistent *Lactobacillus* dominant (LD, *n* = 43) or non‐*Lactobacillus* dominant (nLD, *n* = 18) microbiome type across all timepoints. These sub‐groups were similar in age (avg_LD_ = 29.6, avg_nLD_ = 28.5, *p* = 0.4). LD women showed significantly higher BAb levels compared to nLD women at 2 weeks post‐RV144 regimen, including those in the mucosa against A244 (IgG‐gp120A244gD‐D11, L2FD = 0.82, *p* = 0.0393) and in serum (gp70V1V2‐92TH023, L2FD = 0.78, *p* = 0.004; IgG‐A244, L2FD = 0.66, *p* = 0.0153).

**Conclusions: **These data suggest an association between the vaginal microbiome and vaccine‐induced BAb responses, with a more inflammatory polymicrobial environment being associated with lower vaccine‐specific BAb levels. These results warrant further interrogations into the potential mechanisms by which the vaginal microbiome may impact vaccine responses and could inform interventional strategies.

### OA1104 Similar meta clonotypes recognize HIV‐1 Gag‐KF11 across HLA‐E*01:01/03 and HLA‐B*57:01 with distinct functional profiles

K. Maroney ^1,2^, A. Bansal^2^, P. Goepfert^2^, A. Oman^2^, M. Rose^2^, M. Brockman^3^, S. Kalams^4^

^1^University of Alabama at Birmingham, Infectious Diseases, Birmingham, United States, ^2^University of Alabama at Birmingham, Infectious Diseases, Birmingham, United States, ^3^Simon Fraser University, Health Sciences, Burnaby, Canada, ^4^Vanderbilt University, Microbiology and Immunology, Nashville, United States

**Background: **A recombinant cytomegalovirus (CMV) vaccine that induces MHC‐E restricted CD8 T cells has demonstrated 50% efficacy in preventing the establishment of SIV transmission. However, little is known about HLA‐E restricted HIV‐specific responses in people with HIV (PWH). Our previous work showed that CD8 T cells targeting a Gag epitope (KAFSPEVIPMF or KF11) were restricted by HLA‐B*57:01 (B57‐CD8s) and HLA‐E*01:01 (E‐CD8s) in PWH. This study aims to extensively compare the characteristics of KF11‐specific CD8 T cells depending on their HLA restriction.

**Methods: **We obtained CD8 T cells from PWH, including two non‐controllers (NC), four controllers (C) and two elite controllers (EC), and stimulated them with the KF11 epitope in the presence of HLA‐B*57:01, HLA‐E*01 or 03 antigen‐presenting cells. We analysed the resulting supernatants with a Luminex assay. We sorted antigen‐specific CD8 T cells and assessed them using single‐cell RNA and TCR sequencing. Select TCR clones underwent further analysis in a NFAT‐driven luciferase‐based TCR reporter assay.

**Results: **B57‐CD8s secreted higher levels of conventional HLA‐Ia cytotoxic cytokines like IFNγ, whereas E‐CD8s produced more chemotactic cytokines, including RANTES, IP‐10 and IL27. TCR α/β paired family analysis showed that despite these functional differences, most TCRs were dual‐restricted by B*57 and E*01/03. Furthermore, we discovered an enriched cluster of TRAV5*01‐TRBV19*01 clones, representing a tri‐restricted metaclonotype in controllers. This tri‐restriction was substantiated when TRAV5‐containing clones triggered NFAT signalling in the presence of KF11‐loaded B57‐, E01‐ or E03‐cell lines. Deep sequencing of sorted KF11‐specific CD8 T cells identified a significant increase in the TRAV5 cluster solely in controllers. We additionally identified another tri‐restricted cluster of TRAV27*01‐ and TRAV29/DV5*01‐TRBV2*01 clones isolated to the elite controller. Moreover, within this antigen‐specific CD8 subset, a dually E03‐/B57‐restricted cluster within this elite controller metaclonotype showed activation and stimulation, characterized by an IL15 signalling pathway signature like that in macaques which resisted SIV transmission post‐vaccination with an MHC‐E restricted CMV‐based vaccine, exhibiting upregulation of both IL15 and IL15RA.

**Conclusions: **Our findings suggest that CD8 T cells, while dually restricted by HLA‐B*57 or HLA‐E, are functionally distinct depending on the presenting HLA. These dual‐restricted CD8 T cells were predominantly observed in PWH who naturally control transmission.

### OA1105 HLA‐B*07:02 associated with higher vaccine efficacy in the phase 2b Imbokodo trial among young cisgender women with increased likelihood of HIV acquisition in Southern Africa

S.S. Li ^1^, C.‐W. Pyo^2^, A. Fiore‐Gartland^1^, R. Burnham^1^, J. van Duijn^3^, O. Dintwe^1,4^, C. Margaret^1^, J. Hural^1^, M. Juraska^1^, L. Li^1^, W. Willems^5^, H. Schuitemaker^3^, E. Swan^6^, J.G. Kublin^2^, L. Corey^1,7^, M.G. Pau^3^, S. Buchbinder^8^, F. Tomaka^9^, S. Nijs^5^, L. Lavreys^5^, H. Gelderblom^1^, K. Mngadi^10^, G.E. Gray^11^, E. Borducchi^12^, J. Hendriks^3^, D. Barouch^12^, S. De Rosa^1^, M.J. McElrath^1,7^, E. Andersen‐Nissen^1,4^, D.J. Stieh^3^, G.D. Tomaras^13,14^, C. Williamson^15^, J.I. Mullins^16^, P.B. Gilbert^1,17^, D.E. Geraghty^2^

^1^Fred Hutchinson Cancer Center, Vaccine and Infectious Disease Division, Seattle, United States, ^2^Fred Hutchinson Cancer Center, Clinical Research Division, Seattle, United States, ^3^Janssen Vaccines & Prevention BV, Leiden, the Netherlands, ^4^Cape Town HVTN Immunology Laboratory, Cape Town, South Africa, ^5^Janssen Infectious Diseases BV, Beerse, Belgium, ^6^National Institute of Allergy and Infectious Diseases, Division of AIDS, Bethesda, United States, ^7^University of Washington, Department of Medicine, Seattle, United States, ^8^San Francisco Department of Public Health, San Francisco, United States, ^9^Janssen Research & Development, LLC., Titusville, United States, ^10^The Aurum Institute, Johannesburg, South Africa, ^11^South African Medical Research Council, Cape Town, South Africa, ^12^Beth Israel Deaconess Medical Center, Center for Virology & Vaccine Research, Boston, United States, ^13^Duke University, Department of Surgery, Durham, United States, ^14^Duke Human Vaccine Institute, Durham, United States, ^15^University of Cape Town, Division of Medical Virology, Cape Town, South Africa, ^16^University of Washington, Department of Global Health; Department of Microbiology, Seattle, United States, ^17^University of Washington, Department of Biostatistics, Seattle, United States

**Background: **The phase 2b Imbokodo study (HVTN 705/HPX2008; NCT03060629) evaluated an investigational HIV‐1 vaccine regimen, consisting of a vector‐based vaccine (Ad26.Mos4.HIV) combined with clade C gp140 protein, among young cisgender women behaviourally vulnerable to HIV in Southern Africa. While the trial demonstrated an estimated non‐significant vaccine efficacy (VE) of 14% versus placebo, analysis of HIV sequences isolated from study participants (i.e. sieve analysis) revealed higher VE against HIV‐1 with non‐proline (P) amino‐acid residues compared to a P residue at position 364 in Env (30.1% 95% CI [−4.5%, 53.2%] vs. −235.6% [−929.3%, −9.4%], *p* = 0.010). We investigated whether host HLA genotypes, known to influence vaccine responses and HIV VE, modulated VE.[Table jia226351-tbl-0014]

**Table jia226351-tbl-0014:** OA1105

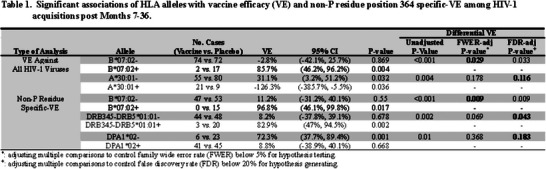

**Methods: **Between months 7 and 36 post‐enrolment, 165 per‐protocol participants acquired HIV‐1. Of these, 115 participants acquired HIV‐1 with a non‐P residue at position 364. HLA class I and II typing was performed on the samples from all per‐protocol participants who acquired HIV‐1. HLA alleles with >5% frequency were selected for assessment of their association with overall and non‐P residue VE. Associations were assessed using the case‐only method. Adjusted *p*‐values, controlling the family‐wise error rate (FWER‐adjusted *p*‐value) and false discovery rate (FDR‐adjusted *p*‐value), were calculated using a resampling method. Associations with FWER‐adjusted *p*‐values <0.05 were considered significant (robust evidence of an association). Associations with FDR‐adjusted *p*‐values <0.20 were considered hypothesis‐generating.

**Results: **HLA‐B*07:02 was significantly associated with an increased overall VE (86% vs. −3%, *p*<0.001) and non‐P residue VE (97% vs. 11%, *p*<0.001) (Table 1). HLA‐DRB5*01:01 exhibited an increased non‐P residue VE. HLA‐A*30:01 exhibited a decreased VE. HLA‐DPA1*02 exhibited a decreased non‐P residue VE.

**Conclusions: **We demonstrate a VE of 86% among HLA‐B*07:02‐positive participants in the Imbokodo trial. HLA‐B*07:02 was reported in association with a broad and potent response targeting Gag epitopes in the literature. Further epitope mapping will elucidate potential mechanisms of vaccine protection.

### OA1106 Characterization of the B‐cell repertoire in African populations to inform HIV vaccine design

M. Muthui ^1^, C. Muriuki^2^, C. Kibet^3^, S. Owuor^1^, Y. Sein^1^, D. Muema^2,4^, E. Nduati^1,5^

^1^KEMRI‐Wellcome Trust Research Programme, Biosciences, Kilifi, Kenya, ^2^Kenya Aids Vaccine Initiative, Nairobi, Kenya, ^3^International Aids Vaccine Initiative, Nairobi, Kenya, ^4^Africa Health Research Institute, Durban, South Africa, ^5^University of Oxford, Centre for Tropical Medicine and Global Health, Nuffield Department of Clinical Medicine, Oxford, United Kingdom

**Background: **Developing an effective HIV vaccine remains key to HIV eradication. One new approach to vaccine design utilizes germline‐targeting immunogens to target and expand broadly neutralizing antibody (bnAb) precursors, hence the success of this approach relies on the composition of the naïve B‐cell repertoire. Unfortunately, the human naïve B‐cell repertoire remains insufficiently characterized, particularly in African populations. More studies are, therefore, required to describe the allelic diversity in African populations, and to assess precursor frequencies for the various bnAb classes for which germline targeting immunogens are being developed. One example is the eOD‐GT8 immunogen that primes the CD4 binding‐site‐specific VRC01‐class antibody precursors. We set up a study to characterize the African antibody repertoire and assess factors that may alter the repertoire, for instance, chronic pathogen exposure.

**Methods: **We recruited participants from three regions in Kenya experiencing varied malaria transmission intensities and collected PBMCs from each participant. Using single‐cell RNA‐Seq, we characterized B‐cell receptor sequences from isolated eOD‐GT8‐binding cells. Additionally, using bulk RNA‐Seq, we characterized the naïve immunoglobulin repertoire. Analysis of the single‐cell sequence data identified sequences with VRC01‐class signatures in order to describe the precursor frequencies in this population. Analysis of the overall antibody repertoire will define gene usage, identify novel alleles and assess the potential impact of malaria exposure on the antibody repertoire.

**Results: **Preliminary analysis indicates an average VRC01‐class precursor frequency of one in 379,377 naïve B cells, in line with previous studies. We have also identified signatures linked to 11 other CD4‐binding site bnAb sub‐classes including the highly potent N6 sub‐class. Furthermore, previous malaria exposure does not seem to influence the precursor frequency. Preliminary analysis of naïve repertoire shows (a) dominance of the IGHV1‐2*02 allele, among VH1‐2 alleles, associated with higher usage of the VH1‐2 gene that is a hallmark of VRC01‐class precursors and (b) the presence of novel alleles in each participant's antibody repertoire.

**Conclusions: **Despite genetic diversity in the naïve B‐cell repertoire and varied levels of malaria exposure, VRC01‐class precursors are present at similar frequencies to previous studies. This supports evaluating the germline targeting vaccine strategy for VRC01‐class bnAb induction within this population.

### OA1202 Mutation‐guided HIV vaccine design: A strategy for developing boosting immunogens for BnAb induction

K. Wiehe ^1^, K. Saunders^1,2^, V. Stalls^1^, D. Cain^1^, S. Venkatayogi^1^, J. Martin Beem^1^, M. Berry^1^, T. Evangelous^1^, R. Henderson^1^, B. Hora^1^, S.‐M. Xia^1^, C. Jiang^1^, A. Newman^1^, C. Bowman^1^, X. Lu^1^, J. Bal^1^, A. Sanzone^1^, H. Chen^1^, A. Eaton^1^, M. Tomai^3^, C. Fox^4^, Y. Tam^5^, C. Barbosa^5^, M. Bonsignori^6^, H. Muramatsu^7^, M. Alam^1^, D. Montefiori^1^, W. Williams^1^, N. Pardi^7^, M. Tian^8^, D. Weissman^7^, F. Alt^8^, P. Acharya^1^, B. Haynes^1^

^1^Duke University, Duke Human Vaccine Institute, Durham, United States, ^2^Duke University, Durham, United States, ^3^3M, St. Paul, United States, ^4^AAHI, Seattle, United States, ^5^Acuitus, Inc, Vancouver, Canada, ^6^NIH, Bethesda, United States, ^7^University of Pennsylvania, Philadelphia, United States, ^8^Harvard Medical School, Boston, United States

**Background: **A major goal of HIV‐1 vaccine development is the induction of broadly neutralizing antibodies (bnAbs). While success has been achieved in initiating bnAb B‐cell lineages via vaccination, design of boosting immunogens that select for bnAb B‐cell receptors with improbable mutations required for bnAb affinity maturation remains a central challenge.

**Methods: **DH270 unmutated common ancestor (UCA) knock‐in mice were immunized with the CH848 10.17DT priming immunogen formulated as a ferritin nanoparticle and boosted with the 10.17WT Env trimer immunogen. Improbable mutations were mapped with the ARMADiLLO computational programme. Vaccine‐induced monoclonal antibodies were isolated from immunized mice using antigen‐specific single B‐cell sorting strategies. B‐cell repertoire single heavy or light chain next‐generation sequencing of immunized mice was performed to analyse the change in frequency of key mutations over time and in response to immunization regimens. Structural determination of vaccine‐induced bnAbs was performed by cryo‐EM.

**Results: **We demonstrate a general process for designing HIV boosting immunogens and apply it to the design of a vaccine regimen targeting the V3‐glycan bnAb DH270 lineage. First, we identified a set of key mutations in the DH270 lineage that are sufficient for neutralization breadth. We then developed a boosting immunogen with a favourable binding profile for selecting these key mutations and tracked the selection of these mutations during affinity maturation in immunized bnAb precursor knock‐in mice using antibody repertoire sequencing. We show that our prime‐boost regimen elicited bnAbs by selecting for the targeted functional improbable mutations. Structural determination of a vaccine‐induced bnAb revealed that selected key improbable mutations make critical interactions with conserved elements within the V3‐glycan epitope. Moreover, we show similar success in prime and boosting with nucleoside‐modified mRNA‐encoded HIV‐1 envelope trimer immunogens with improved selection by mRNA immunogens of improbable mutations required for bnAb binding to critical envelope glycans.

**Conclusions: **These results demonstrate the ability of both protein and mRNA prime‐boost immunogens to select critical improbable mutations in bnAb B‐cell lineages and induce antibodies with neutralization breadth after bnAb precursor expansion, a key proof‐of concept and milestone towards the development of an HIV vaccine.

### OA1203 RV 575 study: a phase 1 double blinded dose optimization study of ALFQ adjuvant with an HIV envelope vaccine containing A244 and B.63521… *Results of a blinded interim analysis*

P. Adjei ^1^, E. Serti^1,2^, D. Leggat^1^, T. Hamby^1,2^, J. Sleesman^1,2^, P. Nguyen^1,2^, B. Slike^1,2^, M. Zemil^1,2^, C. Lee^3^, Y. Petrova^1,2^, T. Wellington^3^, P.M. Robben^4^, A. Moodley^1,2^, J. Dorsey‐Spitz^1,2^, Q. Li^1,2^, F. Hu^1,2^, Z. Hansen^1^, L. Zhu^3^, C. Lange^1,2^, J. Kim^1,2^, S. Krebs^1^, T. Crowell^1,2^, V. Polonis^1^, M. Rao^1^, G. Matyas^1^, S. Vasan^1,2^, M. Thigpen^1^, J.A. Ake^1^, RV575 Study Group

^1^Walter Reed Army Institute of Research, Military HIV Research Program, Forest Glen, United States, ^2^Henry M. Jackson Foundation for the Advancement of Military Medicine, Inc., Bethesda, United States, ^3^Walter Reed Army Institute of Research, Clinical Trials Center, Forest Glen, United States, ^4^Walter Reed Army Institute of Research, Biologics Research & Development, Center for Infectious Diseases Research, Forest Glen, United States

**Background: **The Army Liposomal Formulation containing monophosphoryl lipid A (MPLA) and QS21 saponin (ALFQ), developed and patented by the U.S Army, is a safe and immunogenic adjuvant for vaccines. Prior studies used ALFQ with 100/50 and 200/100 µg MPLA/QS21. We conducted a phase 1 study to further optimize ALFQ dosing.

**Methods: **We randomized sixty‐five 18‐ to 55‐year‐olds without HIV to receive one of three ALFQ doses (200/100, 100/50 and 50/25 µg MPLA)/QS21 and 300 µg each of HIV‐envelope gp120 protein vaccines A244 and B.63521 at weeks 0, 4 and 8. We monitored post‐vaccination reactogenicity, adverse events and safety laboratory tests. We measured vaccine‐specific antibody responses against a custom multiplex array of 30 HIV antigens by ELISA and Luminex, and peripheral antigen‐specific CD4^+^ T‐cell responses at weeks 2, 4, 6 and 10. Laboratory investigators were blinded to treatment. Unblinded statisticians conducted analyses.

**Results: **Mean (SD) age was 34 (8.5) years, 55.4% Caucasian, 30.8% Black, 50.8% female sex, 4.6% nonbinary gender, 1.5% transgender. Fifty‐seven (87.7%) participants each reported at least one local and systemic reactogenicity event, respectively, main events: injection site pain 87.7%, fatigue 78.5%, headache 73.8%, myalgia 69.2%; 39 (60%) reported at least one adverse event, 87.2% study product‐unrelated. Binding and neutralizing antibody responses were detected following the first vaccination and increased after each subsequent vaccination. Although no significant differences in the magnitude of binding antibody responses to cognate vaccine immunogens or neutralization potency to a Tier 1 panel were observed between 100 and 200 µg MPLA ALFQ groups, binding antibodies were significantly higher compared to the 50 µg ALFQ group. Post‐third vaccination, all arms showed equivalent levels of binding and neutralizing antibodies across all isotypes. Similar results were observed with ELISAs examining antigen‐specific antibodies against the vaccine immunogens with titres increasing at weeks 2 and 6 in all arms. Antigen‐specific CD4^+^ T‐cell responses, at 2 weeks post‐second boost, showed significant increases (*p*<0.01 for TNF‐alpha and *p*<0.05 for IL2, IFN‐gamma and CD40L) between low to high doses of ALFQ.

**Conclusions: **Preliminary blinded data show that ALFQ‐adjuvanted bivalent HIV‐1 vaccine is safe and well‐tolerated. Binding antibody titres and CD4^+^ T‐cell responses were ALFQ dose‐dependent and increased with the first two vaccinations.

### OA1204 Vaccination with a novel fractional escalating dose strategy improves early humoral responses with a novel germline targeting HIV vaccine (426.mod.core‐C4b): Preliminary results from HVTN 301

W. Hahn ^1^, K.R. Parks^2^, S. De Rosa^2^, D. Montefiori^3^, L. Baden^4^, S.R. Walsh^4^, G. Tomaras^3^, P. Goepfert^5^, C. Kelly^6^, M. Sobieszcyzk^7^, M.J. McElrath^2^, M. Yacovone^8^, H. Scott^9^, O. Hyrien^2^, L. Stamatatos^2^

^1^Fred Hutchinson Cancer Institute/University of Washington, Seattle, Medicine (Division of Allergy and Infectious Disease), Seattle, United States, ^2^Fred Hutchinson Cancer Institute, Seattle, United States, ^3^Duke University, Department of Surgery, Durham, United States, ^4^Harvard Medical School, Medicine, Boston, United States, ^5^University of Alabama Birmingham, Birmingham, United States, ^6^Emory University, Medicine, Atlanta, United States, ^7^Columbia University, Medicine, New York, United States, ^8^National Institute of Allergy and Infectious Disease, Division of AIDS, Washington, United States, ^9^University of California, San Francisco, Medicine, San Francisco, United States

**Background: **Conventionally, vaccines are delivered through a single “bolus” administration. In preclinical models, HIV vaccination strategies that mimic the sustained antigen load which the immune system experiences during acute infection improve the quality of humoral responses compared to traditional bolus administration. Here, we report the results of a clinical trial testing the effectiveness and tolerability of a strategy based upon these concepts called fractionated escalating dosing.

**Methods: **HVTN301 is a first‐in‐human, double‐blind, placebo‐controlled trial evaluating an HIV immunogen nanoparticle (426.mod.core‐C4b) adjuvanted with the TLR7 agonist 3M‐052 AF/Alum intended to expand CD4‐binding site bnAb lineage B cells. The trial compares 426.mod.Core administered as a single 100 mcg “bolus” dose compared with a “fractional escalating dose” delivering 100 mcg split into increasing doses given over 3 weeks in the priming phase (426.mod.Core dose of 2, 5, 10, 15, 20 and 48 mcg). Outcomes include adverse events (AEs), antigen‐specific CD4^+^ T cells and memory B cells (by flow cytometry), binding antibody (by binding multiplex assay) and serum neutralization (using a pseudovirus panel), all compared by Wilcoxon rank sum test.

**Results: **We enrolled 53 adults without HIV at six sites. The vaccine was safe, with no related serious AEs, related severe unsolicited AEs or unplanned study pauses. Overall tolerability was similar with either fractional or bolus administration. Two weeks after the initial prime, fractional escalating doses led to increased frequency of CD4‐binding‐site specific memory B cells (median 0.050% vs. 0.114%, *p*<0.001), antigen‐specific CD4^+^ T cells (median 0.005% vs. 0.118%, *p*<.0001), increased total quantity of serum IgG that binds the vaccine (MFI 755 vs. 3674 at 1:31250 dilution, *p*<.0001) and higher titres of neutralizing antibodies to HIV pseudovirus strains that identify CD4‐binding‐site antibodies (55 vs. 284, *p*<.001).

**Conclusions: **Fractional escalating dose strategies of a nanoparticle HIV vaccine with a novel adjuvant was safe, tolerable and enhanced antibody, B cell, and CD4^+^ T‐cell responses during the priming phase. HVTN 301 establishes proof‐of‐concept that sustained antigen/adjuvant exposure can improve immune responses for preventive HIV vaccines intended to elicit broadly neutralizing antibodies during the priming phase, supporting the identification of strategies based upon this concept that can be more readily translated into clinical practice.

### OA1205 Membrane‐anchored HIV‐1 Env trimer BG505 MD39.3 mRNA is immunogenic and can elicit tier 2 autologous neutralizing antibodies (HVTN 302)

K. Parks ^1^, Z. Moodie^1^, B. Furch^1^, W. Hahn^1^, J. Heptinstall^2^, K. MacPhee^1^, G. Ozorowski^3^, A. Seese^1^, L. Ballweber‐Fleming^1^, C. Marini‐Macouzet^1^, C. Cottrell^1^, S. Grant^4^, Z. Zheng^4^, H. Lu^4^, L.A. Cheves^1^, M. Abay^1^, M. McBride^1^, A. Woodward‐Davis^4^, A. Ward^3^, S. De Rosa^1^, D. Laufer^5^, J. Kublin^1^, G. Tomaras^2^, O. Hyrien^1^, D. Montefiori^2^, W. Schief^6^, S. Riddler^7^, J. Clark^8^, J. McElrath^1^

^1^Fred Hutch, VIDD, Seattle, United States, ^2^Duke, Durham, United States, ^3^Scripps, San Diego, United States, ^4^Fred Hutch, Seattle, United States, ^5^IAVI, New York, United States, ^6^Moderna, Boston, United States, ^7^Univerity of Pittsburgh, Pittsburgh, United States, ^8^UCLA, Los Angeles, United States

**Background: **Harnessing mRNA vaccine technology has the potential to accelerate HIV‐1 vaccine development, but no published data are available on human immune responses to HIV‐1 envelope (Env) delivered by mRNA. Here, we tested the immune response induced by the BG505 MD39.3 trimer expressed as mRNA in three forms: soluble (gp140), membrane‐bound (gp151) and membrane‐bound with a CD4‐binding site knockout (KO) mutation. Both occluding the trimer base with membrane display and disrupting CD4‐binding site‐induced conformational changes via a KO mutation aim to reduce the elicitation of non‐neutralizing antibodies.

**Methods: **HVTN 302 (NCT05217641) is a phase 1, open‐label, randomized, multicentre trial evaluating the safety and immunogenicity of three mRNA‐encoded HIV envelope trimers at two doses: 100 and 250 mcg. Participants were vaccinated intramuscularly at months 0, 2 and 6. Immune assays quantified the response rate and magnitude of vaccine‐specific binding antibodies, B cells, T cells and serum neutralizing antibodies 2 weeks after the last vaccination.

**Results: **HVTN 302 enrolled 108 participants from February to August 2022. Robust vaccine‐specific antibody binding titres, vaccine‐specific IgG+ B‐cell responses, and vaccine‐specific CD4 and CD8 T‐cell responses were observed in all groups without major differences between soluble and membrane‐bound groups. However, IgG+ B‐cell responses among positive responders to non‐base epitopes were significantly increased in the membrane‐bound group after three immunizations (250 mcg of gp151 vs. gp140, median frequency: 2.0% vs. 0.8%, *p* = 0.013). Autologous tier 2 ID50 neutralizing antibody titres were more frequent in the membrane‐bound groups (100 mcg gp151 vs. gp140, 11/17 vs. 0/16, Fisher's exact test *p*<0.0001; 250 mcg gp151 vs. gp140, 12/14 vs. 1/12, *p*<0.0001). Epitope mapping of elicited antibodies determined the major target of the neutralizing response to be non‐base epitopes (V1 and C3V5).

**Conclusions: **This study indicates that HIV‐1 Env trimers delivered as mRNA are highly immunogenic and more capable of eliciting tier 2 autologous neutralizing antibodies when anchored in a membrane. Therefore, we conclude that strategies to reduce exposure to the immunodominant base of the HIV‐1 Env trimer may help to improve the neutralizing antibody responses in vaccines aiming to elicit broadly neutralizing antibodies.

### OA1206 Immunogenicity of fusion peptide and trimer vaccination: preliminary results from HVTN 303 part B, a phase 1 randomized clinical trial in adults without HIV

L. Serebryannyy^1^, F. Matassoli^1^, M. Castro ^1^, S. Narpala^1^, A. Olia^1^, S. O'Dell^1^, K. McKee^1^, A. Henry^1^, A. Shah^1^, J. Bahorich^1^, K. Carlton^1^, J. Gall^1^, N. Doria‐Rose^1^, D. Douek^1^, L. Gama^1^, L. Dropulic^1^, M. Allen^2^, M. Keefer^3^, D. Montefiori^4^, J. Hural^5^, T. Martin^5^, C. Paez^5^, S. Andrews^1^, P. Kwong^1,6^, T. Pierson^1^, R. Koup^1^

^1^Vaccine Research Center, Bethesda, United States, ^2^National Institute of Allergy and Infectious Diseases, Bethesda, United States, ^3^University of Rochester, Rochester, United States, ^4^Duke University, Durham, United States, ^5^Fred Hutchinson Cancer Center, Seattle, United States, ^6^Columbia University, New York, United States

**Background: **HVTN 303, a trial of HIV‐1 fusion peptide‐directed vaccine, was designed to prime with HIV envelope Trimer 4571, a fusion peptide conjugate (FP8‐rTTHC), or their combination, with subsequent boosting with Trimers 4571 and 6931, all adjuvanted with Adjuplex (20%). In March 2023, vaccinations were permanently discontinued due to grade 3 solicited local and systemic reactogenicity (fever, injection site erythema and induration) and unsolicited adverse events (urticaria, rash and serum sickness). Subsequently, boosting with Trimers 4571 and 6931 was stopped.

**Methods: **We evaluated peak immune responses of participants who had received one vaccination with Trimer 4571 (groups 5+6, *n* = 13), two vaccinations with FP8‐rTTHC (group 7, *n* = 5) or two vaccinations with a combination of both immunogens (group 8, *n* = 6). Sera were tested for binding antibodies to FP8 alone or with Trimer 4571 and two assays assessed percent related to trimer base binding. Neutralization was determined by standard pseudovirus neutralization assays. Immunogen‐specific B cells were quantified by flow cytometry, sorted, and their antibodies were produced by the RATP‐Ig protocol and tested for binding and neutralization.

**Results: **Serum antibodies to FP8 were elicited in all three study groups. The highest titres were elicited after two vaccinations with either FP8‐rTTHC alone or in combination with Trimer 4571 (fold increase from baseline of 1.6 in groups 5+6, 27.9 in group 7 and 23.5 in group 8). Binding antibodies to trimer were detected after two immunizations with Trimer 4571; 65−75% of which was directed at the base. Serum neutralization was not detected in any group. Memory B cells expressing antibodies that bind FP8 in the context of native trimer were detected in 3/4 participants in group 7 and 4/4 in group 8. Some of the antibodies from these B cells can neutralize HIV.

**Conclusions: **Two vaccinations of FP8‐rTTHC regimens induced FP8‐binding antibodies in serum and B cells capable of recognizing FP8 in the context of native HIV envelope trimer. Evaluation of B‐cell responses and safety is ongoing. These findings may inform the development of antigens to generate broad FP‐directed responses that can protect against HIV infection.

### OA1302 Pre‐exposure prophylaxis (PrEP) modality and service delivery preferences among diverse populations in Malawi: Results from a discrete choice experiment

S. Schwartz ^1^, A. Bula^2^, L. Hill^3^, R. Chilongosi^4^, D. Hoege^5^, R. Nyirenda^6^, S. Sikwese^7^, S. Phiri^8^, F. Saidi^2^, W. Ozituosauka^6^, R. West^9^, S. Allinder^5^, M. Hosseinipour^3^, C. Holmes^5^

^1^Johns Hopkins School of Public Health, Epidemiology, Baltimore, United States, ^2^UNC Project, Lilongwe, Malawi, ^3^University of North Carolina, Chapel Hill, United States, ^4^Family Health Services, Lilongwe, Malawi, ^5^Georgetown University, Center for Innovation in Global Health, Washington, DC, United States, ^6^Malawi Ministry of Health, Department of HIV/AID, Lilongwe, Malawi, ^7^Pakachere, Blantyre, Malawi, ^8^Partners in Hope, Lilongwe, Malawi, ^9^IPSOS, London, United Kingdom

**Background: **As long‐acting injectable cabotegravir PrEP is rolled‐out globally, it is critical to understand preferences within and across populations for delivery. We compared PrEP modality and service delivery preferences through a discrete choice experiment (DCE) among diverse populations in Malawi.[Fig jia226351-fig-0010]

**Figure 1 jia226351-fig-0010:**
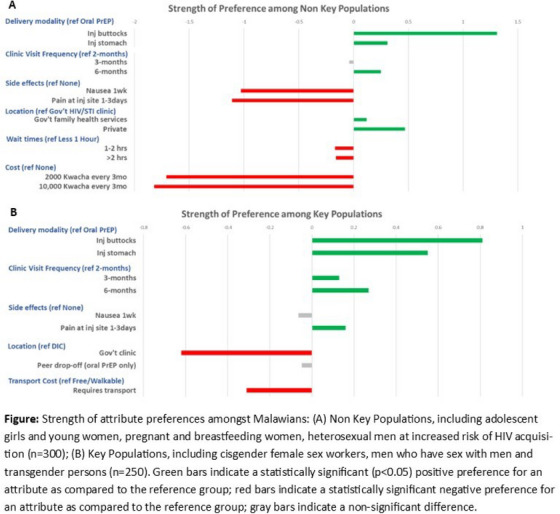
**OA1302**

**Methods: **Participants ≥18 years and potentially at‐risk for HIV acquisition were recruited at public clinics and key populations (KP) drop‐in centres within Lilongwe and Blantyre, Malawi for a cross‐sectional survey. KP groups included female sex workers (FSW, *n* = 100), men who have sex with men (MSM, *n* = 100) and transgender individuals (TG, *n* = 50); non‐KP groups sampled included adolescent girls and young women (AGYW, *n* = 100), pregnant and breastfeeding women (PBFW, *n* = 100) and heterosexual men with increased HIV acquisition risk (*n* = 100). Two DCEs were designed (KP and non‐KP). We assessed levels across six attributes to identify factors driving stated PrEP preferences: PrEP modality (oral vs. injection), visit frequency, side effects, PrEP service delivery location, costs and waiting times* (*non‐KP only). Effects were coded using a multinomial logit model.

**Results: **From August to September 2023, 550 participants were recruited. Among non‐KP, injectable PrEP was strongly preferred to oral (Figure). Side effects and costs were both strong negative drivers of PrEP use among non‐KP; preferences were similar between non‐KP men and women. Among KP, injectable PrEP remained strongly preferred to oral PrEP, side effects were a less important driver, and there were strong preferences against PrEP delivery at government clinics versus drop‐in centres. Notably, side effects did not drive FSW preferences. Overall, PrEP‐stigma was cited by >60% as the key barrier and <50% of participants found getting PrEP at HIV/STI clinics acceptable.

**Conclusions: **Injectable PrEP largely and positively drove preferences, while costs were strong negative drivers. Reinforced counselling around side effects may be particularly important among non‐KP; diverse delivery channels for PrEP are important across populations and recommended for rollout in Malawi.

### OA1303 Knowledge and acceptance of HIV pre‐ and post‐exposure prophylaxis among a cohort of cis and transgender female sex workers in Buenos Aires, Argentina

M.V. Iannantuono ^1^, M.M. Sandoval^1^, M. Loufty^2^, C.M. Romanelli^1^, C. Cesar^1^, A. Nava^1^, M. Ceschel^1^, M.E. Esandi^1^, J. García^1^, D. Sallusso^1^, M. Romero^3^, G. Orellano^4^, V. Salazar^1^, I. Arístegui^1^, M.I. Figueroa^1^, Z. Ortiz^1^, A. Durán^5^, S. Walmsley^6^

^1^Fundación Huésped, Research Department, Ciudad Autonoma de Buenos Aires, Argentina, ^2^Women´s College Hospital, University of Toronto, Toronto, Canada, ^3^Asociación de Travestis, Transexuales y Transgénero de Argentina, Buenos Aires, Argentina, ^4^Sindicato de Trabajadorxs sexuales de Argentina, Buenos Aires, Argentina, ^5^Coordination of Sexual Health, HIV and Sexually Transmitted Infections of the Ministry of Health of Buenos Aires, Buenos Aires, Argentina, ^6^University Health Network, Toronto, Canada

**Background: **Combined strategies for HIV prevention include oral pre‐ and post‐exposure prophylaxis (PrEP and PEP). Sex workers are at high risk of acquiring HIV; however, they face barriers to access healthcare services, including HIV prevention. This study aimed to assess the knowledge of PrEP and PEP, and the acceptance of PrEP in a cohort of female sex workers (FSWs).

**Methods: **“MAS por Nosotras” is an ongoing prospective cohort of FSW recruiting at a non‐governmental organization in Argentina. Medical and psychosocial information is obtained from consenting participants at baseline and each visit including structured questions on PrEP and PEP. Participants are tested for HIV and PrEP is offered for those who test negative. Baseline data are presented.

**Results: **From June to December 2023, 116 FSW were enrolled: 55 cisgender women (CGW) and 61 transgender women (TGW). Median age was 33.5 years (IQR 26.8−44.3). Median years of sex work was 12 [IQR 6−19], with 51.7% of TGW and 26% of CGW with >20 sexual partners in the prior month and 59.5% reporting condomless anal/vaginal intercourse. 21/116 knew they were living with HIV and three other participants were diagnosed at baseline (23/24 TGW). Among those without HIV, 22.1% knew about PEP and 9.5% had used it before. 26.8% TGW were on PrEP at baseline. Among those not receiving PrEP at enrolment, TGW had used PrEP in the past more frequently than CGW (20% vs. 1.9%, *p* = 0.008) and reported more knowledge on what PrEP was used for (53.3% vs. 14.8%, *p*<0.001). When PrEP was offered, acceptance was higher among TGW than CGW (66.7% vs. 33.3%, *p* = 0.003). Reasons for not initiating PrEP included not feeling at risk, potential adverse events, not desiring taking medication and postponing PrEP for another time.

**Conclusions: **A quarter of FSW had previous knowledge of PrEP, lower in CGW than TGW. The acceptance was high among TGW but very low among CGW. Future research should focus on understanding barriers to access HIV prevention in CGW and TGW, in order to develop tailored HIV prevention policies.

### OA1304 The persistent chasm between PrEP awareness and uptake: characterizing the biomedical HIV prevention continuum in a nationwide cohort of transgender women in the United States

E. Cooney ^1^, T. Poteat^2^, M. Stevenson^3^, A. Lint^4^, A. Radix^5,6^, A. Borquez^7^, K. Althoff^3^, C. Beyrer^8^, C. Brown^9^, L. Ragone^9^, V. Vannappagari^9^, S. Reisner^10^, A. Wirtz^3^, ENCORE Study Group

^1^Johns Hopkins Bloomberg School of Public Health, International Health, Baltimore, United States, ^2^Duke University School of Nursing, Division of Healthcare in Adult Populations, Durham, United States, ^3^Johns Hopkins Bloomberg School of Public Health, Epidemiology, Baltimore, United States, ^4^Arianna's Center, Fort Lauderdale, United States, ^5^Callen‐Lorde Community Health Center, New York, United States, ^6^Columbia University, Department of Epidemiology, New York, United States, ^7^University of California San Diego, Division of Infectious Diseases and Global Public Health, La Jolla, United States, ^8^Duke University, Duke Global Health Institute, Durham, United States, ^9^ViiV Healthcare, Durham, United States, ^10^University of Michigan, Epidemiology, Ann Arbor, United States

**Background: **Transgender women (TW) are disproportionately impacted by HIV, yet data on the biomedical HIV prevention continuum (HIVPC) among TW are limited. We characterized the HIVPC among a large, nationwide cohort of TW by PrEP modality (oral and long‐acting injectable, LAI) and identified correlates of uptake and adherence.

**Methods: **From March 2023 to February 2024, we enrolled English‐ and Spanish‐speaking adult TW without HIV (laboratory‐confirmed) into the ENCORE cohort. PrEP data were collected via self‐reported surveys. Descriptive statistics characterized the HIVPC and modified Poisson regression models estimated adjusted prevalence ratios (aPR) and 95% confidence intervals (95% CI) for correlates of HIVPC progression (awareness‐uptake‐adherence). We assessed differences in proportions for each step of the HIVPC by modality.

**Results: **We enrolled 996 participants (mean age = 35.6 years; range = 18−80); 60% were non‐Hispanic White, 17% Black, 15% Latina/x, 6% Indigenous and 5% Asian. Among the 62% who were sexually active (past 6 months), 91% had ever heard of PrEP, 36% had ever used PrEP, 25% recently used PrEP (past 6 months) and 20% were adherent. The largest proportional difference in HIVPC progression was from awareness to uptake (62% of sexually active, PrEP‐aware TW had never used PrEP). This difference was significantly greater in the LAI continuum (96% of LAI PrEP‐aware TW had never used LAI). Correlates of PrEP uptake included receiving health services at an LGBTQ clinic (aPR = 1.5; 95% CI = 1.2−1.9), current hormone use (aPR = 1.8; 95% CI = 1.2−2.5) and higher perceived HIV acquisition risk (aPR = 1.5; 95% CI = 1.1−2.2). Correlates of non‐adherence included higher anticipated discrimination (aPR = 1.1; 95% CI = 1.0−1.1). Among 14 TW who had used LAI PrEP, mean age was 37 years (range = 21−59), 7% (1/14) identified as Black and 21% (3/14) Latina/x. Twenty‐one percent (3/14) were HPTN 083 participants and the remaining 79% (11/14) initiated LAI PrEP in 2023.

**Conclusions: **Interventions to improve HIVPC outcomes—especially PrEP uptake—are needed to optimize PrEP among transgender women.[Fig jia226351-fig-0011]

**Figure 1 jia226351-fig-0011:**
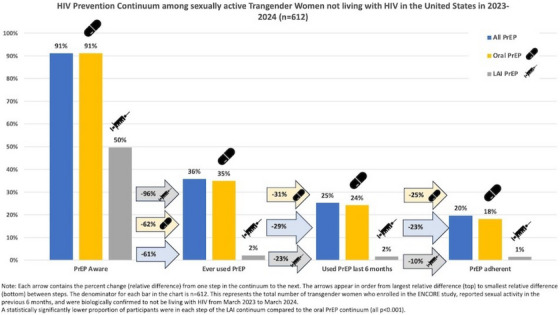
**OA1304**

### OA1305 At elevated risk within these four walls: Expanding access to pre‐exposure prophylaxis to incarcerated males in Zambia

M. Musonda ^1^, L. Kawanga^2^, A. Ndhlovu^2^, J. Musangulule^2^, P. Mundende^2^

^1^United States Agency for International Development, Health Office/Zambia, Lusaka, Zambia, ^2^JSI DISCOVER‐Health, Lusaka, Zambia

**Background: **HIV prevalence in Zambia among incarcerated populations is higher than the community outside. Once as high as 27% in 2009, it has decreased to 14.3%. Reasons for higher risk include poor access to health services, unsafe sex between incarcerated males, sexual assault and the use of contaminated needles for tattoos. In 2019, the provision of pre‐exposure prophylaxis in prisons was introduced, expanding access to effective biomedical prevention to incarcerated populations.

**Methods: **The introduction of PrEP in prisons was initially hampered by the restrictive legal environment around homosexuality in Zambia. Through quiet advocacy and emphasis on other risk factors, authorization was provided in 2019 for PrEP to be provided to incarcerated populations as part of HIV services offered in 26 Zambia Correctional Services (ZCS) facilities supported by the USAID DISCOVER‐Health project. ZCS officers were trained as HIV/AIDS coordinators to oversee HIV/AIDS activities in prisons. Influential incarcerated males are nominated to work as peer educators. These peer mentors, 30 per facility, provide key messaging to other inmates and generate demand for PrEP. Peer mentors hold small group discussions and individual counselling sessions on combination HIV prevention and linkage to PrEP. Peer mentors are also equipped to provide psychosocial support to fellow incarcerated males to improve continuation on PrEP, including safekeeping of PrEP drugs and providing reminders to those on PrEP.

**Results: **In 2019, ZCS facilities supported by USAID DISCOVER‐Health initiated 62 male inmates on PrEP. In 2023, expansion to additional correctional facilities resulted in 3815 inmates initiated on PrEP. In total, from 2019 to 2023, 8392 inmates have been initiated on PrEP. Of the 8392 inmates initiated on PrEP, 514 were 15−19 (6%), 1833 were 20−24 (22%), 5523 were aged 25−29 (67%) and 422 (5%) were aged >50 years old.

**Conclusions: **PrEP provision is critical in averting HIV acquisition during incarceration. Empowering incarcerated individuals to be peer mentors who are equipped as sources of key messaging on PrEP ensures that incarcerated males have access to accurate information on HIV prevention to help avert HIV acquisition. Safeguarding the health of incarcerated populations in turn ensures gains made in reducing HIV transmission in communities are maintained.

### OA1306 Greater PrEP use in a community‐based needle and syringe programme versus facility‐based medication for opioid use disorder programme for people who use drugs in Uganda

R. Heffron ^1^, J. Kibuuka^2^, T. Muwonge^2^, T. Wood^1^, J. Hao^1^, M. Nakitende^2^, F. Nambi^2^, L. Nakabugo^2^, P. Smith^1^, R. Kansiime^3^, G. Pande^3^, P. Kyambadde^3^, L. Komuhangi^2^, H. Kadama^4^, P. Mudiope^4^, B. Lambdin^5^, S. Glick^6^, D. Donnell^6^, A. Mujugira^2^

^1^University of Alabama at Birmingham, Medicine, Birmingham, United States, ^2^Makerere University, Infectious Diseases Institute, Kampala, Uganda, ^3^Most At Risk Persons Initiative, Kampala, Uganda, ^4^Ministry of Health, Kampala, Uganda, ^5^RTI International, Research Triangle Park, United States, ^6^University of Washington, Seattle, United States

**Background: **HIV pre‐exposure prophylaxis (PrEP) is critical for people who use drugs in high HIV‐burdened settings and could be integrated into existing harm reduction services—needle and syringe (NSP) and medication for opioid use disorder (MOUD) programmes. In Uganda, we integrated oral PrEP into a community‐based NSP and facility‐based MOUD programme and compared PrEP refills and adherence.

**Methods: **PrEP delivery was integrated into Uganda's only public MOUD programme (within Butabika Mental Health Hospital) and a non‐profit community‐based NSP. Harm reduction providers were trained on PrEP, programmes received HIV testing commodities and PrEP trainers provided continuous technical assistance. All HIV‐negative people accessing each harm reduction programme were offered PrEP. Those initiating had PrEP dispensing data abstracted from medical records for 12 months and the opportunity to enrol in research procedures (quarterly blood draws for tenofovir [TFV] quantification and behavioural questionnaires). In age‐adjusted Cox proportional hazards models, we compared time to first PrEP discontinuation (using definitions for strict and real‐world discontinuation) among people within each programme.

**Results: **Among 417 participants (325 from NSP, 92 from MOUD), 91% were male, median age = 31 (IQR 25−40), and cannabis, cocaine and street opioids were drugs most commonly used. Injection was the most common route for opioid use and 19.9% of participants had shared injection equipment. After the first visit, 83% had a subsequent refill, after the second, 79%; subsequent visits had a 67% refill rate. TFV was detected in 54% of visits after PrEP dispensing (28% had TFV >40 ng/ml) and similar among NSP and MOUD. The median time to first discontinuation was 1 month (IQR 1−3) for MAT and 4 (IQR 1−6) for NSP participants. Using a real‐world definition based on 2 consecutive months without PrEP coverage, the discontinuation rate was statistically lower among people in the NSP versus MOUD (hazard ratio 0.16, 95% CI 0.12−0.21). Results using a stricter definition had higher discontinuation rates and similar difference between the programmes (*p*<0.001).

**Conclusions: **Initial PrEP use was good to modest among people accessing harm reduction services in Uganda. While both programmes delivered PrEP to this high‐risk population, the community‐based NSP may yield better PrEP continuation than the MOUD programme.

### OA1402 “*They are not HIV treatments drugs; they are preventive drugs (PrEP)*.” Experiences of using PrEP among vulnerable adolescent girls and young women in Tanzania

M.T. Mwakilasa ^1,2^, A. Mwijage^1^, S. Mushy^3^, M. Amour^4^, N. Sirili^5^, E. Maziku^6^, S. Likindikoki^7^, E. Balandya^8^, G. Kwesigabo^9^, B. Kidenya^10^, S.E. Mshana^11^, E. Lyamuya^12^, B. Mmbaga^13^, B. Sunguya^4^, J. Bartlett^14^

^1^Muhimbili University of Health and Allied Sciences, Dar es Salaam, the United Republic of Tanzania, ^2^University College Dublin, School of Nursing, Midwifery and Health System, Dublin, Ireland, ^3^Muhimbili University of Health and Allied Sciences, Department of Community Health Nursing, School of Nursing, Dar es Salaam, the United Republic of Tanzania, ^4^Muhimbili University of Health and Allied Sciences, Department of Community Health, School of Public Health and Social Sciences, Dar es Salaam, the United Republic of Tanzania, ^5^Muhimbili University of Health and Allied Sciences, Department of Development Studies, School of Public Health and Social Sciences, Dar es Salaam, the United Republic of Tanzania, ^6^Ministry of Health, HIV Prevention Unit, Dodoma, the United Republic of Tanzania, ^7^Muhimbili University of Health and Allied Sciences, Department of Psychiatry, School of Medicine, Dar es Salaam, the United Republic of Tanzania, ^8^Muhimbili University of Health and Allied Sciences, Department of Physiology, Dar es Salaam, the United Republic of Tanzania, ^9^Muhimbili University of Health and Allied Sciences, Department of Epidemiology and Biostatistics, School of Public Health and Social Sciences, Dar es Salaam, the United Republic of Tanzania, ^10^Catholic University of Health and Allied Sciences, Department of Microbiology, Mwanza, the United Republic of Tanzania, ^11^Catholic University of Health and Allied Sciences, Department of Biochemistry and Molecular Biology, Mwanza, the United Republic of Tanzania, ^12^Muhimbili University of Health and Allied Sciences, Department of Microbiology, Dar es Salaam, the United Republic of Tanzania, ^13^Kilimanjaro Christian Medical University College, Faculty of Medicine, Kilimanjaro, the United Republic of Tanzania, ^14^Duke University, Duke Global Health Institute, Durham, United States

**Background: **HIV poses a significant global health concern, affecting adolescents among other populations. This is attributed to various vulnerabilities including biological factors, gender inequalities and limited access to comprehensive sexual and reproductive health services in sub‐Saharan Africa. In Tanzania, adolescent girls and young women (AGYW) face double the risk of HIV infection compared to their male counterparts The introduction of pre‐exposure prophylaxis (PrEP) brought hope for changing the HIV cascade in the country. However, numerous challenges still hinder PrEP uptake. Therefore, this study explores the experiences of PrEP uptake among vulnerable AGYW in Tanzania.

**Methods: **This study employed a phenomenological qualitative approach; 52 semi‐structured interviews were carried out between May and November 2022 in the selected healthcare facilities in Tanzania. The study adopted inductive‐deductive thematic analysis guided by the Social Ecological Model (SEM) to elicit the views of AGYW aged 15–24. Nvivo software was utilized to organize data.

**Results: **This study has uplifted barriers and facilitators on PrEP uptake among AGYW in Tanzania. The barriers are categorized at individual, interpersonal and institutional levels. The individual‐level barriers included pre‐requisites for initiating PrEP, disbelief in the effectiveness of PrEP, interference of refill hours with working hours, financial constraints and adherence to the pills. The interpersonal‐level barriers included misconceptions about PrEP pills and labelling of PrEP users. The institutional‐level barriers included inadequate privacy, PrEP drug stockout, being turned away by healthcare facilities (HCFs), long waiting times and distance to the HCF. Facilitators included factors at individual level (experienced benefit of PrEP, adequate PrEP knowledge, having multiple partners, perceived risk due to the nature of the work, PrEP ensuring privacy), interpersonal level (support from social networks) and institutional level (accessibility of PrEP, receiving refill reminders).

**Conclusions: **To overcome barriers to PrEP uptake among AGYW, it is crucial to develop multi‐level interventions that consider personal, social and structural factors hindering PrEP uptake. Implementing strategies like prioritizing off‐site PrEP delivery and expanding community outreach for PrEP awareness can help dispel misconceptions and enhance uptake.

### OA1403 The impact of the “Le Kip Kip” social influence campaign on PrEP knowledge, attitudes and perceptions among women in South Africa

V. Chen ^1^, B.A.S. Nonyane^1^, C. Singh^2^, L. Shipp^3^, J. McLoughlin^4^, M. Mcingana^5^, N. Dladla^6^, N. Matenjwa^6^, K. Rucinski^1^, A. Rao^3^, K. Atkins^1^, J.G. Rosen^1^, S. Baral^3^, H. Hausler^5,7^, S. Schwartz^3^

^1^Johns Hopkins Bloomberg School of Public Health, International Health, Baltimore, United States, ^2^TB HIV Care, uMgungundlovu, South Africa, ^3^Johns Hopkins Bloomberg School of Public Health, Epidemiology, Baltimore, United States, ^4^TB HIV Care, Priority Populations Prevention Programme, uMgungundlovu, South Africa, ^5^TB HIV Care, Cape Town, South Africa, ^6^TB HIV Care, Durban, South Africa, ^7^University of Pretoria, Department of Family Medicine, Pretoria, South Africa

**Background: **Strategies addressing PrEP stigma are needed to facilitate PrEP uptake and persistence among women, including adolescent girls and young women (AGYW) and female sex workers (FSWs). We evaluated the effect of the “Le Kip Kip” social influence campaign, implemented as part of a cluster randomized trial (CRT), on PrEP knowledge, attitudes and perceptions in South Africa.

**Methods: **We conducted cross‐sectional baseline (May−June 2022) and endline (October−November 2023) surveys among AGYW and FSWs receiving decentralized HIV prevention services from TB HIV Care in 6/10 programme districts involved in the CRT. Outcomes included PrEP knowledge (dichotomous), negative PrEP‐user stereotypes (mean of 6 Likert scale items) and anticipated PrEP approval by others (mean of 3 Likert scale items). We conducted difference‐in‐differences analyses using modified Poisson and linear regression models to quantify potential impacts of the intervention on each outcome, adjusting for age and education.

**Results: **Among 1201 women (601 baseline; 600 endline) surveyed (42% AGYW, 58% FSW), PrEP knowledge, PrEP‐user stereotypes and PrEP approval improved over time, but changes were not statistically significant (Figure 1). PrEP knowledge significantly increased over the study period in intervention districts relative to control districts among AGYW (PRR: 4.72, 95% CI: 3.17, 7.05), but not FSW (PRR: 1.20, 95% CI: 0.28, 5.14). Expected PrEP approval from social networks increased in intervention districts relative to control districts in unadjusted analyses among FSWs (b: 0.26, 95% CI: 0.04, 0.47) but not AGYW (b: 0.32, 95% CI: −0.58, 1.23).

**Conclusions: **The “Le Kip Kip” campaign increased PrEP knowledge for decision‐making among AGYW, and positively nudged attitudes towards PrEP within FSW's social networks overall. PrEP‐stereotype scores among FSW and AGYW within communities were not affected. Persistent PrEP stigma manifesting in negative stereotypes warrants complementary interventions enabling PrEP uptake and persistence among women with sustained HIV vulnerabilities in South Africa.[Fig jia226351-fig-0012]

**Figure 1 jia226351-fig-0012:**
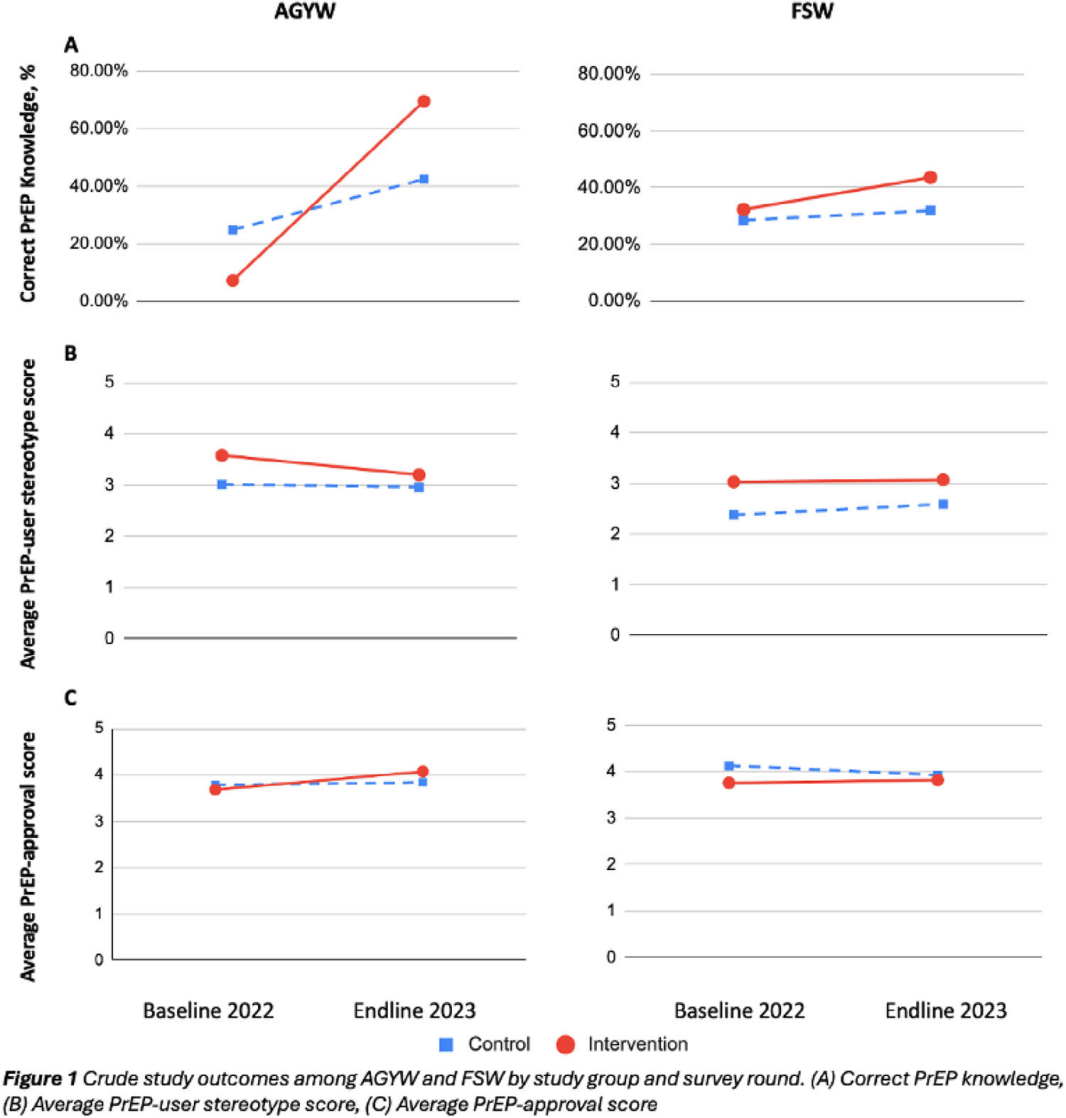
**OA1403**

### OA1404 The influence of male partners on pregnant women's participation in HIV prevention trials: experiences from the MTN‐042 dapivirine vaginal ring and oral PrEP study in Kampala, Uganda

T. Nakyanzi ^1^, S. Nanyonga^1^, S.C. Nanziri^1^, D. Kemigisha^1^, R. Kirungi^2^, C. Nakabiito^1^, B.G. Mirembe^1^, P. Kibalama Ssemambo^1^

^1^Makerere University Johns Hopkins University Research Collaboration, Psychosocial Support, Kampala, Uganda, ^2^Nabweru Health Centre Youth Health Club, Reproductive Health, Kampala, Uganda

**Background: ** Male partners are key health decision‐makers in their families, yet their perceptions about the participation of their pregnant partners in research are not widely known. The MTN‐042/DELIVER study assessed the safety, adherence and acceptability of the monthly dapivirine vaginal ring (DVR) and daily oral tenofovir/emtricitabine when used during pregnancy. Considering the delicate nature of pregnancy and participants’ socio‐cultural settings, MTN key stakeholders recommended that researchers engage with male partners to the extent possible. Recruitment started with third‐trimester cohort, followed by second and first‐trimester cohorts. Here, we describe male partners’ perceptions and influence on pregnant partners’ decisions to participate in HIV prevention research.

**Methods: ​**Potential participants were asked whether they had engaged their male partners about the DELIVER study and what their partners’ reactions were, whether they approved participation or not. For enrolled participants, partners were invited to participate in two of four engagement sessions. The session's objective was to explore perceptions and the influence of male partners on pregnant women's interest in research participation. The content was coded, summarized and thematically analysed. Male partner responses were quantitatively captured from the participant appointment tracking logs and updated regularly.

**Results: **A total of 1183 potential participants were pre‐screened; 344 male partners were approached by their spouses for permission to participate in DELIVER. The majority (88%) were unsupportive; 39.5% (*n* = 136) refused participation, and 48.8% (*n* = 168) switched off phones denying their pregnant partner's participation. Only 11.6% (*n* = 40) accepted study participation and engaged in the male partner sessions. Reasons for male partner refusal included: (1) safety concerns about study products causing stillbirths, abortions and congenital anomalies, especially during early and late trimesters; (2) rumours, and misconceptions about the depopulation of Africans through HIV prevention research; (3) lack of PrEP awareness; and (4) low HIV risk perceptions. Male partners who perceived themselves to be at high risk accepted participation and wanted to know about PrEP and to have HIV‐free babies.

**Conclusions: **Awareness about PrEP is urgently needed among male partners to facilitate uptake among pregnant women who need HIV prevention methods but lack the autonomy to protect themselves and their babies from acquiring HIV.

### OA1405 Impact of community—health facility electronic bidirectional client referral services in Ethiopia

T.S. Gebrehiwot ^1^, G.A. Biks^1^, D.A. Tsegaye^1^, L.A. Mekuria^1^, S. Mookherji^2^, A. Hayes^2^, E. Seid Ebrahim^1^

^1^Project HOPE, Addis Ababa, Ethiopia, ^2^Project HOPE, Washington, United States

**Background: **Effective bidirectional referral between health centres and communities is crucial for ensuring the continuity of HIV care. To improve the speed and quality of referral services in Ethiopia, Project HOPE integrated a bidirectional referral system into the unified data system (UDS) for data collection via CommCare. We summarized the electronic exchange of patient information and its application in Ethiopian community HIV care and treatment activities.

**Methods: **Project HOPE developed a bidirectional referral application on the CommCare platform for the exchange of patient information. The application runs on mobile devices, such as smartphones and tablets. User accounts were created for community and facility health workers. They received bidirectional CommCare data entry training, tablets for data collection and ongoing technical support. Over 30 implementing partners were involved. Trained health facility and community health workers conduct two‐way electronic referrals based on client needs. This includes feedback about services. A retrospective analysis summarized the outcome of the bidirectional electronic referral system from 2020 to 2023. The analysis focused on 772 selected high‐load health facilities in Ethiopia.

**Results: **A total of 130,000 client data were exchanged among the community and health facility care providers including interruption in treatment, contacts of HIV‐positive index cases, high viral loads among people living with HIV, cervical cancer referrals, community care and support, and 95% of the clients have got the service with confirmed feedbacks. The referral system improved communication and feedback between community and health facility workers, alerted users with notifications, provided a job aid guide and used validations to improve the quality of healthcare services to people living with HIV. All implementing partners used the UDS on a regular basis to monitor their performance, report to USAID and PEPFAR's DATIM system, and make timely decisions at all levels.

**Conclusions: **The electronic bidirectional referral system improved the community health facility collaboration to provide holistic care and timely and consistent HIV services at all levels, strengthen programme data quality, and promote data use and real‐time communications. Therefore, we recommend the wider scaleup of digital platforms for bidirectional referral services to strengthen and support community‐based HIV service delivery in resource‐limited settings.

### OA1406 Factors leading to viral load non‐suppression after intensive adherence counselling among HIV patients in TASO Mbale Eastern Uganda. A cross‐sectional study

A. Apio ^1^, D. Soita^1^, S. Ojera^1^

^1^Busitema University, Public Health, Kampala, Uganda

**Background: **Globally, 37.7 million people are living with HIV and of these, only 66% on antiretroviral treatment (ART) are suppressed. According to the United Nations Program on HIV/AIDS through the ambitious 95‐95‐95 target, 95% of people living with HIV (PLHIV) on ART should be virally suppressed by 2025 to eliminate HIV as a public health problem by 2030. Viral load (VL) non‐suppression is associated with poor adherence, and intensive adherence counselling (IAC) has shown to improve suppression.

**Methods: **This was a cross‐sectional study, conducted in TASO Mbale. Data were abstracted for all clients with non‐suppressed viral load between Jan 2018 and August 2021 from the TASO Mbale program database and various parameters were considered. These parameters included the IACs done, the time the repeat VL was done, presence of an opportunistic infection, sex, age and the current regimen. A client was considered to have a non‐virological status if there were >1000 copies/ml. The clients were followed for a maximum period of 43 months. A total of 13,428 person months was observed for different periods. The data were then analysed using statistical software Stata version 14. Bivariate analysis was done for all covariates. Hazard ratios (HRs) were estimated as a degree of association between viral non‐suppression and client features, via a Cox proportional hazards regression.

**Results: **A total of 442 PLHIV with unsuppressed VL were enrolled and underwent IAC of whom 60 (13.6%) had VL non‐suppression after IAC. The overall rate of VL non‐suppression was 4.47 (3.41−5.75) per 1000 person‐months (PM) of observation after IAC. ART regimen was statistically significant with viral load suppression especially DTG‐3TC‐LPV/r‐based regimen (AHR = 5.78, 95% CI, 1.137−29.384, *p* = 0.034). Poor adherence level to ART had a significant contribution to viral load non‐suppression (AHR = 4.88, 95% CI: 1. 607−14.836, *p* = 0.005).

**Conclusions: **Virological non‐suppression after IAC was found at 13.6% and associated with patients’ poor adherence levels. Viral load suppression was associated with good adherence, to ART drug regimens specifically DTG‐3TC‐LPV/r and AZT‐3TC‐LPV/r‐based regimens. The findings lead to a huge boost towards the third UNAIDS target of 95%.

### OA1502 Defining the contribution of HIV‐1 subtype C CD4‐binding site mutations to VRC01 resistance and viral fitness

K.N. Mabombo ^1^, B. Lambson^2^, C. Williamson^3^, L. Morris^2^, P.L. Moore^2^, H.T. Mufhandu^1^

^1^North West University, Microbiology, Mahikeng, South Africa, ^2^National Institute for Communicable Diseases, Centre for HIV and STI, Johannesburg, South Africa, ^3^University of the Witwatersrand, Health Sciences, Johannesburg, South Africa

**Background: **Human immunodeficiency virus type 1 (HIV‐1) continues to be a threat to the public health globally with about 36.7 million people living with the virus. The development of a preventive vaccine is a challenge to the public health.

Individuals from the Antibody Mediated Trial (AMP) [HVTN 704/HPTN 085 and HVTN 703/HPTN 081] were infused with VRC01, a bNAb targeting CD4‐binding sites, every 8 weeks. While it did not decrease HIV‐1 acquisition, highly sensitive isolates showed successful bNAb prophylaxis.

In this study, features of the envelope gene associated with VRC01 neutralization resistance were mutated to generate VRC01‐sensitive viruses and sensitivity was regarded as a confirmation of the resistant sites.

**Methods: **Putative VRC01 novel mutations were generated by identifying VRC01 resistant HIV‐1 subtype C envelope sequences. VRC01‐sensitive pseudovirus mutants were generated by site‐directed mutagenesis. Neutralization assay was performed to measure VRC01 inhibition of the sensitized viruses compared to wildtypes.

**Results: **Neutralization of double (F717L/I646L) and triple mutations (F317I/646L/K683R) was tested. The H703_0842 (F717L/I646L) became neutralization sensitive to VRC01, 3BNC117, PG9 and PGT151 compared to their H703_0842 wildtype. The triple mutation was introduced in the H704_0482 virus (F317I/646L/K683R) and was resistant to VRC01 and sensitive to 3BNC117, PG9 and PGT151, compared to its wildtype.

**Conclusions: **Neutralization resistance of subtype C strains against VRC01 was identified with possible HIV‐1 escape sites in the V3 region, gp41 transmembrane and the C2 region. The study confirmed that some mutations found in gp120 significantly contribute to neutralization by the VRC01.Thus, investigating resistance mechanisms against VRC01 may shed more light on antibody neutralization escape of HIV‐1.

### OA1503 A higher proportion of recent compared to historic HIV viruses are resistant to antibody‐dependent cellular cytotoxicity mediated by monoclonal antibodies

D. Mielke ^1^, E.E. Giorgi^2^, K. Faircloth^1^, T. Eaton^1^, B. Dunn^1^, A. Zalaquett^1^, S. Standfield‐Oakley^1^, E. Domin^1^, D. Montefiori^1^, B. Korber^3^, G. Tomaras^1^, G. Ferrari^1^

^1^Duke University, Surgery, Durham, United States, ^2^Fred Hutch Cancer Center, Seattle, United States, ^3^Los Alamos National Laboratory, Los Alamos, United States

**Background: **HIV diversity represents a significant barrier to effective prevention and treatment strategies; including to the use of monoclonal antibodies (mAbs) which are currently being assessed in clinical trials.

**Methods: **Phylogenetic analysis of 1380 subtype C *envelope* sequences sampled from 1988 to 2021 shows that later sequences (2015−2021) are significantly more diverse than earlier sequences. To determine if increased diversity impacted antibody‐dependent cellular cytotoxicity (ADCC) mediated by mAbs, we constructed infectious molecular clones (Env‐IMCs) containing 11 historic (1998−2008) and 15 recent (2017−2021) *env* sequences that best represented global diversity in C1C2, V1V2 and CD4‐binding site regions. ADCC susceptibility was tested against 16 neutralizing and non‐neutralizing mAbs targeting V2 apex, V3 glycan, CD4‐binding site, MPER, fusion peptide and CD4‐induced epitopes.

**Results: **Overall, only PGT121 had significantly less ADCC activity against recent viruses compared to historic ones (*p* = 0.004, FDR q = 0.06). However, only 1/11 (9%) historic viruses versus 5/15 (33%) recent viruses were resistant to ADCC mediated by the mAb panel (pAUC>50 for <2 mAbs; binomial *p* = 0.009). Phylogenetically corrected signature analysis showed that 379R and 496V were significantly associated with ADCC sensitivity, whereas 379G and 496I were associated with ADCC resistance (the latter not significant after multiple testing correction). Interestingly, both 379G and 496I increase in frequency over time when comparing historic envelopes to the more recent ones, whereas the sensitive residues, 379R and 496V, become less prevalent over time.

**Conclusions: **These data suggest that HIV is evolving to become more resistant to ADCC. Consequently, careful selection of mAb combinations and vaccine immunogens will be needed to successfully prevent and treat HIV‐1 acquisition.

### OA1504 Neutralization sensitivity of currently circulating HIV‐1 India clade C to broadly neutralizing antibodies targeting CD4bs and V3 glycan supersite

P. Jayal ^1^, R. Mullick^1,2^, J. Sutar^1,2^, S. Deshpande^1^, S. Chauhan^1^, S. Mukherjee^1,2^, P. Kamble^1^, S. Srivas^1^, C. Prasad^1^, N. Sharma^1^, N. Kasarpalkar^3^, S. Bhowmick^3^, K. Karandikar^3^, P. Devadiga^3^, V. Bhor^3^, D. Kale^4^, D. Biswas^4^, S. Mondal^5^, B. Bandyopadhyay^5^, S.K. Guha^5^, R. Pranjape^6^, A. Kondapi^7^, S. Agrawal^8^, J. Shastri^8^, S. Mohapatra^9^, T.R. Dinesha^9^, A.K. Srikrishnan^9^, D. Sok^10^, K. Murugavel^9^, V. Patel^3^, J. Bhattacharya^1^

^1^Translational Health Science and Technology Institute, Faridabad, India, ^2^IAVI, New York, United States, ^3^National Institute for Research in Reproductive and Child Health, Mumbai, India, ^4^AIIMS, Bhopal, India, ^5^School of Tropical Medicine, Kolkata, India, ^6^KLEAHER, Belagavi, India, ^7^University of Hyderabad, Hyderabad, India, ^8^BYL Nair Hospital, Mumbai, India, ^9^YRGCARE, Chennai, India, ^10^IAVI‐NAC, La Jolla, United States

**Background: **The disease burden of HIV‐1 is quite high, and currently, no preventive vaccine is available. Broadly neutralizing antibodies have proven to be an effective modality in controlling infections as observed in the preclinical studies. Hence, for the clinical application of bnAbs as preventive or therapeutic intervention, it is imperative to study the effectiveness and the efficiency of relevant bnAbs against the currently circulating HIV‐1 variants. This would not only help optimize best bnAb combination but also the vaccine approach for a given population. Thus, this study was carried out with the aim to examine the neutralization phenotype of contemporary circulating HIV‐1 India clade C virus across different regions and risk groups by a panel of clinically relevant bnAbs. A comparative and comprehensive analysis was also carried out with the historical viruses collected prior to 2015.

**Methods: **For this study, samples were collected from (*n* = 161) people living with HIV (PLHIV) across five different clinical sites in India, for the year 2019−2023. The HIV‐1 envelope gene could be amplified, cloned and pseudoviruses were generated successfully from the 88 of the samples. These 88 pseudoviruses were assessed *in vitro* with different classes of bnAbs for neutralization sensitivity. A comparative study with historical viruses focusing on CD4bs and V3‐directed bnAbs was also carried out to study the phenotype evolution.

**Results: **The neutralization assessment of contemporary HIV‐1 viruses exhibited most sensitivity to CD4bs and V3 glycan supersite‐directed bnAbs among all the classes. Although comparative analysis with historical virus neutralization data revealed increasing resistance to the 3BNC117 (CD4bs) and BG18 (V3 glycan) bnAbs. Several broadly resistant viruses were also identified which were resistant to all but one class of bnAbs (IC50>25 µg/ml).

**Conclusions: **Although the neutralization assessment revealed most sensitivity to the CD4bs and V3‐directed class of bnAbs, yet increasing resistance was also observed to certain individual bnAbs. This neutralization assessment along with identification of broadly resistant viruses warrants continual virus surveillance among PLHIV to optimize bnAb combination with highest coverage and multiple specificity. Thus, a single preventive/therapeutic combination may not be a gold standard, rather population‐specific intervention needs to be strategized.​

### OA1505 Neutralization sensitivity of African HIV‐1 isolates from diverse clades to clinically relevant bNAbs

D. Kitchin ^1^, J. Serwanga^2^, J. Sutar^3^, S. Rasebotsa^1^, T. Naidoo^1^, N. Mzindle^1^, T. Motlou^1^, Z. Makhado^1^, D. Mhlanga^1^, R. van Dorsten^4^, S. Balinda^2^, E. Landais^5^, E. Karita^6^, W. Kilembe^7^, T. Ndung'u^8^, P. Kaleebu^2^, D. Sok^5^, P. Moore^1^

^1^National Institute for Communicable Diseases, Centre for HIV and STIs, Johannesburg, South Africa, ^2^Uganda Virus Research Institute, Entebbe, Uganda, ^3^International AIDS Vaccine Initiative, Faridabad, India, ^4^University of the Witwatersrand, Johannesburg, South Africa, ^5^International AIDS Vaccine Initiative, Neutralising Antibody Centre, San Diego, United States, ^6^Rwanda Zambia Health Research Group, Kigali, Rwanda, ^7^Zambia‐Emory HIV Research Project, Lusaka, Zambia, ^8^University of Kwa‐Zulu Natal, Durban, South Africa

**Background: **Broadly neutralizing antibodies (bNAbs) are a major focus for HIV vaccine design and passive immunization for HIV prevention. Given the significant genetic diversity of the African HIV‐1 epidemic, assessing which bNAbs will be most effective against circulating strains in each region is essential. Studies have shown that over the course of the maturing clade C and B epidemics, recently transmitted viruses show reduced sensitivity to bNAbs. However, it is not known if this is the case for the A1 and D clades, and A1D recombinants, which predominate in East Africa.

**Methods: **We characterized the neutralization sensitivity of 183 African, recently transmitted viruses, from the IAVI‐ADVANCE network, isolated between 2005 and 2020, against potent CD4bs (N6 and 1−18), V2‐Apex (CAP256.VRC26.25 and ePGDM1400.V9) and V3‐glycan‐directed (ePGT121.V1 and BG18) bNAbs. We next identified the 10 most difficult‐to‐neutralize isolates from each clade, and assessed these against a larger panel of 10 bNAbs in clinical development, both individually and in combination.

**Results: **We observed subtle, but significant, changes over time in bNAb sensitivity at different epitopes; however, this varied by clade. The N6, 1−18 and ePGDM1400.V9 bNAbs showed reduced potency against later clade A1 isolates (significant for N6), while CAP256.VRC26.25 had reduced potency against later clade C isolates. In contrast, BG18 had increased potency against later isolates from both clades (significant for clade C). Overall patterns in neutralization sensitivity over time could not be evaluated for clade D and A1D recombinants due to sparse sampling coverage in later years. With the exception of clade D, the combination of VRC07‐523‐LS, ePGDM1400.V9 and ePGT121.V1 had the best coverage (80−100%) of the 10 most difficult‐to‐neutralize isolates per clade, at a concentration of 1 µg/ml. Of concern, 20−70% of difficult‐to‐neutralize isolates were only sensitive to one bNAb in the best clade‐specific combination.

**Conclusions: **Although there was a high overall coverage of difficult‐to‐neutralize isolates with bNAb combinations, we identified isolates in all clades that showed complete resistance to multiple classes of bNAbs. Such viruses with intrinsic resistance represent a challenge to bNAb‐based strategies, but could be valuable for isolating novel bNAbs, which may be more effective at constraining the emergence of resistance mutations.

### OA1506 Diminished sensitivity of historical and contemporary HIV‐1 strains to fusion peptide broadly neutralizing antibodies (bnAbs) may call for a reassessment of bnAbs efficacy

L. Kato ^1^, S. Mugaba^1^, J. Katende^1^, G. Oluka^1^, Y. Mayanja^1^, S. Balinda^1^, P. Kaleebu^1^, J. Serwanga^1^

^1^MRC/UVRI & LSHTM Uganda Research Unit, Basic Science Immunology, Entebbe, Uganda

**Background: **Recognizing the global demand for an HIV‐1 vaccine, numerous broadly neutralizing antibodies (bnAbs) targeting vulnerable epitopes on the HIV‐1 envelope have been isolated. Fusion peptide‐targeted bnAbs have been shown in non‐human primates and human trials to inhibit viral entry by blocking the required conformational changes in Env‐gp120 and gp41 subunits. We tested the hypothesis that continual genetic evolution resulting in emerging HIV‐1 envelope diversity may alter envelope glycoproteins, reducing binding and neutralization by existing fusion protein bnAbs.

**Methods: **We assessed the neutralizing capability of 10 fusion peptide‐targeted bnAbs (DFPHA, DF1Wa.01, ASC02, VRC07.01, VRC01.23, VRC34, OPVa, OPVb, OPVc and PGT151) against historical (>10 years) and contemporary (< 3 years) HIV‐1 pseudoviruses in a TZMBL neutralization assay to assess the potency and breadth of the antibodies using IC50/80 values at concentrations of 100, 2 and 0.1 µg against 226 HIV‐1 pseudotyped viruses, comprising 150 historical and 80 contemporary strains across eight clades. Proportions of neutralized historical and contemporary viruses were compared using the Wilcoxon rank test.

**Results: **At a concentration of 0.1 µg/ml, 70% (7/10) bnabs exhibited greater sensitivity to historical than contemporary viruses, with PGT151, VRC01.23, showing the highest potency at IC50, neutralizing 65% and 58% of historical viruses, respectively. Sensitivity at IC80 was higher for historical viruses (39% of 146) than for contemporary viruses (19% of 80). Significant variations occurred across clades, with A1 and C showing the highest sensitivity, while regional clades D and AD were notably underrepresented, highlighting need for focused research on locally prevalent strains.

**Conclusions: **This study highlights the dynamic change in HIV‐1's vulnerability to fusion peptide‐targeted bnAbs over time. The reducing sensitivity of contemporary HIV‐1 strains to bnAbs than their historical counterparts suggests evolutionary shifts in the virus that might impede current bnAbs‐driven interventions. The better inhibitory effects of PGT151 and VRC01.23 further strengthen the necessity for a tailored approach in antibody‐based therapies. The differential sensitivity observed across clades, and the underrepresentation of clades D and AD data, emphasizes the importance of regional‐specific studies. This study reinforces the need for continued surveillance and adaptation in bnAbs‐driven prevention strategies to remain abreast of HIV‐1's evolutionary trajectory and resistance patterns.

### OA1602 Project PEACH: Offering HIV and STI prevention options to men who have sex with men in Atlanta, Georgia, USA

O. Kaabi ^1^, A. Aldredge^2^, D.S. Carter^1^, C. DeCree^1^, E. Gardner^1^, G.B. Herring^2,3^, R. Moges‐Banks^1^, M. Padilla^1^, R. Valencia^1^, C.F. Kelley^2,3^, P.S. Sullivan^1^

^1^Emory University, Rollins School of Public Health, Epidemiology, Atlanta, United States, ^2^Emory University School of Medicine, Medicine, Division of Infectious Diseases, Atlanta, United States, ^3^Grady Healthcare System, Atlanta, United States

**Background: **In the United States, the HIV epidemic disproportionately impacts men who have sex with men (MSM), especially Black MSM. With the emergence of new sexual health prevention methods including HIV pre‐exposure prophylaxis (PrEP) and doxycycline post‐exposure prophylaxis (doxy‐PEP) for STI prevention, there is a need to better understand preferences in utilizing these strategies in combination with structured support, including Motivational Interviewing (MI).

**Methods: **Between November 2021 and September 2023, we enrolled 240 HIV‐negative MSM in Atlanta to be followed for 2 years in a prospective, observational cohort to evaluate preferences for HIV prevention options. We offered participants the option to use daily or on‐demand oral PrEP and/or doxy‐PEP alone or in combination with PrEP. In March 2023, injectable cabotegravir (CAB‐LA) became locally available and linkage to CAB‐LA services was offered. All participants also received a mobile application (app) to monitor prevention product use, sexual behaviours, desire to change prevention products and risks of PrEP or PEP discontinuation. Four‐monthly surveys, monthly/weekly in‐app quiz and quarterly HIV/STI testing were completed. Targeted MI sessions were offered based on survey responses.

**Results: **The median age among 240 participants was 30; 63% self‐identified as non‐Hispanic Black and 12% as Hispanic. At baseline, 219 participants chose PrEP (166 Daily, 43 On‐Demand, 10 Injectable), 177 of whom also requested doxy‐PEP. Sixteen participants selected doxy‐PEP alone, and five chose the app alone. There were 34 changes in prevention products over 17 months, which occurred on average 286 days from enrolment. Eleven changes occurred following an MI where participants opted to change from Daily to On‐Demand PrEP rather than discontinuing PrEP. In six changes, participants added doxy‐PEP to daily PrEP. Among those who changed prevention methods, 88% did so once. Preliminary HIV incidence is low (0.52/100 person years).

**Conclusions: **In an ongoing study of prevention preferences among MSM, we found high rates of HIV PrEP and doxy‐PEP uptake with persistence over study follow‐up. Some participants opted to change from daily to on‐demand PrEP or add doxyPEP after MI sessions. Future analyses will examine differential HIV and/or STI incidence based on prevention choice and motivators for prevention method adherence, discontinuation and change.

### OA1603 Doxycycline post‐exposure prophylaxis (DoxyPEP) real‐life effectiveness in a cohort of men who have sex with men in Milan, Italy

A.R. Raccagni ^1^, S. Diotallevi^2^, R. Lolatto^2^, E. Bruzzesi^1^, G. Catalano^1^, I. Mainardi^1^, C. Maci^1^, C. Candela^1^, C. Muccini^1^, A. Castagna^1,2^, S. Nozza^1,2^

^1^Vita‐Salute San Raffaele University, Milan, Italy, ^2^IRCCS San Raffaele Scientific Institute, Milan, Italy

**Background: **Aims are to evaluate the uptake of doxycycline post‐exposure prophylaxis (DoxyPEP) among MSM and its effectiveness against bacterial STIs (syphilis [Tp], chlamydia [Ct] and gonorrhoea [Ng]).

**Methods: **Retrospective study on MSM in care for HIV or PrEP at San Raffaele Hospital, Milan, Italy, who received DoxyPEP counselling and prescription between August 2022 (first DoxyPEP prescription, baseline‐BL) and March 2024 (freeze date). DoxyPEP was offered to people with an STI history or who reported condomless sex with ≥1 partner. DoxyPEP with doxycycline 200 mg within 72 hours from potential exposure was suggested for intense sexual activity (>5 partners). All individuals with ≥1 follow‐up visit after BL and ≥1 before BL were included. DoxyPEP uptake was self‐reported during routine visits (users:≥1 intake). Among DoxyPEP users, %‐changes in incidence rate (IR; 95% confidence interval, 95% CI) of bSTIs (Tp, Ct, Ng) before and after DoxyPEP prescription were estimated with a pre‐post intra‐patient analysis based on mixed‐effect Poisson regression (with random slope). DoxyPEP use was quantified as days‐of‐therapy (DOT) per 1000‐patient‐days (1000‐PD).

**Results: **Overall, 444 MSM (67 PLWH, 377 PrEP users) received DoxyPEP counselling and prescription; 121 (27.5%) reported DoxyPEP uptake during follow‐up. Median months of follow‐up in DoxyPEP users were 14.3 (IQR = 10.3−16.9) and 9.11 (7.04−11.6) before and after prescription, respectively. Comparison of characteristics by DoxyPEP uptake is given in Table.

Among users, 247 bSTIs (Tp: 39, Ct: 83, Ng: 125) were detected before BL and 88 (Tp: 14, Ct: 19, Ng: 55) after BL.

Regression models among users showed a significant reduction of −79% (IRR = 0.21, 95% CI = 0.16−0.27, *p*<0.001) in bSTIs’ IRs after DoxyPEP prescription compared to before: Tp:−78% (IRR = 0.22, 95% CI = 0.12−0.4, *p*<0.001), Ct:−86% (IRR = 0.14, 95% CI = 0.08−0.23, *p*<0.001), Ng:−74% (IRR = 0.26, 95% CI = 0.19−0.36, *p*<0.001), even among individuals not vaccinated with 4CMenB.

Overall, DOT per 1000‐PD was 4.02 (median DOT = 2.79) among users.

**Conclusions: **DoxyPEP uptake among MSM was relatively low but more frequent among those at higher STIs risk. A reduction in all expected bSTIs was observed among DoxyPEP users. With proper counselling, low‐level DoxyPEP use retained prophylactic effectiveness.[Table jia226351-tbl-0010]

**Table jia226351-tbl-0010:** OA1603

	Overall *n* = 444	Non‐users *n* = 323	DoxyPEP users *n* = 121	*p*‐value
**Age** (years, IQR)	37.3 [32.5; 43.1]	37.3 [32.3; 43.0]	37.4 [33.5; 43.3]	0.553
**Living with HIV**	67 (15.1%)	42 (13.0%)	25 (20.7%)	0.063
**PrEP user**	365 (82.2%)	270 (83.6%)	95 (78.5%)	0.268
**4CMenB vaccination**	66 (14.9%)	37 (11.5%)	29 (24.0%)	0.002
**At least one concomitant STI** *at BL*	93 (20.9%)	58 (18.0%)	35 (28.9%)	0.016
**At least one previous STI**	337 (75.9%)	229 (70.9%)	108 (89.3%)	<0.001

### OA1604 Community‐based organizations providing health services: nPEP, an emerging response to HIV reduction in Ecuador

H.J. Medina Matamoros ^1^, J.C. Masabanda Perez^2^

^1^Corporacion Kimirina, Comunitario, Quito, Ecuador, ^2^Kimirina, Comunitario, Quito, Ecuador

**Background: **Corporación Kimirina, a community‐based organization, provides health services for the prevention of HIV/AIDS in Ecuador, being a national reference in the detection and response to the epidemic in key populations. Considering that non‐occupational post‐exposure prophylaxis (nPEP) is considered the only strategy to reduce the risk of contracting HIV, Kimirina established an nPEP programme.

**Methods: **The post‐exposure prophylaxis programme was developed within the framework of combined prevention; medical care was provided telematically and in person: for the delivery of medication, users had the option of going to the community centres or through the field promoters, ensuring access to medication for people living in areas far away from the community centres. Medication was always available for delivery.

**Results: **During 2023, the programme covered 19 of the country's 24 provinces, with a total of 299 beneficiaries, who were found to have a negative HIV diagnosis at the end of the programme's follow‐up and control. The epidemiological profile of nPEP beneficiaries is that the vast majority (93%) are men, with an average age of 29.7 years; 38.8% did not use a condom; 9% have been diagnosed treated or have had symptoms of an STI; 2% have shared needles and syringes; 9% knew they had an HIV‐positive sexual partner; of these, 61.5% are on ARV treatment and 42.3% have an undetectable viral load.

**Conclusions: **Of the total number of people who were tested for HIV in 2023, thanks to the access of nPEP in Kimirina services, 8.5 people per thousand were prevented from aquiring HIV. The hybrid care modality was innovative and has allowed greater coverage for the initiation of treatment and linkage of these people to prevention programmes such as pre‐exposure prophylaxis.

### OA1605 Are women interested in a non‐hormonal multipurpose prevention technology (MPT) vaginal ring? Results from a national online survey with US women

A. Gottert ^1^, S. Mathur^1^, T. Abuya^2^, I. Bruce^3^, S. Shetty^4^, M. Nguyen^4^, J.M. Sales^4^, L.B. Haddad^3^, B.A. Friedland^3^

^1^Population Council, Washington, United States, ^2^Population Council, Nairobi, Kenya, ^3^Population Council, Center for Biomedical Research, New York, United States, ^4^Emory University, Atlanta, United States

**Background: **Many US women have an unmet need for/are unsatisfied with their current pregnancy/HIV/STI prevention method(s). We assessed end‐users’ preferences to inform a novel non‐hormonal multipurpose prevention technology vaginal ring (MPT‐ring) for contraception and HIV/STI prevention in development, and to learn more about women's interest in vaginal rings—the most common form of MPTs in development.

**Methods: **We conducted a cross‐sectional online survey with sexually active US women (Dec2023−Jan2024) currently/interested in using contraception, recruited via Prime Panels. We asked women about interest in a non‐hormonal ring that prevents pregnancy, HIV and STIs (at varying levels of protection); prevents bacterial vaginosis; and does not affect menstruation. We used multivariate logistic regression to examine factors associated with high product interest.

**Results: **Two thousand one hundred and five women completed the survey (mean age, 31 years; range 18−49) from all 50 states. Fifty‐three percent were married/cohabiting; 57% had ≥1 child; 43% ever had an unintended pregnancy. Sixty‐one percent said it is important their contraception be non‐hormonal. Seventeen percent ever used a contraceptive ring (4% currently using). Most said they would be very likely (33%) or likely (40%) to use the MPT‐ring at moderate levels of protection (80% for pregnancy, 50% for HIV/STIs); 76% would use it for both pregnancy and HIV/STI prevention. Thirty‐three percent would prefer using the ring continuously (its 1‐month duration); 31% at each sex act; 36% intermittently (a few days/weeks at a time). Seventy‐three percent would remove it during menses. Seventy‐one percent of all participants felt confident about ring insertion/removal, but some were concerned about placement/expulsion (56%) and/or cleaning (47%). Being “very likely” to use the MPT‐ring was associated with older age, having child(ren), perceived HIV risk, perceived STI risk, preference for non‐hormonal contraception, history of heavy menses and experience using a ring or knowing others who have. Among women unlikely to use the MPT‐ring, reasons included happiness with their current method(s) (55%) and/or wanting higher pregnancy (42%) or HIV/STI (21%) effectiveness.

**Conclusions: **We found strong interest in a non‐hormonal MPT‐ring, with heterogeneity in use preferences (e.g. indication; regimen). Women most interested in this product were older, had children, perceived HIV/STI risk, preferred non‐hormonal methods and had experience with vaginal rings.

### OA1606 An HIV‐1 risk assessment tool for men aged 15−59 years: A pooled analysis across 13 nationally representative African surveys

A. Young^1^, Y. Zou^1^, B. Shook‐Sa^1^, N. Sam‐Agudu^2,3,4^, L.‐G. Bekker^5^, L. Stranix‐Chibanda^6,7^, J. Justman^8^, B. Chi^1^, N. Rosenberg ^1^

^1^University of North Carolina at Chapel Hill, Chapel Hill, United States, ^2^University of Minnesota Medical School, Minneapolis, United States, ^3^International Research Center of Excellence, Institute of Human Virology Nigeria, Abuja, Nigeria, ^4^University of Cape Coast School of Medical Sciences, Cape Coast, Ghana, ^5^University of Cape Town, Cape Town, South Africa, ^6^University of Zimbabwe, Harare, Zimbabwe, ^7^University of Zimbabwe Clinical Trials Research Centre, HarareZimba, Zimbabwe, ^8^Columbia University, New York, United States

**Background: **Southern, Eastern and West African countries host 15% of the global population, yet account for more than half of new global HIV‐1 acquisition events. Tools for identifying adults at greatest risk of HIV‐1 can guide focused HIV prevention with pre‐exposure prophylaxis (PrEP). Although considerable attention has been devoted to which women are most likely to acquire HIV‐1, similar assessments are lacking in men. We sought to identify men at highest risk of HIV‐1 and estimate potential PrEP reach and efficiency.

**Methods: **From 2015 to 2019, nationally representative surveys were conducted in Cote d'Ivoire, Eswatini, Kenya, Lesotho, Malawi, Namibia, Nigeria, Rwanda, South Africa, Tanzania, Uganda, Zambia and Zimbabwe. Lasso regression models were fit with 27 individual, partner, household and epidemiologic variables to predict recent HIV‐1 diagnosis among men 15−59 years. Models were trained and internally cross‐validated. Performance was evaluated using area under the receiver‐operating‐characteristic curve (AUC), sensitivity, specificity and number needed to treat (NNT).

**Results: **Among 167,121 participants, 112 had recent HIV‐1, representing 122 million men and 256,000 new annual cases. Only two variables were retained: (1) living in a subnational area with high prevalence of HIV‐1 viraemia (the product of adult HIV‐1 prevalence and non‐suppression among adults living with HIV) and (2) having a male sexual partner. Full‐population AUC was moderately high (0.80); cross‐validated AUC was slightly lower (0.76). When targeting a sensitivity of 33%, 8.1 million men were predicted to have an elevated risk of HIV‐1 acquisition; NNT was 105. When sensitivity was 67%, 28.1 million men were predicted to have an elevated risk of HIV‐1 acquisition; NNT was 164.

**Conclusions: **This parsimonious risk assessment tool was generalizable to the region and had good performance. Our findings emphasize the importance of offering PrEP in areas of highest population HIV‐1 viraemia and to men who have sex with men, with trade‐offs between reach and efficiency.[Fig jia226351-fig-0013]

**Figure 1 jia226351-fig-0013:**
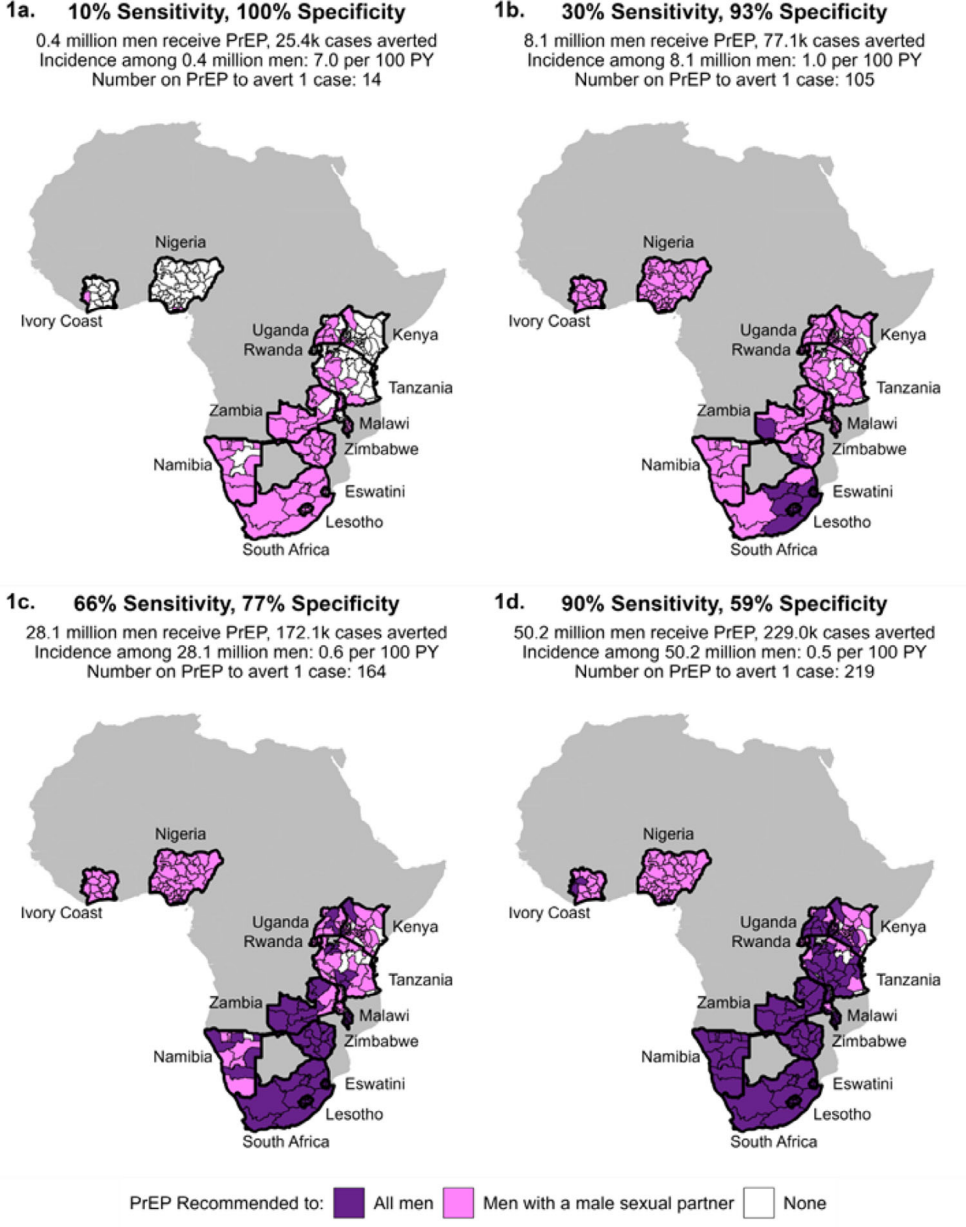
**OA1606**

### OA1702 Money, jobs or schooling? A model‐based evaluation of the potential health impact of economic strengthening in South Africa

L. Johnson ^1^, L. Jamieson^2^, M. Kubjane^2^, G. Meyer‐Rath^2^

^1^University of Cape Town, Centre for Infectious Disease Epidemiology and Research, Cape Town, South Africa, ^2^University of Witwatersrand, Health Economics and Epidemiology Research Office, Johannesburg, South Africa

**Background: **High incidence rates of HIV, sexually transmitted infections (STIs) and teenage pregnancy are major challenges facing South Africa. The role of socio‐economic drivers is complex, with high socio‐economic status protecting against some risk behaviours (condomless sex, early sexual debut and casual/transactional sex in females) but increasing others (e.g. male engagement in transactional/commercial sex). Consequently, the impact of economic strengthening interventions is unclear.

**Methods: **We extended a previously developed agent‐based model of HIV, STIs and fertility in South Africa, to reflect effects of education, employment and per‐capita household income on sexual behaviours. Effects were estimated from literature and from calibration of the model to African randomized controlled trials of economic strengthening interventions. Population attributable fractions (PAFs) were calculated. We considered three intervention types, targeting households with per‐capita income below the national average: school support to reduce dropout; vocational training for unemployed adults; and unconditional cash transfers.

**Results: **Low socio‐economic status accounted for 17% of new HIV infections, 12% of incident STIs (gonorrhoea, chlamydia and trichomoniasis) and 11% of teenage births in South Africa, over 2000−2020. However, because of uncertainties regarding effect sizes, confidence intervals around these PAFs were wide (−11 to 44%, −11 to 35% and 0 to 26%, respectively), with uncertainty in the effect of education on condom use being the most significant correlate of the HIV PAF (*r* = 0.95) and the effect of schooling on sexual debut being the most significant correlate of the teenage birth PAF (*r* = 0.79). Over 2025−2040, vocational training would achieve the greatest reduction in HIV incidence (2.2%, 95% CI: −4.1 to 8.5%), cash transfers would achieve the greatest reduction in STI incidence (1.8%, 95% CI: −2.5 to 6.2%) and school support would achieve the greatest reduction in teenage births (1.6%, 95% CI: 0.0−3.2%).

**Conclusions: **Structural interventions to reduce poverty could modestly improve several reproductive health outcomes in South Africa. However, ambiguity regarding causal pathways precludes a precise quantification of impacts, and there is a small risk of negative outcomes.[Table jia226351-tbl-0011]

**Table jia226351-tbl-0011:** OA1702

	New HIV infections			New STIs			Births to
	Total	Males	Females	Total	Males	Females	teenage girls
School support	0.5% (−2.8 to 3.9)	−0.4% (−4.4 to 3.6)	1.1% (−2.0 to 4.2)	1.7% (−0.8 to 4.1)	1.6% (−0.9 to 4.1)	1.8% (−0.5 to 4.0)	1.6% (0.0−3.2)
Vocational training	2.2% (−4.1to 8.5)	2.2% (−4.5 to 8.9)	2.2% (−4.0 to 8.2)	1.5% (−3.6 to 6.5)	1.5% (−3.8 to 6.8)	1.4% (−3.3 to 6.1)	0.4% (−5.3 to 6.1)
Cash transfers	1.1% (−4.4 to 6.7)	0.6% (−7.3 to 8.4)	1.5% (−3.0 to 6.0)	1.8% (−2.5 to 6.2)	1.8% (−2.7 to 6.4)	1.8% (−2.1 to 5.7)	0.7% (−2.3 to 3.7)

### OA1703 The role of identity worth, mistrust in science, PrEP stigma and PrEP acceptability as a predictor of PrEP self‐efficacy: A structural equation model

A. Gifford ^1^, R. Jaspal^2^, B. Jones^1^, D. McDermott^1^

^1^Nottingham Trent University, Psychology, Nottingham, United Kingdom, ^2^University of Brighton, Brighton, United Kingdom

**Background: **Pre‐exposure prophylaxis (PrEP) is a medication used to prevent the spread of human immunodeficiency virus (HIV). Populations with increased need of HIV prevention (e.g. men who have sex with men [MSM]) are eligible for PrEP for free in the UK. However, HIV surveillance reports indicate stagnated uptake of the drug, alongside increasing rates of HIV acquisition. As such, psychosocial research is needed to explore social barriers to PrEP uptake. This study aimed to explore the role of identity resilience as a predictor for PrEP usage. It was hypothesized that PrEP self‐efficacy would be positively predicted by PrEP acceptability. This would be mediated by mistrust in science, PrEP stigma and perceived risk of HIV.

**Methods: **Five hundred MSM participated in an online cross‐sectional, psychometric study between June and September in 2023. Participants had to be based in the UK but could either be PrEP or non‐PrEP users. Structural equation modelling (SEM) was used to explore a model of best fit to test the hypotheses.

**Results: **A significant model was found. Model Fit: χ^2^ = 1.43, df = 1, *p* = 0.231, CFI = 0.999, TLI = 0.981, RMSEA = 0.029.

**Conclusions: **This model indicates the indirect effects of identity worth on PrEP acceptability and PrEP self‐efficacy. Interestingly, identity continuity provides no predictive power among this sample of participants, nor does perceived risk of HIV (PRHS). This may indicate that the decision to take PrEP is primarily associated with the constructs of identity worth (i.e. self‐esteem, self‐efficacy and distinctiveness). Identity worth was positively associated with PrEP self‐efficacy. The association of identity worth with predicting PrEP acceptability is mediated through mistrust of science and PrEP stigma. When trying to develop interventions for PrEP uptake for those who feel efficacious at taking PrEP but are hesitant to take it, addressing specific parts of identity resilience may be beneficial.[Fig jia226351-fig-0014]

**Figure 1 jia226351-fig-0014:**
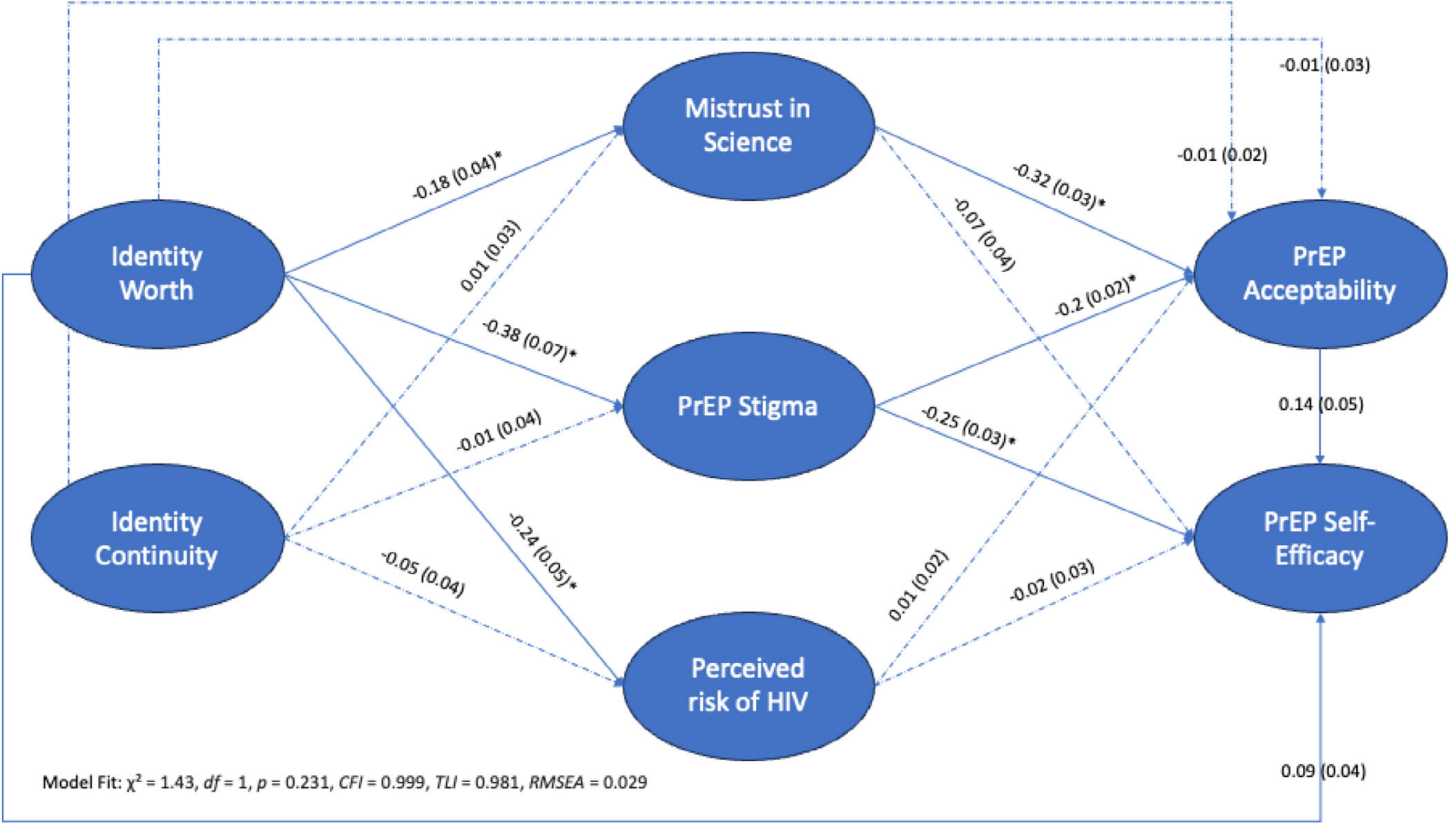
**OA1703**

### OA1704 Group by time outcome differences from baseline to month 3: findings from a couples‐based, HIV serostatus neutral digital intervention (P3) with 74 cisgender male couples in Lima, Peru

J. Mitchell ^1^, Z. Bursac^1^, D. Diaz^2^, E.M. Reyes Diaz^2^, A. Silva‐Santisteban^2^, K. Konda^3^

^1^Florida International University, Stempel College of Public Health and Social Work, Miami, United States, ^2^Universidad Peruana Cayetano Heredia, Lima, Peru, ^3^University of Southern California, Los Angeles, United States

**Background: **No efficacious, couples‐based HIV serostatus neutral interventions exist for cisgender male couples in Lima. Epidemiological evidence estimates a significant proportion of sexual minority men acquire HIV from their primary sexual partners. In response, we developed and are currently evaluating a theoretically guided, couples‐based, HIV serostatus neutral digital intervention to examine changes over time regarding communication, decision‐making and alignment of sexual agreements with using evidence‐based HIV strategies (e.g. routine testing, PrEP, ART/U = U) (i.e. preliminary efficacy).

**Methods: **We conducted a 6‐month, pilot randomized controlled trial with 74 cisgender, sexual minority male couples living in Lima. Assessments occurred every 3 months post baseline. A 3‐month waitlist control condition was used to compare outcome differences between the two groups of couples over time (i.e. immediate intervention vs. waitlist). We conducted generalized linear models (GLMs) to detect changes from baseline to month 3 between the two intervention conditions for individual‐level outcomes regarding communication, decision‐making and alignment of sexual agreements with using evidence‐based HIV strategies (e.g. routine testing, PrEP, ART/U = U).

**Results: **Output from the GLMs found significant improvements over time for the immediate intervention condition. Men's beliefs improved over time about whether their sexual agreement and its permitted behaviours matched their use of evidence‐based HIV strategies (b = 0.27, SE = 0.11, *p* = 0.02). The same group of participants also believed this type of matching had improved over time for their primary partners (b = 0.22, SE = 0.13, *p* = 0.11). Men communicating with their primary partners about having a detailed sexual agreement and using evidence‐based HIV strategies in their relationship also improved over time (b = 0.25, SE = 0.13, *p* = 0.05). Lastly, men making decisions with their partners about having a detailed sexual agreement and using evidence‐based HIV strategies in their relationship also improved over time (b = 0.30, SE = 0.14, *p* = 0.03). Similar types of improvements from baseline to month 3 were not observed among couples/partnered men in the waitlist intervention condition.

**Conclusions: **Compared to participants in the waitlist condition, our findings highlight important improvements among men in the immediate intervention condition. These findings signal that couples in the immediate intervention condition are communicating and making decisions about their sexual agreements and what evidence‐based HIV strategies to use in their relationship.

### OA1705 Food and housing insecurity among cisgender men who have sex with men in the United States and associations with social, behavioural and psychological HIV transmission vulnerabilities

D. Giovenco ^1^, R. Masa^2^, R. Moodley^3^, C. Acero^1^, I.L. Lucas^1^, M. Smith Jr^1^, M. Valentine‐Graves^1^, D. Operario^3^, T. Sanchez^1^

^1^Emory University, Epidemiology, Atlanta, United States, ^2^University of North Carolina at Chapel Hill, School of Social Work, Chapel Hill, United States, ^3^Emory University, Behavioral and Social Health Education Sciences, Atlanta, United States

**Background: **Socio‐economic disparities pose a critical challenge to preventing the transmission of HIV among key populations. This study investigates the prevalence of food and housing insecurity among cisgender men who have sex with men (MSM) in the United States and explores associations between these insecurities and social, behavioural, and psychological vulnerabilities related to HIV transmission.

**Methods: **We used cross‐sectional data from 2021 American Men's Internet Survey (AMIS), an annual survey among MSM in the United States. Participants self‐reported food and housing insecurity, substance use, condomless anal intercourse and transactional sex with male partners, STI diagnoses, and depression symptoms. Log‐binomial regression was used to determine if the prevalence of HIV transmission vulnerabilities was associated with food and/or housing insecurity, adjusting for confounding by age, race/ethnicity, income and HIV status.[Fig jia226351-fig-0015]

**Results: **Among 9061 MSM, the median age was 44 years (IQR = 32−57), the majority were non‐Hispanic white (65%) and had a college degree or higher education (62%), and 14% reported HIV‐positive status. Additionally, 14% (1213/8979) reported any food insecurity, 7.5% (674/9012) any housing insecurity, 3.8% (343/9022) both food and housing insecurity, and 83% (7425/8969) neither insecurity in the past 12 months. Figure 1 depicts associations between food and housing insecurity and HIV transmission vulnerabilities. Insecurities were significantly associated with a higher prevalence of depression symptoms (PHQ‐9 ≥10), as well as substance use, transactional sex and an STI diagnosis in the past 12 months. Food insecurity was associated with a lower prevalence of disordered alcohol use (AUDIT‐C ≥4).

**Figure 1 jia226351-fig-0015:**
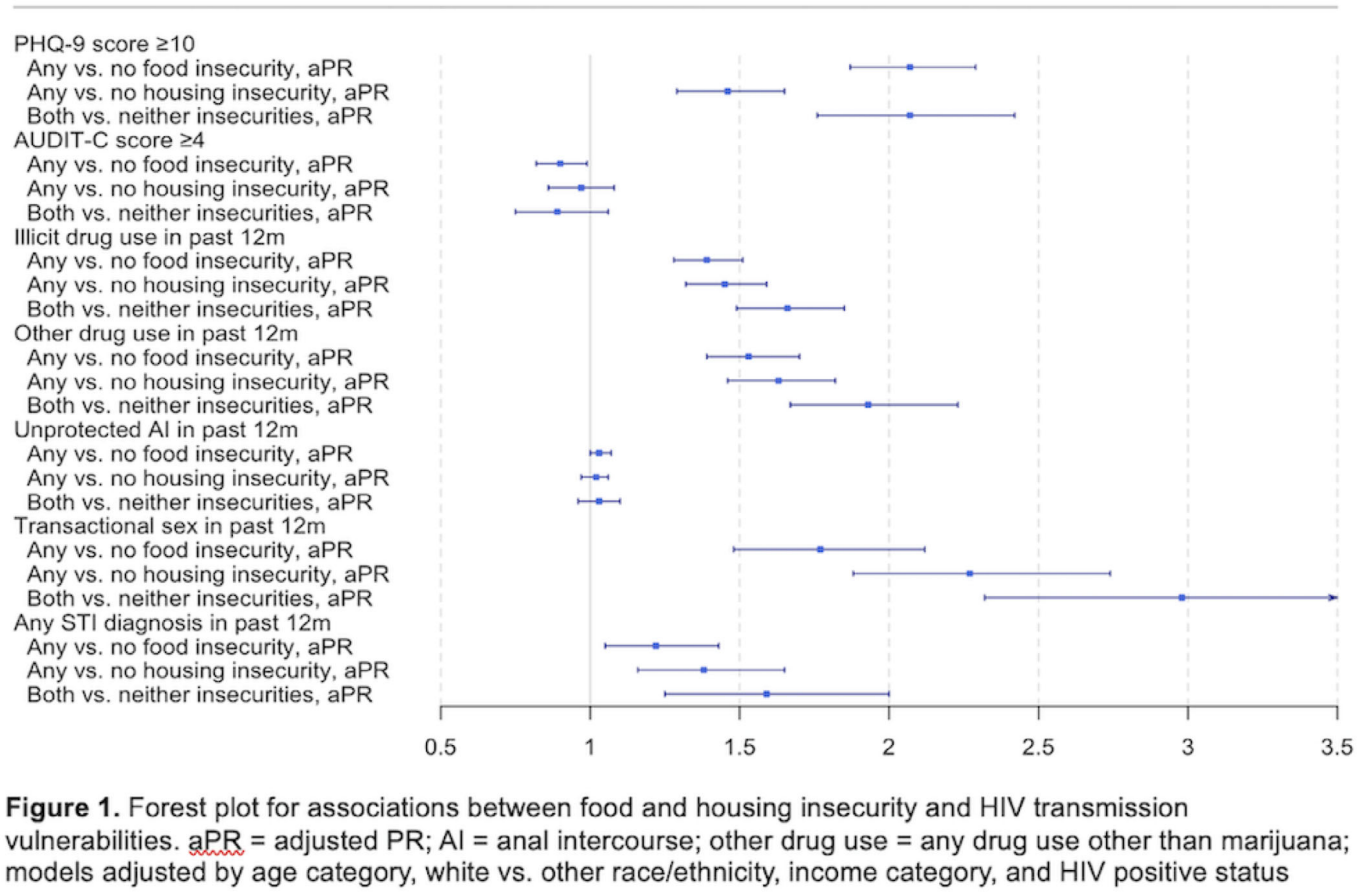
**OA1705**

**Conclusions: **Our study reveals that US MSM have concerning prevalence of food and housing insecurity that is significantly associated with increased vulnerabilities to sexual health and mental health. Addressing these insecurities is critical to mitigating HIV transmission risks and improving overall health outcomes in this population. Future interventions should prioritize addressing socio‐economic disparities to enhance HIV prevention efforts.

### OA1706 Factors associated with retention and adherence on pre‐exposure prophylaxis among men who have sex with men in Kigali, Rwanda: A cross‐sectional study

S. Mubezi ^1^, J. Uwineza^1^, G.N. Rwabasira^2^, J.D.D. Kayisinga^1^, S.S. Malamba^3^, E. Remera^2^, B. Ikuzo^2^, E. Ndengo^4^, N. Umuhoza^3^, B. Sangwayire^3^, R.C.N. Mwesigwa^3^, C.E. Stamatakis^3^, M.G. Wandera^1^, T.O. Olouch^3^, E. Kayirangwa^3^

^1^Society for Family Health (SFH) Rwanda, Health Program Unit, Kigali, Rwanda, ^2^Rwanda Biomedical Center (RBC), Ministry of Health, Kigali, Rwanda, ^3^Division of Global HIV and TB, Global Health Center (GHC), US Centers for Disease Control and Prevention (CDC), Kigali, Rwanda, ^4^United States Agency for International Development, Kigali, Rwanda

**Background: **Pre‐exposure prophylaxis (PrEP) is recommended as an HIV prevention measure for men who have sex with men (MSM). We assessed factors associated with PrEP retention and adherence among MSM in Kigali, Rwanda.

**Methods: **We undertook a cross‐sectional study and used a questionnaire to obtain PrEP retention and adherence history from MSM enrolled who attended follow‐up visits from four health facilities between April 2021 and June 2021. We used multivariable Cox regression to determine factors associated with 3‐month retention and principal component analysis (PCA) to determine factors associated with self‐reported adherence. Data were analysed using STATA (version 16.0).

**Results: **We interviewed 439 MSM aged 18 years and above who were initiated on PrEP. Majority were employed (57%, *n* = 251), between ages 25 and 34 years (49%, *n* = 217), close to half completed primary level education (47%, *n* = 206), were involved in sex work (42%, *n* = 184) and over a half lived in household of 1−2 members (55%, *n* = 241). Ninety percent of the MSM respondents (*n* = 393) were retained on PrEP at 3 months and among those retained, 287 (73%) had good adherence. Multivariable Cox regression revealed that MSM more likely to be retained on PrEP, were those who are sex workers (adjusted hazard ratio [aHR] = 4.139; 95% confidence interval [95% CI]: 1.569, 10.921), had more than one regular sexual partners (aHR = 3.949; 95% CI: 2.221, 7.022), lived in households of 3−5 members (aHR = 3.755; 95% CI: 1.706, 8.261), completed secondary school education (aHR = 2.154; 95% CI: 1.130, 4.108) and were circumcised (aHR = 2.218, 95% CI: 1.232, 3.993). Employed MSM had a 66% decreased likelihood to be retained on PrEP (aHR = 0.345; 95% CI: 0.168, 0.707). Similarly, MSM who used condoms consistently had an 85% decreased likelihood to be retained on PrEP (aHR = 0.149; 95% CI: 0.035, 0.632). Principal component regression analysis showed that the component with MSM with higher numbers of regular sexual partners had increased odds of adhering to PrEP (crude odds ratio [cOR] = 1.32; 95% CI: 1.144, 1.530).

**Conclusions: **Our study highlighted that MSM using PrEP as the main method of HIV prevention were more likely to be retained and adherent to PrEP. There is a need to emphasize PrEP use alongside other HIV prevention methods like condoms.

### OA1802 Short‐term combination immunotherapy with broadly neutralizing antibodies and CCR5 blockade mediates ART‐free viral control in infant rhesus macaques

N. Haigwood^1^, T. Ordonez^1^, S. Pandey^1^, J. Reed^1^, A. Hessell^1^, K. Van Rompay^2^, J. Watanabe^2^, J. Usachenko^2^, J. Sacha ^1^

^1^Oregon Health & Science University, Division of Pathobiology and Immunology, Portland, United States, ^2^University of California, Davis, California National Primate Research Center, Davis, United States

**Background: **In 2022, there were 1.5 million children living with HIV (CLWH), only half of whom had access to antiretroviral therapy (ART). Even when ART is available, lifelong daily adherence can be challenging for CLWH, emphasizing the need for alternative strategies to durably suppress HIV replication. Here, we evaluated whether a triple combination of early ART initiation with HIV broadly neutralizing antibodies (bNAbs) and the CCR5‐blocking mAb Leronlimab could mediate virus clearance in simian‐HIV (SHIV)‐infected infant rhesus macaques.

**Methods: **A total of 16 four‐week‐old infant rhesus macaques were orally infected with SHIV‐SF162P3 and placed into the following treatment groups, with all treatments beginning at 72 hours after infection: (1) ART + bolus doses of the bNAbs PGT121‐LS and VRC07‐523‐LS (*n* = 2), (2) ART + Leronlimab for 8 weeks (*n* = 6) or (3) ART + bolus doses of the bNAbs PGT121‐LS and VRC07‐523‐LS + Leronlimab for 8 weeks (*n* = 8). ART was maintained for 27 weeks, at which time an analytical treatment interruption (ATI) was performed and the kinetics of virus rebound compared between the groups. Untreated infected infants (*n* = 8) served as additional controls.

**Results: **Following ATI, both animals in group 1 (ART + bNAbs) rebounded rapidly, while 4/6 in group 2 (ART + Leronlimab) rebounded with viremia by week 10 post‐ATI, with two remaining aviremic currently through 20‐week post‐ATI. In contrast, 0/8 animals in group 3 (ART + bNAbs + Leronlimab) have rebounded at the time of abstract submission (15 weeks post ATI). Assessment of the viral reservoir is ongoing.

**Conclusions: **Finding a treatment that can prevent reservoir establishment and spread after the first 48 hours has been elusive. The results of this study suggest that the combination of ART, bNAbs and CCR5 blockade via Leronlimab synergize in an undefined mechanism to prevent further seeding of the reservoir early after infection, and may even permanently reduce it. Further studies are warranted to study this combinatorial effect in more detail.

### OA1803 Induction of precursor CD4 binding‐site targeting broadly neutralizing antibodies in infant macaques following immunization with germline‐targeting SOSIP trimers

A. Nelson ^1^, X. Shen^2^, S. Vekatayogi^2^, S. Zhang^3^, G. Ozorowski^3^, M. Dennis^1^, L.M. Sewall^3^, Y. Chen^2^, J. Eudailey^1^, J. Isaac^1^, W.B. Williams^2^, A.B. Ward^3^, D.C. Montefiori^2^, K.K. Van Rompay^4^, K. Wiehe^2^, J.P. Moore^5^, R.W. Sanders^5,6^, K. De Paris^7^, S. Permar^1^

^1^Weill Cornell Medicine, Pediatrics, New York, United States, ^2^Duke University Medical Center, Human Vaccine Institute, Durham, United States, ^3^The Scripps Research Institute, Integrative Structural and Computational Biology, La Jolla, United States, ^4^University of California Davis, California National Primate Research Center, Davis, United States, ^5^Weill Cornell Medicine, Department of Microbiology and Immunology, New York, United States, ^6^Academic Medical Center, Department of Medical Microbiology, Amsterdam, the Netherlands, ^7^University of North Carolina at Chapel Hill, Department of Microbiology and Immunology, Chapel Hill, United States

**Background: **A vaccine that can establish protective immunity before sexual debut is crucial to prevent the estimated 410,000 new cases of HIV that occur annually among adolescents. The time between weaning and sexual debut is a period of relatively low risk for HIV acquisition, thus early childhood is an opportune window for implementation of a multi‐dose HIV immunization strategy to elicit protective immunity prior to adolescence. As the elicitation of bnAbs will be critical for an effective HIV vaccine, the goal of our study was to assess the ability of a B‐cell lineage‐designed HIV envelope SOSIP to induce precursor CD4bs‐targeting bnAbs in early life.

**Methods: **Infant rhesus macaques (RMs) received either the wild‐type (WT) BG505 SOSIP or the CD4bs germline‐targeting BG505 GT1.1 SOSIP (*n* = 5/group) with the 3M‐052‐SE adjuvant at 0, 6 and 12 weeks of age. All infant RMs were then boosted with the WT BG505 SOSIP at weeks 26, 52 and 78, mimicking a paediatric immunization schedule of multiple vaccine boosts within the first 2 years of life.

**Results: **Both immunization strategies induced durable, high magnitude binding antibodies and plasma autologous virus neutralization. Most notably, three GT1.1‐primed infants exhibited a plasma HIV neutralization signature reflective of VRC01‐like CD4bs bnAb precursor development. Negative stain electron microscopy‐based polyclonal epitope mapping (nsEMPEM) of IgG in plasma demonstrated that only GT1.1‐primed infants developed plasma antibodies targeting the CD4bs. High‐resolution CryoEMPEM mapping of polyclonal antibodies in one GT.1‐primed infant with the bnAb precursor signature revealed N276 glycan accommodation of CD4bs‐directed responses. In characterizing the GT1.1 vaccine‐elicited B‐cell repertoire in the three infants exhibiting a CD4bs bnAb precursor response, we isolated one autologous virus neutralizing mAb with epitope specificity for the CD4bs and moderate heterologous virus neutralization activity.

**Conclusions: **Thus, a multi‐dose immunization regimen with bnAb lineage designed SOSIPs is a promising strategy for implementation in early childhood, and the induction of early B‐cell responses with the potential to mature into protective HIV bnAbs prior to adolescence when sexual HIV exposure risk begins.

### OA1804 Induction of V3 glycan‐directed responses post CH848 gp160 mRNA‐LNP vaccination in infant rhesus macaques

D. Davis ^1^, M.‐G. Alameh^2^, E. Lee^3^, A. Nelson^4^, X. Shen^5^, D. Montefiori^5^, K. Wiehe^3^, J. Pollara^5^, K.K.A. Van Rompay^6^, K. Saunders^3^, D. Weissman^2^, B. Haynes^3^, S. Permar^4^, K. De Paris^1^

^1^The University of North Carolina at Chapel Hill, Dept. of Microbiology and Immunology, Chapel Hill, United States, ^2^University of Pennsylvania, Dept. of Medicine, Philadelphia, United States, ^3^Duke University Medical Center, Duke Human Vaccine Institute, Durham, United States, ^4^Weill Cornell Medical College, Dept. of Pediatrics, New York, United States, ^5^Duke University School of Medicine, Dept. of Surgery, Durham, United States, ^6^University of California Davis, California National Primate Research Center, Davis, United States

**Background: **A major goal in a successful HIV vaccine is the induction of a broadly neutralizing antibody (bNAb) response. BNabs need years to develop in adults, whereas infants living with HIV induce bNAbs early and at higher frequency. We hypothesize that a multi‐dose HIV vaccine started during infancy would allow for the necessary time to mature B cells towards the production of bNAbs and could be easily implemented by incorporation into the global paediatric vaccine programme.

**Methods: **Six infant rhesus macaques (RMs) were intradermally immunized at weeks 12, 24 and 52 with the CH848 10.17DT gp160 mRNA‐LNP priming immunogen (50 µg), which lacks the N133 and N138T V1 glycans and can engage the unmutated common ancestor (UCA) of the DH270 V3 glycan bNAb lineage and boosted with 50 µg CH848 10.17 wild‐type (WT) gp160 mRNA‐LNP, with the V1‐glycans restored (weeks 86, 90 and 96). Tier 2 neutralizing antibodies and SOSIP trimer binding were measured and compared between the DT prime and WT boost vaccine regimen.

**Results: **Two weeks post third DT priming immunization, all infants developed tier 2 autologous neutralizing ID_50_ titres against the DT vaccine‐matched pseudovirus, with a geometric mean ID50 titre (GMT) of (78,422 ± 6.41). Two infants showed a three‐fold decrease to the N332 glycan knockout pseudovirus in sera, indicating a potentially V3 glycan‐directed response. CH848 V3‐specific B cells were also detectable by flow cytometry. Two weeks post third WT boost, titres were maintained against the DT vaccine‐matched pseudovirus (GMT ID50 44,237 ± 2.76), but the V3 glycan‐directed neutralizing responses were no longer detectable in the sera. Nonetheless, four of six infants showed plasma antibody cross‐reactivity to other Env trimers, especially to 92RW020, at 6 months post WT boost, although antibody binding was not dependent on the N332 glycan.

**Conclusions: **We plan to boost with heterologous 92RW020 mRNA‐LNP to promote further antibody maturation and breadth. Our studies demonstrate that mRNA‐LNP vaccines can induce V3 glycan‐directed responses in infant RMs, but further optimization, specifically with the shaping boost immunogen, is needed to induce precursors to bNAbs.

### OA1805 Immunogenicity of HIV BG505 germline‐targeting GT1.1 SOSIP envelope trimer immunization in infant and juvenile rhesus macaques

Y. Issah ^1^, A. Nelson^1^, X. Hu^2^, J. Isaac^1^, X. Shen^3^, G. Ozorowski^4^, L.M. Sewall^4^, S. Zhang^4^, A.B. Ward^4^, D.C. Montefiori^3,5^, R.W. Sanders^6,7^, J.P. Moore^6^, K.K. Rompay^8^, K. De Paris^9,10^, S.R. Permar^1^

^1^Weill Cornell Medicine, Department of Pediatrics, New York, United States, ^2^Yasmine Issah, Department of Pediatrics, New York, United States, ^3^Duke University Medical Center, Human Vaccine Institute, Durham, United States, ^4^The Scripps Research Institute, San Diego, United States, ^5^Duke University School of Medicine, Department of Surgery, Durham, United States, ^6^Weill Cornell Medicine, Division of Microbiology and Immunology, New York, United States, ^7^Academic Medical Center, Department of Medical Microbiology, Amsterdam, the Netherlands, ^8^University of California, California National Primate Research Center, Davis, United States, ^9^University of North Carolina at Chapel Hill, Department of Microbiology and Immunology, Chapel Hill, United States, ^10^University of North Carolina at Chapel Hill, Center for AIDS Research, Chapel Hill, United States

**Background: **HIV is a highly mutable virus, therefore, a vaccine that induces protective, broadly neutralizing antibodies (bnAbs) before sexual debut is critical to eliminate the ∼410,000 new cases annually among adolescents worldwide. Recent work has established that children living with HIV develop bnAbs earlier and at a higher frequency than adults. In this study, we compared the ability of a CD4‐binding site (CD4bs) germline‐targeting SOSIP trimer immunization strategy to induce precursor bnAbs in infant and juvenile rhesus macaques (RMs).

**Methods: **Infant (*n* = 5) and juvenile (*n* = 4) RMs received three immunizations of the germline‐targeting BG505 GT1.1 SOSIP trimer (50 µg) with the 3M‐052‐SE adjuvant 6 weeks apart. All RMs were then boosted 12 weeks later with the BG505.664 WT SOSIP trimer three times in 6‐month intervals. After an over 1‐year follow‐up, all animals received two boosts with a mixed Clade B SOSIP nanoparticle. Vaccine‐elicited antibody responses were monitored through 2.5 years after the first vaccination.

**Results: **BG505 GT1.1 SOSIP trimer immunization consistently induced higher magnitude vaccine‐specific IgG binding and tier 1 neutralization responses in infants compared to juvenile RMs. Plasma tier 2 autologous virus neutralization responses were similar between the groups, yet nsEMPEM demonstrated that the infant response generally targeted more epitopes. Notably, after the fifth immunization, three of five GT1.1 SOSIP‐immunized infants exhibited a plasma neutralization signature indicating CD4bs bnAb precursor development, compared to only one of four juvenile RMs. Additionally, at this time, two infants and one juvenile exhibited low‐level heterologous tier 2 virus neutralization activity. By week 150, 2 weeks post‐second nanoparticle boost, an additional infant developed the CD4bs precursor bnAb signature, whereas this response was not maintained in the one juvenile. Nanoparticle boosting also improved the breadth of heterologous neutralization in four out of the five infants and two out of the four juveniles.

**Conclusions: **Our data indicate that sequential immunization with germline‐targeting BG505 SOSIP trimers may induce neutralizing antibodies and CD4bs bnAb precursors more frequently in infant compared to juvenile. Our results highlight the potential for an HIV immunization strategy in early life to induce protective bnAb responses and can inform approaches for future human paediatric clinical trials.

### OA1806 Anti‐SIV Env RhmAbs +/− CD8a depletion and N‐803 in ART‐suppressed rhesus macaques leads to post‐treatment control of viraemia associated with transcriptomic changes in CD8^+^ and CD4^+^ T cells

V. Singh^1^, D. Burgess^1^, A. Dashti^1^, H. King^2^, E. Fray^3^, M. Mavigner^1^, R. Mason^2^, J. Safrit^4^, J. Lifson^5^, R. Siliciano^3^, J. Siliciano^3^, M. Roederer^2^, A. Sharma^1^, G. Silvestri^1^, A. Chahroudi ^1^

^1^Emory School of Medicine, Atlanta, United States, ^2^Vaccine Research Center, Bethesda, United States, ^3^Johns Hopkins School of Medicine, Baltimore, United States, ^4^ImmunityBio, Culver City, United States, ^5^Frederick National Laboratory for Cancer Research, Frederick, United States

**Background: **Building upon robust latency reversal seen after CD8a‐depletion and IL‐15 superagonist treatment in SIV‐infected, ART‐suppressed rhesus macaques (RMs), here we combined these agents with four rhesus‐derived anti‐SIV Env‐specific rhesus IgG1 monoclonal antibodies (RhmAbs) with the goal to reduce reservoirs and/or modulate viral rebound dynamics after ART interruption.

**Methods**: Twenty‐eight RMs were infected with SIV_mac239_; ART was initiated 8 weeks post‐infection. Groups were assigned after 96 weeks on‐ART: Group 1 (*n* = 7): ART‐only; Group 2 (*n* = 7): ART+RhmAbs; Group 3 (*n* = 14): ART+RhmAbs+CD8a‐depletion+N‐803. Analytical treatment interruption (ATI) of ART was performed when SIV RhmAb were no longer detected (for 3/4 RhmAbs) or well below the IC90 (for 1/4 RhmAbs).

**Results: **Latency reversal (defined as on‐ART viraemia >60 copies/ml) was achieved in 11/14 Group 3 RMs versus 0/14 Group 1+2 RMs. Median CD4^+^ T cell‐associated SIV‐DNA and intact provirus levels were lower post‐ versus pre‐intervention in all Groups, with the greatest change seen in Group 3 RMs (*p*<0.0001 for both reservoir measures in blood; *p* = 0.0003 and *p*<0.0001 for total and intact SIV‐DNA in lymph nodes, respectively). By ATI day 21, all RMs rebounded to >60 copies/ml with no intergroup difference in time‐to‐rebound. A greater fold reduction in viral setpoint 3 months post‐ATI compared to pre‐ART was seen in Group 2 (*p* = 0.018) and Group 3 (*p*<0.008), but not Group 1. Post‐treatment viral control off‐ART (PTC, defined as ≥3 consecutive viral loads <10^3^ copies/ml) was observed in eight RMs from Groups 2 and 3 and none from Group 1. Transcriptomic analyses revealed a post‐intervention increase in immune activation and metabolism pathways in CD8^+^ T cells from RMs with PTC. CD4^+^ T cells from RMs with PTC had increased interferon pathway and decreased TGFbeta signalling genes post‐intervention. Similar changes were not found in RMs with typical rebound viraemia.

**Conclusions: **Time‐to‐viral‐rebound was not impacted by SIV RhmAbs+CD8a‐depletion+N‐803 despite robust latency reversal and evidence of reduced infected cell levels. PTC was observed only in animals receiving SIV RhmAbs and SIV RhmAbs+CD8a‐depletion+N‐803, likely explained by treatment‐induced changes in the T‐cell transcriptome towards an activated and antiviral state.

### OA1902 Mission (NOT) impossible: digital intelligence‐guided care cascade management to eliminate mother to child transmission of HIV. Results from Ahana project supported by The Global Fund 13 states of India

K. Biswas ^1^, M.K. Singh^1^, S. Dasgupta^1^, R. Rana^1^, A. Muhammad^1^, M. Jain^1^, A. Satsangi^1^, J. Holker^1^

^1^Plan India, Global Fund, New Delhi, India

**Background: **Evidence suggests that access to HIV testing remained low at 36% in the 13 Plan India project states during 2016−17 with an estimated annual pregnancy of 14 million (48% of total in the country) per year. Complementing Govt. of India's EMTCT of HIV strategy, Plan India has been implementing Ahana project supported by The Global Fund towards attaining Elimination of Mother to Child Transmission in 13 priority states of India.

**Methods: **Care cascade monitoring and management has been guided through a digitally operated intelligent approach: (1) a digital MIS application has been developed, (2) all 330 field workers have been provided with tablets to (a) manage and monitor early identification and linkages to ART treatment, (b) follow‐up of PPWs with care and support services towards drug adherence through digitally guided intelligence, (c) due management and proofing of leakages to ensure viral suppression.

**Results: **With expansion in the PMTCT service coverage, HIV testing among pregnant women increased from 36% during 2016−17 to 89% during 2022−23 resulted in increasing identification of HIV‐positive pregnant women. More than 25,000 pregnant women identified as HIV positive during April 16 to Sept 23 were linked to ART. Linkage to ART services improved from 86% during 2016−17 to 99.7% during 22−23. Institutional deliveries among PPW increased from 90% during 2016−17 to more than 94% in April−Dec 23. While linkages of HIV‐exposed infants with EID services within 60 days improved from 55% during 2016−17 to 90% in 22−23, HIV testing among spouses of PPWs increased from 74% during 2018−19 to 97% during 22−23. More than 87% babies followed up till 18 months and 13,000 babies confirmed as negative at confirmatory testing.

**Conclusions: **The result suggests that digitally guided outreach has enabled to early linkages to treatment, follow up for adherence and management of EID algorithm. Intelligence‐guided prioritization enabled successful due management and improved cascade. Plan India's Ahana project shows the application of digital platform and intelligence‐guided cascade management as an effective tool in the journey to achieve EMTCT.

### OA1903 Findings from the Todurujo na Kadurok (Empowering Youth) HIV self‐testing and edutainment comic randomized controlled trial with refugee youth in a humanitarian setting in Uganda

C. Logie ^1^, M. Okumu^2^, M. Loutet^3^, S. Odong Lukone^4^, N. Kisubi^4^, P. Kyambadde^5^, L. Mbuagbaw^6^

^1^University of Toronto, Factor‐Inwentash Faculty of Social Work, Toronto, Canada, ^2^University of Illinois Urbana‐Champaign, Urbana, United States, ^3^University of Toronto, Dalla Lana School of Public Health, Toronto, Canada, ^4^Uganda Refugee and Disaster Management Council, Yumbe, Uganda, ^5^Uganda Ministry of Health, Kampala, Uganda, ^6^McMaster University, Hamilton, Canada

**Background: **HIV vulnerabilities among refugee youth are shaped by structural and social factors in humanitarian settings that constrain access to HIV prevention and testing. Although HIV self‐testing (HIV‐ST) is particularly promising among youth, humanitarian contexts are underserved by HIV‐ST. Comic books, a form of graphic medicine whereby images are juxtaposed by text reflecting internal and external narratives to share health information (known as “edutainment”), are understudied in the context of HIV‐ST. Uganda is African's largest refugee hosting nation with over 1.5 million refugees. We evaluated the effectiveness of HIVST and edutainment comics in increasing HIV testing with refugee youth in Bidi Bidi refugee settlement, Uganda.

**Methods: **We conducted a qualitative formative phase with focus groups with refugee youth to create an edutainment comic about HIV testing barriers and facilitators in Bidi Bidi. We then conducted a randomized controlled trial in Bidi Bidi with a purposive sample of refugee youth aged 16−24. Arms included: (1) HIV‐ST; (2) comics; (3) HIV‐ST with comics; and (4) standard of care (SOC). Intervention effects on primary (HIV testing uptake) and secondary (e.g. HIV knowledge) outcomes at 3‐month follow‐up (T2) were assessed using generalized estimating equation (GEE) models.

**Results: **There was 98% retention (*n* = 117/120) of participants (*n* = 120; mean age: 20, standard deviation: 2.3) at T2. In adjusted analyses, in comparison with the SOC at T2, HIV testing odds were highest in Arm 3 (adjusted odds ratio [aOR]: 8.46; 95% confidence interval [CI]: 2.87−24.97) followed by Arm 2 (aOR: 4.14; 95% CI: 1.58−10.87), with no significant differences with Arm 1 (aOR: 2.81; 95% CI: 0.96−8.16). Arm 1 at T2 reported lower HIV‐related stigma (ab: −0.95, 95% CI: −1.9, −0.03), reduced condom use at last sex (aOR: 0.21, 95% CI: 0.07−0.65) and lower consistent condom use compared to the SOC (aOR: 0.010, 95% CI: 0.02−0.58). In secondary analyses including all participants, there were statistically significant T1 to T2 increases in HIV testing (aOR: 21.79; 95% CI: 4.57−103.93), HIV knowledge (ab: 1.45; 95% CI: 0.93−1.97; *p*<0.001) and safer sex efficacy (ab: 3.64; 95% CI: 2.09−5.19; *p*<0.001).

**Conclusions: **HIV self‐testing is feasible with youth in a Ugandan refugee settlement and can be supplemented with edutainment comics to advance HIV prevention.

### OA1904 Use of WhatsApp chatbot technology to support effective use of HIV self‐testing among men and young adults in the private sector in Kenya

H. Ayallo ^1^, I. Nzuki^2^, C. Pahe^2^

^1^Population Services Kenya, Kisumu, Kenya, ^2^Population Services Kenya, Nairobi, Kenya

**Background: **The evolution of social media has impacted health programmes in unique ways. A lot of people have access to and use various social media differently. WhatsApp has 2 billion users making it the most popular app in over 100 countries including Kenya. This paper seeks to illustrate the role of a digital counsellor (WhatsApp chatbot) in promoting the effective use of HIV self‐testing (HIVST) kits in Kenya.

**Methods: **PS Kenya developed a WhatsApp chatbot to promote access to HIVST information and products. The chatbot content was reviewed by the target audience to make it desirable. One hundred and twenty pharmacists were trained to support HIVST users in land on chatbot and access information. We conducted pharmacy activations where trained sales agents engaged HIVST users on the chatbot. Clients landing on the chatbot were assisted to register and access the main menu with information on where to find HIVST kits, how to test, HIVST self‐reporting and risk assessments. The client's data were captured electronically as they navigated the chatbot and analysed.

**Results: **Out of 722 clients who landed on the WhatsApp chatbot, 384 (53.2%) were male, 283 (39.2%) were females and 55 (7.6%) did not reveal their identity in the 10‐month time. 83.5% (603) of the chatbot users were aged 16−30 years, while 16.5% of the users were people above 30 years. One hundred and seventy of the users reported having never tested for HIV, while 230 mentioned having tested more than 12 months ago with the rest of the users tested within the last 12 months. Of the total users who landed on the chatbot, 386 (53.5%) wanted to find HIVST kits, while 344 (47.6%) reported to have performed HIVST screening. On the self‐reporting menu, 697 (96.5%) users reported to have not tested before using the chatbot, while 73 (10.1%) reported to have accessed HIVST kits before. On whether the test results were reactive, 36 users reported positive results, while 17 users reported to have conducted confirmatory testing at a facility indicated on the chatbot.

**Conclusions: **WhatsApp chatbot is a viable solution in promoting the effective use of HIVST kits among young people hence sustaining HIV prevention programmes globally.

### OA1905 Home‐based STI self‐testing and diagnosis to trigger PrEP restart among adolescent girls and young women in South Africa

K. Reddy ^1^, J. Hao^2^, N. Sigcu^1^, N. Dladla^1^, M. Govindasami^1^, N. Matswake^1^, B. Jiane^1^, L. Bopape^1^, H. Ishmail^1^, R. Kgoa^1^, L. Kew^1^, N. Ndlovu^1^, H. Mposula^1^, R. Stuurman^1^, R. Heffron^2^, T. Palanee‐Phillips^1,3^

^1^Wits RHI, University of the Witwatersrand, Faculty of Health Sciences, Johannesburg, South Africa, ^2^The University of Alabama at Birmingham Heersink School of Medicine, Center for AIDS Research, Birmingham, United States, ^3^University of Washington, Department of Epidemiology, School of Public Health, Seattle, United States

**Background: **Intermittent adherence and poor persistence on oral pre‐exposure prophylaxis (PrEP) for HIV prevention are common among adolescent girls and young women (AGYW). Discrete mechanisms for AGYW to accurately self‐identify periods of heightened risk and trigger PrEP initiation are lacking. We report on preliminary effectiveness of home‐based STI self‐testing and a self‐administered behavioural risk tool to trigger PrEP restart.

**Methods: **PALESA, a pilot randomized controlled trial, was launched in June 2023 at a clinical research site in Johannesburg, South Africa and is due for completion in June 2024. HIV‐negative, non‐pregnant, sexually active, cisgender AGYW, aged 16−18 years who previously used PrEP with discontinuation within the last 6 months, were enrolled and randomized to one of two study arms: (1) STI self‐testing (using the Visby Medical Sexual Health Test) coupled with a self‐administered behavioural risk tool and (2) self‐administered behavioural risk tool only. Participants were followed for 6 months through a combination of virtual (Months 1, 2, 4 and 5) and in‐person visits (Months 3 and 6) to collect data on sexual behaviour and experiences with the STI self‐testing kits, and self‐administered behavioural risk assessment. We used a negative binomial model to compare PrEP restart between study arms at 3 months.

**Results: **Of 55 AGYW enrolled in PALESA (median age = 18; IQR 17−18), 25.5% reported never using condoms with their current partner and 45.5% reported inconsistent condom use. During the first 3 months of follow up, *Chlamydia trachomatis* was diagnosed in 39.3% in the self‐testing arm and 33.3% in the comparator arm and *Neisseria gonorrhoea* was diagnosed among 10.7% in the self‐testing arm and 11.1% in the comparator arm. By 3 months of study follow‐up, 71.4% of participants in the STI self‐testing arm and 44.4% in the comparator arm restarted oral PrEP use (adjusted RR 2.27, 95% CI 0.98−5.24).

**Conclusions: **In this pilot study to collect preliminary data on novel tools to trigger PrEP re‐start among AGYW, STI self‐testing may have a higher impact on PrEP restart. Further research with a larger cohort is warranted to confirm these findings and further explore the potential for STI self‐testing to impact PrEP use in AGYW.

### OA1906 Scaling up HIV self‐testing through different distribution channels in Myanmar

L. Htet^1^, T. Soe ^1^, K.P. Naing^1^, L.A. Thu^1^, M.K. Kyaw^1^, T.T. Win^1^, Y.M. Thaung^1^, S.L. Tip^2^, M. Mishra^2^, T.Y. Pyone^1^, P.P. Phyo^1^

^1^Community Partners International, HIV/TB Agency Information and Services Activity, Yangon, Myanmar, ^2^United States Agency for International Development, Yangon, Myanmar

**Background: **Myanmar is one of 35 countries accounting for 90% of global new HIV acquisitions with concentrated epidemic among key populations (KPs). HIV testing coverage was 31% among men having sex with men (MSM), 41% among female sex workers (FSWs), and 28% among people who inject drugs (PWID). Access to HIV testing is impeded by ongoing political unrest, limited public health sector, movement restrictions and armed conflicts. The study was done to demonstrate the feasibility of HIV self‐testing (HIVST) distribution through different channels using client‐centred approaches.

**Methods: **From May 2022 to Dec 2023, the programme distributed HIVST in three states and regions through the multi‐level strategy that included five KP‐led CSOs at the community level, three implementing partners at the facility level and online through the Facebook chatbot linked with 16 private pharmacies. A descriptive analysis of the distribution data was done.

**Results: **Four thousand two hundred and sixty‐one HIVSTs were distributed through three channels. Ninety‐eight percent (*n* = 4174) of HIVSTs were received by KPs including 2727 MSM, 178 TGW, 823 SW and 446 PWID. The result return rate was 97%(*n* = 4137): 100% (*n* = 2687) with the facility, 98% (*n* = 1156) with the community and 76% (*n* = 294) with online. The reactive rate was 4% (*n* = 156): 3% (*n* = 81) with facility, 7% (*n* = 69) with community and 2% (*n* = 6) with online. One hundred and fifty‐six HIVSTs were reactive, 86 of them received confirmation testing and received ART, and 172 received PrEP. Three hundred and thirty KPs used HIVST for index testing with a reactive rate of 5% (*n* = 15) and 1385 KPs were used for PrEP follow‐up. Two thousand eight hundred and seventy‐six case‐finding HIVSTs (excluding PrEP follow‐up) distributed: 49% (*n* = 1415) with facility, 38% (*n* = 1080) with community and 13% (*n* = 381) with online. Reactive rate of case‐finding HIVST was 5% (*n* = 141): 6% (*n* = 78) with facility, 6% (*n* = 57) with community and 2% (*n* = 6) with online. One thousand eight hundred and seventy‐one (47%) first‐time testers and 1005 (53%) subsequent testers received case‐finding HIVST with reactive rates of 6% (*n* = 106) and 3% (*n* = 35).

**Conclusions: **HIVST can be scaled up in Myanmar through three distribution channels: facility, community and online. This approach can help the country achieve the first 95 targets by reaching more KPs with HIV testing services. Access to HIVSTs can also increase the demand for PrEP and other HIV services among KPs.

### OA2002 PrEP uptake and adherence among transgender women: findings from a randomized clinical trial of a multicomponent intervention (HPTN 091)

T. Poteat ^1^, G. Beauchamp^2^, M.A. Marzinke^3^, K. Gomez‐Feliciano^4^, B. Akingbade^4^, J. Beck^5^, I. Bell^2^, V. Cummings^3^, L. Emel^2^, J. Franks^6^, E.M. Jalil^7^, J.E. Lake^8^, A. Liu^9^, J. Lucas^4^, K.H. Mayer^10^, A.E. Radix^11^, J. Rooney^12^, H. Spiegel^13^, D.L. Watson^14^, S. Zangeneh^15^, S.L. Reisner^16^, HPTN 091 Study Team

^1^Duke University, School of Nursing, Durham, United States, ^2^Fred Hutchinson Cancer Center, Seattle, United States, ^3^Johns Hopkins University School of Medicine, Baltimore, United States, ^4^FHI360, Durham, United States, ^5^National Institute of Allergy and Infectious Diseases (NIAID), Division of AIDS, Rockville, United States, ^6^ICAP at Columbia University Mailman School of Public Health, New York, United States, ^7^Instituto Nacional de Infectologia (INI), Fundação Oswaldo Cruz (FIOCRUZ), Rio de Janeiro, Brazil, ^8^McGovern Medical School, Houston, United States, ^9^San Francisco Department of Public Health, San Francisco, United States, ^10^Harvard Medical School, Boston, United States, ^11^Callen Lorde Community Health Center, New York, United States, ^12^Gilead Sciences, Foster City, United States, ^13^Kelly Government Solutions, Contractor to Division of AIDS, NIAID, National Institutes of Health, Rockville, United States, ^14^University of Pennsylvania School of Medicine, Philadelphia, United States, ^15^RTI International, Research Triangle Park, United States, ^16^University of Michigan School of Public Health, Ann Arbor, United States

**Background: **Transgender women (TW) have the highest HIV prevalence of any key population, yet experience suboptimal PrEP engagement. HPTN 091 assessed a novel, multicomponent, integrated service delivery strategy to enhance daily oral PrEP uptake and adherence.

**Methods: **The study enrolled 303 TW, ≥18 years, at four US sites (Houston, New York, Philadelphia and San Francisco) and one Brazil site (Rio de Janeiro). Participants were randomized 1:1 to Immediate (IA) or Deferred Arms (DA). All were offered Truvada® or Descovy®. The IA was offered co‐located gender‐affirming hormone therapy (GAHT) and six structured peer health navigation (PHN) sessions after enrolment, while the DA was linked to external GAHT until transition to IA interventions at week 26. Socio‐behavioural surveys and biomarkers were collected at baseline and week 26. PrEP adherence was assessed via intraerythrocytic tenofovir diphosphate (TFV‐DP) concentrations. PHN implementation was assessed via focus group discussions (FGDs) with all eight peer navigators.

**Results: **Median age at enrolment was 28 years (IQR: 25−35). Over half (54%) identified as Hispanic/Latina; 31% Black, 35% White and 28% another race. At enrolment, 11% and 42% reported ongoing PrEP and GAHT use, respectively, with no difference by arm. Acceptance of study‐provided PrEP at enrolment was 73% (71% IA, 75% DA). Ninety‐nine percent in the IA completed ≥1 PHN session (median: 6 sessions). At week 26, retention was similar across arms (85% IA, 89% DA). PrEP uptake (IA 86%, DA 88%, *p* = 0.65) and adherence (IA 44%, DA 48%, *p* = 0.48) rates were similar between arms based on drug concentrations consistent with ≥4 doses/week. The frequency of quantifiable TFV‐DP concentrations was comparable across arms (IA 70%, DA 76%, *p* = 0.25). While structured PHN sessions were limited to the IA, FGDs indicated that PHNs provided psychosocial support and referrals regardless of arm.

**Conclusions: **PrEP engagement was high among all participants, likely due to peer support and referrals to needed services in both study arms. Co‐location of PrEP/GAHT with structured PHN sessions was not associated with higher PrEP uptake nor adherence. Findings highlight the flexibility available to PrEP programmes to co‐locate PrEP/GAHT or facilitate access to external GAHT to promote PrEP engagement in the context of peer support for TW.

### OA2003 Acceptability of semiannual HIV pre‐exposure prophylaxis (PrEP) dispensing with interim HIV self‐testing for streamlined PrEP delivery in Kenya

N. Thuo ^1^, A.R. Bardon^2^, P. Mogere^1^, C. Kiptinness^1^, E. Casmir^1^, N. Wairimu^1^, E. Owidi^1^, P. Okello^1^, N. Mugo^1,3^, J.M. Baeten^3^, K.F. Ortblad^4^, K. Ngure^5^

^1^Kenya Medical Research Institute, Partners in Health and Research Development, Centre for Clinical Research, Nairobi, Kenya, ^2^Washington University, St Louis, United States, ^3^University of Washington, Department of Global Health, Seattle, United States, ^4^Fred Hutchinson Cancer Center, Public Health Sciences Division, Seattle, United States, ^5^Jomo Kenyatta University of Agriculture and Technology, School of Public Health, Nairobi, Kenya

**Background: **In Africa, the delivery of oral HIV pre‐exposure prophylaxis (PrEP) within already strained public health facilities has resulted in prolonged waiting times and suboptimal experiences for clients. We sought to explore the acceptability of dispensing PrEP semiannually supported with interim HIV self‐testing (HIVST) versus quarterly to optimize clinic‐delivered PrEP services.

**Methods: **We conducted a qualitative study within a randomized controlled trial testing the effect of 6 months PrEP dispensing supported with interim HIVST (semiannual clinic visits) compared to the standard 3 months PrEP dispensing (quarterly visits) on PrEP clinical outcomes in Kenya (NCT03593629). Eligible participants were ≥18 years, refilling PrEP for the first time and were either in an HIV sero‐different couple (men and women) or singly enrolled (women). We conducted serial in‐depth interviews (IDIs) with participants in the intervention arm at enrolment, 6 and 12 months. We applied thematic analyses of participants’ perceptions of the intervention and mapped themes to acceptability constructs outlined in the Theoretical Framework of Acceptability (TFA).

**Results: **Between May 2018 and June 2021, 55 participants completed 120 serial IDIs; 64% (35/55) of participants were in sero‐different couples, 64% (35/55) were women and the median age was 32 years (IQR 27−40 years). Overall, participants perceived this novel PrEP delivery model as highly acceptable; it was well‐liked (TFA: affective attitude) and less burdensome than the standard quarterly PrEP refill visits (TFA: burden). Participants also valued the increased privacy and confidentiality that came with HIV testing at home (TFA: ethicality) and were confident in their ability to participate in the intervention (TFA: self‐efficacy). Some participants, however, highlighted potential disadvantages of the model, including fewer opportunities for counselling with providers and potentially less accurate HIV testing (TFA: opportunity costs). Ultimately, most participants reported that the intervention allowed them to achieve their HIV prevention goals (TFA: perceived effectiveness) and discussed increased confidence in HIVST and PrEP use after each visit.

**Conclusions: **Semiannual PrEP clinic visits supported with 6 months dispensing and interim HIVST was perceived as highly acceptable among PrEP users who experienced the intervention in Kenya. More comprehensive pre‐intervention counselling and HIV self‐test training may help address the concerns presented.

### OA2004 Integrating strengths from various sources through a digital health platform: An online‐to‐offline service model for PrEP

Y. Gu ^1^, Y. Luo^1^, Y. Lu^2^, G. Meng^2^, L. Sun^2^, Y. Cai^1^, Z. Han^1^

^1^Guangzhou Center for Disease Control and Prevention, Guangzhou, China, ^2^Lingnan Partners Community Support Center, Guangzhou, China

**Background: **Over the last decade, global evidence has shown PrEP's high efficacy, reducing HIV acquisition risk by over 90% in MSM. China approved TDF/FTC for PrEP in 2020. However, PrEP services, primarily in HIV ART hospitals, face low utilization due to complex clinic procedures and limited LGBT friendliness. In Guangzhou, a prominent city in China, the proportion of MSM seeking PrEP services in 2022 was merely 4.9% compared to PEP cases in these HIV ART hospitals, emphasizing the need for convenient, LGBT‐friendly and complementary services.

**Methods: **The HIV digital health platform (“Chabei”), developed by our team, serves as a central hub connecting organizations to create an online‐offline PrEP service model. Initially, LGBT community‐based organizations (CBOs) provide online/offline MSM‐friendly consultation and assessments. Guangzhou centre for disease control and prevention, in collaboration with third‐party testing facilities, conducts offline testing for HIV, HBV/HCV/STIs and creatinine. Physicians familiar with ART medicines review, prescribe and facilitate the delivery of PrEP medications to MSM through online medical platforms. Subsequent follow‐up is managed by CBOs. The HIV digital health platform acts as a unified portal for online/offline services, integrating processes and data from different organizations to enhance efficiency, user experience and data flow. Consultation, assessment, testing and follow‐up services are provided free of charge, with MSM responsible only for medication costs.

**Results: **Launched in Guangzhou in December 2021, our model provided PrEP consultations for 920 MSM by June 2023. Of them, 223 underwent pre‐medication testing. Four with positive HIV antibodies were unsuitable, and six with positive HBV antigen were referred. Twenty‐seven had abnormal creatinine levels, but none precluded PrEP. Ultimately, 207 MSM initiated PrEP, 3.6 times more than all HIV ART hospitals in Guangzhou. 99.5% opted for event‐driven PrEP, with 50.7% in follow‐up. No HIV seroconversions occurred.

**Conclusions: **Our model integrates strengths from various sources through the HIV digital health platform, combining online convenience with standardized, safe and MSM‐friendly procedures. It serves as a distinctive complement to HIV ART hospitals. The model enhances the applicability of PrEP and holds potential for adaptation in other Chinese cities. Subsequent stages will explore streamlined processes and adaptable follow‐ups in line with WHO guidelines.

### OA2005 Promoting access to combined HIV prevention services by mobile populations (truck drivers) using health hub vans equipped with virtual health platforms at Kasumbalesa border in Zambia

J. Mwanza ^1^, A.P. Ndhlovu^1^, L. Kawanga^1^, M. Nyumbu^1^, M. Musonda^2^

^1^JSI/USAID DISCOVER Health, Lusaka, Zambia, ^2^USAID Zambia, Lusaka, Zambia

**Background: **Kasumbalesa boarder is the busiest border of Zambia due to the volume of trucks transporting commodities from East, Central and Southern Africa to the Democratic Republic of the Congo. Every day, between 1300 and 1500 long‐haul trucks are cleared in both directions. Most of the time, truck drivers are stranded at the border and must wait in enormous lines to be cleared. Due to security issues and the distance to the health facilities, truck drivers who spend the most of their time on the road have difficult or no access to healthcare, particularly combined HIV prevention treatments.

**Methods: **In June 2023, the USAID DISCOVER‐Health project implemented by JSI introduced innovative health hub vans equipped with virtual health platforms to enhance mobile populations’ (truck drivers’) access to combined HIV prevention services. The virtual health platform leverages technology to generate demand for combined HIV prevention services, while the health hub vans employ person‐centred concepts by converting a modified Landcruiser into a mobile clinic. The virtual health platform supports multiple languages that are utilized by truck drivers. At the baseline and endline, quantitative data were gathered and analysed using Microsoft Excel.

**Results: **Before the innovation, the data reveal that, out of the 32,753 new pre‐exposure prophylaxis (PrEP) clients in Fiscal Year 2022, 0% were mobile population (truck drivers). Following the innovation, truck drivers accounted for 2749 (5%) of the 55,257 PrEP new clients by September of Fiscal Year 2023. By March 2024, 202,174 condoms had been delivered, and 15,834 truck drivers had received various integrated HIV prevention messages and services, of which 6207 had accessed PrEP and 101 had accessed ART. The mobile population's access to PrEP increased by 126% between September 2023 and March 2024. In the same period, 2259 truck drivers accessed treatment for various sexually transmitted diseases as the service was integrated.

**Conclusions: **Health hub vehicles installed with virtual health platforms facilitated easier access to integrated HIV prevention services. The invention has thus been extended to key population such as female sex workers and other border regions like Sakania in Ndola. This innovation is recommended for mobile population.

### OA2006 Uptake of pre‐exposure prophylaxis among out‐of‐school girls aged 15−19

E. Sendaula ^1^, H. Achayo^2^, J. Kaleebi^3^

^1^Reach Out Mbuya Community Health Initiative, Uganda, Uganda, ^2^Reach Out Mbuya Community Health Initiative, Executive, Kampala, Uganda, ^3^Reach Out Mbuya Community Health Initiative, Kampala, Uganda

**Background: **In Uganda, adolescents make up a quarter of the population, yet they encounter a multitude of challenges that hinder their wellbeing. Factors such as poverty, HIV/AIDS, early marriage and limited educational opportunities significantly impact their lives. Notably, adolescent girls bear a disproportionate HIV burden, accounting for two‐thirds of new acquisitions. Despite the increasing recognition of pre‐exposure prophylaxis (PrEP) as an effective tool in HIV prevention, there exists a substantial gap in understanding its accessibility and acceptance, particularly among those who are out of school.

**Methods: **This was a cross‐sectional study involving 4185 adolescent girls and young women (AGYW) aged 15−19 years who were enrolled in the DREAMS programme between 1st September and 30th February 2024, in Kampala, Uganda. Information regarding socio‐demographics, initiation of pre‐exposure prophylaxis (PrEP) and factors contributing to HIV risk was obtained from an electronic medical records system. A root cause analysis was conducted to understand the factors underlying the low adoption of PrEP. Descriptive data analysis was performed using SPSS version 26.

**Results: **There was a low uptake of PrEP among adolescents aged 15−19 years, with only 2.9% (125) initiated on the regimen. Patterns of risky sexual behaviours were evident, with 45.6% (57) reporting irregular condom use, 10.4% (13) acknowledging multiple sex partners and 32.0% (40) engaging in transactional sex. Additionally, a considerable proportion of adolescent girls, totalling 55.2% (69), were found to be sexually active. A root cause analysis revealed various concerns contributing to the low uptake of PrEP among girls, including challenges with pill size and dosing, fear of stigma due to packaging resembling antiretroviral medication, and side effects such as nausea and headaches.

**Conclusions: **Despite the majority of AGYWs being sexually active, the low uptake of PrEP persists. Overcoming barriers necessitates the development of youth‐friendly formulations that address concerns related to pill size and dosing. Strategies aimed at mitigating stigma surrounding PrEP, including educational campaigns and community engagement to foster acceptance and uptake among adolescent girls. Additionally, healthcare providers should offer comprehensive information, counselling and support to PrEP users to ensure optimal utilization and effectiveness of the intervention.

### OA2102 A descriptive analysis of rapid response social and legal support activities on HIV prevention service delivery among LGBTQI+ clients in Uganda following passage of the 2023 Anti‐Homosexuality Act

S. Melillo ^1^, D. Kasozi^1^, W. Bikokye^1^, A. Renquist^1^, M. Tauras^1^

^1^USAID/Uganda, Kampala, Uganda

**Background: **Uganda's Anti‐Homosexuality Act 2023 (AHA)—passed by Parliament on 21 March 2023 and largely upheld by the Constitutional Court on 3 April 2024—poses grave threats to HIV epidemic control and LGBTQI+ human rights. It stipulates harsh penalties for “homosexual behaviour” and its “promotion,” ranging from 10 years up to the death penalty. USAID/Uganda's HIV/TB team collaborated with the Democracy, Human Rights, and Governance team to co‐fund and manage three Ugandan LGBTQI+‐led AHA rapid emergency response support awards in May 2023, recognizing that structural determinants were likely to impact HIV prevention uptake and continuity among LGBTQI+ people.

**Methods: **We analysed rapid response awards data (June 2023−February 2024), PEPFAR Key Population (KP) Tracker HIV dashboard data (March 2023−21 July 2023), and triangulated it with USAID HIV partner reports for referrals to rapid response mechanisms to identify any trends.

**Results: **PEPFAR data demonstrate a sharp increase in human rights incidents among LGBTQI+ clients following the passage of AHA, with a decrease of up to 60% in HIV service uptake at 84 PEPFAR‐supported KP drop‐in centres during that same period. Emergency activities responded to 2323 incidents, mostly among gay men or lesbians (62%) and transgender individuals (19%). The bulk of these incidents were eviction/forced relocation (42%), threats/discrimination (24%) and assault (17%). HIV prevention uptake among KPs largely rebounded to near normal levels beginning in June 2023. Data limitations prevent us from attributing HIV service delivery improvements solely to rapid response services, as USAID worked with the Ministry of Health (MOH), partners and LGBTQI+ civil society to develop concurrent HIV service delivery adaptations, while the MOH issued a helpful 5 June 2023 circular calling for non‐discriminatory client care in Ugandan health facilities regardless of sexual orientation.

**Conclusions: **Despite limitations, our analysis from Uganda suggests that working closely with LGBTQI+ leaders to provide emergency social and legal support, in combination with LGBTQI+‐led HIV service delivery adaptations, is a critical strategy for maintaining HIV prevention service continuity among LGBTQI+ people in the 6 months following passage of anti‐gay laws. These findings may inform policymakers in the rising number of African countries considering similar laws.

### OA2103 Hierarchical cluster analysis reveals a sub‐group of persons who inject drugs living with HIV that are more likely to experience sub‐optimal HIV care in Kenya

N. Ludwig‐Barron ^1,2^, L. Mbogo^3,4^, B. Sambai^4^, E. Juma^5^, A. Monroe‐Wise^2^, D. Bukusi^6^, S. Masyuko^7^, E. Gitau^8^, W. Sinkele^8^, H. Kingston^2^, B. Chohan^2^, C. Farquhar^2^, B. Guthrie^2^

^1^University of California, San Francisco, Medicine, San Francisco, United States, ^2^University of Washington, Department of Global Health, School of Public Health and School of Medicine, Seattle, United States, ^3^Emory University, Global Health, Atlanta, United States, ^4^University of Washington Global Assistance Program‐Kenya, Nairobi, Kenya, ^5^University of Washington Global Assistance Program‐Kenya, Mombasa, Kenya, ^6^VCT and HIV Prevention, Kenyatta National Hospital, Nairobi, Kenya, ^7^Kenyatta Ministry of Health, National HIV and STI Control Programme (NASCOP), Nairobi, Kenya, ^8^Support for Addictions Prevention and Treatment in Africa (SAPTA), Nairobi, Kenya

**Background: **In Kenya, persons who inject drugs living with HIV (PWID‐LH) struggle with suboptimal HIV care, defined as: >6 months without care, not on ART or virally unsuppressed (>1000 copies/ml). PWID‐LH are often referenced as a monolithic group, which overshadows underlying subgroups needing additional support. We conducted hierarchical cluster analyses (HCAs) to subdivide a population of PWID‐LH in Kenya and determined subgroups more likely to experience suboptimal HIV care.[Fig jia226351-fig-0016]

**Figure 1 jia226351-fig-0016:**
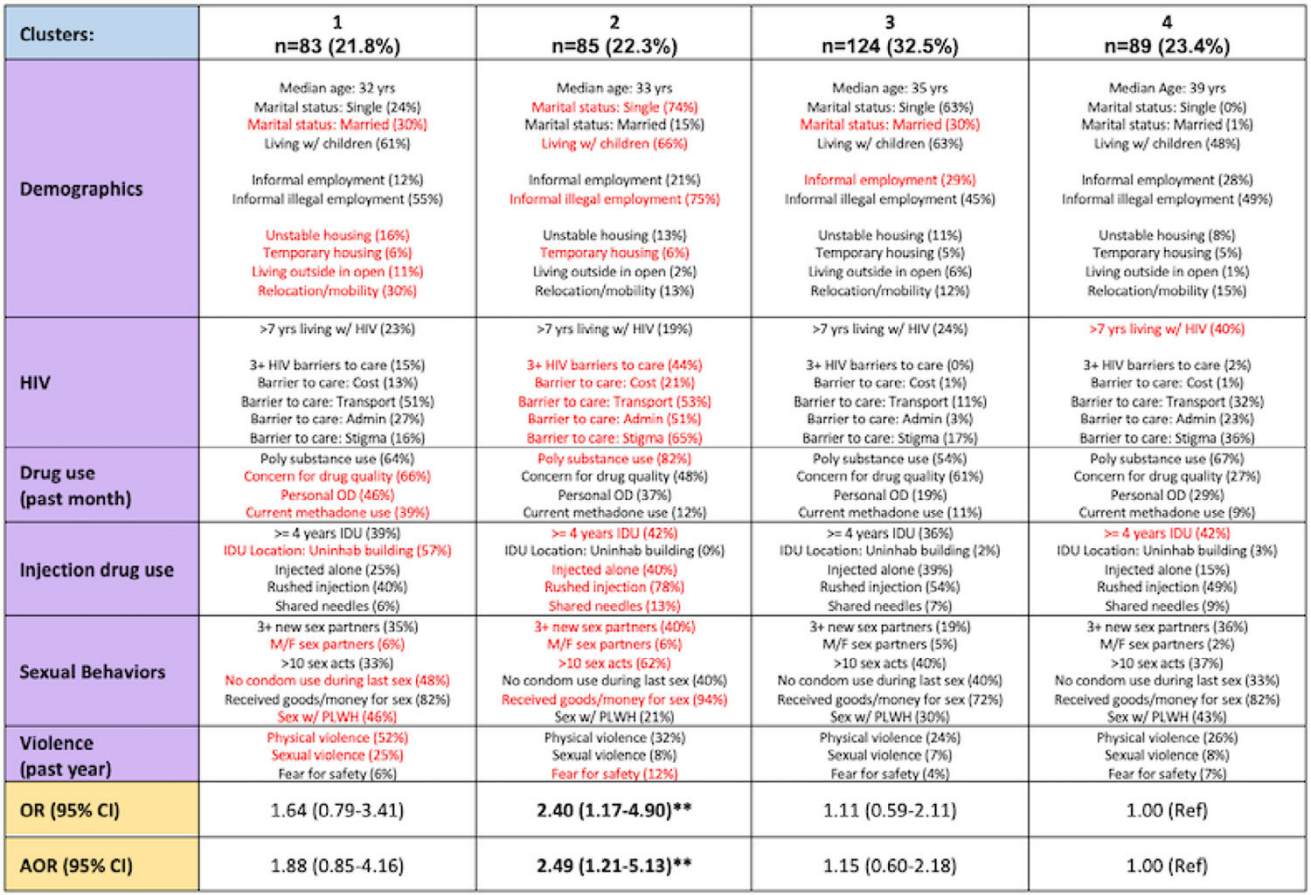
**OA2103**

**Methods: **Our analysis uses baseline data of 783 PWID‐LH enrolled in an HIV cohort study from 2017 to 2021 in Nairobi and Coastal Kenya. Recruitment occurred through harm reduction organizations, with eligibility criteria: age ≥18 years, HIV positive and injected drugs (previous year). We conducted an HCA for men and women that included 66 variables spanning demographics, HIV risks, drug use and violence. Logistic regression identified clusters of PWID‐LH associated with HIV suboptimal care, adjusting for recruitment region.

**Results: **Of the 783 PWID‐LH, nearly half were women (48.7%) and 33% experienced sub‐optimal HIV care. Among men, the median age was 38 years (IQR: 33−44 years), most injected heroin (91%) multiple times per day (83%) and engaged in transactional sex (48%). Among women, the median age was 35 years (IQR: 30−40 years), most injected heroin (92%) multiple times per day (76%) and engaged in transactional sex (81%). HCA revealed four clusters among men, with no significant associations between clusters and suboptimal HIV care; however, HCA identified four clusters among women and showed a cluster of young women (Cluster 2) twice as likely to experience sub‐optimal HIV care compared to a cluster of older women (Cluster 4) [AOR: 2.49; 95% CI: 1.21−5.13]. Women's cluster characteristics are presented (Table 1).

**Conclusions: **HCA highlighted a subgroup of women more likely to experience suboptimal HIV care in Kenya, which is fundamental for developing tailored HIV prevention interventions.

### OA2104 The Transgender Scorecard: ensuring representation in HIV prevention research

B. Minalga ^1^, C. Feuer^2^

^1^Fred Hutchinson Cancer Center, Office of HIV/AIDS Network Coordination (HANC), Seattle, United States, ^2^AVAC, New York, United States

**Background: **The field of HIV prevention research has begun to recognize transgender and gender‐diverse (TGD) people as a key population in the global HIV response. Though limited, estimates suggest that global HIV incidence is 66 times higher for transgender women, seven times higher for transgender men and unknown for gender nonbinary people (relative to cisgender people). Despite these stark disparities, TGD people remain underrepresented in HIV research, resulting in a data desert for HIV prevention in TGD communities. To address the underrepresentation of TGD people in HIV prevention research, HANC and AVAC sought to design an evidence‐based policy and advocacy tool to ensure that TGD people and priorities are considered in the design and implementation of all HIV prevention clinical trials. Here, we present the TGD Scorecard and findings from our analysis of TGD representation in HIV research over the past 35 years.

**Methods: **We undertook a project to synthesize global TGD HIV‐related priorities into a series of scoreable indicators of TGD representation in HIV clinical trials. We then pilot‐tested the Scorecard, assessing 41 milestone HIV studies that took place from 1991 to 2023. Source documents for this assessment included study protocols, study publications and study records on clinicaltrials.gov.

**Results: **Only 12 out of the 41 studies in our analysis included TGD people. From 1991 to 2006, there was no TGD representation in milestone HIV trials. Out of the 174,944 participants in the 38 completed studies, less than 1% were TGD (*n* = 1663). Among the TGD participants who were included, 90% were transgender women; transgender men and gender nonbinary participants represented only 3% and 7% of TGD participants, respectively. Only six studies reported the gender of participants in primary publications, and research systems to support TGD enrolment are underutilized.

**Conclusions: **Findings from our analysis of 41 milestone HIV clinical trials over the past 35 years confirm that TGD people remain underrepresented in HIV prevention research. Albeit, a trend towards greater inclusivity in recent years has been observed. These findings support our recommendation to utilize our TGD Scorecard as a policy and advocacy tool by researchers and advocates alike to operationalize our commitment to TGD representation in HIV prevention research.

### OA2105 Age of access policy reform needed for adolescents and PrEP in some sub‐Saharan African countries

S. Lynch ^1^, T. Parenti^1^, V. Srivatsan^1^, A. Sharma^1^, C. Parshall^1^, M. Kavanagh^1^

^1^O'Neill Institute for National and Global Health Law, Georgetown University, Center for Global Health Policy and Politics, Washington, DC, United States

**Background: **Adolescents are at increased risk of HIV acquisition, and account for 10% of all the new HIV infections in 2022. Pre‐exposure prophylaxis (PrEP) is an effective prevention method that vastly reduces the risk of HIV acquisition among high‐risk vulnerable populations. In this study, we examine national age of access (AOA) policies by evaluating if adolescents can access PrEP without parental consent. Where inconsistent with WHO recommendations, such policies, guided by the legal definition of adulthood, curtail adolescents’ sexual and reproductive rights, and risk limiting access to vital HIV services by requiring them to obtain consent from a parent or guardian.

**Methods: **The HIV Policy Lab, a collaboration between the O'Neill Institute for National and Global Health Law, UNAIDS, and the Global Network of People Living with HIV (GNP+) tracks the adoption status of 33 globally recommended laws and policies for 194 countries. To evaluate the Age of Access (AoA) for adolescents, we conducted a review of national policies to determine age restrictions on adolescents’ access to PrEP without parental consent.

**Results: **In our preliminary analysis, we found relevant PrEP guidelines for 28/46 countries in sub‐Saharan Africa (SSA)

In SSA, the region with the highest number of children and adolescents living with HIV (CALHIV), only 14/28 countries do not require parental consent for adolescents aged 12 years and above for PrEP. Among them, 65% (9/14) are PEPFAR‐supported countries, 50% (7/14) also have optimal AOA guidelines for HIV testing and 57% (8/14) also have optimal AOA guidelines for HIV treatment.

Out of the 28 countries where relevant guidelines were found: four (14%) require parental consent for individuals <15 years; two countries (7%) require parental consent for individuals <16 years, 3.5% (1/28) require parental consent for individuals <17 years, 11% (3/28) require parental consent for individuals ≤18 years and 7% (2/28) lack age‐specific parameters for access.

**Conclusions: **Countries still need to review and update their age consent policies to be in alignment with WHO testing guidelines for adolescents. Tracking and analysing the age of consent policies should be considered an important part of expanding adolescents’ access to HIV testing, prevention and care services.

### OA2106 Impact of public insurance expansion on equitable HIV pre‐exposure prophylaxis (PrEP) coverage in the United States: A staggered differences‐in‐differences approach

E.M. Stone^1,2^, J.G. Rosen ^3^

^1^Rutgers University Robert Wood Johnson Medical School, Department of Psychiatry, New Brunswick, United States, ^2^Rutgers University Institute for Health, Center for Health Services Research, New Brunswick, United States, ^3^Johns Hopkins Bloomberg School of Public Health, Department of International Health, Baltimore, United States

**Background: **Despite growing availability over the last decade, pre‐exposure prophylaxis (PrEP) coverage in the United States remains suboptimal and insufficient for population‐level HIV incidence reductions. Beginning in 2014, through a provision in the Affordable Care Act, US states could elect to expand public insurance (Medicaid) coverage to low‐income adults who were previously ineligible for this coverage. We evaluated the impact of Medicaid expansion on state‐level PrEP prescribing outcomes.

**Methods: **We abstracted HIV surveillance data from 50 US states (2012−2022) to assess the impact of Medicaid expansion on two state‐level PrEP indicators: PrEP coverage (the number of individuals prescribed PrEP per 100,000 residents) and the PrEP‐to‐need ratio (PnR, the number of individuals prescribed PrEP per new HIV diagnoses). To quantify the effects of Medicaid expansion on state‐level PrEP coverage and PnR, we used the Callaway‐Sant'Anna differences‐in‐differences method for staggered policy adoption, accounting for state‐level variations in the timing of Medicaid expansion. We assessed outcomes for the overall state population and across population strata (age, sex, race/ethnicity).

**Results: **We observed increases, albeit non‐significant, in PrEP coverage attributable to Medicaid expansion (average treatment effect on treated states [ATT] = 37.7, 95% confidence interval [CI]: −25.4 to 100.7). Medicaid expansion was associated with significant increases in the PnR (ATT = 4.45, CI: 2.45−6.45), with treatment effects growing over calendar time (Figure). In subgroup analyses, Medicaid expansion was associated with significant PnR increases across age strata and racial/ethnic groups (Black: ATT = 1.54, CI: 0.24−2.85; Hispanic/Latinx: ATT = 2.97, CI: 1.35−4.59; White: ATT = 10.95, CI: 1.72−20.17), as well as among males (ATT = 5.34, CI: 2.89−7.79).

**Conclusions: **Medicaid expansion was associated with a four‐fold increase in state‐level PnR, indicating public insurance expansion effectively increased PrEP access and initiations in populations with elevated risks of HIV acquisition. However, we observed differential impacts of Medicaid expansion by race/ethnicity, suggesting widening racial/ethnic PrEP disparities in the context of public insurance expansion.[Fig jia226351-fig-0017]

**Figure 1 jia226351-fig-0017:**
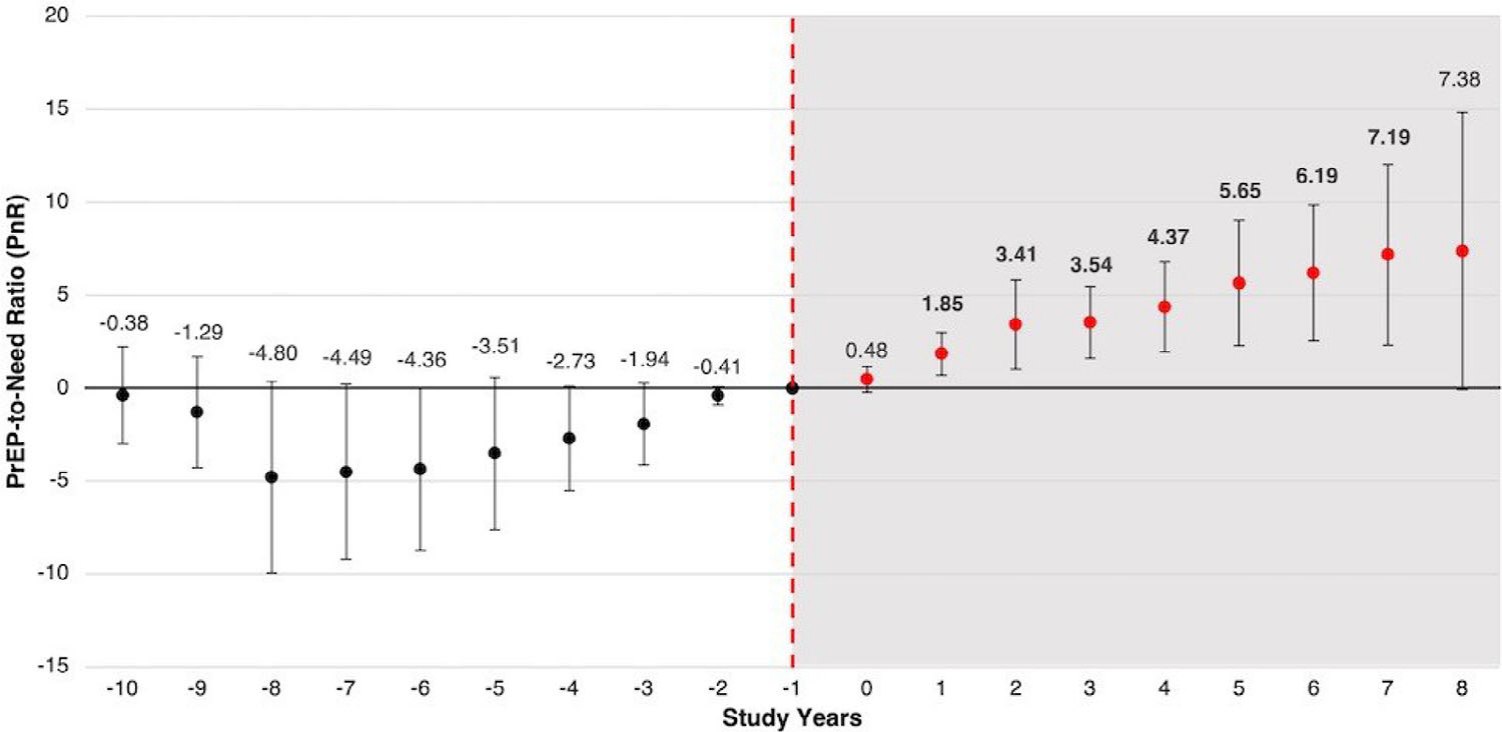
**OA2106**

### OA2202 Linking host sensing of microbiota, inflammation and HIV acquisition in the female genital tract

J.M. Gammon ^1^, S. Carpe Elias^1^, J. Frempong^1^, E. Lam^1,2^, M. Phelps^1,2^, J. Brady^1,2^, A. Balazs^1,2^, D. Kwon^1,2,3^

^1^Ragon Institute of MGH, Harvard and MIT, Medicine, Cambridge, United States, ^2^Harvard Medical School, Medicine, Cambridge, United States, ^3^Massachusetts General Hospital, Division of Infectious Diseases, Cambridge, United States

**Background: **Young women in sub‐Saharan Africa bear a disproportionate burden of global HIV infection with 80% of HIV‐infected women ages 15−24 years residing in this region. Previous studies have shown that South African women with cervicovaginal microbial communities dominated by diverse anaerobes and low *Lactobacillus* abundance experience heightened genital inflammation and a four‐fold increase in HIV acquisition rates. Certain species, such as *Prevotella bivia* (*P. bivia*) and *Sneathia sanguinegens* (*S. sanguingens*), abundant in these diverse communities, correlate with upregulated inflammatory pathways in cervical antigen‐presenting cells (APCs) and increased HIV acquisition risk in the female genital tract (FGT). No animal models exist for studying bacteria‐associated HIV infection in the FGT. Humanized mice, which reconstitute a human immune system, provide a platform for this purpose. We hypothesized that intravaginal inoculation of humanized mice with FGT bacteria could serve as a novel system to investigate genital inflammation and HIV risk.

**Methods: **Humanized mice were synchronized via sub‐cutaneous progesterone injection, followed 5 and 7 days later by intravaginal inoculation with *Lactobacillus crispatus* (*L. cripspatus*), *S. sanguingens* or *P. bivia*. Two days after the second bacterial inoculation, mice were intravaginally challenged with HIV. This cycle was repeated for 20 weeks, and HIV infection was analysed via RT‐PCR. Differences in infection rates were analysed by Log‐Rank test. Parallel experiments without HIV challenge analysed local inflammatory cytokine concentrations in cervicovaginal lavages by Luminex and immune responses in the FGT using flow cytometry. Differences were analysed using one‐way ANOVA with Tukey's post‐test.

**Results: **Intravaginal inoculation of humanized mice with *P. bivia* increased local concentrations of inflammatory cytokines and APC numbers compared to *L. crispatus*, while *S. sanguingens* inoculation resulted in a non‐significant increase in cytokine concentrations and APC numbers. Inflammatory cytokine concentrations positively correlated with cervicovaginal APC numbers. Inoculation with *P. bivia* significantly increased HIV infection rates in humanized mice (*p* = 0.039), while *S. sanguingens* drove a non‐significant increase in HIV infection (*p* = 0.11).

**Conclusions: **Intravaginal inoculation of humanized mice with genital bacteria induces inflammation and increased HIV infection. This system offers a novel *in vivo* model for modelling genital bacteria inflammation and evaluating potential interventions to prevent HIV infection in women.

### OA2203 Inhibitory activity of lactic acid isomers against *L. iners* and BV‐associated vaginal bacteria to prevent HIV transmission

P. Ellenberg ^1^, D. Tyssen^1^, J. Wilson^1^, S. Amir Hamzaha^1^, A. Hearps^1,2,3^, P. Gumbi^4,5^, C. Mehou‐Loko^6^, L.‐G. Bekker^6,7^, J.‐A. Passmore^4,6,8^, H. Jaspan^6,9^, H. Humphries^10,11^, L. Masson^1,2,6,4^, G. Tachedjian^1,3,12^

^1^Burnet Institute, Life Sciences, Melbourne, Australia, ^2^Monash University, Central Clinical School, Melbourne, Australia, ^3^University of Melbourne, Peter Doherty Institute, Melbourne, Australia, ^4^Centre for AIDS Programme of Research in South Africa, Durban, South Africa, ^5^University of KwaZulu‐Natal, Biochemistry Department, School of Life Sciences, Pietermaritzburg, South Africa, ^6^University of Cape Town, Institute of Infectious Disease and Molecular Medicine and Departments of Pathology and Immunology, Cape Town, South Africa, ^7^University of Cape Town, Desmond Tutu HIV Centre, Cape Town, South Africa, ^8^National Health Laboratory Service, Cape Town, South Africa, ^9^University of Washington, Seattle Children's Research Institute, Seattle, United States, ^10^Centre for Community‐Based Research, Human Science Research Council, Cape Town, South Africa, ^11^University of KwaZulu‐Natal, Department of Psychology, Pietermaritzburg, South Africa, ^12^Monash University, Department of Microbiology, Clayton, Australia

**Background: **In 2022, the global HIV burden remained high with women accounting for 46% of the 1.3 million new HIV infections (UNAIDS). Women with diverse, non‐optimal vaginal microbiota, depletion of optimal lactobacilli and local inflammation, as exemplified by bacterial vaginosis (BV), are at increased risk of acquiring HIV. We have discovered that lactic acid (LA), a metabolite produced by *Lactobacillus* species, has potent immunobiological activities that may help protect women from acquiring HIV. The aim of this study was to investigate the bactericidal activity of LA isomers against vaginal bacteria.

**Methods: **To determine bactericidal activity, cultures of ∼10^6^ colony forming units (CFU) of *L. iners* (ATCC 55195), *L. crispatus* (ATCC 33820) and *G. vaginalis* (ATCC 14018), and 4−5 different vaginal isolates from South African (SA) women (*L. crispatus*; and *L. iners* and *G. vaginalis* isolated from BV‐negative and BV‐positive women as determined by Nugent scoring), were treated with physiological levels of LA isomers 1% w/v (D‐LA and L‐LA) at different pH levels (3.8−7) at 37°C for 1 hour under anaerobic conditions. Cultures were then plated and viable bacteria quantified by determining CFU/ml. Bactericidal activity of LA isomers over time was investigated starting with cultures standard to 0.5 OD and propagated for 24 hours with viable bacteria determined as above at different time points.

**Results: **Treatment with LA isomers for 1 hour showed similar selective bactericidal activity against *G. vaginalis* (50,000‐fold reduction, *n* = 3 *p*<0.0001) and *L. iners* (128‐fold reduction, *n* = 6, *p* = 0.003), whereas *L. crispatus* (*n* = 3, *p*>0.93) viability was not affected compared to untreated bacteria. This activity was more potent than media at the same pH 3.8 indicating an LA‐specific effect. A similar pattern was observed with primary SA isolates. LA isomers showed potent selective bactericidal activity over 24 hours against *G. vaginalis* and *L. iners* (>5000 fold‐reduction in CFU/ml) versus untreated controls, while *L. crispatus* viability was not affected.

**Conclusions: **LA not only targets key BV‐associated vaginal bacteria but also the less stable and suboptimal *L. iners*, which persists following metronidazole treatment of BV. These data reveal a potential antibiotic sparing strategy to promote colonization with optimal lactobacilli as a potential intervention to prevent HIV acquisition.

### OA2204 Reduced abundance of colonic CD4^+^ tissue resident memory T cells despite early initiation of ART is linked to systemic inflammation and changes in microbiota composition

S. Vimonpatranon ^1^, A. Ssemaganda^2^, Y. Phuang‐Ngern^1^, C. Sajjaweerawan^1^, P. Saetun^1^, T. Shakery^2^, S. Sukhumvittaya^1^, S. Rathore^2^, R. Rerknimitr^3^, P. Ehrenberg^4^, N. Ratnaratorn^5^, S. Choi^2^, G. Severini^2^, D. Eiser^4,6^, R. Thomas^4^, L. Trautmann^4,6^, C. Sacdalan^5,7^, J. Cowden^1^, S. Vasan^4,6^, N. Phanuphak^8^, L.R. McKinnon^2,9,10^, A. Schuetz^1,4,6^

^1^Walter Reed Army Institute of Research‐Armed Forces Research Institute of Medical Sciences, Retrovirology, Bangkok, Thailand, ^2^University of Manitoba, Winnipeg, Canada, ^3^Chulalongkorn Hospital, Department of Medicine, Faculty of Medicine, Bangkok, Thailand, ^4^Walter Reed Army Institute of Research, U.S. Military HIV Research Program, CIDR, Silver Spring, United States, ^5^SEARCH Research Foundation, Bangkok, Thailand, ^6^Henry M. Jackson Foundation for the Advancement of Military Medicine, Inc, Silver Spring, United States, ^7^Chulalongkorn University, Research Affairs, Faculty of Medicine, Bangkok, Thailand, ^8^Institute of HIV Research and Innovation (IHRI), Bangkok, Thailand, ^9^Centre for the AIDS Programme of Research in South Africa (CAPRISA), Durban, South Africa, ^10^University of Nairobi, Department of Medical Microbiology and Immunology, Nairobi, Kenya

**Background: **HIV is primarily a disease of tissues, which are irreversibly damaged during acute HIV infection (AHI). CD4^+^ tissue resident memory T cells (Trm) play an important role in mucosal homeostasis but may also be preferential targets of HIV infection. Here, we assess the impact of early initiated ART on mucosal CD4^+^ Trm in relation to systemic inflammation and the local microbiome.

**Methods: **A cross‐sectional study was conducted in 16 people living without HIV (PWOH) and 35 people living with HIV (PWH), who initiated ART during early AHI (Fiebig [F] stage I to V) and were treated for ≥2 years. All participants underwent optional sigmoid colon biopsy. Mucosal immune phenotyping was carried out using flow‐cytometry, with Trm defined as CD4^+^CD103^+^CD69^+^ and non‐Trm as CD4^+^CD103^−^/CD69^−^. Soluble biomarkers were evaluated using ELISA and the mucosa‐associated microbiome was assessed using 16s rRNA sequencing.

**Results: **Mucosal Trm had higher CCR5 expression compared to non‐Trm (Trm: 81.7%, non‐Trm: 38.4%; *p*<0.001). In PWH, the frequency of total CD4^+^ T cells was slightly reduced when ART was initiated during FI/II compared to PWOH (PWH: 44.8% vs. PWOH: 48.9%; *p* = 0.11). However, if ART was initiated in FIII and later, a significant decrease in CD4^+^ T cells was observed (PWH: 40.4% vs. PWOH: 48.9%; *p*<0.001). Interestingly, the depletion was preferentially seen in Trm and was already observed when ART was initiated as early as FI/II (FI/II: 2.28% PWH vs. 3.87% PWOH, *p* = 0.03; FIII‐V: 2.39% PWH vs. 3.87% PWOH, *p* = 0.03). No statistically significant difference was observed in the population of non‐Trm. The frequency of Trm correlated inversely with plasma levels of Eotaxin‐3 (*r* = −0.46, *p* = 0.04) and MDC (*r* = −0.52, *p* = 0.02). Additionally, we observed a positive correlation between the frequency of Trm and the abundance of *Lachnospiraceae* (*r* = 0.61, *p* = 0.01), a taxa that plays a major role in mucosal barrier maintenance.

**Conclusions: **Overall, these data suggest that CD4^+^ Trm are preferentially depleted and/or not replenished, even if ART is initiated as early as FI/II. Trm depletion is associated with increased systemic inflammation and changes in the microbiota composition, which may be detrimental to mucosal barrier integrity. Future interventions to restore Trm may be warranted to limit systemic inflammation during ART.

### OA2205 Impact of antimicrobials on foreskin HIV susceptibility, immunology and bacteria in uncircumcised men from Uganda: a randomized, open‐label, phase I/II clinical trial

R. Kaul ^1^, B. Okech^2^, D. Park^3^, L. Buchanan^4^, Z. Shao^4^, B. Bagaya^2^, J. Mpendo^2^, V. Joag^1^, S. Yegorov^1^, A. Nanvubya^2^, V. Biribawa^2^, T. Namatovu^2^, B. Kawoozo^2^, A. Ssetaala^2^, M. Muwanga^5^, S. Huibner^1^, A. Tobian^6^, C. Liu^3^, J. Prodger^4^, R. Galiwango^7^

^1^University of Toronto, Medicine/Infectious Diseases, Toronto, Canada, ^2^UVRI‐IAVI HIV Vaccine Program, Entebbe, Uganda, ^3^George Washington University, Department of Environmental and Occupational Health, Washington, United States, ^4^Western University, Departments of Microbiology and Immunology and Epidemiology and Biostatistics, London, Canada, ^5^Entebbe General Hospital, Entebbe, Uganda, ^6^Johns Hopkins University, Pathology, Baltimore, United States, ^7^Rakai Health Sciences Program, Kalisizo, Uganda

**Background: **Bacteria Associated with Seroconversion, Immunology and Cells (BASIC species) may enhance penile HIV susceptibility in heterosexual uncircumcised men by inducing inflammatory cytokines that recruit HIV‐susceptible CD4^+^ target cells to the inner foreskin. We performed a phase 1/2 clinical trial examining the impact of antimicrobials on *ex vivo* foreskin HIV susceptibility, penile immunology and BASIC species.

**Methods: **This randomized open label clinical trial allocated 125 HIV‐uninfected Ugandan men seeking voluntary medical male circumcision (VMMC) to one of five arms (*n* = 25): immediate VMMC (control group), oral tinidazole (TZ) for 2 days, or one of topical metronidazole (MTZ), topical clindamycin (CDM) or topical hydrogen peroxide (HP) twice daily for 1 week then biweekly until VMMC at 4 weeks. The primary endpoint was *ex vivo* HIV pseudovirus entry into foreskin‐derived CD4^+^ T cells. Secondary endpoints were foreskin immune parameters, the preputial density of BASIC bacteria and product tolerability.

**Results: **A total of 125 participants were enrolled and randomized; 116 (93%) completed the protocol. Demographics did not vary significantly between groups, and all antimicrobial treatments were well tolerated. Oral TZ and topical MTZ reduced the inner foreskin tissue density of HIV‐susceptible CD4^+^ T cells. Oral TZ, topical MTZ and topical CDM also reduced the density of other key CD4^+^ T‐cell targets, including CCR5/CD4^+^ T cells and Th17 cells. All topical antimicrobials reduced BASIC species and enhanced epithelial integrity; MTZ and CDM also reduced inflammatory cytokines in the prepuce. Changes in epithelial integrity and cytokines were strongly correlated with alterations in the abundance of BASIC bacteria.

**Conclusions: **This phase 1/2 randomized clinical trial provides the first proof‐of‐principle that antimicrobials can reduce *ex vivo* HIV entry into foreskin‐derived T cells, reduce coronal sulcus inflammation, enhance foreskin epithelial integrity and reduce numerous T‐cell subsets in foreskin tissue. These effects were mediated via a reduction in BASIC species linked to HIV acquisition. A more in‐depth analysis of microbiome effects may guide specific agent selection for future clinical trials.

### OA2206 Microbiota effects of a *Lactobacillus crispatus* live biotherapeutic to prevent recurrent bacterial vaginosis: Findings from a randomized, placebo‐controlled trial

S. Bloom ^1,2,3^, L. Symul^4^, J. Elsherbini^3^, J. Xu^3^, S. Hussain^3^, J. Shih^3^, A. Sango^3^, C. Mitchell^2,3,5^, A. Hemmerling^6^, F. Hussain^3^, A. Kannan^3^, C. Cohen^6^, S. Holmes^7^, D. Kwon^1,2,3^

^1^Massachusetts General Hospital, Infectious Diseases, Boston, United States, ^2^Harvard Medical School, Boston, United States, ^3^Ragon Institute of Mass General, MIT, and Harvard, Cambridge, United States, ^4^UCLouvain, Louvain‐la‐Neuve, Belgium, ^5^Massachusetts General Hospital, Department of Obstetrics and Gynecology, Boston, United States, ^6^University of California, San Francisco, San Francisco, United States, ^7^Stanford University, Department of Statistics, Palo Alto, United States

**Background: **Bacterial vaginosis (BV) affects ≥25% of women globally and increases risk for HIV and other diseases. BV is characterized by pro‐inflammatory, *Lactobacillus*‐deficient vaginal microbiota and frequently recurs after standard treatment with metronidazole (MTZ). A phase 2b randomized, controlled trial found that Lactin‐V, a live biotherapeutic product containing *Lactobacillus crispatus* strain CTV‐05, significantly reduced recurrent BV (rBV) compared to placebo when administered after MTZ. However, efficacy was incomplete, with 39% of Lactin‐V recipients developing rBV within 6 months. We analysed samples from the trial to assess microbiota and immune effects and correlates of treatment success.

**Methods: **The multi‐centre trial conducted in the United States enrolled premenopausal, non‐pregnant women aged 18−45 years with BV (≥3 Amsel criteria and Nugent score ≥4). All received MTZ, then were randomized 2:1 to 11 weeks of intravaginal Lactin‐V or placebo. Vaginal swabs from 142 Lactin‐V and 70 placebo recipients were collected pre‐MTZ, post‐MTZ (at randomization), and at 4, 8, 12 and 24 weeks post‐randomization. Microbiota composition, cytokines and bacterial load were measured by sequencing, Luminex assay and qPCR, respectively. CTV‐05 strain percentage in metagenomes was estimated using StrainFacts, supported by bacterial isolations. Multi‐block analyses of “omic, clinical, behavioural and demographic data” provided weight‐ranked correlates of treatment success.

**Results: **Lactin‐V increased rates of high‐level (≥50%) *L. crispatus* colonization by 3.2‐fold (95% CI: 1.3−7.7; Figure) at week 12 and by 4.4‐fold (95% CI: 1.6−11.6) at week 24. CTV‐05 colonization accounted for much of this effect, but ∼30% of participants with high‐level *L. crispatus* colonization at week 24 had a dominant strain other than CTV‐05. BV‐associated cytokines including IL‐1B, IL‐1A and TNFa decreased in both arms after MTZ, reverting to baseline by week 24 in the placebo but not the Lactin‐V arm. *L. crispatus* colonization in Lactin‐V recipients correlated positively with product adherence and inversely with post‐MTZ total bacterial load, sexual activity, douching, lower education levels and Black/African‐American race.

**Conclusions: **An *L. crispatus* biotherapeutic improved BV treatment outcomes by enhancing high‐level *L. crispatus* colonization. Identification of factors linked to treatment success suggests biological and behavioural targets to improve product efficacy.

### OA2302 Pregnancy and infant outcomes among individuals exposed to dapivirine ring during the first trimester of pregnancy in the MTN‐025/HOPE open‐label extension trial

A. Mayo ^1^, R. Scheckter^1^, L. Chinula^2^, B. Gati Mirembe^3^, L.E. Mansoor^4^, N. Mgodi^5^, L. Naidoo^6^, T. Palanee‐Phillips^7^, N. Singh^6^, S. Siva^6^, D.W. Szydlo^8^, E. Brown^8^, J. Baeten^9^, L. Noguchi^10^, R.H. Beigi^11^, L. Soto‐Torres^12^, K. Bunge^11^

^1^FHI 360, Science Facilitation, Durham, United States, ^2^University of North Carolina Project Malawi, Department of Obstetrics and Gynecology's Division of Global Health, Lilongwe, Malawi, ^3^Makerere University ‐ Johns Hopkins University Research Collaboration, Kampala, Uganda, ^4^Centre for the AIDS Programme of Research in South Africa (CAPRISA), University of KwaZulu‐Natal, Durban, South Africa, ^5^University of Zimbabwe Clinical Trials Research Centre (UZ‐CTRC), Harare, Zimbabwe, ^6^South African Medical Research Council (SAMRC), Durban, South Africa, ^7^Wits Reproductive Health and HIV Institute (WRHI), Johannesburg, South Africa, ^8^Statistical Center for HIV/AIDS Research and Prevention (SCHARP), Fred Hutchinson Cancer Center, Seattle, United States, ^9^University of Washington, Department of Global Health, Seattle, United States, ^10^Jhpiego/Johns Hopkins University, Washington, DC, United States, ^11^University of Pittsburgh, Department of Obstetrics, Gynecology, and Reproductive Sciences, Pittsburgh, United States, ^12^National Institute of Allergy and Infectious Diseases, National Institutes of Health, Bethesda, United States

**Background: **Understanding the available safety data of the dapivirine vaginal ring (DVR) when used for HIV prevention during pregnancy is important to support expanded use of this PrEP method in this population.

**Methods: **Participants previously enrolled in MTN‐020/ASPIRE were offered the DVR in the open‐label extension study, MTN‐025/HOPE. Participants were HIV negative, not pregnant or breastfeeding and using an effective contraceptive with intention to continue use at enrolment. The DVR was provided monthly for the first 3 months, then quarterly thereafter. Participants could decline DVR and remain on study. Pregnancy testing was performed at all follow‐up visits and DVR discontinued on awareness of pregnancy. We describe the incident pregnancies in MTN‐025/HOPE and their outcomes.

**Results: **There were 70 participants with at least one pregnancy among 1456 participants enrolled in MTN‐025/HOPE over 1402 person‐years (5.0 pregnancies/100 person‐years). One participant was excluded from the incidence calculation for being pregnant at enrolment and one became pregnant twice. The median gestational age at the time of positive pregnancy test was 45 days (IQR: 30, 64) based on estimated date of delivery (EDD). Of 72 total pregnancies, 59 (82%) had potential for exposure to DVR. Seventy pregnancy outcomes had data available, including one multiple gestation (twins). Adverse pregnancy outcomes were uncommon, did not differ based on potential DVR exposure and were similar to those observed in MTN‐020/ASPIRE (Table 1). Four cases (6%) of gestational hypertension were observed; there were no other pregnancy complications reported. The median birthweight of infants was 3.3 kg (IQR: 2.8, 3.7), with five babies weighting <2.5 kg (11%), and no congenital anomalies.

**Conclusions: **There were no notable adverse effects on pregnancy or infant outcomes observed when DVR was used during early pregnancy. These findings add[Table jia226351-tbl-0012] to the growing evidence that the DVR is safe to use throughout pregnancy.

**Table 1 jia226351-tbl-0012:** OA2302: Pregnancy outcomes in MTN‐025/HOPE compared to MTN‐020/ASPIRE

	MTN‐020/ASPIRE^1^			MTN‐025/HOPE		
Pregnancy outcomes^a^	Placebo (*N* = 94)	DVR (*N* = 87)	All (*N* = 181)	No DVR (*N* = 12)	DVR (*N* = 58)	All (*N* = 70)
Full‐term birth (≥37 weeks)	53 (56%)	52 (60%)	**105 (58** **%)**	8 (67%)	36 (62%)	**44 (63%)**
Preterm birth (<37 weeks)	9 (10%)	0 (0%)	**9 (5%)**	0 (0%)	3 (5%)	**3 (4%)**
Stillbirth/intrauterine foetal demise (≥20 weeks)	2 (2%)	2 (2%)	**4 (2%)**	0 (0%)	3 (5%)	**3 (4%)**
Spontaneous abortion (<20 weeks)	21 (22%)	18 (21%)	**39 (22%)**	3 (25%)	11 (19%)	**14 (20%)**
Therapeutic/elective abortion	8 (9%)	14 (16%)	**22 (12%)**	1 (8%)	5 (9%)	**6 (9%)**
Ectopic pregnancy	1 (1%)	1 (1%)	**2 (1%)**	0 (0%)	0 (0%)	**0 (0%)**

The bold values indicate the total numbers for each of the two groups.

^a^In ASPIRE, 175 pregnancies resulted in a single outcome, three pregnancies resulted in two outcomes (twins) and one pregnancy had no outcome available, resulting in a total of 181 pregnancy outcomes. In HOPE, 68 pregnancies resulted in a single outcome, one pregnancy resulted in two outcomes (twins), three pregnancies had no outcome available, resulting in a total of 70 pregnancy outcomes.

^1^Makanani B, Balkus JE, Jiao Y, et al. Pregnancy and infant outcomes among women using the dapivirine vaginal ring in early pregnancy. J Acquir Immune Defic Syndr. 2018;79(5):566−572.

### OA2303 Motivations of pregnant women initiating PrEP within antenatal care enrolled in a randomized trial to improve adherence: the mWACh‐PrEP study

L. Gómez ^1^, J. Kinuthia^2^, F. Abuna^2^, E. Akim^2^, K. Beima‐Sofie^1^, J. Dettinger^1^, G. John‐Stewart^1^, M. Marwa^2^, N. Ngumbau^2^, J. Mogaka^1^, K. Ngure^3^, B. Odhiambo^2^, J. Stern^1^, J. Unger^1^, S. Watoyi^2^, J. Pintye^1^

^1^University of Washington, Seattle, United States, ^2^Kenyatta National Hospital, Nairobi, Kenya, ^3^Jomo Kenyatta University, Nairobi, Kenya

**Background: **The mWACh‐PrEP study (NCT04472884) is an RCT evaluating an interactive text messaging platform to improve adherence among women initiating PrEP during routine antenatal care at five clinics in Kenya. We analysed baseline data from the mWACh‐PrEP study to describe characteristics and motivations of pregnant women initiating PrEP.

**Methods: **Enrolled women were HIV negative, ≥18 years, between 24 and 32 weeks gestation, had high HIV risk scores (corresponding to HIV incidence 7.3 per 100 person‐years) and initiated daily oral PrEP that day. Participants were randomized 1:1 to receive the mWACh‐PrEP adherence support intervention or standard of care (in‐clinic adherence counselling only) and were followed through 9 months postpartum. The intervention platform sends PrEP‐tailored, theory‐based, pre‐programmed SMS to PrEP users on a weekly basis and allows users to communicate via text message with a remote nurse.

**Results: **Between January 2022 and July 2023, 600 cisgender women initiating PrEP enrolled during pregnancy at a median gestational age of 26 (IQR:24−29) weeks; 36% were primigravida. Median age was 25 years (22−29) and 71% of women were married, of which 11% were in a polygamous marriage. Only 5% of women had tested for HIV with their partners during this pregnancy and most (95%) women did not know their partners’ HIV status, while 3% had partners known to be living with HIV. Over one‐third (39%) of women believed their partner had other sexual partners and 1% tested positive for syphilis during pregnancy. All women had high HIV risk scores, yet only 36% perceived high HIV risk. Perceiving high HIV risk was more common among women who had symptoms of an STI (66% vs. 35%, *p*<0.05). Only 9% personally knew someone who was taking PrEP. The most frequent reasons for initiating PrEP were not knowing their partner HIV status (92%), wanting to protect their baby from HIV (51%), feeling at risk for HIV (40%) and believing their partner has other sexual partners (39%). Almost all (96%) reported high self‐efficacy for taking PrEP pills.

**Conclusions: **Knowledge of partner HIV status was extremely low, yet PrEP self‐efficacy was high among pregnant women initiating PrEP who enrolled in an mHealth trial aiming to improve PrEP adherence.

### OA2304 Preferences for long‐acting PrEP among pregnant and breastfeeding women in Southern Africa: A discrete choice experiment study

L.A. de Vos ^1^, A. Mussa^2,3^, E. Rousseau^4^, M. Strauss^5^, G. George^5^, P. Vundhla^4^, A. Gebengu^1^, C. Morroni^2,6^, R.PH. Peters^1,7^, C.N. Babalola^8^, J.D. Klausner^8^, D. Joseph Davey^4,9,10^

^1^Foundation for Professional Development, Research Unit, East London, South Africa, ^2^Botswana Harvard Health Partnership, Gaborone, Botswana, ^3^University of Edinburgh, Usher Institute, Edinburgh, United Kingdom, ^4^Desmond Tutu HIV Centre, Cape Town, South Africa, ^5^Health Economics and HIV and AIDS Research Division (HEARD), University of KwaZulu‐Natal, Durban, South Africa, ^6^University of Edinburgh, MRC Centre for Reproductive Health, Edinburgh, United Kingdom, ^7^University of Pretoria, Department of Medical Microbiology, Pretoria, South Africa, ^8^Keck School of Medicine, University of Southern California, Department of Population and Public Health Science, Los Angeles, United States, ^9^University of California Los Angeles, Division of Infectious Diseases, Geffen School of Medicine, Los Angeles, United States, ^10^University of Cape Town, Division of Epidemiology and Biostatistics, School of Public Health and Family Medicine, Cape Town, South Africa

**Background: **South Africa and Botswana have one of the highest HIV prevalence globally, particularly affecting women during pregnancy and postpartum, who face a two‐fold increased HIV acquisition risk. Persistent structural and individual barriers hinder women's daily oral PrEP effective use. A discrete choice experiment (DCE) among pregnant and breastfeeding women (PBFW) not living with HIV explored preferences for long‐acting PrEP, and multi‐purpose prevention technologies, to inform delivery.

**Methods: **Between April and December 2023, we evaluated preferences for long‐acting PrEP among PBFW accessing maternal services at primary healthcare facilities. The cohort included PBFW with PrEP use experience (Cape Town, South Africa), and those unfamiliar with PrEP (East London, South Africa and Gaborone, Botswana). Data collection incorporated surveys and discrete choice experiments (DCEs), with hypothetical scenarios describing various options. This approach was informed by qualitative interviews and focus group discussions. Analysis included demographic characterization, site stratification and latent class modelling.

**Results: **The study surveyed 450 PBFW (52% pregnant, 47% breastfeeding); median age was 26 (IQR 22−31). Women strongly favoured non‐vaginally inserted (coefficient −1.57, 95% CI −1.89 to −1.29) and non‐implanted (coefficient −0.79, 95% CI −1.00 to −0.59) PrEP compared to oral PrEP. HIV, STI and pregnancy prevention combination prevention (coefficient 1.02, 95% CI 0.80−1.24) was preferred over HIV prevention only. Site‐specific differences were evident (Figure 1), with clinic PrEP pick‐up preferred in East London and Gaborone compared to pharmacy or community delivery. PBFW in East London and Gaborone prioritized effectiveness over frequency of use. Three latent classes emerged: Class 1 (43%) prioritized combination prevention and less frequent dosing; Class 2 (25%) focused on physiological aspects; Class 3 (32%) avoided vaginal insertion, preferring specific pickup locations.

**Conclusions: **PrEP modality, frequency and pickup location are crucial in PrEP delivery choices. Future PrEP programmes should prioritize user‐centred approaches, ensuring alignment with PBFW's values and preferences to foster effective use.[Fig jia226351-fig-0018]

**Figure 1 jia226351-fig-0018:**
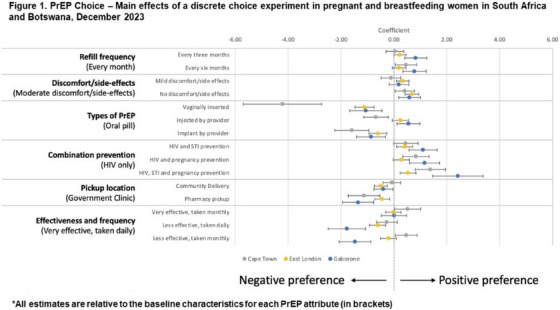
**OA2304**

### OA2305 Discrete choice experiment on the preferences for long‐acting pre‐exposure prophylaxis (PrEP) among pregnant women without HIV in Kisumu and Siaya, Kenya

T. Concepcion ^1^, J. Kinuthia^2^, F. Abuna^2^, E. Akim^2^, J. Dettinger^1^, L. Gómez^1^, E. Mukenyi^2^, N. Ngumbau^2^, J. Nyabiage^1^, B. Ochieng^2^, S. Watoyi^2^, J. Pintye^3^

^1^University of Washington, Global Health, Seattle, United States, ^2^Kenyatta National Hospital, Nairobi, Kenya, ^3^University of Washington, Nursing, Seattle, United States

**Background: **Suboptimal adherence to daily oral PrEP is common during pregnancy. New long‐acting (LA‐) PrEP methods may overcome barriers to taking daily oral PrEP, though research on long‐acting PrEP among pregnant women lags behind other populations. Incorporating feedback from pregnant end‐users could help understand preferences for PrEP attributes to support acceptability and effective use as long‐acting methods are introduced.

**Methods: **We conducted a discrete choice experiment (DCE) among pregnant women without HIV who initiated daily oral PrEP during antenatal care and were enrolled in an ongoing RCT in Kisumu and Siaya Counties, Kenya (NCT04472884). In a series of 12 questions, pregnant participants were asked to choose between three hypothetical PrEP products composed of five attributes (form and dose, collection place, effectiveness, side effects and availability of safety data). We used latent class modelling to identify patterns of PrEP preferences based on attributes.

**Results: **Between February 2023 and April 2024, 151 women completed the DCE. The median age was 24 years (IQR 21–29), median gestational age was 37 weeks (IQR 36.2−38.4), 84.8% had a partner of unknown HIV status and 4.0% had a partner known to be living with HIV. We identified four patterns of preferred PrEP attributes, characterized as “Primarily prefers injection form and high effectiveness driven” (29.2% of participants), “High safety and high effectiveness driven” (40.0%), “Oral PrEP or no PrEP” (6.6%) and “Injection form or No PrEP, high safety, and high effectiveness driven” (24.2%)

**Conclusions: **Our results indicate that there is a large proportion of pregnant women with a high HIV risk who are interested in a long‐acting injectable PrEP. PrEP attributes such as form/dosing, effectiveness and safety drove PrEP preferences among pregnant women. Accruing safety data on the use of long‐acting PrEP methods in pregnancy will support introduction into pregnant populations.[Fig jia226351-fig-0019]

**Figure 1 jia226351-fig-0019:**
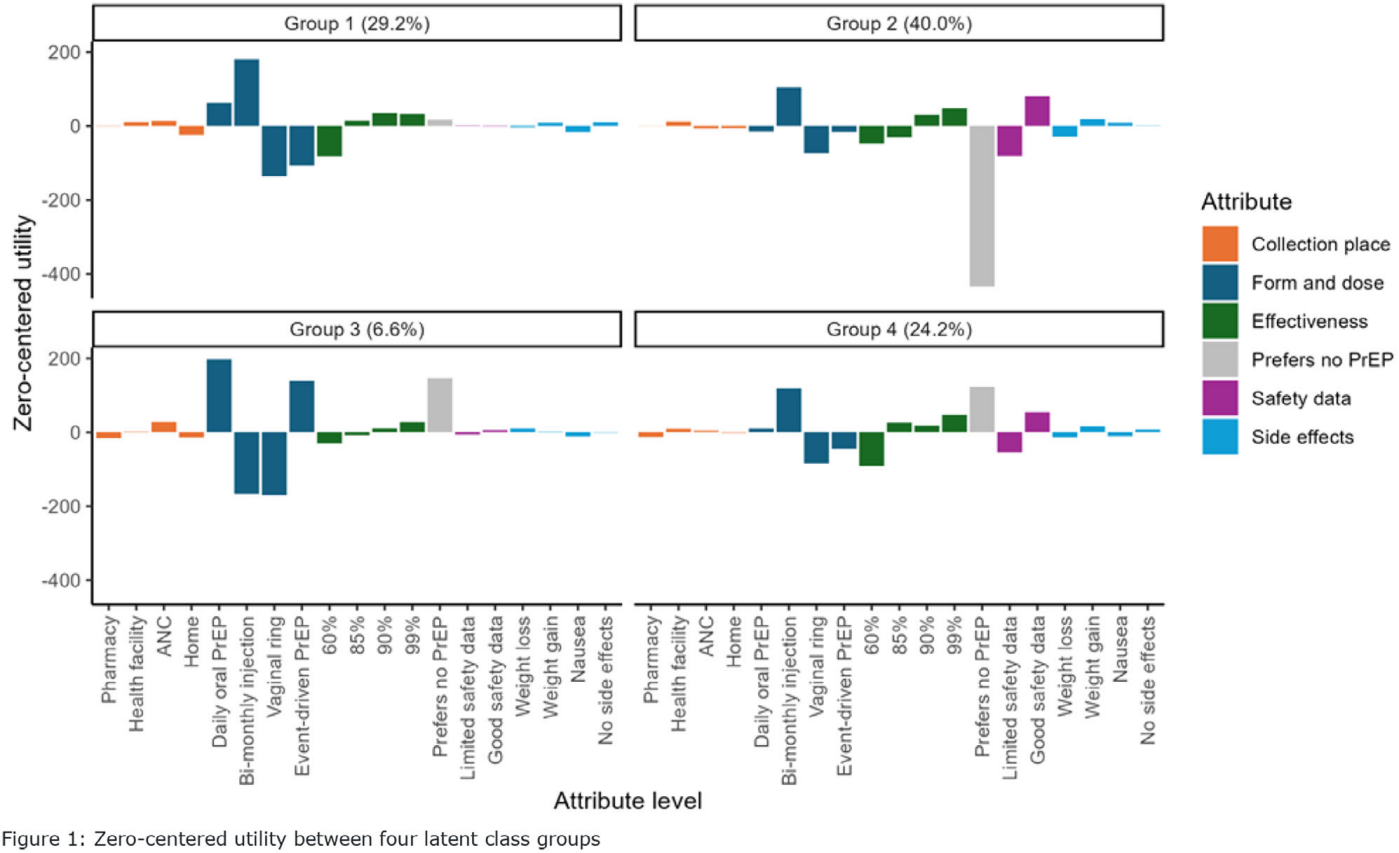
**OA2305**

### OA2306 Evaluation of potential pharmacologic interactions between CAB‐LA or TDF/FTC and hormonal contraceptive agents: A tertiary analysis of HPTN 084

M. Marzinke ^1^, B. Hanscom^2^, D. Haines^2^, K. Scarsi^3^, Y. Agyei^1^, S. Rose^4^, C. Mathew^5^, R. Panchia^6^, E. Spooner^7^, D. Kalonji^7^, N. Singh^7^, P. Bock^8^, A. Rinehart^9^, S. Ford^10^, J. Rooney^11^, L. Soto‐Torres^12^, M. Cohen^13^, M. Hosseinipour^13,14^, S. Delany‐Moretlwe^5^, HPTN 084 Study Team

^1^Johns Hopkins University School of Medicine, Pathology, Baltimore, United States, ^2^Fred Hutchinson Cancer Center, Vaccine and Infectious Disease Division, Seattle, United States, ^3^University of Nebraska Medical Center, College of Pharmacy, Omaha, United States, ^4^FHI 360, Durham, United States, ^5^University of the Witwatersrand, Wits RHI, Johannesburg, South Africa, ^6^University of the Witwatersrand, Perinatal HIV Research Unit, Johannesburg, United States, ^7^South African Medical Research Council, HIV and other Infectious Diseases Research Unit, Durban, South Africa, ^8^Stellenbosch University, Desmond Tutu TB Centre, Department of Paediatrics, Cape Town, South Africa, ^9^ViiV Healthcare, Durham, United States, ^10^GlaxoSmithKline, Research Triangle Park, United States, ^11^Gilead Sciences, Foster City, United States, ^12^National Institute of Allergy and Infectious Diseases, Division of AIDS, Rockville, United States, ^13^University of North Carolina at Chapel Hill, Division of Infectious Disease, Chapel Hill, United States, ^14^UNC Project‐Malawi, Lilongwe, Malawi

**Background: **HPTN 084 found that long‐acting cabotegravir (CAB‐LA) was well‐tolerated and significantly reduced the risk of HIV acquisition in women compared to tenofovir disoproxil fumarate/emtricitabine (TDF/FTC). During the blinded phase of the trial, participants were required to use long‐acting reversible contraceptives. A nested hormonal contraceptive sub‐study assessed potential pharmacologic interactions between PrEP agents (CAB‐LA or TDF/FTC) and etonogestrel (ETO), medroxyprogesterone acetate (MPA) or norethindrone (NOR).[Fig jia226351-fig-0020]

**Figure 1 jia226351-fig-0020:**
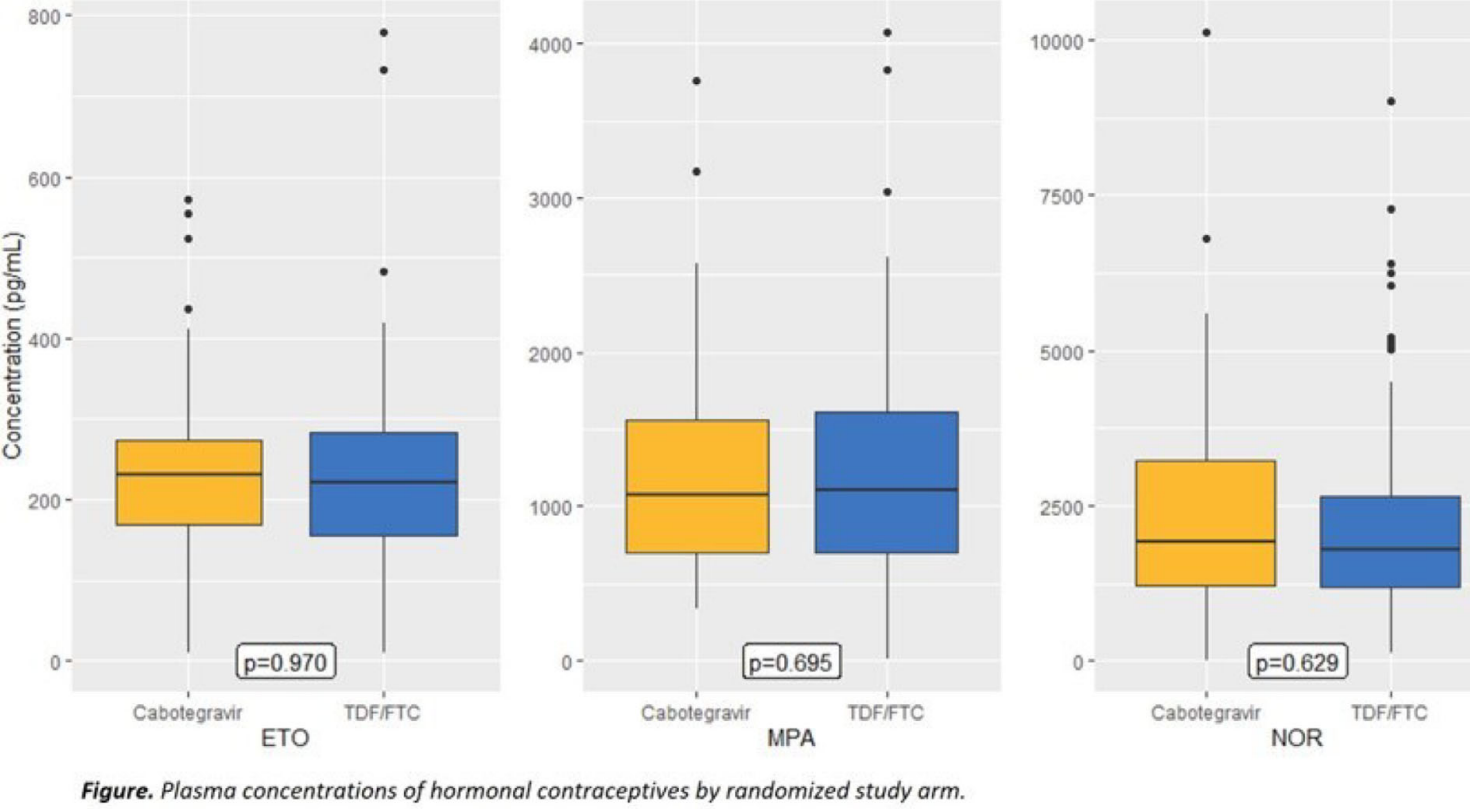
**OA2306**

**Methods: **Participants were enrolled into the sub‐study between 24 Feb 2020 and 26 Oct 2020. Based on the reported contraceptive regimen at baseline and subsequent study visits, plasma concentrations of ETO, MPA and NOR were evaluated at enrolment and weeks 25, 49 and 73; plasma tenofovir (TFV) and CAB concentrations were determined at contemporaneous visits. Participants were allowed to switch contraceptives and pharmacokinetic (PK) assessments were adjusted accordingly. Geometric mean concentrations were calculated and compared using *t* tests or Fisher's exact tests.

**Results: **One hundred and ninety participants consented to the sub‐study; 170 provided samples beyond the enrolment visit (CAB‐LA: 80; TDF/FTC: 90). Changes in contraceptive regimens were common across both study arms (CAB‐LA: 23.8%; TDF/FTC: 24.4%). Post‐enrolment contraceptive concentrations were comparable between study arms for all three contraceptive types (Figure). The percentage of participants with concentrations above thresholds associated with ovulation suppression (≥90 pg/ml ETO, ≥100 pg/ml MPA and ≥1000 pg/ml NOR) was high and did not differ between arms (*p*‐value range: 0.423−1.00). The time from last contraceptive dose until assessment (95% CI) ranged from 316 to 544 (ETO), 50 to 68 (MPA) and 41 to 54 (NOR) days. CAB concentrations were comparable across contraceptive types. TFV concentrations were unquantifiable for most participants, irrespective of contraceptive agent, rendering comparisons largely uninformative.

**Conclusions: **Interactions between CAB‐LA and ETO, MPA, and NOR were not observed. Associations between TDF/FTC and hormone concentrations could not be effectively evaluated due to low adherence to TDF/FTC.

### OA2402 PrEP choice for women in Africa: Uptake of oral PrEP and PrEP ring

E. Irungu ^1^, M. Conlon^2^, R. Soothoane^3^, K. K'Orimba^4^, N. Naidoo^5^, C. Akello^6^, E. Gwavava^7^, P. Jeckonia^4^, I. Mahaka^7^, M. Etukiot^8^, R. Wafula^9^, R. Phate‐Lesihla^10^, H. Subedar^11^, H. Kadama^12^, R. Wilcher^2^, A. Mayo^2^, K. Torjesen^2^

^1^Jhpiego, Nairobi, Kenya, ^2^FHI 360, Durham, NC, United States, ^3^Jhpiego, Maseru, Lesotho, ^4^LVCT Health, Nairobi, Kenya, ^5^Wits RHI, University of Witwatersrand, Johannesburg, South Africa, ^6^FHI 360, Kampala, Uganda, ^7^Pangaea Zimbabwe, Harare, Zimbabwe, ^8^TASO Uganda, Kampala, Uganda, ^9^Kenya Ministry of Health, Nairobi, Kenya, ^10^Lesotho Ministry of Health, Maseru, Lesotho, ^11^National Department of Health, Pretoria, South Africa, ^12^Uganda Ministry of Health, Kampala, Uganda

**Background: **Understanding uptake of oral PrEP and PrEP ring when women are offered choice is needed to guide the expansion of PrEP programmes in Africa.

**Methods: **The PEPFAR/USAID‐supported CATALYST study aims to characterize an enhanced service delivery package for informed PrEP choice for women at public health sites in five African countries. Individuals aged ≥18 years, including those pregnant and/or breastfeeding in permitting countries, are eligible to choose between PrEP ring and oral PrEP. We describe PrEP uptake among those offered PrEP choice between May and December 2023, explore factors associated with method choice among PrEP‐naïve individuals using logistic regression and describe reasons for choices made.

**Results: **Of 2383 participants eligible for PrEP choice, 978 (41%) were ≤24 years, 709 (30%) reported sex work, 84 (4%) were pregnant, 243 (10%) were breastfeeding and 1526 (64%) were PrEP‐naïve. At enrolment, 1464 (61%), 842 (35%) and 77 (3%) participants chose oral PrEP, PrEP ring and no method, respectively. PrEP ring uptake among pregnant and breastfeeding women where allowed was 14 (17%) and 49 (20%), respectively.

Among PrEP‐naïve individuals eligible for PrEP choice, those reporting sex work, having condomless sex in the past month and having multiple sex partners in the last 3 months were more likely to choose PrEP ring (Table 1). In a multivariable analysis that adjusted for country, no factors remained significantly associated with PrEP ring uptake. In permitting countries, pregnant and breastfeeding women were less likely to choose PrEP ring.

Participants chose oral PrEP because it is easy to use (50%) and works well (29%), while PrEP ring was chosen because it is easy to use (56%) and does not require swallowing pills (53%).

**Conclusions: **We demonstrate moderate uptake of PrEP ring when offered within existing real‐world oral PrEP programmes. Our findings inform the implementation of PrEP choice in the region.[Fig jia226351-fig-0021]

**Figure 1 jia226351-fig-0021:**
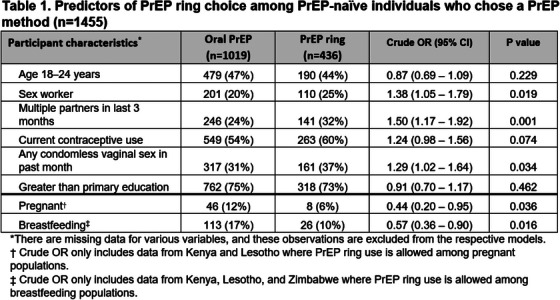
**OA2402**

### OA2403 Provider and client perspectives on PrEP choice: quality of PrEP choice and factors influencing its provision in the CATALYST implementation study

M. Kiruki ^1^, H. Tzindoli^1^, M. Niyibeshaho^1^, M. Chabela^2^, M. Nkongoane^2^, O. Maboa^3^, M. Pleaner^3^, R. Adams^3^, I. Segawa^4^, A. Mijumbi^5^, J. Kabongo^6^, C. McGuire^7^, K. Louis‐Charles^7^, H. Sisel^7^, V. Fonner^7^

^1^LVCT Health, Nairobi, Kenya, ^2^Jhpiego, Maseru, Lesotho, ^3^Wits RHI, Johannesburg, South Africa, ^4^FHI 360, Kampala, Uganda, ^5^The AIDS Support Organization (TASO), Kampala, Uganda, ^6^Pangaea Zimbabwe, Harare, Zimbabwe, ^7^FHI 360, Durham, North Carolina, United States

**Background: **Women in sub‐Saharan Africa bear a disproportionate HIV burden, highlighting the need for new PrEP technologies. Limited evidence exists on the delivery of PrEP choice. This mixed methods study describes early experience with PrEP choice implementation for women in the PEPFAR/USAID‐supported CATALYST study.

**Methods: **We analysed enrolment cohort survey data (May–December 2023) across public health sites in Kenya, Lesotho, South Africa, Uganda and Zimbabwe. We adapted a PrEP method information index (PrEP MII) to ascertain the quality of provision of PrEP choice counselling using three indicators: informed about multiple methods (oral PrEP and PrEP ring), side effects and possibility of switching methods. Logistic regression assessed variations in receipt of PrEP choice counselling across population subsets. We thematically analysed 27 in‐depth interviews with providers (October 2023–January 2024) across 10 sites to explore barriers, facilitators and strategies to improve PrEP choice.

**Results: **Among cohort participants, 87% received quality PrEP choice counselling upon enrolment (*N* = 2548; PrEP MII scores = 3/3). Rates varied by country, with Lesotho and Uganda scoring higher (96% and 95%), Zimbabwe (85%), and Kenya and South Africa scoring lower (80%). PrEP MII scores differed across populations. Pregnant and breastfeeding populations had lower odds of receiving PrEP choice (AOR = 0.65, 95% CI [0.44, 0.96], *p* = 0.032; AOR = 0.49, 95% CI [0.36, 0.68], *p* = <0.001, respectively) as did those with prior PrEP experience (use within 30 days of enrolment) (AOR = 0.74, 95% CI [0.55, 0.99], *p* = 0.041) compared to PrEP‐naive individuals. Providers emphasized the comparative benefits of choice counselling to address clients’ HIV prevention needs despite it being more time‐consuming than counselling for oral PrEP alone. Overall, providers were positive towards offering PrEP choice, overcoming initial implementation reluctance and challenges. Providers proposed strategies to boost motivation for offering PrEP choice, including more training, incentives, sufficient staffing and tailored counselling. Additionally, they emphasized the importance of expanding community outreach and fostering opportunities for cross‐learning.

**Conclusions: **Despite challenges, providers offered most participants key elements of PrEP choice counselling. However, providers require enhanced support to offer PrEP choice to pregnant and breastfeeding individuals and those with prior oral PrEP experience. PrEP implementation should address identified barriers and facilitators to enhance PrEP choice access.

### OA2404 Contraceptive method mix and PrEP choice among young women seeking contraception at retail pharmacies in Kenya

B. Rono ^1^, F. Mogaka^1^, B. Nyerere^1^, G. Rota^1^, E. Harrington^2^, H. Lagat^2^, J. Odoyo^1^, K.F. Ortblad^3^, M. Lenn^2^, M. Mugambi^2^, M. Sharma^2^, V. Omollo^1^, Z. Kwena^1^, E. Bukusi^1,4^, J. Pintye^5^

^1^Kenya Medical Research Institute, Centre for Microbiology Research, Kisumu, Kenya, ^2^University of Washington, Department of Global Health, Seattle, United States, ^3^Fred Hutchinson Cancer Center, Seattle, United States, ^4^University of Washington, Department of Global Health, Seattle, United States, ^5^University of Washington, Department of Nursing, Seattle, United States

**Background: **Modern contraceptives choice and use among adolescent girls and young women (AGYW) remain a challenge. Understanding such preferences and HIV PrEP methods could inform co‐delivery for this group. A differentiated approach where pharmacy delivery models are considered is key to improving access.

**Methods: **We analysed baseline data on contraception method mix and PrEP choices among AGYW enrolled in an ongoing cluster randomized trial assessing pharmacy‐based PrEP delivery models at 20 retail pharmacies in Kenya (NCT05467306). HIV‐negative AGYW (15−24 years) purchasing contraception (emergency contraception [EC], oral contraceptive pills, injectables, implants, condoms) offered oral PrEP or dapivirine vaginal ring (DPV‐VR) per national guidelines. AGYW who accepted PrEP received a 1‐month supply. We obtained socio‐demographic characteristics, contraception preference and sexual behaviour data using structured questionnaires. We compared association between HIV PrEP option and contraceptive preference using adjusted Poisson regression model.

**Results: **Between May 2023 and March 2024, 1003 AGYW were enrolled: median age 21 years (IQR19−23); 19% (156/1003) were ≤18 years. Most AGYW (84%, 713/1003) were unmarried, condomless sex was common (88%, 765/1003) and 46% (461/1003) had a prior pregnancy. About 21% (211/1003) of AGYW reported ≥1 sexual partner, 9% (90/1003) reported sex while intoxicated in the last 6 months and 10% (100/1003) reported transactional sex. A quarter (23%, 231/1003) perceived themselves at moderate/high HIV risk. Among AGYW dispensed PrEP (*n* = 864), 664 (77%) chose oral PrEP. EC was the most common contraceptive (55%, 475/864) purchased, followed by injectables (19%, 164/864), pills (13%, 112/864), condoms alone (11%, 95/864) and implants (2%, 17/864); 71% (613/864) of AGYW reported prior EC use. EC purchase was less frequent among AGYW who chose DPV‐VR compared to those choosing oral PrEP (53% vs. 63%, *p* = 0.019); no other differences in contraceptive method mix were detected (all *p*>0.05): injectables (20% vs. 16%), pills (12% vs. 14%), condoms alone (12% vs. 8%) and implants (2% vs. 0%). Self‐selecting DPV‐VR was not associated with purchase of long‐acting (injectable or implant) contraception (aRR = 0.95, CI: 0.91−1.01).

**Conclusions: **We found varying preferences for short‐ and long‐acting methods for contraception and PrEP among AGYW in Kenya, suggesting that providing multiple HIV and pregnancy prevention options at pharmacies may increase coverage.

### OA2405 Switching between oral and injectable pre‐exposure prophylaxis (PrEP) regimens in the United States: An investigation of reasons for switching in the real world

L. Tao ^1^, J. Yang^1^, D. Mezzio^1^, C. Nguyen^1^, W. Zachry^1^, J. Gruber^1^

^1^Gilead Sciences, Inc., Foster City, California, United States

**Background: **Real world factors influencing individuals’ switching patterns between daily oral and every‐2‐month injectable PrEP regimens for HIV‐1 prevention are unclear.

**Methods: **HIV‐1‐negative adults receiving ≥1 dispensed prescription of oral PrEP between 1 January and 31 December 2021, were identified from the IQVIA LAAD pharmacy claims database. Switching between injectable cabotegravir (CAB) and oral F/TAF or F/TDF was monitored after 1 January 2022. Key determinants driving PrEP switches were identified by a classification tree model and ordered by relative importance. Logistic regression was used to evaluate the likelihood of switching PrEP modalities.

**Results: **Of 173,572 oral PrEP users, 2.6% switched to CAB; key switching determinants included education attainment, HIV‐1 risk behaviours and oral regimens used (Figure 1A). Individuals using Medicaid had increased odds of switching to CAB versus individuals with commercial insurance (1.84 [1.69–2.00]) (Figure 1B). Individuals with HIV‐1 risk behaviours also had increased odds of switching to CAB (2.12 [1.96–2.30]); however, individuals using F/TDF only had decreased odds of switching versus F/TAF only users (0.25 [0.20–0.32]) (Figure 1B).

Of 5721 CAB users (new initiation and switched from oral), 26.1% switched or switched back to oral PrEP; key switching determinants included being PrEP‐naïve prior to CAB initiation, having previous abandoned/rejected injectable claims and insurance type (Figure 1A). People with increased odds of switching to oral PrEP had abandoned/rejected CAB claims (2.68 [2.37–3.03]) or lived in Southern versus Western states (1.62 [1.38–1.90]) (Figure 1B). Approximately 67% of all CAB users missed ≥1 injection window; among these users, 5% subsequently filled new oral PrEP claims.

**Conclusions: **PrEP switches were driven by identified individual characteristics and formulary coverage factors. Results underscore the need to develop interventions that can address the diverse requirements of individuals who would benefit from PrEP and provide oral bridging, to improve adherence and reduce HIV‐1 acquisition.[Fig jia226351-fig-0022]

**Figure 1 jia226351-fig-0022:**
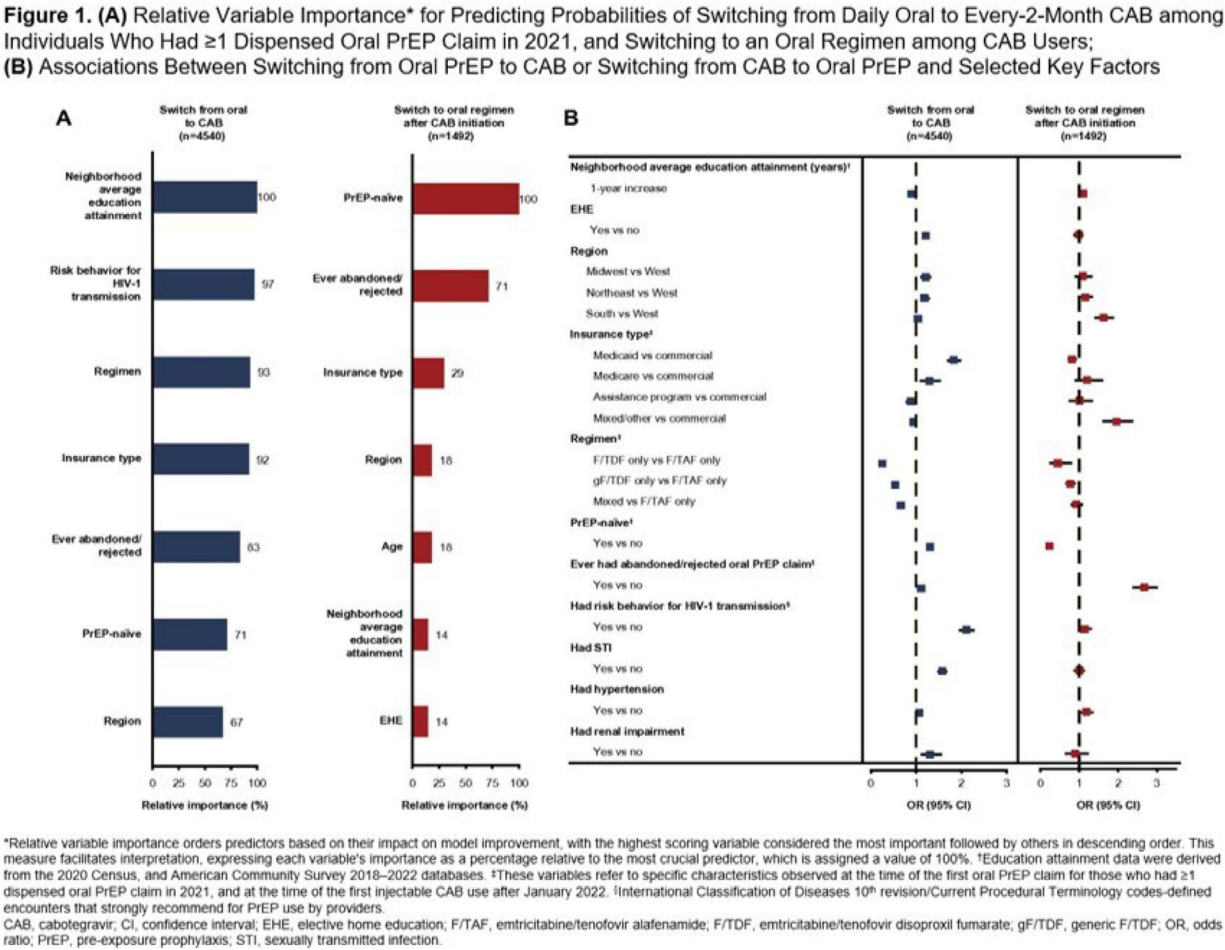
**OA2405**

### OA2406 PrEP method (oral or ring) choice in a community‐based programme: Findings from the DREAMS PrEP choice study in South Africa

N.P. Naidoo ^1^, N. Mthimkhulu^1^, G. Chidumwa^1^, B. Chabalala^1^, C. Fakude^1^, N. Jama^1^, P. Kunene^1^, T. Mhakakora^1^, L. Parmley^2^, S. Mullick^1^

^1^Wits RHI, University of Witwatersrand, Johannesburg, South Africa, ^2^United States Agency for International Development (USAID), South Africa, Johannesburg, South Africa

**Background: **Dapivirine vaginal ring (PrEP ring) was approved in 2022 as an additional PrEP method in South Africa. Understanding choice and uptake will be important parameters to guide countrywide introduction and scale up. This abstract presents uptake and factors associated with PrEP method choice in communities.

**Methods: ​**DREAMS PrEP Choice is an implementation science study offering oral PrEP and PrEP ring to women 18 years and older through a community‐based programme utilizing mobile clinics and pop‐up gazebos in 36 sites across Johannesburg. Socio‐demographic, behavioural and routine clinical data were extracted. Logistic regression using STATA version 18 was conducted to describe PrEP choice at enrolment and factors associated with uptake, adjusting for age *a priori*.

**Results: **Between October 2023 and March 2024, 709 clients were screened for study inclusion, with 87% meeting eligibility. Of 614 participants who consented, 589 (96%) chose a method. Seventy‐three percent (430/589) were between 18 and 24 years; and 84% completed secondary or tertiary education. Of those reporting a current sexual partner(s) (*n* = 517), 66% did not know their partner's HIV status and 5% reported more than one sexual partner. Seventy‐one percent chose oral PrEP, 28% PrEP ring and 1% selecting no method, noting that 21% were former oral PrEP users. Participants aged 25−34 years (aOR = 1.68, 95% CI: 1.07−2.63, *p* = 0.0001); and reporting recent transactional sex (aOR = 3.43, 95% CI: 1.09−10.74, *p* = 0.0001) had higher odds of choosing PrEP ring at enrolment compared to oral PrEP. PrEP ring uptake differed by delivery site with higher learning institutions (aOR =  2.44, 95% CI: 1.18−5.05, *p* = 0.001) and community safe spaces (aOR =  2.38, 95% CI: 1.14−4.99, *p* = 0.001) having greater odds of choosing PrEP ring. PrEP‐naïve participants had lower odds of choosing PrEP ring (OR = 0.50, 95% CI: 0.32−0.76, *p* = 0.001). We found no association between method choice and past pregnancy, contraceptive use or implementation model (mobile or gazebo).

**Conclusions: **Our findings demonstrate moderate PrEP ring uptake with participants 25−34 years, reporting transactional sex, tertiary education, and found at community safe spaces and tertiary institutions more likely to choose PrEP ring. Understanding who is likely to take up PrEP ring, and characteristics that drive choice can inform how programmes are designed and delivered at scale.

### OA2502 Appropriateness of a social influence campaign to de‐stigmatize, de‐medicalize and generate demand for PrEP among young women and female sex workers in South Africa: A qualitative study

B. Wong ^1^, J.G. Rosen^1^, M. Mcingana^2^, J. Mcloughlin^3^, L. Shipp^4^, C. Singh^5^, N. Matenjwa^6^, N. Dladla^6^, J. Steingo^2^, S. Baral^4^, H. Hausler^2,7^, S. Schwartz^4^

^1^Johns Hopkins Bloomberg School of Public Health, Department of International Health, Baltimore, United States, ^2^TB HIV Care, Cape Town, South Africa, ^3^TB HIV Care, Priority Populations Prevention Programme, uMgungundlovu, South Africa, ^4^Johns Hopkins Bloomberg School of Public Health, Department of Epidemiology, Baltimore, United States, ^5^TB HIV Care, uMgungundlovu, South Africa, ^6^TB HIV Care, Durban, South Africa, ^7^University of Pretoria, Department of Family Medicine, Pretoria, South Africa

**Background: **Despite growing PrEP availability, intersectional HIV and PrEP stigma has demotivated PrEP uptake and persistence among female sex workers (FSWs) and adolescent girls and young women (AGYW) in South Africa. We examined the appropriateness of Le Kip Kip, a multi‐component social influence campaign implemented to de‐stigmatize and de‐medicalize PrEP—among FSW, AGYW and their communities in South Africa.

**Methods: **We conducted in‐depth interviews with Le Kip Kip campaign implementers (venue‐based peer PrEP champions and volunteer community mobilizers; *n* = 30) and community members with potential campaign exposure (*n* = 36) in uMgungundlovu, Zululand and Ngaka Modiri Molema Districts. We used deductive thematic analysis to evaluate campaign appropriateness across five domains: attractiveness, comprehension, relevance, acceptability and credibility.

**Results: **PrEP de‐medicalization via rebranding as Le Kip Kip was attractive to implementers and community members. Implementers noted that community members approached them with curiosity when wearing campaign‐branded merchandise, availing opportunities to disseminate PrEP information. Attention‐grabbing marketing materials alongside social media pages enhanced campaign credibility, building community trust in the campaign and PrEP via informational content and consistent branding. Comprehension was high, with most community members demonstrating a correct understanding of PrEP; implementers noted that after clarifying the meaning of campaign materials, community members conceptualized PrEP and Le Kip Kip synonymously. Community members emphasized the campaign's relevance in their community, noting that PrEP knowledge and acceptability were suboptimal before the campaign but improved with campaign exposure. Campaign implementers felt validated by their communities for their labour.

**Conclusions: **De‐medicalization was perceived as appropriate and acceptable in this South African campaign, with attention‐grabbing branding effectively disseminating destigmatizing, empowering PrEP messaging. Community members reported positive improvements in PrEP perceptions, reaffirming the promise of Le Kip Kip to cultivate a more enabling environment for PrEP uptake. Demand‐generation for daily and long‐acting PrEP formulations in South Africa may benefit from de‐medicalization and messaging around empowerment.[Fig jia226351-fig-0023]

**Figure 1 jia226351-fig-0023:**
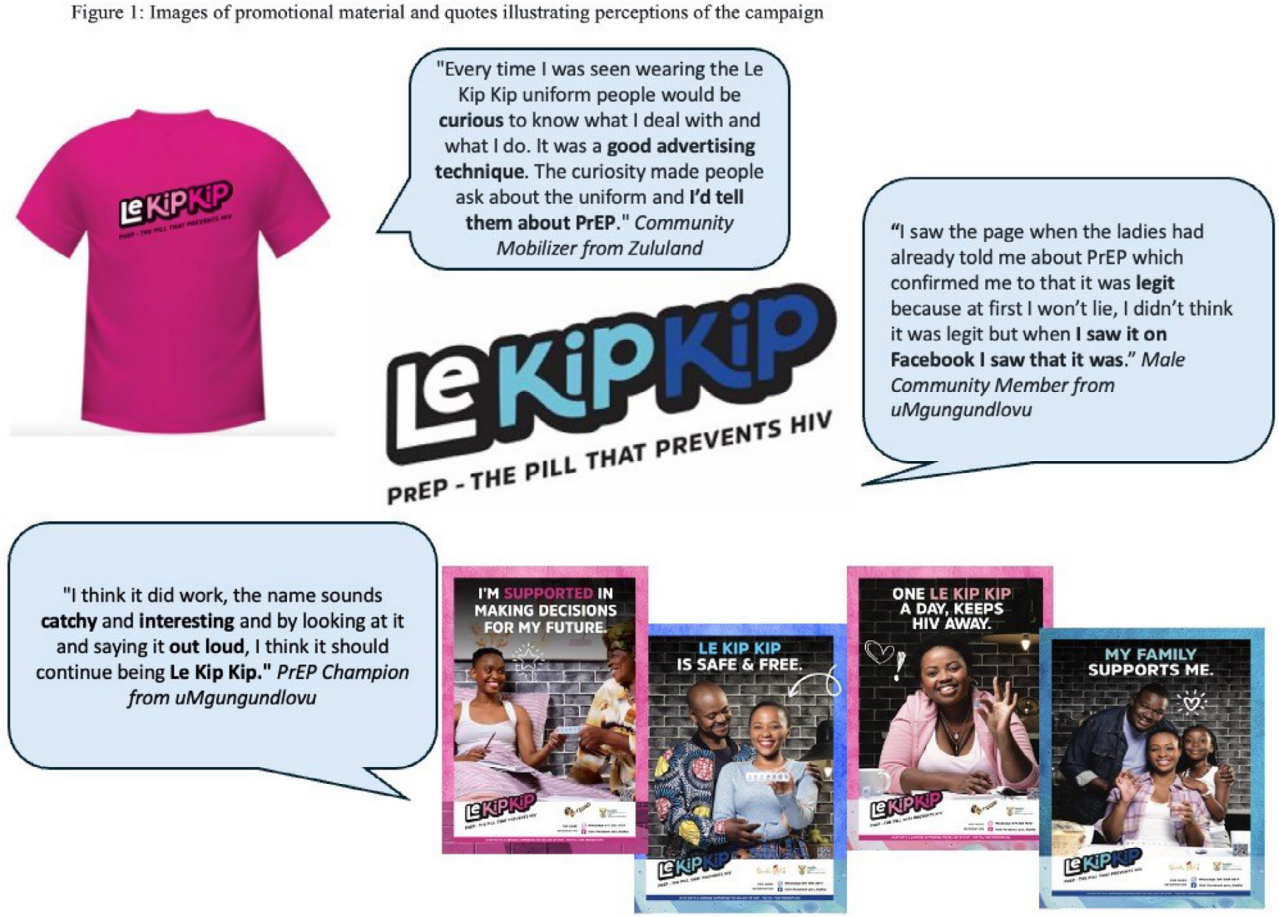
**OA2502**

### OA2503 Science simplified—co‐creating games with key communities to communicate HIV germline targeting vaccine design concepts in India and South Africa

K. Goyal ^1^, S. Ul Hadi^2^, J. Mukherjee^2^, P. Saha^2^, K. Seth^2^

^1^DCT Mindlinks (OPC) Pvt, Ltd., New Delhi, India, ^2^IAVI, Gurugram, India

**Background: **The onset of experimental medicine vaccine design trials (EMVT) warrants an increasing need to enhance literacy towards the scientific rationale and purpose of new approaches like: germline targeting, mRNA vaccines and complex boosting schedules. Towards this, we co‐created game‐based tools for community engagement inspired by theories from behavioural sciences, social psychology and gamification in working with community stakeholders.

**Methods: **Ten consultations with over 30 scientists, community representatives and design experts across India and South Africa helped identify appropriate, accurate and relevant messages around EMVT including:

- Iterative process of germline targeting: step‐by‐step creation of immunogen in the lab (as recombinant protein or mRNA) and corresponding antibody response in the body
- B‐cell maturation process in response to the sequential immunogens

Inspired by Kolb's experiential learning cycle, a puzzle‐based competitive building‐block game was created to understand the scientific rationale behind EMVT (Figure 1). The game was finetuned and finalized through iterative prototyping with community representatives.

A training‐of‐trainers was then conducted on the game implementation with 12 Community Liaison officers from India and South Africa.

**Results: **Interactive community feedback sessions helped identify ways to enhance intuitiveness and simplicity of gameplay while maintaining scientific accuracy through relatable metaphors including:

- Lock‐and‐key mechanism for immunogen and antibody interaction,
- Incremental complexity of process to showcase b‐cell maturation.

Participant feedback from the training indicated:

- A “definite” and “significant” enhancement of understanding and capabilities in implementing socio‐behavioural research studies by 75% and 25%, respectively.
- For scientific concepts of germline targeting and mRNA vaccine, exposure to training moved the average score on a 10‐point familiarity scale to positive side by 50−55%.

**Conclusions: **Co‐creating interactive gamified experiences with communities aided in enhancing a sense of collaboration and ownership in the research process. Simplifying the science through games helped participants better engage and understand the scientific rationale for research, increasing their motivation for participation.[Fig jia226351-fig-0024]

**Figure 1 jia226351-fig-0024:**
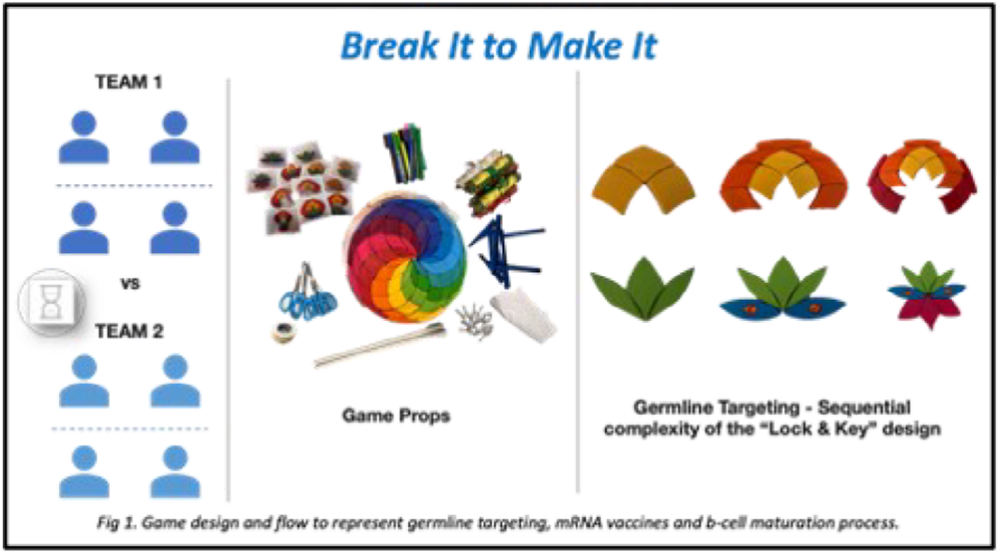
**OA2503**

### OA2504 Using local languages for accurate science reporting in Media Science Cafés in East and Southern Africa

E. Nakkazi ^1^, I. Jooste^2^, C. Mwita^3^, K. Marshall^4^, A. Katumba^4^, M. Chatani‐Gada^4^

^1^Health Journalists Network in Uganda, Kampala, Uganda, ^2^Internews, Durban, South Africa, ^3^Internews, Nairobi, Kenya, ^4^AVAC, New York, United States

**Background: **The Media Science Café Program in Kenya, Malawi, Tanzania, Uganda, Zambia and Zimbabwe fosters an environment conducive to accurate reporting on HIV science by engaging journalists through interactive media science cafés. The programme promotes accurate reporting on HIV science by engaging journalists in both English and the local languages in which many journalists report. These sessions, hosted by national health journalism associations, facilitate knowledge exchange among scientists, civil society organizations and other experts, and journalists to unpack science and research and development.

**Methods: **All Media Science Café Programs promote accurate reporting on science and health by engaging journalists through interactive café sessions conducted in both English and local languages. Utilizing local languages at Media Science Cafés empowers journalists to convey stories with greater precision. This approach benefits both scientists and journalists by facilitating clear communication and enhancing the understanding of scientific concepts among diverse participants in a language they are most familiar with.

A translation database (https://avac.org/translation‐index/) containing scientific and health terminology in local languages has been developed with input from café journalists and their communities, further aiding in the selection and packaging of content for wider dissemination. This approach promotes consistency in comprehension and fosters trust among participants.

**Results: **Key lessons learned include the simplification of scientific terminologies used during regular media science café discussions, enabling journalists to convey complex research outcomes more accurately to their audiences. Furthermore, utilizing local languages helps cultivate confidence between experts, journalists and the community by ensuring mutual understanding and reducing the risk of miscommunication or misrepresentation.

**Conclusions: **Insights gained from utilizing local languages at media science cafés in diverse countries have facilitated the creation of a comprehensive translation database covering scientific and health terms. This resource pinpoints areas requiring further translation efforts, thereby enhancing the effectiveness and accuracy of science reporting for a diverse audience. The database is also a tool for research literacy, supporting dissemination of scientific and health information across various linguistic communities. The use of local languages during these engagements plays a pivotal role in enhancing comprehension of scientific concepts and assists journalists in articulating stories effectively for broader audiences.

### OA2505 Lessons learned from engaging community stakeholders in product development for a proposed novel HIV intervention in early‐phase clinical development

K. Chapman ^1^, A. Van der Westhuizen^2^, K. Richards^3^, L. Sam‐Urom^4^

^1^IAVI, New York, United States, ^2^IAVI, Cape Town, South Africa, ^3^IAVI, Johannesburg, South Africa, ^4^IAVI, Abuja, Nigeria

**Background: **Good Participatory Practice (GPP) guidelines, among others, form the basis for community engagement when conducting HIV prevention trials, but engagement is generally initiated and focused on the trials including protocol and trial site preparation. In early 2023, after a broad consultation on clinical trial design for HIV bnAbs identified earlier and more robust community engagement as a significant need, a community engagement working group (CEWG) was formed to promote and strengthen early involvement and to develop materials to increase community awareness of bnAbs for infant HIV prophylaxis (PNP) prior to trial site selection. This abstract highlights key lessons from CEWG efforts to strengthen community‐driven engagement in early‐phase clinical development.

**Methods: **The CEWG formed in June 2023 to address this gap in early engagement by (1) convening different community groups from different countries with varying HIV contexts, (2) mobilizing these community stakeholders to develop materials for future direct engagement, (3) harmonizing community‐level feedback on the product development (PD) pathway prior to development of trial protocols and (4) creating a space for community voices on PNP. This strategy supported early awareness and education in communities, which can lead to products and trials better tailored to their target populations.

**Results: **Varying baseline levels of knowledge and participation were a challenge, highlighting a need for increased research literacy at a generalized community level and resources for capacity strengthening. For meaningful engagement with community stakeholders, funding and support need to be mobilized for community stakeholder engagement outside of clinical trials and a strategy for engagement throughout product development needs to be communicated and integrated into GPP plans. This model of early engagement demonstrates how the research community can rethink community engagement and the benefits to the end‐to‐end development for products.

**Conclusions: **These early activities bring attention to the importance of community‐driven strategies for meaningful engagement across each step of the bnAb development process. Creating a strong foundation for collective dialogue across different areas of expertise requires significant preparation and ongoing engagement throughout the PD process to support informed trial participation, future introduction and acceptability.

### OA2506 Long‐acting HIV technologies access in LMICs: Stakeholder engagement to understand concerns of the community

J.W. Mwangi ^1^, B. Nzano^2^

^1^Afrocab Treatment Access Partnership, HIV, Malindi, Kenya, ^2^Clinton Health Access Initiative, Clinical Sciences Team, Kampala, Uganda

**Background: **HIV treatment and prevention therapies have predominantly been pursued through oral antiretroviral drugs for decades. Significantly, treatment has evolved from complex multi‐pill regimens to more efficacious once‐daily fixed‐dose combinations. However, social stigma and high pill burden persist among other challenges. The novel technology in long‐acting injectables like cabotegravir (CAB‐LA) and lenacapavir (LEN), therefore, herald a new era. Yet, little has been asked of the community to identify any concerns prior to their introduction. To address this, Afrocab Treatment Access Partnership organized two community forums in Kampala and Nairobi in June 2022 and May 2023.

**Methods: **The forums brought together advocates and representatives from Ministries of Health, the WHO, academia, implementers and donors. CAB‐LA and LEN's product profiles were introduced to prompt facilitated stakeholder dialogue.

**Results: **The crucial voice and role of community in product introduction (from development to policy discussions to introduction and scale‐up) ensures a comprehensive and inclusive approach. Furthermore, key concerns below emerged:

1. Voluntary Licenses: The urgent need to issue voluntary licenses for both products, and for effective technology transfer to expedite generic development.
2. Affordable Pricing: Can sustained collaboration be ensured between donors and manufacturers towards an affordable CAB‐LA/LEN price point, ensuring access for low‐ and middle‐income countries (LMICs)?
3. Transparency in Regulatory Processes: Regulatory authorities and normative bodies (WHO PQ, US FDA, national regulatory authorities) and their commitment to increased transparency on review timelines for both products.
4. Treatment Literacy: Continuous education and sensitization efforts were emphasized to bridge knowledge gaps among stakeholders, disseminating information about product specifics, licensing and regulatory timelines.
5. Community‐Led Decision‐Making: Value addition of community‐led decision‐making throughout the lifecycle of long‐acting HIV products. Dialogue between communities and stakeholders empowers affected populations to timely shape policies, programmes and access strategies.

**Conclusions: **The forums laid a robust foundation for continued community engagement ahead of LMIC entry of long‐acting HIV products. A collective commitment to collaboration, transparency and community‐centric approaches will foster a more effective and inclusive global response to HIV in this new era of long‐acting injectables.

### OA2602 Cryo‐EM characterization of diverse antibody interactions with HIV envelope to facilitate vaccine and therapeutic antibody development

N.C. Morano ^1^, H. Wang^2^, S. Pletnev^2^, R.S. Roark^1^, S. Wang^2^, J. Gorman^3^, J. Xu^4^, T. Zhou^2^, P.D. Kwong^1^, L. Shapiro^1^

^1^Columbia University Vagelos College of Physicians and Surgeons, Aaron Diamond AIDS Research Center, New York, United States, ^2^National Institute of Allergy and Infectious Diseases, Vaccine Research Center, Bethesda, United States, ^3^Food and Drug Administration, Division of Viral Products, Silver Spring, United States, ^4^Georgia State University, Department of Biology, Atlanta, United States

**Background: **In the last few years, advances in cryo‐EM data collection and data processing technology have allowed for rapid structural annotation of antibody interactions with HIV‐1 envelope. Such data inform the development of a wide array of interventions at various stages of progress.

**Methods: **HIV‐1 envelope complexes with antigen‐binding fragment of antibody or with nanobody were prepared at a concentration of 3−4 mg/ml, with 0.005% DDM, and vitrified on copper holey grids in liquid ethane using a Vitrobot Mark IV. Cryo‐EM data were collected on a Titan Krios operating at 300 keV, equipped with a K3 detector (Gatan) operating in counting mode. Data were acquired using Leginon. For all structures, cryo‐EM data was processed using CryoSPARC, and models were generated using Coot, Phenix and UCSF Chimera.

**Results: **Here, we highlight single particle cryo‐EM contributions through four HIV‐focused vignettes. First, we determined antibody‐envelope trimer structures in a proof‐of‐concept study to induce high serum‐neutralizing titres of over 50% breadth in macaques at the fusion peptide‐site of vulnerability by a combination of fusion peptide vaccination and SHIV infection. Cryo‐EM structure determinations revealed 15 of 16 isolated antibodies with cross‐clade breadth to be directed towards the fusion peptide‐site of vulnerability. Second, we used cryo‐EM to characterize antibodies elicited in humans by a phase I clinical trial of the “DS‐SOSIP”‐stabilized envelope trimer from strain BG505. In specific, we showed how vaccine‐elicited fusion peptide‐directed antibodies may be highly strain specific. Third, we determined cryo‐EM structures of PGDM1400 (currently in clinical trials for the treatment of HIV‐1) and an improved variant of the clonal relative PGT145 bound to BG505 envelope trimers; these structures revealed how different clades of a single antibody lineage can adopt different strategies for broad recognition at the V2 apex‐site of vulnerability. Fourth, we determined the structure of vaccine‐elicited nanobody R27 linked to the light chain of the V2 apex‐targeting broadly neutralizing antibody, CAP256V2LS. This bispecific chimera exhibited ultrapotent neutralization and breadth greater than other published HIV‐1 broadly neutralizing antibodies.

**Conclusions: **Collectively, these vignettes demonstrate how structural biology will continue to accelerate the development of vaccines and therapeutics against HIV‐1.

### OA2603 Elicitation of HIV‐1 cross‐neutralizing serum IgG and monoclonal antibodies following sequential immunization of NFL Env trimers into non‐human primates

R.T. Wyatt ^1^, S. Bale^1^, F. Schleich^2^, J. Guenaga^1^, G. Ozorowski^3^, M. Àdori^2^, R. Wilson^1^, J. Cluff^1^, T. Dzvelaia^1^, W. Li^1^, E. Doyle^1^, X. Lin^3^, M. Cochran^2^, I. Wilson^3^, D. Carnathan^4^, G. Silvestri^4^, A.B. Ward^3^, G.B. Karlsson Hedestam^2^

^1^The Scripps Research Institute, Department of Immunology and Microbiology, La Jolla, United States, ^2^Karolinska Institute, Department of Microbiology, Tumor and Cell Biology, Stockholm, Sweden, ^3^The Scripps Research Institute, Department of Integrative Structural and Computational Biology, La Jolla, United States, ^4^Emory University, Department of Medicine, Atlanta, United States

**Background: ​**The elicitation of cross‐neutralizing antibodies towards broadly neutralizing determinants on the HIV‐1 envelope glycoprotein (Env) remains a major vaccine challenge. Most cross‐conserved sites on the trimeric HIV spike are occluded by host‐encoded “self” N‐glycans, limiting B‐cell recognition of the underlying protein surface. Exceptions are the Env primary receptor CD4‐binding site (CD4bs) and the furin cleavage site. We have pursued N‐glycan deletion to better expose one or both Env regions for enhanced B‐cell priming in pre‐clinical studies. Initially, we eliminated N‐glycans proximal to the CD4bs while maintaining the native‐like state of cleavage‐independent (NFL) trimers. Following glycan‐deleted priming, we used heterologous boosting coupled with N‐glycan restoration to preferentially drive B cells directed to conserved sites. This strategy elicited two “tier 2” broadly neutralizing antibodies (bNAbs); E70, a CD4bs‐directed, N‐glycan‐dependent cross‐neutralizing mAb and, 1C2, an 87% bNAb directed to the gp41‐gp120 interface both structurally resolved by cryoEM.

**Methods: **In an ongoing experiment in non‐human primates (NHPs), we immunized subjects with CD4bs glycan‐deleted Env trimers in SMNP adjuvant followed by sequential heterologous trimer boosting coupled with glycan restoration to elicit cross‐neutralizing antibodies.

**Results: **We elicited HIV‐1 “tier 2” cross‐neutralizing serum IgG activity in a subset of NHPs that maps proximal to the CD4bs and the Env furin cleavage site by EMPEM (EM polyclonal epitope mapping). To “close the loop,” we isolated over 200 monoclonal antibodies from memory B cells, several of which cross‐neutralize multiple HIV clinical isolates in a 90‐member global panel. The most potent/broad antibody lineage displays somatic hypermutation levels exceeding 20% from germline and maps proximal to the CD4bs as determined initially by cross‐competition. By negative stain EM, these heavily somatically mutated antibodies interact with the gp120:gp120 interface spanning adjacent Env trimer protomers. High‐resolution cryoEM structures of two representatives from this broadly neutralizing lineage reveal a new cross‐neutralizing epitope bridging between the CD4bs and the gp120 interface of the adjacent protomer. We are performing similar monoclonal antibody analysis on other NHPs exhibiting cross‐neutralization present in their serum IgG.

**Conclusions: **Glycan deletion and heterologous Env trimer prime:boosting elicits HIV‐1 bNAbs in NHPs and we are beginning to test this concept in humans.

### OA2604 Structure of a neutralizing VRC01‐class antibody elicited after prime‐boost germline‐targeting immunization regimen

M. McGovern ^1^, P. Agrawal^1^, G. Kher^1^, L. Stamatatos^1,2^, M. Pancera^1^

^1^Fred Hutchinson Cancer Center, Vaccine and Infectious Disease Division, Seattle, United States, ^2^University of Washington, Department of Global Health, Seattle, United States

**Background: **∼20% of people living with HIV‐1 can develop broadly neutralizing antibodies (bnAbs) against the envelope glycoprotein (Env) present at the surface of the virus. These bnAbs have been shown to be protective in animal models and the VRC01 bnAb prevented HIV‐1 acquisition by susceptible viruses. VRC01‐class antibodies have been isolated from multiple donors, target the conserved CD4‐binding site and share common gene characteristics, including extensive somatic hypermutation from their germline precursors. VRC01‐class antibodies are difficult to elicit by immunizations.

**Methods: **Here, we employed a germline targeting immunization approach that consisted of using a prime immunogen designed to engage germline VRC01‐like B cells and subsequent boosts of more native Env immunogens to guide the maturation of VRC01‐class B‐cell precursors. We isolated an antibody, G3‐1, after immunizations in VRC01‐class knock‐in mice, that neutralizes the autologous virus and 25% of the heterologous tier 2 viruses panel (eight viruses) tested indicating it can bypass a major steric hindrance of VRC01‐class precursors. We obtained cryoEM structures of G3‐1 bound to WT 426c DS.SOSIP and of VRC01 bound to the same SOSIP.

**Results: **G3‐1 and VRC01 approach the SOSIP with slightly different angles. G3‐1, unlike VRC01, has no deletions in the CDRL1 and is still able to accommodate the N276 glycan and neutralize the virus. G3‐1 also induces conformational changes in Env upon binding, similar to CD4, in part due to a rare phenylalanine mutation that mimics Phe43 of CD4.

**Conclusions: **The structure of the G3‐1 bound to WT 426c DS.SOSIP can be used to determine why different somatic hypermutations are necessary to become broadly neutralizing.

### OA2605 Structural characterization of a vaccine‐elicited NHP broadly neutralizing antibody lineage reveals a new quaternary neutralizing epitope on HIV Env

J. Guenaga ^1^, S. Bale^1^, F. Schleich^2^, G. Ozorowski^3^, M. Adori^2^, R. Wilson^4^, J. Cluff^1^, X. Lin^3^, M. Corcoran^2^, I. Wilson^3^, D. Carnathan^5^, G. Silvestri^5^, A. Ward^3^, G. Karlsson Hedestam^2^, R. Wyatt^1^

^1^Scripps Research, Immunology and Microbiology, La Jolla, United States, ^2^Karolinska Institute, Microbiology, Tumor and Cell Biology, Stockholm, Sweden, ^3^Scripps Research, Integrative Structural and Computational Biology, La Jolla, United States, ^4^Scripps Research, Immunology and Microbiology, San Diego, United States, ^5^Emory University, Medicine, Atlanta, United States

**Background: **Immunization of non‐human primates with stabilized single‐chain NFL trimers in a heterologous prime‐boosting soluble protein regimen resulted in the elicitation of broad serum antibody responses in NHPs. By single‐cell sorting, we isolated multiple antibodies that neutralized HIV‐1 in a TZM‐bl assay. Four antibodies belonging to the same lineage displayed broad neutralization of HIV‐1 in an 80 pseudo‐virus panel with neutralizing breadth exceeding 50% of the viruses tested (IC50).

**Methods: **Cross‐competition mapping by Octet suggests that a lineage representative antibody competed with VRC01 for binding to Env. By cryo‐EM, we obtained a high‐resolution structure of two antibodies (LJF‐0034 and LJF‐0085) in complex with a stabilized JR‐FL NFL.664 trimer.

**Results: **The structure revealed a novel epitope with an extensive footprint on two adjacent protomeric Env units at the gp120 interface. The antibody light chain exclusively contacts the CD4‐binding site of one protomer with most contacts being nearly 100% conserved. In contrast, the antibody heavy chain targets the V2V3 trimerization domain of the adjacent protomer where residues are less conserved.

**Conclusions: **Ongoing structural analysis of the antibody contacts in conjunction with sequence analysis of lineage‐resistant viruses identifies specific residues in V3 that may explain escape from members of this broadly neutralizing lineage. These findings will help to redefine the immunization regimen to increase HIV neutralization coverage and more efficiently elicit these types of antibodies in future immunization studies.

### OA2606 Recognition determinants of improved HIV‐1 neutralization by a heavy chain matured paediatric antibody

S. Singh ^1^, S. Kumar^1,2^, A. Chatterjee^3^, P. Bajpai^4^, S. Sharma^1^, S. Katpara^1^, H. Bhakhri^1^, R. Lodha^5^, S. Dutta^3^, K. Luthra^1^

^1^All India Institute of Medical Sciences, Biochemistry, New Delhi, India, ^2^International Center for Genetic Engineering and Biotechnology, ICGEB‐Emory Vaccine Center, New Delhi, India, ^3^Indian Institute of Science, Molecular Biophysics Unit, Bangalore, India, ^4^International Center for Genetic Engineering and Biotechnology, ICGEB‐Emory Vaccine Center, New Delhi, India, ^5^All India Institute of Medical Sciences, Pediatrics, New Delhi, India

**Background: **HIV‐1 bnAbs offer a promising template for structure‐guided vaccine design, targeting highly potent therapeutic and preventive strategies against HIV‐1. We observe rapid development of bnAbs in HIV‐infected infants, contrasting with delayed response in adults, suggesting distinct maturation pathways in children. Despite extensive characterization in adults, paediatric bnAbs lack comprehensive study. Our research focuses on a paediatric bnAb from antiretroviral‐naive, chronically infected elite donors (EN), showing superior breadth and potency compared to the parental antibody.

**Methods: **We synthesized and extensively characterized a matured heavy chain lineage antibody known as 44m, originating from paediatric EN. 44m was isolated through longitudinal bulk BCR analysis of elite paediatric neutralizers, utilizing NGS. The heavy chain genes of 44m were codon‐optimized for improved expression in mammalian systems. Antibody characteristics were assessed using IMGT/V‐QUEST and ARMADiLLO. Binding reactivity and kinetics to BG505 gp140 trimer were confirmed through ELISA and Octet BLI. The neutralization potential against diverse HIV‐1 viruses was evaluated at varying concentrations via TZM‐bl assay. Structural insights, particularly in complex with BG505 gp40 trimer, were obtained through single‐particle Cryo‐EM.

**Results: **Our study addresses the knowledge gap in the evolving HIV‐1 bnAb lineage in chronically infected children. The study revealed the functional importance of the 44m heavy chain for HIV‐1 envelope binding and neutralization, with increased heterologous breadth when paired with the AIIMS‐P01 light chain. Notably, the 44m antibody showed improved breadth (79%) and potency (geometric mean IC50 titre of 0.36 mg/ml) against heterologous viruses compared to AIIMS‐P01. Testing against a global virus panel demonstrated a 58% breadth with an IC50 titre of 0.43 mg/ml. ARMADiLLO analysis identified four improbable amino acid mutations in 44m with less than a 2% frequency. Additionally, high‐resolution structure analysis at 4.4 A° resolution highlighted the specificity of the paediatric HIV‐1 bnAb 44m for the N332 supersite and the GDIR motif.

**Conclusions: **In conclusion, our study highlights key factors boosting HIV‐1 neutralization by the paediatric antibody 44m within the AIIMS‐P01 lineage. Factors such as recognition determinants heightened somatic hypermutation, and precise target sites enhanced 44m breadth and potency. These findings contribute to the potential of highly effective bnAb‐based strategies for HIV‐1 treatment and prevention.

### OA0207 Global racial, ethnic, and gender diversity among participants enrolled in the PURPOSE‐2 trial of lenacapavir for pre‐exposure prophylaxis (PrEP)

J. Gallardo‐Cartagena ^1^, N. Phanuphak^2^, N. Ndlovu^3^, M.H. Losso^4^, M. Clement^5^, V.D.C. Lucio^6^, B. Grinsztejn^7^, K.H. Mayer^8^, Y. Zhao^9^, L. Brown^9^, C. Carter^9^, M. Das^9^, O. Ogbuagu^10^

^1^Centro de Investigaciones Tecnológicas, Biomédicas y Medioambientales, Universidad Nacional Mayor de San Marcos, Lima, Peru, ^2^Institute of HIV Research and Innovation (IHRI), Bangkok, Thailand, ^3^Wits Reproductive Heath and HIV Institute, University of the Witwatersrand, Johannesburg, South Africa, ^4^Hospital General De Agudos J M Ramos Mejía and Fundacion IBIS/CICAL, Buenos Aires, Argentina, ^5^Louisiana State University Health Sciences Center, New Orleans, United States, ^6^Emory University School of Medicine, Atlanta, United States, ^7^Instituto Nacional de Infectologia Evandro Chagas/Fundação Oswaldo Cruz (Fiocruz), Rio de Janeiro, Brazil, ^8^Harvard Medical School, Harvard University, and The Fenway Institute, Fenway Health, Boston, United States, ^9^Gilead Sciences, Inc., Foster City, CA, United States, ^10^Yale School of Medicine, New Haven, CT, United States

**Background**: Despite World Health Organization recommendations to make pre‐exposure prophylaxis (PrEP) available to all people with increased likelihood of HIV‐1 acquisition, disparities in new HIV‐1 infections and PrEP use persist globally. People of color and gender‐diverse people (transgender women, transgender men, and gender non‐binary individuals) experience disproportionate HIV‐1 incidence and have been historically underrepresented in PrEP clinical trials. These disparities are magnified in low‐ and middle‐income countries and the Global South.

**Methods**: The PURPOSE‐2 trial (NCT04925752) is evaluating the comparative efficacy and safety of long‐acting (twice yearly) subcutaneous injectable lenacapavir for HIV‐1 PrEP among cisgender men, transgender women, transgender men, and gender non‐binary individuals who have condomless receptive anal sex with partners assigned male at birth. Trial sites in locations with relatively high prevalence of HIV‐1 were selected. Consultative engagement with stakeholders and community advocates at these sites was initiated during the planning stages and continued throughout study enrollment to intentionally recruit a globally, racially, ethnically, and gender diverse participant population.

**Results**: A total of 3272 participants were randomized from seven countries across the United States, Latin America, South Africa, and Asia (Figure). Mean age of participants was 30 (range, 17–74) years, and 22.2% were gender diverse (including 14.7% transgender women, 1.3% transgender men, and 6.2% gender non‐binary individuals). The majority of participants were non‐White (67.6%), including 35.7% Black or of Black ancestry and 12.6% Asian; 62.7% were of Hispanic/Latinx ethnicity.[Fig jia226351-fig-0028]

**Figure 1 jia226351-fig-0028:**
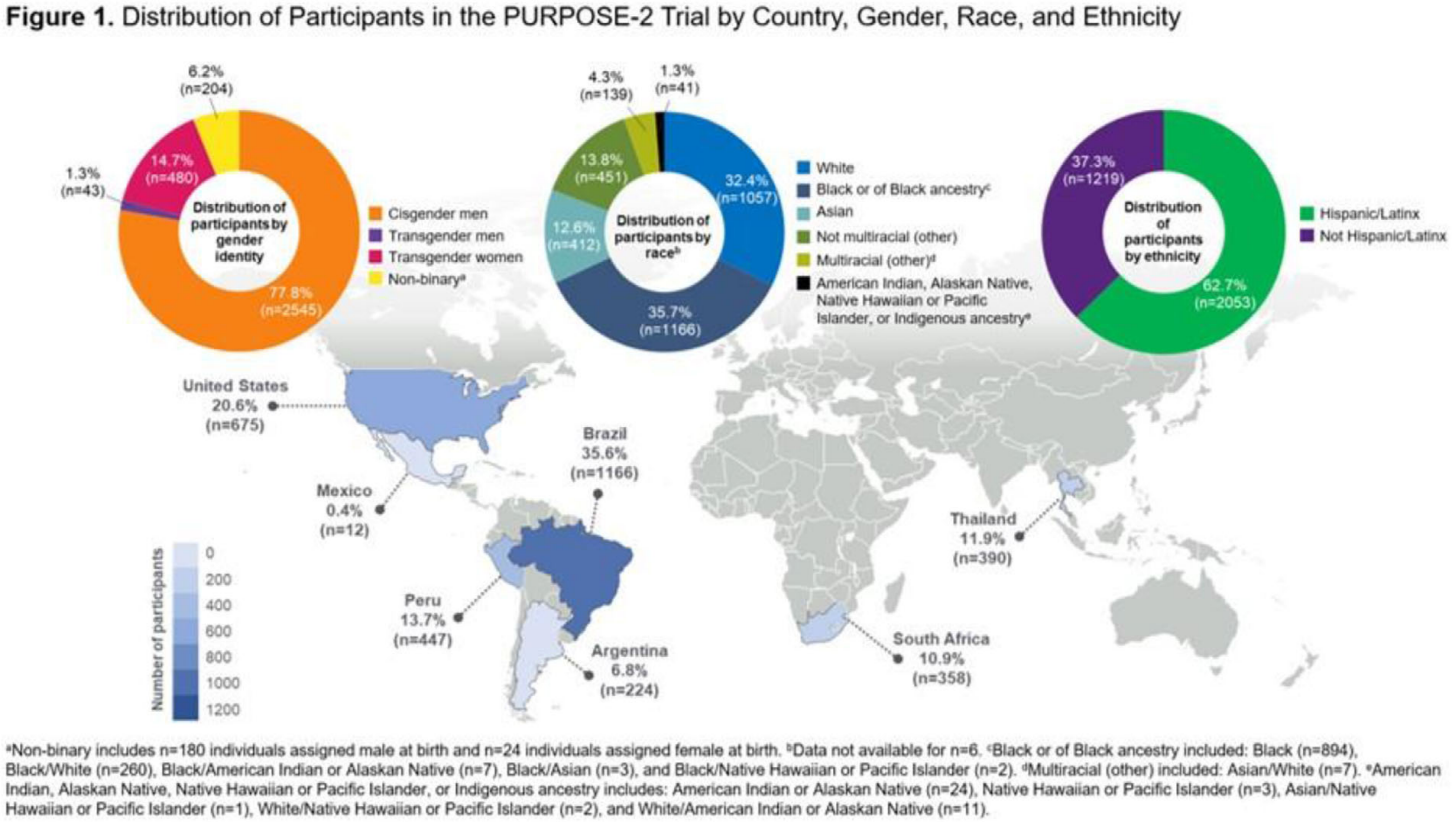
**OA0207**

**Conclusions**: Purposeful recruitment led to a global PrEP trial with a significant majority of racially, ethnically, and gender diverse participants from settings including low‐ and middle‐income countries and communities highly vulnerable to HIV‐1. Inclusion of these diverse participant populations will be important for understanding the efficacy of lenacapavir for PrEP in communities disproportionately affected by HIV‐1 and who could benefit from PrEP.

## LATE BREAKING ABSTRACTS

### OA0107LB HIV trends in metropolitan U.S. cities from 2014 to 2022: Baseline for the U.S. ending the HIV epidemic (EHE) initiative

R. Gupta ^1,2^, R. Bindlish^3^, S. Vermund^2^

^1^University of Minnesota Medical School, Minneapolis, United States, ^2^Yale School of Public Health, New Haven, United States, ^3^University of Minnesota, Minneapolis, United States

**Background: **The EHE initiative aims to decrease HIV incidence by 90% by 2030 in the 57 counties/states responsible for half the incident infections. EHE was announced in 2019 and phase I nears completion, our analysis provides a counterfactual comparator for future evaluation.[Fig jia226351-fig-0025]

**Methods: **We used 2014−2022 Metropolitan Statistical Area (MSA) data to compare trends in HIV incidence rate between MSAs subsuming EHE regions (*N* = 46) and other MSAs (*N* = 73). A difference‐in‐difference analysis illustrated potential early‐stage programme effects.

**Results: **Across 119 MSAs from 2014 to 2022, 384,793 HIV cases were diagnosed with a mean change in MSA‐level incidence of −4.94% (range: −55.14% to 333.3%) over the period. On average, MSA‐level incidence changed by −19.70% (range: −55.14% to 19.30%) among MSAs including EHE regions and by 4.35% (range: −59.1% to 333.33%) in other MSAs over the study. A difference‐in‐difference analysis indicated the incidence change among EHE MSAs was 2.07 cases per 100,000 people‐years (*p* = 0.03) higher than the base change rate in 2020−2022. These findings were robust to timeframe variations.[Table jia226351-tbl-0015]

**Figure 1 jia226351-fig-0025:**
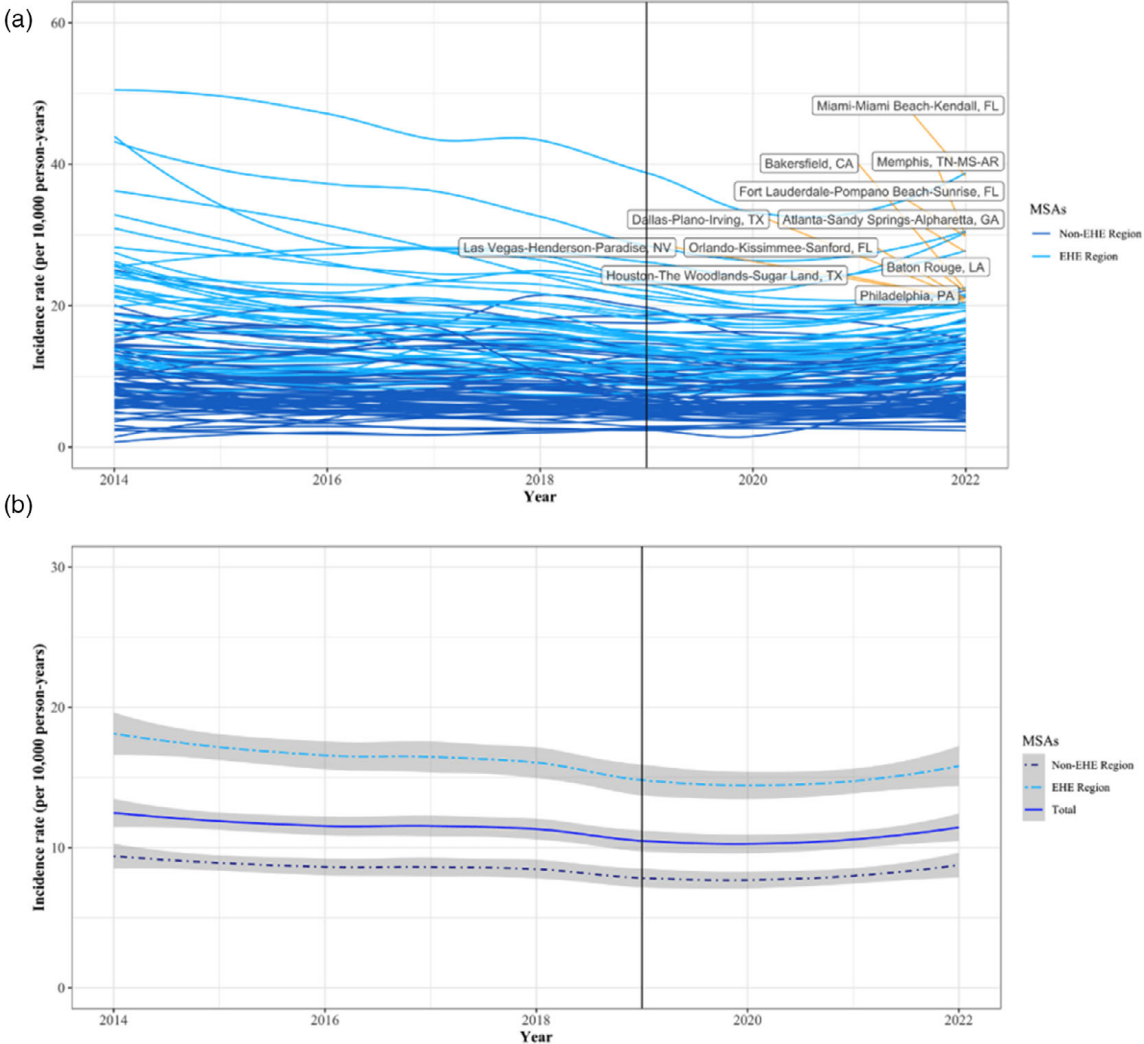
**OA0107** MSA‐level HIV incidence from 2014 through 2022 by EHE classification (Panel A). Mean HIV incidence by EHE classification from 2014 through 2022 (Panel B)

**Conclusions: **Although MSAs including EHE regions experienced greater HIV incidence reductions compared to other MSAs, high inter‐regional variability requires exploration of programme implementation and transmission drivers. These trend insights provide a baseline to aid future assessments of EHE programme impacts.

**Table 1 jia226351-tbl-0015:** OA0107 Summary of difference‐in‐difference (DiD) outcomes

Measure	2014−2022	2015−2022	2016−2022
MSAs not targeted by EHE (*n* = 73) (range)			
HIV incidence (period start)	9.55 (0.6–25.6)	8.52 (1.9–21.1)	8.76 (1.7–21.7)
HIV incidence (period end)	8.65 (2.1–22.2)	8.65 (2.1–22.2)	8.65 (2.1–22.2)
EHE regions MSAs (*n* = 46) (range)			
HIV incidence (period start)	21.22 (8.3–49.9)	21.22 (6.3–51.2)	21.22 (9.1–47.0)
HIV incidence (period end)	16.15 (7.2–38.3)	16.15 (7.2–38.3)	16.15 (7.2–38.3)
Model outcomes (95% CI)			
Baseline annual change to HIV incidence	−**0.73** * (−0.91 to −0.56)	−0.72* (−0.92 to −0.52)	−0.83* (−1.07 to −0.59)
DiD Estimate: difference in annual change to HIV incidence with EHE programme	2.07* **(0.68– 3.46)**	2.05* **(0.73– 3.38)**	2.17* **(0.88– 3.45)**

*Note*: DiD Estimate is for illustration only; we do not expect an impact of EHE programmes in this time period.

### OA0307LB HIV BG505 SOSIP.664 trimer with 3M‐052‐AF/alum induces broad and potent ADCC‐mediating antibodies

D. Mielke ^1,2^, B. Dunn^1^, T. Keyes^1^, S. Stanfield‐Oakley^1^, L. Schmitzberger^3^, D. Schuster^1,2,4^, H. Janes^3^, J. Hural^3^, G. Tomaras^1,2,4,5^, J. McElrath^3^, G. Ferrari^1,2,5^

^1^Duke University, Surgery, Durham, United States, ^2^Duke University, Center for Human Systems Immunology, Durham, United States, ^3^Fred Hutch Cancer Center, Seattle, United States, ^4^Duke University, Integrative Immunobiology, Durham, United States, ^5^Duke University, Molecular Genetics and Microbiology, Durham, United States

**Background: **Adjuvants are known to play a significant role in the quality of immune responses elicited by a vaccine, but direct comparisons of adjuvants with the same immunogen in a clinical trial setting are rare. HVTN 137‐Part B is a phase 1 clinical trial to evaluate the immunogenicity of the HIV‐1 subtype A stabilized trimer, BG505 SOSIP.664 gp140, in combination with different adjuvants: the TLR9 agonist CpG 1018, the TLR7/8 agonist 3M‐052‐AF, both given with Alum, the TLR4 agonist GLA‐LSQ, or Alum alone. The vaccine adjuvanted with 3M‐052‐AF+Alum was able to elicit autologous tier 2 neutralizing antibodies.

**Methods: **We measured antibody‐dependent cellular cytotoxicity (ADCC) responses to understand the impact of different adjuvants using an ADCC Luciferase‐based assay to quantitate the killing of BG505 Envelope‐Infectious Molecular Clone (Env‐IMC)‐infected target cells. We subsequently assessed the breadth of ADCC responses in positive responders using cells infected against a panel of four Env‐IMCs previously reported to have differing levels of susceptibility to ADCC (breadth panel).

**Results: **3M‐052‐AF+Alum‐adjuvanted vaccine elicited robust ADCC responses against BG505‐infected cells in 6/16 and 11/16 participants 2 weeks post second and third dose, respectively, with the average magnitude of responses also increasing among positive responders after the third dose. In comparison, only 1/18 participants in the CpG 1018 group mounted detectable responses 2 weeks post second dose, and no other participants in the other groups had detectable ADCC activity. Additionally, between 2 and 8 of the 11 positive responders in the 3M‐052‐AF+Alum group post third dose were able to mediate ADCC against the different Env‐IMCs in the breadth panel. Interestingly, vaccine‐elicited responses could recognize Envelopes less susceptible to ADCC.

**Conclusions: **3M‐052‐AF+Alum represents a potent adjuvant that can elicit broad ADCC responses in addition to neutralizing responses and represents a strong candidate for use in future HIV vaccine trials.

### OA0507LB Early virologic success on ART following breakthrough infection on CAB‐LA PrEP

U. M. Parikh ^1^, J. Altamirano^2^, H. Safa^3^, A. Hazra^4^, P. Shukla^2^, T. Hedberg^5^, L. Georgetti Gomez^6^, A. L. Heaps^1^, E. K. Halvas^1^, K. Kuncze^6^, H. Okochi^6^, C. Walworth^7^, C. Chu^6^, J. W. Mellors^1^, M. Gandhi^6^, C. A. Koss^6^, SeroPrEP Study Team

^1^University of Pittsburgh, Infectious Diseases, Pittsburgh, United States, ^2^CAN Community Health, Tampa, United States, ^3^Jefferson Health, Philadelphia, United States, ^4^University of Chicago, Chicago, United States, ^5^Howard Brown Health, Chicago, United States, ^6^University of California San Francisco, San Francisco, United States, ^7^Monogram Biosciences/Labcorp of America® Holdings, Burlington, United States

**Background: **Long‐acting cabotegravir (CAB‐LA) PrEP could reduce HIV incidence globally. Breakthrough infections are rare but reported; the clinical consequences of acquiring HIV on CAB‐LA are not well‐defined. We report the first case series of treatment outcomes following HIV acquisition on CAB‐LA PrEP.

**Methods: **SeroPrEP is an ongoing U.S. study examining HIV infection on PrEP. Clinical test results were obtained from participants’ medical records. Serology was confirmed by BioPlex 2200 HIV Ag‐Ab. Mutations in plasma HIV RNA were identified by single‐genome sequencing (SGS) of full‐length *integrase*. CAB concentrations were quantified in plasma and segmented hair via liquid chromatography‐tandem mass spectrometry.

**Results: **Among three SeroPrEP participants who developed breakthrough HIV infection despite on‐time CAB‐LA injections, median time on CAB‐LA was 280 days before the first detectable HIV‐1 RNA. Two participants had negative point‐of‐care antigen/antibody testing with RNA required for HIV detection (PID1: RNA 3940 c/ml; 6 injections; PID2: RNA 4880 c/ml; 5 injections). One participant (PID3) had reactive laboratory‐based antigen/antibody with HIV RNA 2,430,000 c/ml after 13 injections. In PID1, SGS revealed low‐frequency (<5%) INSTI mutations E138K and N155K on separate dates not identified by commercial Sanger. No INSTI mutations were detected in PID2 by Sanger. PID3 had Q148Q/R by Sanger and additional low‐frequency mutations: G140G/S, N155N/H, R263R/K by commercial NGS. CAB was detected in both plasma and hair in all participants. All participants initiated DRV/c/F/TAF; PID1 and PID2 had undetectable viral loads 35 and 47 days post‐ART start, respectively. In PID3, HIV RNA declined from 248,103 c/ml while on DRV/c/F/TAF to 22 c/ml 28 days post‐switch to BIC/F/TAF.

**Conclusions: **Among three individuals in the United States who acquired HIV on CAB‐LA PrEP despite on‐time injections and detectable drug levels, two required RNA for HIV detection. All achieved early virologic success on ART, with two of three on protease inhibitor‐based regimens. One participant with multiple low‐frequency INSTI mutations achieved early viral suppression on BIC/F/TAF. Follow‐up is needed to assess the durability of INSTI‐based ART following breakthrough on CAB‐LA PrEP.

### OA0607LB Phase 1 dose‐escalation trial to evaluate the safety, tolerability, pharmacokinetics and neutralization activity of PGDM1400LS in combination with VRC07‐523LS and PGT121.414.LS in healthy participants without HIV (HVTN 140/HPTN 101)

S. Mahomed ^1^, K. E. Seaton^2^, C. A. Paez^3^, C. Yu^3^, K. Gillespie^3^, S. T. Karuna^3^, T. Gamble^4^, J. Heptinstall^2^, L. Zhang^2^, F. Gao^3^, M. Yacovone^5^, H. Spiegel^6^, J. Dumond^7^, M. Anderson^3^, E. Piwowar‐Manning^8^, B. Dye^4^, I. Tindale^3^, L. Proulx‐Burns^3^, M. Trahey^3^, S. Takuva^3^, A. Takalani^9^, V. C. Bailey^9^, S. Kalams^10^, H. Scott^11^, J. Kosgei^12^, S. Delany‐Moretlwe^13^, S. Kassim^14^, F. Laher^15^, Z. M. Chirenje^16^, Y. Musodza^17^, F. Mhlanga^17^, N. Mkhize^18^, J. Weiner^19^, M. Ackerman^19^, M. J. McElrath^3^, M. Pensiero^5^, L. Gama^20^, D. H Barouch^21^, D. Montefiori^2^, G. D. Tomaras^2^, L. Corey^3^, M. Cohen^7^, Y. Huang^3^, M. Siegel^22^, C. Kelley^23^, HVTN 140/HPTN 101 study team

^1^Centre for the AIDS Programme of Research in South Africa (CAPRISA), Durban, South Africa, ^2^Duke Center for Human Systems Immunology, Departments of Surgery, Integrative Immunobiology, Molecular Genetics and Microbiology, Durham, United States, ^3^Fred Hutchinson Cancer Center, Vaccine and Infectious Disease Division, Seattle, United States, ^4^FHI 360, Durham, United States, ^5^National Institute of Allergy and Infectious Diseases, Rockville, United States, ^6^Kelly Government Solutions, Contractor to NIAID/NIH/HHS, Troy, United States, ^7^University of North Carolina, Chapel Hill, United States, ^8^Johns Hopkins University, Department of Pathology, School of Medicine, Baltimore, United States, ^9^Hutchinson Centre Research Institute of South Africa, Chris Hani Baragwanath Academic Hospital, Johannesburg, South Africa, ^10^Vanderbilt University Medical Center, Division of Infectious Diseases, Department of Medicine, Department of Pathology, Microbiology and Immunology, Nashville, United States, ^11^San Francisco Department of Public Health, San Francisco, United States, ^12^Kenya Medical Research Institute/Walter Reed Project, Kericho, Kenya, ^13^Wits RHI, University of the Witwatersrand, Johannesburg, South Africa, ^14^Desmond Tutu HIV Centre, University of Cape Town, Cape Town, South Africa, ^15^Perinatal HIV Research Unit (PHRU), Soweto, South Africa, ^16^Institute of Global Health, University of California, San Francisco, United States, ^17^University of Zimbabwe Clinical Trials Research Center (UZ‐CTRC), Harare, Zimbabwe, ^18^National Institute for Communicable Diseases, Johannesburg, South Africa, ^19^Dartmouth College, Hanover, United States, ^20^Vaccine Research Centre, National Institute of Allergy and Infectious Diseases, Bethesda, United States, ^21^Center for Virology and Vaccine Research, Beth Israel Deaconess Medical Center, Harvard Medical School, Boston, United States, ^22^George Washington University, School of Medicine and Health Sciences, Washington, United States, ^23^Emory University, Atlanta, United States

**Background: **Passive immunization with broadly neutralizing antibodies (bNAbs) presents a promising HIV prevention modality. Studies suggest that bNAb combinations targeting multiple HIV‐1 epitopes and clades are necessary for effective prevention. HVTN140/HPTN101 evaluated the safety, tolerability, pharmacokinetics and neutralization activity of PGDM1400LS (V2 apex) administered in combination with VRC07‐523LS (CD4 binding site) and PGT121.414.LS (V3 glycan) in healthy adults, without HIV.

**Methods: **The study was a multicentre, randomized, open‐label study conducted in Africa and the United States. After establishing the safety of a single administration of PGDM1400LS in Part A (*n* = 15), Part B (*n* = 80) enrolled adults aged 18−50 years without HIV who received two doses of PGDM1400LS, VRC07‐523LS and PGT121.414.LS 4 months apart. In the five bNAb combination groups, each bNAb was administered at weight‐based doses of 20 or 40 mg/kg intravenously, 20 mg/kg subcutaneously or a fixed dose of 1.4 g either intravenously or subcutaneously. Safety was evaluated through solicited and unsolicited adverse events. Pharmacokinetic parameters were estimated using a two‐compartment population pharmacokinetic model. BNAb serum concentrations were measured by anti‐idiotypic binding antibody assays. Serum neutralization was assessed against viruses sensitive to each of the three bNAbs administered and a panel of recently circulating HIV‐1 strains.

**Results: **Median age was 25.5 years, and 50.5% were assigned female sex at birth. Most participants reported mild‐to‐moderate solicited local and systemic symptoms. The median estimated elimination half‐life of PGDM1400LS was 54 days, not significantly influenced by co‐administration with VRC07‐523LS and PGT121.414.LS. Compared to IV administration, the bioavailability of PGDM1400LS administered subcutaneously was 75.5%. The median estimated elimination half‐life of PGT121.414.LS was 66 days, with a subcutaneous bioavailability of 77.7%. The median estimated elimination half‐life of VRC07‐523LS was 45 days, with a subcutaneous bioavailability of 80.1%. Weight‐based and fixed‐dose regimens showed similar pharmacokinetic patterns. ID80 neutralization titres aligned with predicted values, indicating sustained neutralization activity in vivo, with broad and potent neutralization against both bNAb‐sensitive isolates and recently circulating HIV‐1 strains. No treatment‐induced anti‐drug‐antibody responses were observed.

**Conclusions: **The bNAb combination of PGDM1400LS, PGT121.414.LS and VRC07‐523LS was safe and well‐tolerated, with no pharmacokinetic interactions or loss of complementary neutralization. These findings strongly support the evaluation of this triple combination in future efficacy trials.

### OA0706LB Transcriptional microenvironment of persistent SIV tissue reservoirs is associated with tertiary lymphoid organs in the colon and characterized by stress‐induced decreased protein synthesis

R. Lorenzo‐Redondo ^1,2^, M. S. Arif^3^, C. T. Thuruthiyil^3^, S. S. Pascoe^3^, M. A. Shaaban^1,2^, Y. Thomas^3^, J. M. Hasson^1,2^, S. Samer^3^, M. R. Haque^3^, F. A. Engelmann^3^, M. D. McRaven^3^, M. Araínga^4^, E. Martinelli^1^, F. Villinger^4^, T. J. Hope^3^

^1^Northwestern University Feinberg School of Medicine, Department of Medicine (Infectious Diseases), Chicago, United States, ^2^Center for Pathogen Genomics and Microbial Evolution, Northwestern University Robert J. Havey, MD Institute for Global Health, Chicago, United States, ^3^Northwestern University Feinberg School of Medicine, Cell and Developmental Biology, Chicago, United States, ^4^University of Louisiana at Lafayette, New Iberia Research Center, New Iberia, United States

**Background: **Despite effective antiretroviral therapy (ART), HIV‐1 persistence is the major obstacle to a functional cure. Thus, understanding the tissue microenvironment during ART of the reservoirs that lead to a rapid viral rebound after treatment failure or analytical treatment interruption (ATI) is key.

**Methods: **We have developed immunoPET/CT‐guided spatial transcriptomics, combined with immunofluorescence detection of viral proteins and viral sequencing using the SIV/rhesus macaque model. With this approach, we can find and study foci of viral replication in tissues of all animals. Here, we compare the local neighbourhood of the rebound eclipse‐phase foci (4−6 days post‐ATI) from animals initiating ART 4 days (early‐seeded reservoir with short lifespan) or 10 weeks (well‐seeded reservoir) after high‐dose challenge, as well as early‐seeded tissues from animals on ART for 3 or 7 weeks. Colon tissues containing infected cells foci were identified with a ^64^Copper‐labelled probe against the viral envelope. Subsequently, sections were validated by PCR for SIV DNA, evaluated by immunofluorescence to localize SIV proteins, and characterized by 10x Visium Spatial transcriptomics system.

**Results: **Overall, SIV presence in every condition is associated with higher transcriptional levels and up‐regulation of genes related to SIV infection. Activation of innate immune responses is observed only in the eclipse‐phase of the rebound for both types of reservoirs indicating the specificity of the analysis. Notably, we also detect significant differences between early‐ and well‐seeded reservoirs. Translation activation is associated with SIV presence in early‐seeded but downregulated in well‐seeded reservoirs, while mitochondrial translation is activated in all cases. This is consistent with stress‐induced decreased protein synthesis in well‐seeded reservoirs. Afterwards, we inferred frequencies of cell types per foci by transcriptional. SIV presence after ATI in well‐seeded reservoirs was associated with epithelial cells, IgA plasma cells, monocytes and cycling gamma‐delta T cells, while in early‐seeded reservoir is associated with epithelial cells, IgG plasma cells, Th17 cells and cycling DCs.

**Conclusions: **Our results indicate that persistent reservoirs are associated with tertiary lymphoid organs in the colon and might be characterized by a status of low translation consistent with stress responses. This status could be favouring long‐term viral persistence during ART and rapid rebound of robust viral populations post‐ATI.

### OA0802LB Pharmacokinetic superiority of a 3‐month dapivirine vaginal ring (100 mg) compared to the 1‐month dapivirine vaginal ring (25 mg)

J. Nuttall ^1^, L. Haddad^1^, M. Plagianos^1^, W. Kriel^2^, J. Visser^2^, L. Solai^2^, A. Garg^1^, J. Steytler^2^, B. Devlin^1^

^1^Population Council, Center for Biomedical Research, New York, United States, ^2^IPM South Africa NPC, Johannesburg, South Africa

**Background: **The dapivirine vaginal ring (monthly DVR), currently approved for use in 11 African countries, is an HIV prevention method for women for use over 1 month. An extended‐use option, the 3‐month DVR (3‐month DVR), offers several advantages over the monthly DVR, including reductions in cost, waste and replacement frequency. To bridge the efficacy data for the monthly DVR to the 3‐month ring, we conducted a relative bioavailability study comparing the PK profiles of the two rings.

**Methods: **We conducted a phase I, open‐label, randomized, crossover trial (IPM‐054) to investigate the relative bioavailability of monthly DVR, replaced every 30 days, to 3‐month DVR for 90 days. We randomized healthy female participants to a treatment sequence separated by a 28‐day washout period. Plasma and vaginal fluid samples were collected at multiple time points over the two treatment periods and residual dapivirine levels in used rings were measured. We compared the primary endpoints of plasma dapivirine concentration at Day 90 just prior to ring removal (C_90_) and plasma exposure during the last 30‐day use period (AUC_60‐90_) between the two treatments using mixed effects models with treatment sequence, treatment and period as fixed effects, and participant within the sequence as a random effect. PK parameters were log‐transformed in the model so that the treatment difference provides ratio estimates when back‐transformed. Non‐inferiority for the 3‐month to monthly DVR was predefined as the lower bound of the 90% confidence interval of the ratio (90% CI) exceeding −5% for both primary endpoints with superiority predefined as the lower bound of the 90% CI exceeding 1 for both primary endpoints.

**Results: **One hundred and twenty‐four women (mean age 28.8, range 19−45) were enrolled and randomized, with 104 completing all study visits. Criteria for both non‐inferiority and superiority were met for C_90_ (90% CI of ratio: 1.06; 1.22) and AUC_60‐90_ (90% CI of ratio 1.02; 1.15).

**Conclusions: **This study demonstrated that the 3‐month DVR is pharmacokinetically superior to monthly DVR, suggesting that the efficacy of the 3‐month ring will be at least equal to that of the 1‐month ring. These data support the use of the 3‐month ring as an alternative to the 1‐month ring.

### OA0803LB Acceptability, adherence, preference and safety of placebo long‐acting pre‐exposure prophylaxis (LA‐PrEP) injections and implants for HIV‐prevention in South African men: the SAMURAI study

E. Montgomery ^1,2^, T. Palanee‐Phillips^3,4^, M. Atujuna^5^, C. Hart^1^, K. Reddy^3^, K. Gill^5^, J. Michaels^1^, L.‐G. Bekker^5^, N. Ndlovu^3^, P. MacDonald^3^, C. Chappell^6^, S. Zulu^3^, M. Nomvuyo^5^, G. Mbochi^1^, A. Minnis^1^, The SAMURAI Study Team

^1^RTI International, Women's Global Health Imperative, Berkeley, United States, ^2^University of California, San Francisco, San Francisco, United States, ^3^Wits RHI, Johannesburg, South Africa, ^4^University of Washington, Epidemiology, Seattle, United States, ^5^Desmond Tutu HIV Centre, Cape Town, South Africa, ^6^University of Pittsburgh, Obstetrics, Gynecology and Reproductive Sciences, Pittsburgh, United States

**Background: **Globally and in South Africa (SA), cisgender men underutilize safe and effective HIV‐prevention strategies. Early‐stage research of new prevention modalities with male end‐users offers the opportunity to assess attitudinal, behavioural and safety factors that may influence initiation and persistence with novel drug delivery platforms for long‐acting (LA)‐PrEP.

**Methods: **We conducted a 12‐month crossover study called SAMURAI where men‐who‐have‐sex‐with‐men (MSM) and men‐who‐have‐sex‐with‐women‐only (MSW) in Cape Town and Johannesburg, SA, used placebo versions of bimonthly injections and a 6‐month implant in randomized order. Acceptability, defined as satisfaction with, and future willingness to use, each product, was measured with 10‐point rating scales (10 = high). Adherence was defined as initiation and persistent product use during their respective periods. Adverse events (AEs) and social harms (SHs) were monitored at all visits. LA‐PrEP preference was assessed at the study exit.

**Results: **From July 2022 through June 2024, 184 cisgender men (*n* = 84 MSM, *n* = 100 MSW) were enrolled, with 86% retention. More men initiated injections than implants (97.8% vs. 91.3%, *p* = 0.009); 164 (89%) used both products. 80.6% of men initiating injections were persistent users (35 missed bi‐monthly doses). 91.1% of men initiating implants persisted with use (median 6.0, range 2.3−14.6 months); 15 (9%) requested early removal. Persistent use was significantly higher for implant versus injections (OR 3.11, 95% CI: 1.38, 7.03, *p* = 0.006). The average user‐satisfaction rating was 8.6 (SD 1.8) for injections and 8.4 (SD 1.9) for implants (*p* = 0.44). Likelihood of future use was 8.9 (SD 1.8) for injections and 8.7 (SD 2.3) for implants (*p* = 0.28). Among those who tried both products, injections were preferred by 48.4% and implants by 47.7%. All AEs (200 reported by 102 participants) and serious AEs (4 reported by 4) were unrelated to product use. Four participants (2%) reported study‐related SH of physical or emotional violence, or stigma.

**Conclusions: **South African MSM and MSW were successfully enrolled and followed to use novel placebo LA‐PrEP products. Each delivery form was highly acceptable and equally preferred, reinforcing the importance of PrEP choice, including within LA options. Significant differences between product initiation and persistence suggest that familiarity with delivery form and frequency of clinic dosing visits influenced men's behaviour, with implications for persistence in the use of future LA‐PrEP methods.

### OA0804LB Early implementation of long‐acting injectable cabotegravir for HIV prevention in a safety net primary care centre in U.S. South

D. Baker ^1^, L. Collins^1^, V. Cantos^1^, E. Hollenberg^1^, A. Kaplan^1^, T. Cowan^2^, J. Garcia^2^, M. Lora^1^

^1^Emory University School of Medicine, Atlanta, United States, ^2^Grady Memorial Hospital, Atlanta, United States

**Background: **There is limited data on the implementation of long‐acting injectable cabotegravir (LAI‐CAB) for HIV pre‐exposure prophylaxis (PrEP) in real‐world settings. We describe the early implementation and outcomes of our LAI‐CAB programme.

**Methods: **First, we described the clinical and logistical steps to integrate LAI‐CAB into a primary care‐based oral PrEP programme. Through manual retrospective chart review, we described the socio‐demographic, clinical and social determinants of health (SDOH) of all patients referred to the LAI‐CAB programme from 12/1/2022 to 8/1/2023, and assessed LAI‐CAB PrEP linkage, eligibility, enrolment and initiation. We also assessed reasons for interest or declining LAI‐CAB, total numbers of injections administered, proportion of on‐time injections, HIV seroconversions, self‐reported adverse events and discontinuation.[Fig jia226351-fig-0026], [Fig jia226351-fig-0027]

**Results: **To address known structural barriers to PrEP uptake, we applied a multidisciplinary team‐based approach, streamlined our medication procurement process and HIV testing strategy, and adapted our integrated patient tracking system to minimize late injections. Of all referred individuals, 77 (35%) initiated LAI‐CAB. Their median age was 37 (IQR 29−42), and gender identities were cisgender man (63%), cisgender woman (26%) and transgender woman (8%). Barriers to initiation included: scheduling delays for intake appointments (29%), individuals declining LAI‐CAB (19%) and delays in medication procurement (16%). In total, 275 injections were administered, with 94% of subsequent injections delivered on time. Of patients who initiated LAI‐CAB, six (8%) reported adverse effects and eight (10%) discontinued LAI‐CAB. There were no HIV seroconversions.

**Figure 1 jia226351-fig-0026:**
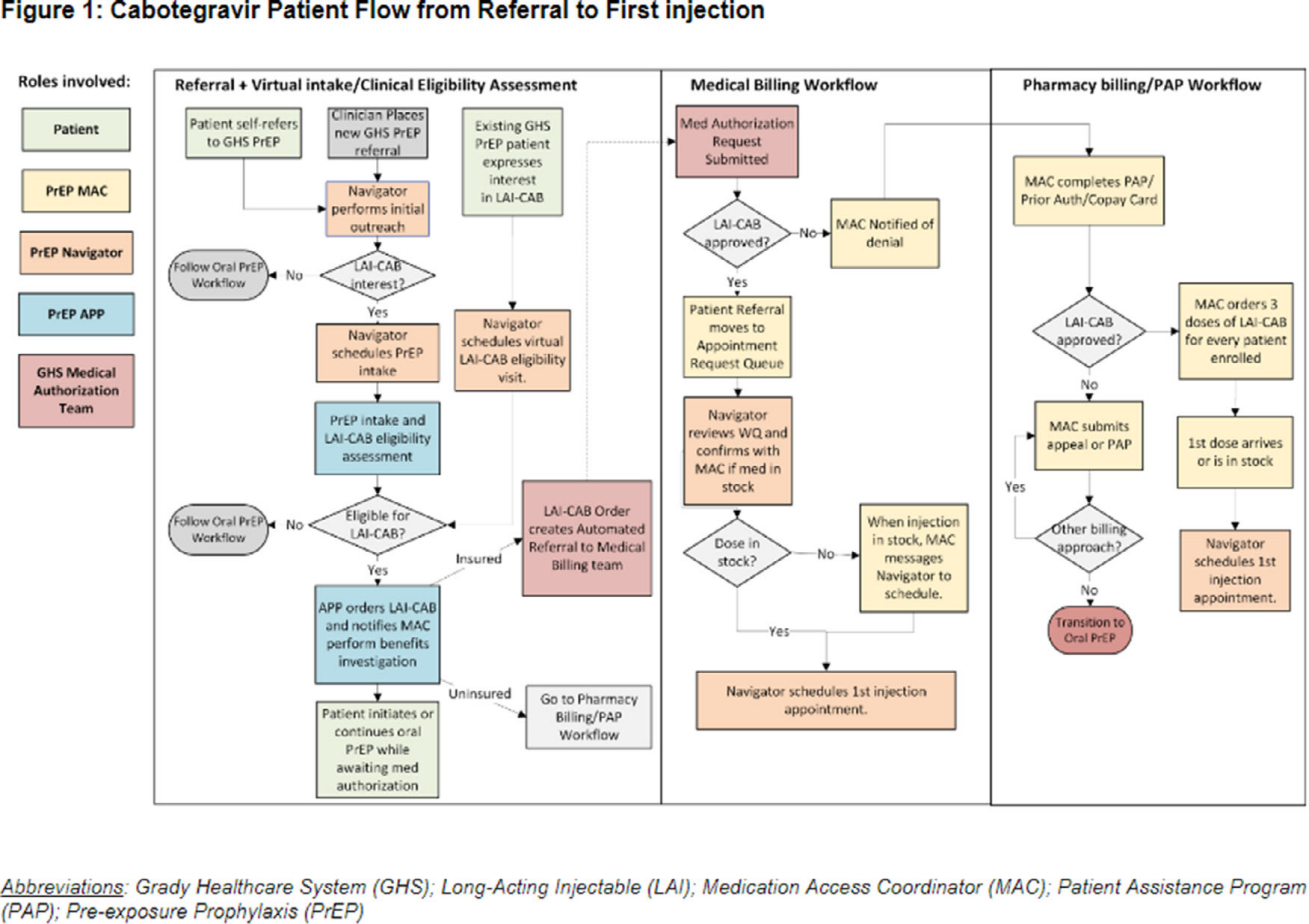
**OA0804**

**Figure 2 jia226351-fig-0027:**
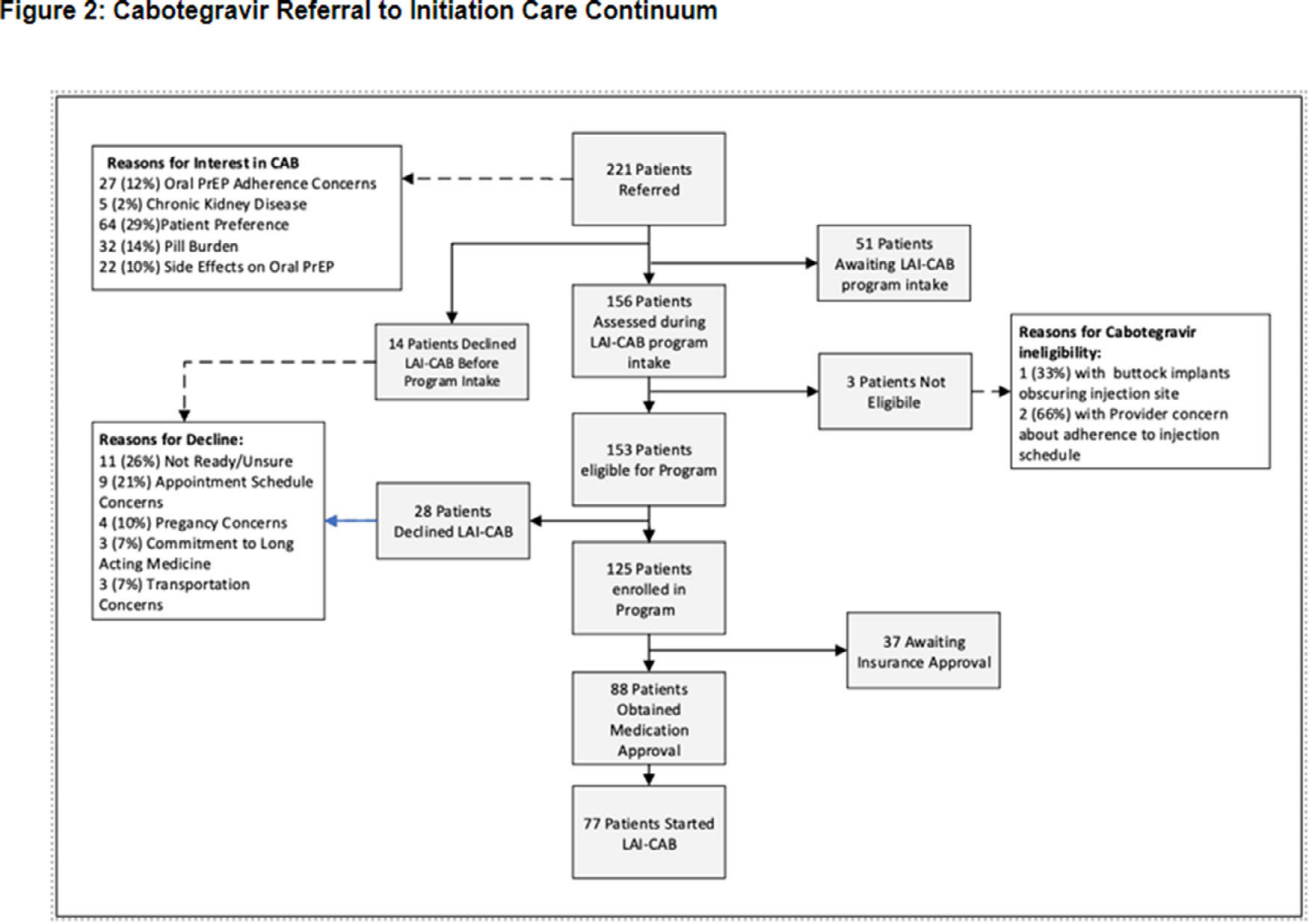
**OA0804**

**Conclusions: **We successfully implemented an LAI‐CAB PrEP programme in a primary care centre designed to address structural barriers to PrEP uptake and persistence.

### OA0805LB Use of HIV rapid detection tests when initiating long‐acting cabotegravir for HIV prevention, within an implementation science project

P. Macdonald ^1^, C. Pike^1^, E. Rousseau^1^, B. Leonard^1^, P. Mapukata^1^, O. Vanto^1^, N. Mathola^1^, L.‐G. Bekker^1^

^1^University of Cape Town, Department of Medicine, Cape Town, South Africa

**Background: **Viral resistance mutations may occur during acute HIV infection (AHI) if long‐acting injectable cabotegravir (CAB‐LA) is initiated concurrently. HIV rapid detection tests (RDTs) may miss AHI, while nucleic acid amplification testing (NAAT) is not feasible in many resource‐constrained settings. Data is needed to quantify the risk of third‐ and fourth‐generation HIV RDT use with CAB‐LA pre‐exposure prophylaxis (PrEP).

**Methods: **HIV RDT results were reviewed for people initiating CAB‐LA in the PrEPared to Choose study, in Cape Town, South Africa, from February to June 2024. A 3‐step HIV screening process was used, which included checking for signs and symptoms of AHI, ruling out HIV exposure in the past 72 hours (requiring postexposure prophylaxis) and a negative HIV RDT at point‐of‐care (POC). ONSITE Ab/Ag (4th generation) RDTs (at a mobile clinic site) and ABON HIV Ab (3rd generation) RDTs (at a government clinic site) were compared to NAAT (HIV RNA‐1 viral load) results from the same day, done at an offsite laboratory. The viral load (VL) result was not known before CAB‐LA initiation.

**Results: **Seven hundred and eighteen people (female 68.0%, [488/718], median age 23 years, IQR 15−29; male 32.0%, [230/718] median age 30 years, IQR 16−59) commenced CAB‐LA following a negative HIV RDT. A 4th Gen HIV RDT was used in 68.1% 489/718 (female 62.2%, 304/489; male 37.8%, 185/489) and a 3rd Gen HIV RDT was used in 31.9% 229/718 (female 80.3%, 184/229; male 19.7%, 45/229). A total of 3/718 (0.4%) had a discrepant detectable VL: 1/489 (0.2%) 4th Gen RDT (>10,000,000 cp/ml) and 2/229 (0.8%) 3rd Gen RDTs (253,469 and 9251 cp/ml). There was no difference observed between those who received 3rd and 4th generation RDTs when stratified by gender, age, prior PrEP use and compared to VL positivity. Participants with detectable VLs were started on tenofovir/lamivudine/dolutegravir treatment, within 5 days of CAB‐LA initiation.

**Conclusions: **Acute HIV infection was not detected by HIV RDTs in 3/718 (0.4%) people who were started on CAB‐LA. Public health benefit must be weighed up against individual risk of same‐day RDT for CAB‐LA PrEP where NAAT testing is not feasible.

### OA0806LB Whole‐genome sequencing of HIV in rural Kwa Zulu Natal does not support the role of age‐disparate disease transmission underpinning the HIV prevalence gender‐gap

J. Hartley^1^, S. Kelly ^2^, S. Kemp^3^, D. Pillay^4^, F. Tanser^5^, O. Ratmann^6^, R. K. Gupta^2^, Vukuzazi Team

^1^Cambridge University Hospitals, Department of Infectious Diseases, Cambridge, United Kingdom, ^2^University of Cambridge, The Cambridge Institute of Therapeutic Immunology and Infectious Disease, Cambridge, United Kingdom, ^3^University of Oxford, Pandemic Sciences Institute, Oxford, United Kingdom, ^4^University College London, Division of Infection and Immunity, London, United Kingdom, ^5^African Health Research Institute, KwaZulu‐Natal, South Africa, ^6^Imperial College London, Department of Mathematics, London, United Kingdom

**Background: **Women aged 15−24 years old carry a disproportionate burden of the new HIV infections diagnosed in Southern Africa. While the causes of this HIV gender‐and‐age are unclear, one key hypothesis argues that age‐disparate relationships with older men drive infection in younger women.

**Methods: **We conducted whole‐genome sequencing of HIV in a large phylogenetic study to model the transmission of the virus between demographic groups in the rural setting of KwaZulu‐Natal, South Africa, utilizing data from the Vukuzazi cohort. Samples with HIV viral loads >50 copies/ml were submitted for whole‐genome sequencing. Adequate sequences were arranged into genomic clusters using a maximum likelihood phylogeny. Potential transmission pairs within clusters were analysed alongside the age and sex data of participants. The distribution of pairings between different age and sex groups was compared to a model in which pairs were drawn from the HIV‐positive population by chance.

**Results: **The Vukuzazi cohort enrolled 18,025 participants, 6069 of whom were HIV positive, and 1232 of whom had viral loads over 50 copies/ml; sequencing of these samples yielded 1097 adequate genomes. Phylogenetic analysis produced 89 clusters containing a total of 205 individuals, and 73 possible linkages between men and women. Only 32 of these 89 potential transmission links (36%) were between men in an older age group than the women. When compared to a model population in which pairings between men and women in different age groups occur randomly within the sample, the number of pairs in all age‐sex combinations was within the 95% confidence interval if pairs were drawn randomly.

**Conclusions: **Our data does not support the assertion that the gender gap in HIV prevalence is driven by older men pairing with younger women. This dogma has been used to influence public health messaging over the last two decades, based on previous research, and may be less true than initially thought. Current work suggests that there likely is a contribution of age‐disparity in transmission, though with significantly smaller age gaps than initially suggested.

### OA0807LB Bayesian methods provide a posterior probability by day for possible dates of HIV acquisition which is useful in clinical trials

R. Rossenkhan ^1^, E. E. Giorgi^1^, D. Shao^1^, J. Ludwig^1^, P. Labuschagne^1,2^, C. A. Magaret^1^, T. Ndung'u^3,4,5,6^, D. Muema^3,4^, K. Gounder^3,4^, K. L. Dong^6^, B. D. Walker^3,6,7^, M. Rolland^8,9^, M. L. Robb^9^, L. A. Eller^8,9^, F. Sawe^10,11^, S. Nitayaphan^12^, E. Grebe^13,14,15^, M. P. Busch^14,15^, K. P. Delaney^16^, S. Facente^17^, L. N. Carpp^1^, A. C. deCamp^1^, Y. Huang^1^, B. Korber^18,19^, M. Juraska^1^, E. Kosmider^1^, D. B. Reeves^1,20^, B. T. Mayer^1^, J. Hural^1^, W. Deng^21^, D. H. Westfall^21^, A. Yssel^22^, T. Bhattacharya^19,23^, D. Matten^22^, L. Corey^24,25,26^, P. B. Gilbert^1,24,27^, C. Williamson^22,28,29^, J. I. Mullins^20,21,26^, P. T. Edlefsen^1^

^1^Fred Hutchinson Cancer Center, Seattle, United States, ^2^South African National Bioinformatics Institute, University of the Western Cape, South African Medical Research Council Bioinformatics Unit, Cape Town, South Africa, ^3^The Doris Duke Medical Research Institute, University of KwaZulu‐Natal, HIV Pathogenesis Programme, Durban, South Africa, ^4^Africa Health Research Institute, Durban, South Africa, ^5^University College London, Division of Infection and Immunity, London, United Kingdom, ^6^Ragon Institute of Massachusetts General, Massachusetts Institute of Technology and Harvard University, Cambridge, United States, ^7^Howard Hughes Medical Institute, Chevy Chase, United States, ^8^Walter Reed Army Institute of Research, U.S. Military HIV Research Program, Silver Spring, United States, ^9^Henry M. Jackson Foundation for the Advancement of Military Medicine, Inc., Bethesda, United States, ^10^Henry M. Jackson Foundation Medical Research International, Nairobi, Kenya, ^11^Kenya Medical Research Institute, U.S. Army Medical Research Directorate, Nairobi, Kenya, ^12^Armed Forces Research Institute of Medical Sciences, Bangkok, Thailand, ^13^Stellenbosch University, South African Centre for Epidemiological Modeling and Analysis (SACEMA), Stellenbosch, South Africa, ^14^University of California at San Francisco, Laboratory Medicine, San Francisco, United States, ^15^Vitalant Research Institute, San Francisco, United States, ^16^National Center for HIV Viral Hepatitis, STD and TB Prevention, Division of HIV Prevention, Atlanta, United States, ^17^Facente Consulting, Richmond, United States, ^18^New Mexico Consortium, Los Alamos, United States, ^19^Los Alamos National Laboratory, Theoretical Division, Los Alamos, United States, ^20^University of Washington, Department of Global Health, Seattle, United States, ^21^University of Washington, Department of Microbiology, Seattle, United States, ^22^Institute of Infectious Disease and Molecular Medicine, University of Cape Town, Cape Town, South Africa, ^23^Santa Fe Institute, Santa Fe, United States, ^24^Fred Hutchinson Cancer Center, Public Health Sciences Division, Seattle, United States, ^25^University of Washington, Department of Medicine, Seattle, United States, ^26^University of Washington, Department of Laboratory Medicine, Seattle, United States, ^27^University of Washington, Department of Biostatistics, Seattle, United States, ^28^University of Cape Town, Wellcome Centre for Infectious Diseases Research in Africa, Department of Pathology, Faculty of Health Sciences, Cape Town, South Africa, ^29^National Health Laboratory Service, Cape Town, South Africa

**Background: **Precisely identifying the time of acquisition is important to evaluate correlates and protective thresholds in HIV prevention clinical trials. However, estimating acquisition timing is often challenging due to relatively sparse visit schedules and variable biomarker detection during early HIV acquisition. To address these challenges, statistical methods were developed to better estimate the time of acquisition from control cohorts (FRESH/RV217) for application to prevention trials such as the AMP trials.

**Methods: **Timing estimation methods utilized either diagnostic test markers or sequence diversity from HIV‐1 GP (gag/Δpol) and REN (rev/vpu/env/Δnef) regions. For AMP, we used Bayesian methods to combine these estimators and thus obtain higher precision and accuracy. The resulting Bayesian estimator outputs posterior probability distributions, from which we generated point estimates and credible intervals (CI) for the timing estimates, as well as the date of diagnosable acquisition (DDA), with the goal of comparing them to the gold standard data from the accurately timed acute acquisition FRESH and RV217 studies. The “gold standard” estimator for DDA was computed as the mid‐point between a participant's true last negative and first positive diagnostic tests, which is accurate and very precise (4 days, range 2−7 days) due to the frequent sampling in these studies.

**Results: **Overall, the 95% credible interval (CI) was smaller for the combined estimator (median width 6 days, IQR 4−21.5 days) than for the diagnostic estimator alone (median width 15 days, IQR 11−23 days). The overall coverage of the COB was lower for the combined than the diagnostic estimator (0.515 vs. 0.835, respectively) although the CI is more accurate for combined. The differences between the estimators were especially notable in antibody‐negative samples (median 95% CI width [IQR] 3 days [3−5 days] vs. 12 days [7−14 days]) for combined and diagnostic, respectively.

**Conclusions: **Accurate timing estimators help evaluate the effectiveness of preventive measures and understanding the time of HIV acquisition can help tailor intervention doses, in bNab studies such as the AMP and future trials. Acute infection data, as described here for the RV217 and FRESH cohorts, also provide valuable insights into viral dynamics associated with the acquisition and establishment of infection.

### OA1107LB Assessment of infusion‐related reactions after intravenous administration of HIV monoclonal antibodies PGT121.414.LS, PGDM1400.LS or VRC07‐523LS (alone or in combination with PGT121, PGDM1400, 10–1074, PGT121.414.LS, PGDM1400.LS), in five phase 1 studies

S. P Goulart ^1^, M. Anderson^1^, C. A Paez^1^, R. De La Grecca^1^, D. Sanchez Thomas^2^, M. Jones^1^, C. Yu^1^, S. T Karuna^1^, S. Takuva^1^, T. Gamble^3^, P. Andrew^3^, V. Bailey^4^, C. L Gay^5^, M. P Lemos^1^, M. E Sobieszczyk^6^, S. B Mannheimer^7^, S. Edupuganti^8^, C. B Hurt^5^, K. E Stephenson^9^, C. Kelley^8^, M. Siegel^10^, S. Mahomed^11^, L. L Polakowski^12^, M. Yacovone^12^, S. Regenold^12^, C. Yen^12^, J. G Baumblatt^12^, H. Spiegel^13^, M. Pensiero^12^, M. Caskey^14^, L. Gama^15^, D. H Barouch^9^, O. Hyrien^1^, M. S Cohen^5^, L. Corey^1^, S. R Walsh^16^

^1^Fred Hutchinson Cancer Center, Vaccine and Infectious Disease Division, Seattle, United States, ^2^Hospital Italiano de Buenos Aires, Buenos Aires, Argentina, ^3^FHI 360, Durham, United States, ^4^Hutchinson Centre Research Institute of South Africa, Chris Hani Baragwanath Academic Hospital, Johannesburg, South Africa, ^5^University of North Carolina at Chapel Hill, Institute for Global Health & Infectious Diseases, Chapel Hill, United States, ^6^Columbia University Vagelos College of Physicians and Surgeons, Division of Infectious Diseases, New York, United States, ^7^Columbia University Mailman School of Public Health, Department of Epidemiology, New York, United States, ^8^Emory University School of Medicine, Atlanta, United States, ^9^Harvard Medical School, Center for Virology and Vaccine Research, Beth Israel Deaconess Medical, Boston, United States, ^10^George Washington University, School of Medicine and Health Sciences, Washington, DC, United States, ^11^CAPRISA, Durban, South Africa, ^12^National Institute of Allergy and Infectious Diseases, Rockville, United States, ^13^Kelly Government Solutions, Contractor to National Institute of Allergy and Infectious Diseases, National Institutes of Health, Bethesda, United States, ^14^Rockefeller University, New York, United States, ^15^National Institute of Allergy and Infectious Diseases, Vaccine Research Center, Bethesda, United States, ^16^Harvard Medical School, Boston, United States

**Background: **Monoclonal antibodies (mAbs) are used to treat and prevent diverse clinical conditions, including infectious diseases, and hold promise for HIV prevention via passive immunization. Infusion‐related reactions (IRRs) include a constellation of systemic signs and symptoms, that have a temporal and likely causal relationship to mAb administration. Knowing the frequency and characteristics of IRRs after mAb administrations is critical to establishing a safe and well‐tolerated HIV prevention strategy.

**Methods: **This retrospective, cross‐protocol analysis assessed five phase 1 randomized, multicentre studies conducted in the United States, Switzerland, South Africa, Kenya and Zimbabwe in people living without HIV. We assess the frequency and characteristics of IRRs among participants who received intravenous (IV) infusions of single mAbs and dual or triple combinations. The dosing range was 2.5−40 mg/kg of one mAb single dose or every 16 weeks. Additionally, mAbs were sequentially administered in dual or triple combinations at 20−40 mg/kg or 1.4 g per mAb every 16 weeks. HVTN127/HPTN087 (*n* = 59), HVTN128 (*n* = 24), HVTN130/HPTN089 (*n* = 27), HVTN136/HPTN092 (*n* = 20) and HVTN 140/HPTN 101 (*n* = 57).

**Results: **One hundred and eighty‐seven participants received 492 infusions. Median age was 27 years and 56% were assigned female at birth. Sixteen participants (9%) reported 21 IRRs (4% of infusions), all of which were mild (*n* = 16) or moderate (*n* = 5). Twelve (57%) occurred in participants who received VRC07‐523LS alone and nine (42.9%) occurred after a combination. The most common reported symptoms were fatigue, chills, myalgia and headache. Most reactions started within a few hours after the infusions were completed and were resolved by 24 hours without intervention. Ten (47.6%) occurred after the first administration, and IRRs were not noted to be more severe after a subsequent infusion. IRRs led to permanent discontinuation in six (38%) participants, three after their second IRR.

**Conclusions: **IRRs after IV administration of anti‐HIV mAbs in these trials were uncommon, mild to moderate, and most were self‐limited. These findings support the safe administration of these mAbs combinations by IV infusions in future HIV prevention clinical trials.

### OA1207LB HIV BG505 SOSIP.664 trimer with 3M‐052‐AF+alum induces a lasting IFN signature that correlates with the development of HIV‐1 antibodies

L. Ballweber Fleming ^1^, M. P. Lemos^1^, V. Voillet^1,2^, C. Marini‐Macouzet^1^, R. Astronomo^1^, M. Shen^1^, Y. W Azzam^1^, J. Cheney^1^, M. Brewinski^3^, W. Hahn^1,4^, H. Janes^1^, M. C Keefer^5^, S. Edupuganti^6^, I. Frank^7^, J. Maenza^1,4^, L. R Baden^8^, D. Montefiori^9,10^, G. Tomaras^10,11,12^, N. Rouphael^13^, E. Andersen‐Nissen^1,2^, S. De Rosa^1,4^, M. J. McElrath^1,4^

^1^Fred Hutchinson Cancer Center, Vaccine & Infectious Diseases, Seattle, United States, ^2^Cape Town HVTN Immunology Lab, HCRISA, Cape Town, South Africa, ^3^National Institute of Allergy and Infectious Diseases, NIH, Division of AIDS, Bethesda, United States, ^4^University of Washington, Medicine, Seattle, United States, ^5^University of Rochester, Medicine, Rochester, United States, ^6^Emory University School of Medicine, Medicine/Division of Infectious Diseases, Atlanta, United States, ^7^University of Pennsylvania, Perelman School of Medicine, Philadelphia, United States, ^8^Brigham and Women's Hospital, Boston, United States, ^9^Duke University, Surgery, Durham, United States, ^10^Duke University, Duke Human Vaccine Institute, Durham, United States, ^11^Duke University, Center for Human Systems Immunology, Durham, United States, ^12^Duke School of Medicine, Integrative Immunobiology, Durham, United States, ^13^Emory University School of Medicine, Hope Clinic, Atlanta, United States

**Background: **HVTN137 is a phase 1 trial evaluating the safety and immunogenicity of HIV‐1 BG505 SOSIP.664 gp140 (BG505SOSIP), a stable soluble envelope, with distinct adjuvant formulations, including 5 mcg of TLR7/8 adjuvant 3M‐052‐AF+alum, and alum alone. During dose‐escalation testing (Part A), BG505SOSIP+3M‐052‐AF+alum generated autologous tier 2 HIV‐1 neutralizing antibody responses in three of five vaccinees. In comparison with other adjuvants (Part B), we studied immunological signatures of blood and rectal tissue to identify how these adjuvants activate innate responses, potentiate antibody responses and return to homeostasis.

**Methods: **Participants received placebo (*n* = 6), BG505SOSIP+alum (*n* = 6) or BG505SOSIP+3M‐052‐AF+alum (*n* = 8) at months 0, 2 and 6. Blood was collected at baseline, days 1, 3 and 7 post‐first immunization and 2.5 weeks post‐third vaccination (2.5WPTV); with cells evaluated by flow cytometry and differentially expressed genes (DEGs) assessed by bulk RNA‐seq. Optional rectal biopsies were collected at baseline and 2.5WPTV for DEG analysis. Serum binding Abs (bAbs) against BG505SOSIP were measured by binding antibody multiplex assay at 2.5WPTV.

**Results: **Innate immune responses after BG505SOSIP+3M‐052‐AF+alum, but not placebos or BG505SOSIP+alum, peaked at days 1−3, showing a strong IFN type I signature, increased activated CD86^+^ monocytes, and transient depletion of neutrophils, NKs and CD4^+^ T cells from the blood. Although cellular responses returned to baseline by Day 7, 96 IFN DEGs remained upregulated at 2.5WPTV, 25 of which were also increased in rectal tissue, including MX1, IFIT1, TRIM6, IFI44, IFI44L, CMPK2 and HERC5. At 2.5WPTV, 5/6 BG505SOSIP+3M‐052‐AF+alum recipients had developed a high magnitude of BG505SOSIP bAbs (>3000 MFI), 1/8 for BG505SOSIP+alum and 0/6 were seen in placebos. At 2.5WPTV in blood, but not rectum, the cumulative expression of the IFN module (M127) positively correlated with the magnitude of bAbs (*r* = 0.942 *p* = 0.017).

**Conclusions: **IFN signalling pathways in the blood of BG505SOSIP+3M‐052‐AF+alum vaccinees peaked 1−3 days post‐immunization, and mostly returned to baseline by 2.5WPTV. However, both in blood and rectal tissue, a subset of type I IFN DEGs persisted only in BG505SOSIP+3M‐052‐AF+alum vaccinees, indicating a 2.5‐week‐long TLR7/8 response in blood and mucosa. This IFN signature in blood correlated with BG505SOSIP bAbs, suggesting it may support the development of a strong B cell response.

### OA1307LB Observed adherence‐concentration benchmarks for emtricitabine/tenofovir alafenamide pre‐exposure prophylaxis for African women

K. Mugwanya ^1,2^, N. Mugo^1,3^, M. Saina^3^, C. Brown^1^, N. Akelo^3^, T. Schaafsma^1^, E. Rechkina^1^, K. Ngure^1,4^, S. Mbugua^3^, E. Hill^1^, B. Chohan^1^, E. Gichuru^3^, P. Nzuve^3^, L. Wu^1,2^, S. Morrison^1^, J. Rooney^5^, L. Bushman^6^, P. Anderson^6^, for the Women Benchmark Study Team

^1^University of Washington, Global Health, Seattle, United States, ^2^University of Washington, Epidemiology, Seattle, United States, ^3^Kenya Medical Research Institute, Nairobi, Kenya, ^4^Jomo Kenyatta University of Agriculture and Technology, Nairobi, Kenya, ^5^Gilead Sciences, Inc, Foster City, United States, ^6^University of Colorado Anschutz Medical Campus, Pharmaceutical Sciences, Skaggs School of Pharmacy and Pharmaceutical Sciences, Aurora, United States

**Background: **Tenofovir diphosphate (TFV‐DP) thresholds in dried blood spots (DBS) derived from U.S. populations for emtricitabine/tenofovir alafenamide (F/TAF) pre‐exposure prophylaxis (PrEP) helped to interpret F/TAF efficacy for men who have sex with men. No study has yet defined TAF benchmarks for African women or whether U.S. population‐derived benchmarks apply to them.

**Methods: **Between March and December 2023, we conducted a randomized pharmacokinetic study of oral F/TAF PrEP in DBS and peripheral blood mononuclear cells (PBMCs) among 54 Kenyan women without HIV. Women were randomized to 2, 4 or 7 oral F/TAF PrEP doses/week (clinicaltrials.gov: NCT05140954). Dosing was directly observed for 10 weeks. TFV‐DP was quantified in DBS and PBMCs using validated LC‐MS/MS assays at the University of Colorado. Observed median concentrations were compared between dosing groups using the Wilcoxon test. Geometric mean ratios of steady‐state PBMC TFV‐DP concentrations from F/TAF were compared to respective concentrations from a contemporaneous Women Benchmark study of emtricitabine/tenofovir disoproxil fumarate (F/TDF).

**Results: **Median (IQR) age and creatinine clearance at baseline were 23 years (21–25) and 126 ml/min (110−149), respectively. Overall, >99.9% of expected doses were observed. Observed median (IQR) week 10 DBS TFV‐DP concentrations were 432 (407−504), 1214 (930−1377) and 2345 (2063−3006) fmol/punch for 2, 4 and 7 doses/week. These thresholds are comparable to the respective 25th/75th percentiles from the U.S. TAF‐DBS study (432−657, 952−1449 and 1980−2962 fmol/punch). TFV‐DP from F/TAF concentrated in PBMCs with median (IQR) steady‐state concentrations of 70 (55−94), 229 (159−278) and 680 (462−864) fmol/10^6^ cells for 2, 4 and 7 doses/week. In contrast, median (IQR) steady‐state PBMC TFV‐DP concentrations from F/TDF were 9 (7−19), 28 (21−34) and 49 (36−63) for 2, 4 and 7 doses/week (*p*<0.001 for all). Notably, 2 doses/week of F/TAF generated TFV‐DP levels 1.41‐fold (1.04–1.89) higher versus daily dosing with F/TDF (*p* = 0.027).

**Conclusions: **We have established F/TAF PrEP adherence benchmarks for African women and show they are within range of historical benchmarks from U.S. populations. F/TAF PrEP produced more than seven‐fold higher PBMC TFV‐DP levels versus F/TDF across adherence groups. These data reinforce the strong intrinsic potential F/TAF PrEP potency and pharmacologic forgiveness for African women in the PBMC compartment.

### OA1407LB Tackling HIV through teen pregnancy prevention: results from an innovative intervention among African American teens

R. Chande ^1^, C. Obidoa^1^, A. Potts^1^, N. Osei‐Tutu^1^, Coalition for Collaboration on HIV/AIDS Research and Intervention in Middle Georgia (CCHR)

^1^Mercer University, International and Global Studies, Macon, United States

**Background: **African Americans bear a disproportionate burden of the HIV epidemic in the U.S. Deep South, yet tailored socio‐ecologically based interventions focused on addressing the underlying factors in this epidemic in this region remain sparse. One of the overlooked factors of HIV vulnerability in this population is teen pregnancy. Despite declining rates of teen pregnancy nationally, teen pregnancy remains high in Georgia. African Americans are disproportionately affected by teen pregnancy in Middle Georgia. Between 2018 and 2022, the average teen pregnancy rate was 46.5 for African Americans and 11.5 for whites. The continued progression of these dual epidemics in this population calls for context‐based interventions that challenge cyclical factors that perpetuate teen pregnancy.

**Methods: **We designed an innovative *curriculum: Untrapped: Teen Pregnancy Prevention Curriculum for African American Teens ages 11–19*. The multi‐layered, co‐educational intervention. It targets four main aspects of the social ecology of the teenager: cognitive growth through didactic instruction, intra‐personal growth through journaling, interpersonal peer growth enhanced through collaborative development of a problem tree and socio‐structural influence through a problem tree display in the learning space.

**Results: **The curriculum was pilot‐tested among middle‐school and high‐school students at an after‐school programme in a mid‐sized city in Middle Georgia. A pre‐intervention and post‐intervention survey was administered, with individual interviews at the programme's conclusion. Results were analysed quantitatively and qualitatively. Results showed a change in the ideal age for sexual debut. The age at which one should start having sex was higher after the intervention. Sixty‐nine percent (69%) reported that having a child as a teenager was a problem post‐intervention compared to 47% pre‐intervention. When the programme concluded, 85% reported high confidence in their ability to prevent pregnancy compared to 26% pre‐intervention. Qualitative responses revealed that the teenagers enjoyed the interactive nature of the programme. Several reported appreciating how it allowed them to voice their opinions. Post‐intervention problem tree activity revealed a more versatile understanding of the various factors that shape teen pregnancy.

**Conclusions: **The intervention was effective in addressing overlooked aspects of risks associated with teen pregnancy and instrumental in raising critical awareness about factors that shape teen sexual choices and attitudes about teen pregnancy.

### OA1607LB Drug‐agnostic transcutaneously refillable subdermal implant for ultra‐long‐acting delivery of antiretrovirals for HIV prevention

F. Pons‐Faudoa^1^, N. Di Trani^1^, I. Facchi^1^, A. Simeone^1^, L. Bushman^2^, J. Mai^1^, Y. Liu^1^, K. Shelton^3^, P. Nehete^3^, J. Kimata^4^, C. Y. X. Chua^1^, P. Anderson^2^, R. Arduino^5^, A. Grattoni ^1^

^1^Houston Methodist Hospital, Nanomedicine, Houston, United States, ^2^University of Colorado at Denver, Denver, United States, ^3^MD Anderson Cancer Center, Bastrop, United States, ^4^Baylor College of Medicine, Houston, United States, ^5^University of Texas Health Science Center Medical School, Houston, United States

**Background: **Long‐acting sustained antiretroviral (ARV) release systems for protection against human immunodeficiency virus (HIV) could improve and maintain adherence, a longstanding global health challenge. Drawing parallel from contraceptives, having options that enable personal preferences could increase uptake, acceptability and efficacy of HIV pre‐exposure prophylaxis (PrEP). These long‐acting delivery systems include oral tablets, injectables and implants. Here, we present a drug‐agnostic transcutaneously refillable subdermal implant for ultra‐long‐acting controlled delivery of potent ARVs, with a primary focus on islatravir (ISL) and MK‐8527.

**Methods: **The subdermal implant comprises of a biocompatible titanium casing acting as the drug reservoir, internally mounted with a silicon nanochannel membrane to control release. Constant and sustained ARV release occurs through constrained diffusion of drug molecules across the nanochannels. Subdermal ISL‐eluting implants were evaluated in non‐human primates (NHPs) for a 29‐month pharmacokinetic study as well as PrEP efficacy against repeated rectal and vaginal simian HIV (SHIV) challenges in males and females, respectively. Long‐term safety and tolerability to the implants were evaluated. Further, the implants were evaluated for in vivo release of MK‐8527, lenacapavir (LEN), bictegravir (BIC) and other hydrophobic ARVs.

**Results: **Sustained ultra‐long release of ISL from the subdermal implant was shown through stable plasma and peripheral mononuclear blood cell drug levels for over 29 months in NHP without fluctuation or implant refilling. More importantly, 100% protection against rectal and vaginal SHIV exposures in NHP substantiated PrEP efficacy of the ISL‐eluting implants. Further, safety and tolerability demonstration in NHP validated the feasibility of long‐term implant deployment. Additionally, sustained release and tolerability of MK‐8527 was also achieved in vivo. Further, the release of hydrophobic ARVs, namely LEN, BIC and DTG, highlighted drug‐agnosticity of the implant.

**Conclusions: **Our ultra long‐acting subdermal implant offers safe and effective long‐lasting protection against HIV, where minimally invasive transcutaneous refillability extends release potentially throughout the recipient's lifespan. Implant drug‐agnosticity imparts flexibility for single‐ or multi‐ARV delivery for PrEP or treatment, as well as combination with contraceptives, serving as a multi‐prevention technology. Importantly, the cost of good analysis estimates drug‐loaded implant could be as low as USD 36/year, substantiating clinical viability for adoption in low‐resource countries.

### OA1807LB Intravenously delivered broadly neutralizing antibody VRC01 reaches the rectal lamina propria and provides partial protection in ex‐vivo challenges with HIV‐1 (HVTN116)

M. P Lemos ^1^, R. Astronomo^1^, E. Andersen‐Nissen^1,2^, Y. Huang^1^, S. Narpala^3^, M. Prabhakaran^3^, O. Hyrien^1^, Y. Lu^1^, S. Srinivasan^1^, A. Naidoo^2^, G. Mize^1^, H. Glantz^1^, R. Gomez^1^, H. Crossgrove^1^, C. Ochsenbauer^4^, P. Mann^1^, C. Paez^1^, J. Hutter^5^, J. Czartoski^1^, C. Orrell^6^, Y. Singh^7^, P. Garnett^7^, S. Thomson^8^, J. Maenza^1^, D. Fredricks^1^, J. Mascola^3^, A. B. McDermott^3^, L.‐G. Bekker^7^, M. J. McElrath^1,9^

^1^Fred Hutchinson Cancer Center, Vaccine and Infectious Diseases Division, Seattle, United States, ^2^Cape Town HIV Immunology Lab, Hutchinson Centre Research Institute of South Africa, Cape Town, South Africa, ^3^National Institute of Allergy and Infectious Diseases, National Institute of Health, Vaccine Research Center, Bethesda, United States, ^4^University of Alabama Birmingham, Birmingham, United States, ^5^National Institute of Allergy and Infectious Diseases, National Institute of Health, Bethesda, United States, ^6^Desmond Tutu HIV Centre, University of Cape Town, Institute of Infectious Diseases and Molecular Medicine and Department of Medicine, Cape Town, South Africa, ^7^Desmond Tutu HIV Centre, University of Cape Town, Cape Town, South Africa, ^8^University of Cape Town, Department of Gastroenterology, Cape Town, South Africa, ^9^University of Washington, Department of Medicine, Seattle, United States

**Background: **Effective concentrations of broadly neutralizing monoclonal antibodies are likely needed in the intestinal mucosa for HIV‐1 immune‐prophylaxis. Intravenous VRC01 at 10 and 30 mg/kg was evaluated in two trials which demonstrated partial efficacy against neutralization‐sensitive HIV‐1 strains. A parallel study, HVTN116, compared mucosal VRC01 levels, localization and functionality by doses, sex‐assigned‐at‐birth, and geographic locations.

**Methods: **Thirty‐one participants without HIV from Cape Town and Seattle received four VRC01 infusions (10 or 30 mg/kg) given once every 2 months. Paired blood, rectal secretions and faecal matter were collected pre‐infusion, and after the first and fourth infusions to examine bacterial diversity (16S sequencing) and VRC01 levels (Singulex). Rectal biopsies were collected pre‐infusion and 1−28 weeks post‐fourth infusion to assess VRC01 levels, localization (immunohistochemistry) and functionality (ex‐vivo explant challenge).

**Results: **Within 2 weeks post‐fourth infusion, VRC01 levels were ∼2 × higher in both sexes assigned at birth (SAAB) in serum (*p* = 0.008), rectal biopsies (*p* = 0.008) and rectal secretions (*p* = 0.047) in the 30 versus 10 mg/kg group. There were no dose‐related differences in mucosal half‐life, accumulation (*p* = 0.46), or penetration into rectal tissue (*p* = 0.93) or secretions (*p* = 0.67). Rectal biopsies from both doses showed significant ex‐vivo protection against HIV‐1_Bal26_ challenges 1−2 weeks post infusion, which waned by 5−6 weeks (EC80 = 0.17 μg/ml). Neither dose protected against HIV‐1_Du422_ (EC80>50 μg/ml) or HIV‐1_1086_ (EC80 = 2 μg/ml) challenges, which require higher IC80.

There were no differences in VRC01 rectal levels (*p* = 0.78), half‐life (∼22 days) or penetration into rectal lamina propria (*p* = 0.11) by SAAB. The composition of Cape Town faecal microbiome differed from Seattle (*p*<0.001) but was not modified by VRC01 infusion. Cape Town participants also had shorter VRC01 half‐life and lower levels at 5−6 weeks in serum (*p* = 0.048) and rectal biopsies (*p* = 0.048), even after adjustment for body weight and VRC01 dose.

**Conclusions: **Both VRC01 doses reached the rectal compartments of participants regardless of SAAB with similar pharmacokinetics, although peak levels differed by dose and geographic region. Both doses mediated short‐term, partial protection against HIV‐1_Bal26_ in ex‐vivo explant challenges; but were insufficient against strains with higher IC80s. Increased dose and repeated infusions did not improve penetration or rectal half‐life. Longer‐lived and more potent antibody cocktails may be needed for effective HIV‐1 immuno‐prophylaxis.

### OA2007LB HIV PEP‐in‐Pocket (“PIP”) facilitates the de‐medicalization of HIV prevention

K. Fisher^1^, M. Billick^2^, I. Bogoch ^1,2^

^1^Toronto General Hospital, Toronto, Canada, ^2^University of Toronto, Toronto, Canada

**Background: **Current HIV prevention programmes tend to offer relatively static advice even though individual HIV risk is dynamic; for example, daily pre‐exposure prophylaxis (PrEP) is routinely offered with limited re‐evaluation of appropriateness. While this approach works for many, offering alternative approaches to HIV prevention can broaden the reach to more individuals who may benefit from pharmacologic prevention tools and promote maintained engagement in care.

PIP is a novel approach to HIV prevention that mitigates gaps in care and significantly reduces the number of healthcare interactions associated with the provision of post‐exposure prophylaxis (PEP). PIP involves proactively identifying clients who may have infrequent and occasionally unexpected HIV exposures and providing them with a full 28‐day prescription for guideline‐approved ARVs. Clients are instructed to self‐initiate medications in the event of an exposure and follow‐up for baseline screening within 14 days of initiation. Importantly, PIP clients have access to a full 28‐day course of treatment without needing to visit urgent care or the emergency department.

**Methods: **PIP was integrated into two HIV prevention clinics as part of a “buffet approach” that prioritizes client autonomy and choice by offering a range of options and the flexibility to change as needed. Clinicians assess prevention strategies and HIV risk during every interaction and provide education on alternative approaches within the context of current life circumstances. Using a standardized form, we conducted a retrospective evaluation of PIP uptake, PIP use and client‐led modifications to HIV prevention strategies.

**Results: **PIP was prescribed to 126 individuals who were followed for 212 patient‐years. There were 85 instances of PIP initiation by 36 individuals; though baseline screening was attended only 30.3% of the time, 6‐month follow‐up attendance following PIP initiation was 98.7%. Zero HIV seroconversions were detected. 31.7% of clients switched from PIP to PrEP, and 29.4% switched from PrEP to PIP.

**Conclusions: **The introduction of PIP has expanded available options for HIV prevention, which is of particular importance for those who have a lower frequency of HIV exposures. PIP promotes autonomy and reduces many burdens associated with emergency PEP access. PIP may be considered a cost‐effective harm‐reduction approach to HIV prevention.

### OA2407LB Impact evaluation of a combination HIV prevention intervention for adolescent girls and young women in South Africa: a non‐randomized controlled trial (the HERStory 3 study)

K. Jonas ^1^, K. Bergh^1^, C. Mathews^1^, N. Morris^1^, Z. Duby^1^, T. McClinton Appollis^1^, N. Ebrahim^1^, B. Singh^2^, E. Cutler^2^, C. Kuo^3^, A. Puren^2,4^, G. Gray^1^, C. Lombard^1^

^1^South African Medical Research Council, Cape Town, South Africa, ^2^National Institute for Communicable Diseases, Johannesburg, South Africa, ^3^American University, Washington, DC, United States, ^4^University of Witwatersrand, Johannesburg, South Africa

**Background: **Adolescent girls and young women (AGYW) in South Africa are at high risk of HIV infection. A combination HIV prevention intervention for AGYW, the My Journey Programme, was implemented in South Africa from 2016 to the present, aiming to reduce HIV incidence, teenage pregnancy and GBV, and to increase retention in school and access to economic opportunities. The objective of this study is to determine the impact of the My Journey Programme on HIV prevalence (primary outcome), knowledge of HIV status, coverage of HIV prevention and care, pregnancy prevention, and school dropout.

**Methods: **We conducted a “post‐intervention” survey in 12 intervention sub‐districts, and 12 comparison sub‐districts with equivalent demographics and HIV prevalences, across eight provinces in South Africa. Two sites were purposefully selected within each intervention and comparison area, generating 48 sites. In each site, we conducted a representative household survey of 100 AGYW aged 15−24 years, (*N* = 4800). The study was powered to detect a decrease in HIV prevalence from 12% to 6%. Dried blood spot specimens were collected and participants self‐completed an electronic questionnaire. Frequencies and percentages for each outcome were presented for intervention and comparison arms. Using a mixed effect model, accounting for clustering at four levels and potential confounders (age, maternal orphanhood, socio‐economic status and educational enrolment), the odds ratio of the study arms was determined.

**Results: **Across study arms, 37,714 households were visited, 22,263 were screened, 5150 AGYW were invited and 5025 participated. HIV prevalence was 9.5% in the intervention and 10.4% in the comparison arm (OR: 0.88; 95% CI: 0.46–1.70). Knowledge of HIV status was 84.7% in the intervention and 80.5% in the comparison arm (OR: 1.46; 95% CI: 1.05–2.03). In the intervention arm, there were substantially fewer participants who did not know what PrEP was (28.2% vs. 35.0%; *p* = 0.03) and substantially more participants who had ever used PrEP (26.4% vs. 13.0%; *p* = 0.03). There was almost no intervention impact on condom or contraception use, but potential barriers to access were less prevalent in the intervention arm.

**Conclusions: **These findings demonstrate the potential and value of combination HIV prevention for AGYW.

### OA2507LB Crowdsourcing strategies to implement CAB‐LA for sexual minority men in Chicago through a cutting‐edge innovation tournament

A. Van Pelt ^1,2,3,4^, E. Casline^2^, G. Cook^2^, G. Phillips II^1,5^, J. Cestou^6^, B. Mustanski^1,3,5^, R. Beidas^1,2^

^1^Northwestern University, Medical Social Sciences, Chicago, United States, ^2^Northwestern University, Center for Dissemination and Implementation Science, Chicago, United States, ^3^Northwestern University, Third Coast Center for AIDS Research, Chicago, United States, ^4^Northwestern University, Havey Institute for Global Health, Chicago, United States, ^5^Northwestern University, Institute for Sexual and Gender Minority Health and Wellbeing, Chicago, United States, ^6^Chicago Department of Public Health, Chicago, United States

**Background: **Reducing HIV incidence requires the effective implementation of evidence‐based prevention practices. In Chicago, HIV disproportionately impacts sexual minority men (SMM). Long‐acting injectable cabotegravir (CAB‐LA) offers a new form of HIV prevention. To maximize the uptake of CAB‐LA in Chicago, co‐designing implementation strategies is critical. Through a partnership with key constituents, this research employed cutting‐edge participatory methods to inform the implementation of CAB‐LA among SMM (≥13 years), particularly Black and Latino populations.

**Methods: **Innovation tournaments follow a three‐step process: (1) participant submission of ideas, (2) participant voting on ideas and (3) evaluation of ideas by a committee. A platform was developed to host the innovation tournament and allow participants to respond to the following prompt: “Describe your idea for how to get this new form of PrEP to gay, bisexual, and other men who have sex with men (12 years and older) who want it in Chicago.” Participants were recruited through social media, train advertisements, fliers and in‐person recruitment. Ideas were submitted in English and Spanish. At the conclusion of the tournament, a committee of constituents with diverse expertise convened to evaluate the ideas for feasibility and acceptability and to select winning ideas.

**Results: **Forty‐two participants completed 53 submissions comprised of 72 discrete ideas for implementation strategies. Advertisement on the public train yielded the most submissions (31%). Participants represented the key populations (31% Black, 19% Latino, 64% sexual minority population). Submissions described ideas to increase awareness (e.g. campaign on social media and dating apps, identification of LGBTQ champion), reduce cost (e.g. shot subsidization, transportation voucher), integrate care (e.g. STI services, pharmacies) and partner with community spaces (e.g. pop‐up clinics, schools).

**Conclusions: **This research will contribute to the production of a menu of co‐designed implementation strategies, which can guide plans for CAB‐LA integration in Chicago and provide insights for other regions. As the first innovation tournament focused on HIV prevention, this research can provide a framework for participatory approaches across the care continuum. Given that the co‐design of implementation strategies often does not involve the participation of individuals with lived experiences, this work will centre the voices of those who will benefit most.

### OA2607LB Germline targeting SHIVs for the assessment of HIV vaccine immunogens

R. Habib ^1,2^, K. Bayruns^1^, Z. Lin^1^, K. Sowers^2^, Y. Park^2^, B. Hahn^2^, D. Weiner^1^, J. Pallesen^1^, G. Shaw^2^, D. Kulp^1,2^

^1^Wistar Institute, Vaccine and Immunotherapy Center, Philadelphia, United States, ^2^University of Pennsylvania, Perelman School of Medicine, Philadelphia, United States

**Background: **The induction of broadly neutralizing antibodies (bnAbs) against HIV Env remains a major goal of HIV vaccine development. Germline targeting, where immunogens are designed to engage bnAb unmutated common ancestor (UCA) precursor B cells, is a promising strategy for the development of priming immunogens. Here, we explore the use of the SHIV model for the evaluation of HIV immunogens through the design of germline targeting SHIVs. As proof of principle of this approach, we design and infect rhesus macaques (RMs) with a SHIV with enhanced affinity for the UCA of an RM V2‐apex bnAb lineage V033‐a.01 to re‐elicit V033‐like lineages.

**Methods: **V033 UCA affinity‐enhancing mutations were identified through mammalian display mutagenesis of a stabilized Env Q23.17‐based trimer. These mutations were then introduced into the WT SHIV.Q23.17, resulting in the germline targeting SHIV Q23.V033GT. This germline targeting SHIV demonstrated over 500‐fold enhanced sensitivity to neutralization by the V033 UCA. Three RMs were inoculated with SHIV.Q23.V033GT. Neutralization assays, EMPEM and single genome sequencing were used to characterize the antibody response and Env escape in these RMs.

**Results: **All RMs were productively infected with SHIV.Q23.V033GT. One RM developed a rapid progressor phenotype and failed to mount a detectable antibody response. In the two evaluable RMs, Env escape at the V2‐apex C‐strand and neutralization mapping with Env mutants confirmed the accelerated development of V2‐apex C‐strand targeted nAb responses as soon as week 12 post‐infection. EMPEM suggested that these lineages had binding footprints similar to the V033 bnAb. Both RMs developed limited, C‐strand targeted, heterologous tier‐2 neutralization.

**Conclusions: **We find several advantages to the use of the SHIV model for immunogen evaluation: (i) high antigenic loads during infection maximize the probability of engaging bnAb UCAs; (ii) SHIVs coevolve with bnAb lineages resulting in the acquisition of neutralization breadth, verifying that primed B cells are genuine bnAb precursors; and (iii) sequencing of Env escape serves as a sensitive indicator of nAb‐targeted epitopes. Overall, these data demonstrate a novel use of the SHIV model for the design and testing of HIV immunogens, and show the promise of Q23.V033GT as an immunogen to prime V2‐apex bnAb lineages in RMs.

## AUTHOR INDEX

### A

Abay, M. OA1205

Abbate, M.C. OA0402

Abuna, F. OA2303, OA2305

Abuya, T. OA1605

Acero, C. OA1705

Acharya, P. OA1202

Achayo, H. OA2006

Ackerman, M. OA0607LB

Adamczyk, A. OA1002

Adams, R. OA2403

Adamu, V. OA0103

Adamu‐Oyegun, S. OA0103

Adjei, P. OA1203

Àdori, M. OA2603

Adori, M. OA2605

Agrahari, V. OA0206

Agrawal, P. OA2604

Agrawal, S. OA1504

Aguilar‐Gurrieri, C. OA0304

Agwu, A. OA0204

Agyei, Y. OA2306

Ahmed, K. OA0202

Ainsua‐Enrich, E. OA0304

Akapirat, S. OA0305, OA1103

Ake, J. OA0704, OA1103

Ake, J.A. OA0305, OA0306, OA1203

Akello, C. OA2402

Akelo, N. OA1307LB

Akieh‐Pirkanniemi, M. OA0206

Akim, E. OA2303, OA2305

Akingbade, B. OA2002

Akom, E.E. OA0102

Alam, M. OA1202

Alameh, M.‐G. OA1804

Alamo, S. OA0103

Aldredge, A. OA1602

Allen, E. OA0603

Allen, M. OA1206

Allinder, S. OA1302

Alrubayyi, A. OA0302

Alt, F. OA1202

Altamirano, J. OA0507LB

Althoff, K. OA1304

Amir Hamzaha, S. OA2203

Amour, M. OA1402

Andersen‐Nissen, E. OA0306, OA1105, OA1207LB, OA1807LB

Anderson, M. OA0607LB, OA1107LB

Anderson, P. OA1307LB, OA1607LB

Anderson, T. OA0204

Andrew, P. OA0605, OA0606, OA1107LB

Andrews, S. OA1206

Angumua, C. OA0103

Antinori, A. OA0205, OA1006

Antivero Battistelli, R.B. OA1002

Apedaile, D. OA0405

Apio, A. OA1406

Apps, R. OA0703

Arévalo, A. OA1002

Arístegui, I. OA1303

Araínga, M. OA0603, OA0706LB

Arduino, R. OA1607LB

Arif, M.S. OA0706LB

Arrington‐Sanders, R. OA0204

Arunmozhi, A. OA0102

Astronomo, R. OA1207LB, OA1807LB

Atkins, K. OA1403

Atujuna, M. OA0803LB

Avettand‐Fenoël, V. OA0702

Ávila‐Nieto, C. OA0304

Awor, A.C. OA0103

Ayallo, H. OA1904

Azzam, S. OA1103

Azzam, Y.W. OA1207LB

### B

Babalola, C.N. OA2304

Baden, L. OA1204

Baden, L.R. OA0306, OA1207LB

Badia, L. OA1006

Baeten, J. OA2302

Baeten, J.M. OA2003

Bagaya, B. OA2205

Baggaley, R. OA0104

Bahorich, J. OA1206

Bailey, S. OA1103

Bailey, V. OA1107LB

Bailey, V.C. OA0607LB

Bajpai, P. OA2606

Baker, D. OA0804LB

Bal, J. OA1202

Balandya, E. OA1402

Balazs, A. OA2202

Bale, S. OA2603, OA2605

Balinda, S. OA1505, OA1506

Balkus, J. OA0904

Ballweber Fleming, L. OA1207LB

Ballweber‐Fleming, L. OA1205

Bandyopadhyay, B. OA1504

Bansal, A. OA1104

Bar, K. OA0605

Baral, S. OA0106, OA1403, OA2502

Barbosa, C. OA1202

Barbosa, M.A. OA0402

Bardon, A.R. OA2003

Barouch, D. OA1105

Barouch, D.H. OA0606, OA0607LB, OA1107LB

Bartlett, J. OA1402

Bauermeister, J. OA0204

Baumblatt, J.G. OA1107LB

Bautista, A. OA0906

Bayruns, K. OA2607LB

Beauchamp, G. OA2002

Beaumont, K. OA0705

Beck, J. OA2002

Beidas, R. OA2507LB

Beigi, R.H. OA2302

Beima‐Sofie, K. OA2303

Bekker, L.‐G. OA0306, OA0803LB, OA0805LB, OA1003, OA1606, OA1807LB, OA2203

Bell, I. OA2002

Bellagamba, R. OA1006

Benmeziane, K. OA0702

Bergh, K. OA2407LB

Berry, M. OA1202

Bershteyn, A. OA0106

Beyrer, C. OA0905, OA1304

Bhakhri, H. OA2606

Bhatia, R. OA0102, OA0103

Bhattacharya, J. OA1504

Bhattacharya, T. OA0807LB

Bheemraj, K. OA0105

Bhor, V. OA1504

Bhowmick, S. OA1504

Bianchi, A. OA1006

Bikokye, W. OA2102

Biks, G.A. OA1405

Billick, M. OA2007LB

Bindlish, R. OA0107LB

Biribawa, V. OA2205

Biswas, D. OA1504

Biswas, K. OA1902

Biswas, S. OA1102

Blanco, J. OA0304

Bloom, S. OA2206

Boccardi Vidarte, A.L. OA0404

Bock, M. OA0103

Bock, P. OA2306

Bogoch, I. OA2007LB

Boily, M.‐C. OA0106

Bonsignori, M. OA1202

Booton, R.D. OA0106

Bopape, L. OA1905

Borducchi, E. OA1105

Borgognone, A. OA1103

Borquez, A. OA1304

Bowman, C. OA1202

Bracamonte, P. OA0404

Brady, J. OA2202

Brewinski, M. OA1207LB

Brinson, C. OA0203

Brockman, M. OA1104

Broder, G. OA0605

Brown, C. OA1304, OA1307LB

Brown, E. OA2302

Brown, L. OA0207

Bruce, I. OA1605

Bruzzesi, E. OA1603

Buchanan, L. OA2205

Buchbinder, S. OA1105

Budnik, P. OA0502

Bukusi, D. OA2103

Bukusi, E. OA2404

Bula, A. OA1302

Bunga, S. OA0103

Bunge, K. OA2302

Burgener, A. OA1103

Burgess, D. OA1806

Burnham, R. OA1105

Bursac, Z. OA1704

Burton, I. OA0604

Busch, M.P. OA0807LB

Bushman, L. OA1307LB, OA1607LB

### C

Cai, Y. OA2004

Cain, D. OA1202

Calzavara, D. OA1006

Camargo, M.L.F. OA0402

Camargo, R.F. OA0402

Camp, C. OA0503

Canda, M. OA0103

Candela, C. OA1603

Cantos, V. OA0804LB

Carabelli, J. OA0304

Carias, A.M. OA0603

Carlton, K. OA1206

Carnathan, D. OA2603, OA2605

Carpe Elias, S. OA2202

Carpp, L.N. OA0306, OA0807LB

Carrillo, J. OA0304

Cartasso, C. OA1002

Carter, C. OA0207

Carter, D.S. OA1602

Caruso, E. OA1006

Caskey, M. OA1107LB

Casline, E. OA2507LB

Casmir, E. OA2003

Castagna, A. OA1006, OA1603

Castaneda, C. OA0103

Castillo, M. OA0404

Castro, M. OA1206

Castro‐Arteaga, M. OA0405

Castronuovo, P. OA0202

Catala‐Moll, F. OA1103

Catalano, G. OA1603

Cavarelli, M. OA0702

Celum, C. OA0202

Cernuschi, M. OA1006

Cesar, C. OA1303

Ceschel, M. OA1303

Cestou, J. OA2507LB

Chabalala, B. OA2406

Chabela, M. OA2403

Chahroudi, A. OA1806

Chande, R. OA1407LB

Chandra, N. OA0206

Chapman, K. OA2505

Chappell, C. OA0803LB

Chatani‐Gada, M. OA2504

Chatterjee, A. OA2606

Chauhan, S. OA1504

Chawana, T.D. OA0606

Chen, H. OA1202

Chen, S. OA0306

Chen, V. OA1403

Chen, Y. OA1803

Cheney, J. OA1207LB

Cheves, L.A. OA1205

Chew, K. OA0302

Chi, B. OA1606

Chiang, C.‐I. OA0303

Chidumwa, G. OA2406

Chilongosi, R. OA1302

Chimezie, A. OA0503

Chin, E. OA0604

Chinula, L. OA2302

Chirenje, Z.M. OA0306, OA0606, OA0607LB

Chisenga, T. OA0103

Chituwo, O. OA0103

Chohan, B. OA1307LB, OA2103

Choi, S. OA2204

Chowhan, D. OA0705

Chu, C. OA0507LB

Chua, C.Y.X. OA1607LB

Cingolani, A. OA0205, OA1006

Clark, J. OA1205

Clark, M. OA0206

Clement, M. OA0207

Clotet, B. OA0304

Cluff, J. OA2603, OA2605

Coates, T.J. OA0105

Cochran, M. OA2603

Cohen, C. OA2206

Cohen, M. OA0605, OA0607LB, OA2306

Cohen, M.S. OA1107LB

Coleman Lewis, J. OA0204

Collins, L. OA0804LB

Concepcion, T. OA2305

Conlon, M. OA2402

Cook, G. OA2507LB

Cooney, E. OA1304

Corcoran, M. OA2605

Corey, L. OA0306, OA0605, OA0607LB, OA0807LB, OA1105, OA1107LB

Cottrell, C. OA1205

Cowan, T. OA0804LB

Cowden, J. OA0305, OA2204

Cozzi‐Lepri, A. OA0205

Creegan, M. OA0703

Crofoot, G. OA0203

Crossgrove, H. OA1807LB

Crowell, T. OA1203

Cummings, V. OA2002

Cunyat, F. OA0304

Currier, J. OA0605

Cutler, E. OA2407LB

Czarnogorski, M. OA0502

Czartoski, J. OA1807LB

### D

d'Arminio Monforte, A. OA0205, OA1006

Daar, E. OA0703

Das, M. OA0207

Dasgupta, S. OA1902

Dashti, A. OA1806

Davey, D.L.J. OA0105

Davis, D. OA1804

Davis, D.A. OA0403

Davis, S. OA0103

De Bona, A. OA1006

De Jesus Leon Morris, D.F. OA0405

De La Grecca, R. OA0605, OA1107LB

De Paris, K. OA1803, OA1804, OA1805

De Rosa, S. OA0306, OA1105, OA1204, OA1205, OA1207LB

de Ruiter, A. OA0502

De Vito, A. OA0205

de Vos, L.A. OA2304

De Zottis, F. OA1006

DeCamp, A. OA0605

deCamp, A.C. OA0807LB

DeCree, C. OA1602

Deeks, S.G. OA0302

Delaney, K.P. OA0807LB

Delany‐Moretlwe, S. OA0202, OA0607LB, OA0903, OA2306

Demeke, H.B. OA0103

Deng, W. OA0807LB

Dennis, M. OA1803

Dereuddre‐Bosquet, N. OA0702

Deshpande, S. OA1504

Desjardins, D. OA0702

Dettinger, J. OA2303, OA2305

Devadiga, P. OA1504

Devlin, B. OA0802LB

Dhitavat, J. OA0305

Di Trani, N. OA1607LB

Diaz, D. OA1704

Dimant, N. OA0702

Dimitrov, D. OA0106

Dinesha, T.R. OA1504

Dintwe, O. OA0306, OA1105

Diotallevi, S. OA1603

Djomand, G. OA0102

Dladla, N. OA0904, OA1403, OA1905, OA2502

Dladla, S. OA0103

Domin, E. OA1503

Doncel, G. OA0206

Dong, K.L. OA0807LB

Donnell, D. OA1306

Doria‐Rose, N. OA0303, OA0604, OA1206

Dorsey‐Spitz, J. OA1203

Dorward, E. OA0102

Douek, D. OA1206

Doyle, E. OA2603

Dropulic, L. OA1206

Du, J. OA0203

Duby, Z. OA2407LB

Dumond, J. OA0607LB

Dunn, B. OA0307LB, OA1503

Durán, A. OA1303

Dusic, E. OA0506

Dutta, S. OA2606

Dye, B. OA0607LB

Dzvelaia, T. OA2603

### E

Eakle, R. OA0102

Eaton, A. OA1202

Eaton, T. OA1503

Ebrahim, N. OA2407LB

Eckert, R. OA1103

Edlefsen, P.T. OA0807LB

Edupuganti, S. OA0606, OA1107LB, OA1207LB

Ehrenberg, P. OA0703, OA2204

Eiser, D. OA2204

Elias Rodas, D.M. OA0403

Ellenberg, P. OA2203

Eller, L.A. OA0704, OA0807LB

Elsherbini, J. OA2206

Ely, A. OA0602

Emel, L. OA2002

Engelmann, F.A. OA0706LB

Eron, J. OA0605

Esandi, M.E. OA1303

Esvan, R. OA1006

Etukiot, M. OA2402

Eudailey, J. OA1803

Evangelous, T. OA1202

Evans, B. OA0202, OA0203

### F

Facchi, I. OA1607LB

Facente, S. OA0807LB

Faircloth, K. OA1503

Fakude, C. OA2406

Fang, J. OA0502

Farach, N. OA0103

Farquhar, C. OA2103

Ferrari, G. OA0307LB, OA1503

Ferrell, D.E. OA0603

Feuer, C. OA2104

Figueroa, M.I. OA1303

Fiore‐Gartland, A. OA1105

Fisher, K. OA2007LB

Fonner, V. OA2403

Fontenot, J.A. OA0603

Ford, S. OA2306

Forsback, A.‐P. OA0206

Fox, A. OA0705

Fox, C. OA1202

Frank, I. OA0306, OA1207LB

Franks, J. OA2002

Frattini, N. OA1006

Fray, E. OA1806

Fredricks, D. OA1807LB

Frempong, J. OA2202

Friedland, B.A. OA1605

Furch, B. OA1205

Furlott, J. OA0604

### G

Gómez, L. OA2303, OA2305

Gagliardini, R. OA0205

Gaiha, G.D. OA0302

Galarraga, O. OA0506

Galiwango, R. OA2205

Galkin, A. OA0303

Gall, J. OA1206

Gallardo‐Cartagena, J. OA0207, OA0605

Gama, L. OA0605, OA0606, OA0607LB, OA1107LB, OA1206

Gamble, T. OA0606, OA0607LB, OA1107LB

Gammon, J.M. OA2202

Gandhi, M. OA0503, OA0507LB

Gao, F. OA0607LB

García, J. OA1303

Garcia, J. OA0804LB

Gardner, E. OA1602

Garg, A. OA0802LB

Garner, A. OA0906

Garnett, P. OA1807LB

Garrett, N. OA0306

Gati Mirembe, B. OA2302

Gay, C.L. OA0606, OA1107LB

Gebengu, A. OA2304

Gebrehiwot, T.S. OA1405

Gelderblom, H. OA1105

George, G. OA2304

Georgetti Gomez, L. OA0507LB

Geraghty, D.E. OA1105

Geretz, A. OA0703

Gianotti, N. OA0205

Gichuru, E. OA1307LB

Gifford, A. OA1703

Gilbert, P.B. OA0306, OA0807LB, OA1105

Gill, K. OA0803LB

Gillespie, K. OA0607LB

Giorgi, E.E. OA0306, OA0807LB, OA1503

Giovenco, D. OA1003, OA1705

Girardi, E. OA0205

Gitau, E. OA2103

Giusti, P. OA0404

Glantz, H. OA1807LB

Glick, S. OA1306

Goepfert, P. OA1104, OA1204

Goepfert, P.A. OA0306

Goetzmann, J.E. OA0603

Gomez, R. OA1807LB

Gomez‐Feliciano, K. OA2002

Gordon‐Johnson, K.‐A. OA0103

Gorman, J. OA2602

Gottert, A. OA1605

Goulart, S.P. OA1107LB

Gounder, K. OA0807LB

Govindasami, M. OA1905

Goyal, K. OA2503

Grant, S. OA1205

Grattoni, A. OA1607LB

Gray, G. OA2407LB

Gray, G.E. OA0306, OA1105

Grebe, E. OA0807LB

Grinsztejn, B. OA0207

Grove, D. OA0605

Gruber, J. OA2405

Grunenberg, N. OA0306

Gu, Y. OA2004

Guenaga, J. OA0303, OA2603, OA2605

Guha, S.K. OA1504

Gumbi, P. OA2203

Gupta, R. OA0107LB

Gupta, R.K. OA0806LB

Guthrie, B. OA2103

Gwavava, E. OA2402

### H

Habib, R. OA2607LB

Haddad, L. OA0802LB

Haddad, L.B. OA1605

Hahn, B. OA2607LB

Hahn, W. OA1204, OA1205, OA1207LB

Haigwood, N. OA1802

Haines, D. OA2306

Halvas, E.K. OA0507LB

Hamby, T. OA1203

Han, Z. OA2004

Hanscom, B. OA2306

Hansen, Z. OA1203

Hao, J. OA1306, OA1905

Haque, M.R. OA0706LB

Harrington, E. OA2404

Hart, C. OA0803LB

Hartley, J. OA0806LB

Hasan, Z. OA1102

Hasselschwert, D.L. OA0603

Hasson, J.M. OA0706LB

Hausler, H. OA1403, OA2502

Hayes, A. OA1405

Haynes, B. OA1202, OA1804

Hazra, A. OA0507LB

Heaps, A.L. OA0507LB

Hearps, A. OA2203

Hedberg, T. OA0507LB

Heffron, R. OA1306, OA1905

Heffron, R.A. OA0202

Heise, M.J. OA0503

Hemmerling, A. OA2206

Henderson, R. OA1202

Hendriks, J. OA1105

Hendrix, C. OA0204

Henry, A. OA1206

Heptinstall, J. OA0306, OA0607LB, OA1205

Herrera, C. OA1103

Herring, G.B. OA1602

Hessell, A. OA0604, OA1802

Hidalgo, J. OA0605

Hightow‐Weidman, L. OA0204

Hill, E. OA1307LB

Hill, L. OA1302

Hinestrosa, F. OA0203

Hinojosa, J. OA0605

Hoang, T. OA0103

Hoege, D. OA1302

Holker, J. OA1902

Hollenberg, E. OA0804LB

Holloway, I. OA0906

Holmes, C. OA1302

Holmes, S. OA2206

Homony, B. OA0203

Hong, C. OA0906

Hope, T.J. OA0603, OA0706LB

Hora, B. OA1202

Hosseinipour, M. OA1302, OA2306

Hosseinipour, M.C. OA0306

Howett, R. OA0904

Htet, L. OA1906

Hu, F. OA1203

Hu, X. OA1805

Hua, S. OA0702

Huang, W. OA0505

Huang, Y. OA0606, OA0607LB, OA0807LB, OA1807LB

Huibner, S. OA2205

Humeau, L. OA0302

Humphries, H. OA2203

Hural, J. OA0307LB, OA0807LB, OA1105, OA1206

Hurt, C.B. OA0606, OA1107LB

Hussain, F. OA2206

Hussain, S. OA2206

Hutter, J. OA1807LB

Hwang, P. OA0202, OA0203

Hyrien, O. OA0606, OA1107LB, OA1204, OA1205, OA1807LB

### I

Iannantuono, M.V. OA1002, OA1303

Ikuzo, B. OA1706

Imai‐Eaton, J.W. OA0106

Irungu, E. OA2402

Isaac, J. OA1803, OA1805

Ishmail, H. OA0904, OA1905

Issah, Y. OA1805

Izumi, T. OA0703

### J

Jackson, B. OA0203

Jain, M. OA1902

Jalil, E.M. OA2002

Jama, N. OA2406

Jamieson, L. OA1702

Janamnuaysook, R. OA1004

Janes, H. OA0307LB, OA1207LB

Jardine, J. OA0604

Jaspal, R. OA1703

Jaspan, H. OA2203

Jayal, P. OA1504

Jeckonia, P. OA2402

Jiane, B. OA1905

Jiang, C. OA1202

Joag, V. OA2205

John‐Stewart, G. OA2303

Johnson, L. OA1702

Johnson, L.F. OA0106

Jonas, K. OA2407LB

Jones, B. OA1703

Jones, M. OA1107LB

Jongrakthaitae, S. OA0305

Jooste, I. OA2504

Joseph Davey, D. OA2304

Joshi, N. OA0202

Juma, E. OA2103

Juraska, M. OA0807LB, OA1105

Justman, J. OA1606

### K

K'Orimba, K. OA2402

Kaabi, O. OA1602

Kabongo, J. OA2403

Kadama, H. OA1306, OA2402

Kaku, Y. OA1102

Kalams, S. OA0607LB, OA1104

Kale, D. OA1504

Kaleebi, J. OA2006

Kaleebu, P. OA1505, OA1506

Kalonji, D. OA2306

Kamble, P. OA1504

Kannan, A. OA2206

Kansiime, R. OA1306

Kaplan, A. OA0804LB

Kaplan, R. OA0203

Karandikar, K. OA1504

Karita, E. OA1505

Karlsson Hedestam, G. OA2605

Karlsson Hedestam, G.B. OA2603

Karuna, S. OA0605

Karuna, S.T. OA0606, OA0607LB, OA1107LB

Kasarpalkar, N. OA1504

Kasozi, D. OA2102

Kassim, S. OA0607LB

Katende, J. OA1506

Kato, L. OA1506

Katpara, S. OA2606

Katumba, A. OA2504

Kaul, R. OA2205

Kaushal, G. OA0206

Kavanagh, M. OA2105

Kawanga, L. OA0504, OA1005, OA1305, OA2005

Kawoozo, B. OA2205

Kayirangwa, E. OA1706

Kayisinga, J.D.D. OA1706

Keawboon, B. OA0305

Keefer, M. OA0306, OA1206

Keefer, M.C. OA1207LB

Kelley, C. OA0607LB, OA1107LB

Kelley, C.F. OA1602

Kelly, C. OA1204

Kelly, S. OA0806LB

Kelly, S.L. OA0106

Kemigisha, D. OA1404

Kemp, S. OA0806LB

Kew, L. OA0904, OA1905

Keyes, T. OA0307LB

Kgoa, R. OA0904, OA1905

Khan, T. OA0502

Kher, G. OA2604

Kibalama Ssemambo, P. OA1404

Kibet, C. OA1106

Kibuuka, J. OA1306

Kidenya, B. OA1402

Kilembe, W. OA1505

Killick, M.A. OA0602

Kim, D. OA0704

Kim, H.‐Y. OA0106

Kim, J. OA1203

Kimata, J. OA1607LB

King, H. OA1806

Kingston, H. OA2103

Kinuthia, J. OA2303, OA2305

Kiptinness, C. OA2003

Kirchhoff, F. OA0703

Kiruki, M. OA2403

Kirungi, R. OA1404

Kisaakye, L. OA0902

Kisone, V. OA0902

Kisubi, N. OA1903

Kitchin, D. OA1505

Klausner, J.D. OA2304

Knodel, S. OA1103

Komuhangi, L. OA1306

Konda, K. OA0405, OA1704

Kondapi, A. OA1504

Korber, B. OA0807LB, OA1503

Kosgei, J. OA0607LB

Kosmider, E. OA0807LB

Koss, C.A. OA0507LB

Kotze, P. OA0202

Koup, R. OA0606, OA1206

Krebs, S. OA0305, OA1203

Krebs, S.J. OA0704

Kriel, W. OA0802LB

Kubjane, M. OA1702

Kublin, J. OA1205

Kublin, J.D. OA0306

Kublin, J.G. OA1105

Kulp, D. OA2607LB

Kumar, S. OA2606

Kuncze, K. OA0507LB

Kunene, P. OA2406

Kuo, C. OA2407LB

Kuwata, T. OA1102

Kwena, Z. OA2404

Kwesigabo, G. OA1402

Kwon, D. OA2202, OA2206

Kwong, P. OA1206

Kwong, P.D. OA2602

Kyambadde, P. OA1306, OA1903

Kyaw, M.K. OA1906

Kyokushaba, J. OA0902

### L

Labuschagne, P. OA0807LB

Lagat, H. OA2404

Laher, F. OA0306, OA0607LB

Lake, J.E. OA2002

Lam, E. OA2202

Lama, J. OA0605

Lambdin, B. OA1306

Lambson, B. OA1502

Landais, E. OA0604, OA1505

Landovitz, R.J. OA0203

Lange, C. OA1203

Lanini, S. OA1006

Laufer, D. OA1205

Lavreys, L. OA1105

Le Grand, R. OA0702

Lee, C. OA1203

Lee, E. OA1804

Lee, J.H. OA0604

Leggat, D. OA1203

Lei, L. OA0303

Leino, L. OA0206

Lemos, M.P. OA1107LB, OA1207LB, OA1807LB

Lenderking, W.R. OA0502

Lenn, M. OA2404

Leonard, B. OA0805LB, OA1003

Li, H. OA0604

Li, L. OA1105

Li, Q. OA1203

Li, S.S. OA0306, OA1105

Li, W. OA2603

Li, Y. OA0303

Lifson, J. OA1806

Likindikoki, S. OA1402

Lin, W. OA0204

Lin, X. OA2603, OA2605

Lin, Z. OA2607LB

Lint, A. OA1304

Liu, A. OA0502, OA2002

Liu, C. OA2205

Liu, S. OA0306

Liu, Y. OA0505, OA1607LB

Lodha, R. OA2606

Logie, C. OA1903

Lolatto, R. OA1603

Lombard, C. OA2407LB

Lopez Malizia, Á. OA1002

Lora, M. OA0804LB

Lorenzo‐Redondo, R. OA0706LB

Losso, M.H. OA0207

Loufty, M. OA1303

Louis, M. OA0603

Louis‐Charles, K. OA2403

Loutet, M. OA1903

Louw, C. OA0202

Lu, H. OA1205

Lu, S. OA0306

Lu, X. OA1202

Lu, Y. OA1807LB, OA2004

Lucas, I.L. OA1705

Lucas, J. OA0605, OA2002

Lucas, T. OA0102

Lucio, V.D.C. OA0207

Ludwig, J. OA0807LB

Ludwig‐Barron, N. OA2103

Luo, Y. OA2004

Luthra, K. OA2606

Lyamuya, E. OA1402

Lynch, S. OA2105

### M

Maboa, O. OA2403

Mabombo, K.N. OA1502

MacDonald, P. OA0803LB, OA0805LB

Macedo, B.L. OA0402

Machava, R. OA0103

Machemedze, D. OA0903

Machmach Leggat, K. OA0704

Maci, C. OA1603

MacPhee, K. OA1205

MacRae, J. OA0605

Madeddu, G. OA0205

Maenza, J. OA1207LB, OA1807LB

Magaret, C.A. OA0807LB

Maggiolo, F. OA0205

Mahaka, I. OA2402

Maheu‐Giroux, M. OA0106

Mahomed, S. OA0607LB, OA1107LB

Mai, J. OA1607LB

Mainardi, I. OA1603

Makhado, Z. OA1505

Makhale, L. OA0903

Malaba, R. OA0103

Malamba, S.S. OA1706

Mann, P. OA1807LB

Mannheimer, S.B. OA0606, OA1107LB

Mansoor, L.E. OA2302

Mapukata, P. OA0805LB

Maraba, M. OA0903

Marchetti, G. OA0205, OA1006

Marelli, C. OA0205

Marfil, S. OA0304

Margaret, C. OA1105

Marini‐Macouzet, C. OA1205, OA1207LB

Marone, R. OA1002

Maroney, K. OA1104

Marshall, K. OA2504

Martin Beem, J. OA1202

Martin, T. OA1206

Martin‐Hughes, R. OA0106

Martindale, L. OA0102

Martinelli, E. OA0706LB

Marwa, M. OA2303

Marzinke, M. OA2306

Marzinke, M.A. OA2002

Masa, R. OA1705

Masabanda Perez, J.C. OA1604

Mascola, J. OA0303, OA1807LB

Mashauri, A. OA0103

Mason, R. OA1806

Masson, L. OA2203

Mastroianni, C. OA1006

Masyuko, S. OA2103

Matassoli, F. OA1206

Matenjwa, N. OA1403, OA2502

Mathew, C. OA2306

Mathews, C. OA2407LB

Mathola, N. OA0805LB, OA1003

Mathur, S. OA1605

Matos, C.M. OA0402

Matsushita, S. OA1102

Matswake, N. OA1905

Matten, D. OA0807LB

Mattioli, S. OA1006

Matyas, G. OA1203

Mavigner, M. OA1806

Mayanja, Y. OA1506

Mayer, B.T. OA0807LB

Mayer, K.H. OA0207, OA2002

Mayo, A. OA2302, OA2402

Mayorga‐Munoz, F. OA0503

Maziku, E. OA1402

Mazzotta, V. OA1006

Mbochi, G. OA0803LB

Mbogo, L. OA2103

Mbuagbaw, L. OA1903

Mbugua, S. OA1307LB

McBride, M. OA1205

McClinton Appollis, T. OA2407LB

McDermott, A.B. OA1807LB

McDermott, D. OA1703

McElrath, J. OA0307LB, OA1205

McElrath, M.J. OA0306, OA0607LB, OA1105, OA1204, OA1207LB, OA1807LB

McGovern, M. OA2604

McGuire, C. OA2403

Mcingana, M. OA1403, OA2502

McKee, K. OA1206

McKenney, K. OA0604

McKinnon, L.R. OA2204

McLoughlin, J. OA1403, OA2502

McRaven, M.D. OA0603, OA0706LB

Medina Matamoros, H.J. OA1604

Mehou‐Loko, C. OA2203

Meijomil, M. OA1002

Mekuria, L.A. OA1405

Melillo, S. OA2102

Mellors, J. OA0703

Mellors, J.W. OA0507LB

Meng, G. OA2004

Merrill, D. OA0502

Meyer‐Rath, G. OA1702

Mezzio, D. OA2405

Mgodi, N. OA2302

Mhakakora, T. OA2406

Mhlanga, D. OA1505

Mhlanga, F. OA0607LB

Mi, G. OA0505

Michael, N. OA0703

Michaels, J. OA0803LB

Mielke, D. OA0307LB, OA1503

Mijumbi, A. OA2403

Minalga, B. OA2104

Minnis, A. OA0803LB

Mirembe, B.G. OA1404

Mishra, M. OA1906

Mishra, S. OA0106

Mitchell, C. OA2206

Mitchell, J. OA1704

Mitchell, K.M. OA0106

Mize, G. OA1807LB

Mkhize, N. OA0607LB

Mmbaga, B. OA1402

Mngadi, K. OA0306, OA1105

Mogaka, F. OA2404

Mogaka, J. OA2303

Mogere, P. OA2003

Moges‐Banks, R. OA1602

Mohapatra, S. OA1504

Mokhele, T. OA0903

Molinos‐Albert, L.M. OA0304

Mondal, S. OA1504

Mondi, J. OA0103

Monroe‐Wise, A. OA2103

Montefiori, D. OA0607LB, OA1202, OA1204, OA1205, OA1206, OA1207LB, OA1503, OA1804

Montefiori, D.C. OA1803, OA1805

Montenegro‐Idrogo, J. OA0605

Montgomery, E. OA0803LB

Moodie, Z. OA0306, OA1205

Moodley, A. OA1203

Moodley, R. OA0502, OA1705

Mookherji, S. OA1405

Moore, J.P. OA1803, OA1805

Moore, P. OA1505

Moore, P.L. OA1502

Morando, N. OA1002

Morano, N.C. OA2602

Morioka, H. OA1102

Morris, L. OA1502

Morris, N. OA2407LB

Morrison, S. OA1307LB

Morroni, C. OA2304

Motlou, T. OA1505

Mpendo, J. OA2205

Mposula, H. OA1905

Mshana, S.E. OA1402

Mthimkhulu, N. OA2406

Mubezi, S. OA1706

Muccini, C. OA1603

Mudiope, P. OA1306

Muema, D. OA0807LB, OA1106

Mufhandu, H.T. OA1502

Mugaba, S. OA1506

Mugambi, M. OA2404

Mugo, N. OA1307LB, OA2003

Mugwanya, K. OA1307LB

Muhammad, A. OA1902

Mujugira, A. OA1306

Mukenyi, E. OA2305

Mukherjee, J. OA2503

Mukherjee, S. OA1504

Mullick, R. OA1504

Mullick, S. OA2406

Mullins, J.I. OA0807LB, OA1105

Mundende, P. OA1305

Muramatsu, H. OA1202

Muriuki, C. OA1106

Murrell, B. OA0604

Murugavel, K. OA1504

Musangulule, J. OA1305

Mushy, S. OA1402

Musodza, Y. OA0607LB

Musonda, M. OA0504, OA1005, OA1305, OA2005

Mussa, A. OA2304

Mustanski, B. OA2507LB

Muthui, M. OA1106

Mutseta, M. OA0103

Muwanga, M. OA2205

Muwonge, T. OA1306

Mvududu, R. OA0105

Mwakilasa, M.T. OA1402

Mwangi, J.W. OA2506

Mwanza, J. OA2005

Mwelase, N. OA0202

Mwesigwa, R.C.N. OA1706

Mwijage, A. OA1402

Mwita, C. OA2504

Myer, L. OA0105

Mzindle, N. OA1505

### N

N'Guessan, K.F. OA0704

Naidoo, A. OA1807LB

Naidoo, L. OA0202, OA2302

Naidoo, N. OA2402

Naidoo, N.P. OA2406

Naidoo, T. OA1505

Naing, K.P. OA1906

Nakabiito, C. OA1404

Nakabugo, L. OA1306

Nakitende, M. OA1306

Nakkazi, E. OA2504

Nakyanzi, T. OA1404

Namakula, A. OA0902

Namatovu, T. OA2205

Nambi, F. OA1306

Nanvubya, A. OA2205

Nanyonga, S. OA1404

Nanziri, S.C. OA1404

Narpala, S. OA1206, OA1807LB

Nava, A. OA1303

Ndeikemona, L. OA0103

Ndengo, E. OA1706

Ndhlovu, A. OA1305

Ndhlovu, A.P. OA0504, OA1005, OA2005

Ndlovu, N. OA0207, OA0803LB, OA0904, OA1905

Nduati, E. OA1106

Ndung'u, T. OA0807LB, OA1505

Nehete, P. OA1607LB

Nelson, A. OA1803, OA1804, OA1805

Nelson, K.L. OA0502

Nemazee, D. OA0303

Nepo, E. OA0404

Newman, A. OA1202

Ngo, J.P. OA0905

Ngumbau, N. OA2303, OA2305

Ngure, K. OA1307LB, OA2003, OA2303

Nguyen, C. OA2405

Nguyen, J.Q. OA0503

Nguyen, M. OA1605

Nguyen, P. OA1203

Nijs, S. OA1105

Nitayaphan, S. OA0305, OA0306, OA0807LB

Niyibeshaho, M. OA2403

Nkongoane, M. OA2403

Nkwoh, K.T. OA0103

Noel‐Romas, L. OA1103

Noguchi, L. OA2302

Nomvuyo, M. OA0803LB

Nonyane, B.A.S. OA1403

Nozza, S. OA1006, OA1603

Nuttall, J. OA0802LB

Nyabiage, J. OA2305

Nyagah, W. OA0104

Nyagonde, N. OA0103

Nyerere, B. OA2404

Nyirenda, R. OA1302

Nyumbu, M. OA0504, OA1005, OA2005

Nzano, B. OA2506

Nzuki, I. OA1904

Nzuve, P. OA1307LB

### O

O'Connor, C.J. OA0602

O'Dell, S. OA0303, OA1206

Oakes, M. OA0604

Obidoa, C. OA1407LB

Ochieng, B. OA2305

Ochsenbauer, C. OA1807LB

Odhiambo, B. OA2303

Odong Lukone, S. OA1903

Odoyo, J. OA2404

Odoyo‐June, E. OA0103

Ogbuagu, O. OA0207

Ojera, S. OA1406

Okazaki, K. OA1102

Okech, B. OA2205

Okello, P. OA2003

Okochi, H. OA0507LB

Okumu, M. OA1903

Olia, A. OA1206

Oliva, A. OA1006

Olouch, T.O. OA1706

Oluka, G. OA1506

Oman, A. OA1104

Omollo, V. OA2404

Ong, J.J. OA0505

Operario, D. OA0506, OA1705

Ordonez, T. OA1802

Orellana, E.R. OA0403

Orellano, G. OA1303

Orrell, C. OA1807LB

Ortblad, K.F. OA2003, OA2404

Ortiz, Z. OA1303

Osei‐Tutu, N. OA1407LB

Oskarsson, J. OA0503

Ota, M. OA0303

Ota, T. OA0303

Owidi, E. OA2003

Owuor, S. OA1106

Oyugi, E. OA0103

Ozituosauka, W. OA1302

Ozorowski, G. OA1205, OA1803, OA1805, OA2603, OA2605

### P

Padilla, M. OA1602

Paez, C. OA1206, OA1807LB

Paez, C.A. OA0607LB, OA1107LB

Pahe, C. OA1904

Palanee‐Phillips, T. OA0803LB, OA0904, OA1905, OA2302

Pallesen, J. OA2607LB

Pancera, M. OA2604

Panchia, R. OA2306

Pande, G. OA1306

Pandey, S. OA1802

Pando, M.Á. OA1002

Pantaleo, G. OA0306

Papathanasopoulos, M.A. OA0602

Paquin‐Proulx, D. OA0703, OA0704

Pardi, N. OA1202

Paredes, R. OA1103

Parenti, T. OA2105

Parera, M. OA1103

Parikh, U.M. OA0507LB

Park, D. OA2205

Park, Y. OA2607LB

Parks, K. OA1205

Parks, K.R. OA1204

Parmley, L. OA2406

Parshall, C. OA2105

Pascoe, S.S. OA0706LB

Passmore, J.‐A. OA2203

Patel, V. OA1504

Pau, M.G. OA1105

Peck, M. OA0103

Pedone, M. OA1006

Peet, M. OA0206

Pegu, A. OA0604

Pensiero, M. OA0607LB, OA1107LB

Perez‐Brumer, A. OA0405, OA0406

Perlotto, S. OA0502

Permar, S. OA1803, OA1804

Permar, S.R. OA1805

Peters, R.PH. OA2304

Petrova, Y. OA1203

Phanuphak, N. OA0207, OA0703, OA1004, OA2204

Phate‐Lesihla, R. OA2402

Phelps, M. OA2202

Phillips II, G. OA2507LB

Phiri, D. OA0504, OA1005

Phiri, S. OA1302

Phuang‐Ngern, Y. OA0305, OA2204

Phyo, P.P. OA1906

Pierson, T. OA1206

Pike, C. OA0805LB

Pilgrim, N. OA0502

Pillay, D. OA0806LB

Pinedo, Y. OA0203

Pinheiro, L. OA0402

Pintye, J. OA2303, OA2305, OA2404

Pitisuttithum, P. OA0305, OA0306

Piwowar‐Manning, E. OA0607LB

Plagianos, M. OA0802LB

Plank, R.M. OA0202, OA0203

Pleaner, M. OA2403

Pletnev, S. OA2602

Polakowski, L.L. OA0606, OA1107LB

Pollara, J. OA1804

Polonis, V. OA1203

Pons‐Faudoa, F. OA1607LB

Poteat, T. OA1304, OA2002

Potts, A. OA1407LB

Powell, R. OA0705

Pozharski, E. OA0303

Prabhakaran, M. OA1807LB

Pradenas, E. OA0304

Pranjape, R. OA1504

Prasad, C. OA1504

Predreño‐López, N. OA0304

Prochazka, M. OA0104

Prodger, J. OA2205

Proulx‐Burns, L. OA0607LB

Puren, A. OA2407LB

Purnell, C. OA0403

Pyo, C.‐W. OA1105

Pyone, T.Y. OA1906

### R

Raccagni, A.R. OA1603

Radix, A. OA1304

Radix, A.E. OA2002

Ragone, L. OA1304

Rakasz, E. OA0604

Ramirez, J.C. OA0406

Rana, R. OA1902

Rao, A. OA1403

Rao, M. OA1203

Rasebotsa, S. OA1505

Rathore, S. OA2204

Ratmann, O. OA0806LB

Ratnaratorn, N. OA2204

Rechkina, E. OA1307LB

Reddy, K. OA0803LB, OA0904, OA1905

Reed, J. OA1802

Reeves, D.B. OA0807LB

Regenold, S. OA0606, OA1107LB

Reisner, S. OA1304

Reisner, S.L. OA2002

Relouzat, F. OA0702

Remera, E. OA1706

Renquist, A. OA2102

Repossi, R. OA1006

Rerknimitr, R. OA2204

Rerks‐Ngarm, S. OA0306

Restar, A. OA0506

Reyes Diaz, E.M. OA1704

Rice, W.S. OA0505

Richards, K. OA2505

Ricketts, J. OA0103

Riddler, S. OA1205

Rinehart, A. OA2306

Rittiroongrad, S. OA1103

Roark, R.S. OA2602

Robb, M. OA0704

Robb, M.L. OA0807LB

Robben, P.M. OA1203

Robertson, M.N. OA0202, OA0203

Rodolph, M. OA0104

Rodrigues, G.M. OA0402

Rodrigues, S.F.M. OA0402

Rodriguez de la Concepción, M.L. OA0304

Roederer, M. OA1806

Rogers, K.A. OA0603

Rolland, M. OA0807LB

Romanelli, C.M. OA1303

Romero, M. OA1303

Rompay, K.K. OA1805

Rono, B. OA2404

Rooney, J. OA1307LB, OA2002, OA2306

Rose, M. OA1104

Rose, S. OA2306

Rosen, J.G. OA1403, OA2106, OA2502

Rosenberg, N. OA1606

Ross, J. OA0904, OA1004

Rossenkhan, R. OA0807LB

Rossotti, R. OA1006

Rota, G. OA2404

Rouphael, N. OA1207LB

Rousseau, E. OA0805LB, OA1003, OA2304

Rouzeau, R. OA0604

Rovirosa, C. OA0304

Roxby, A.C. OA0606

Rucinski, K. OA1403

Rutishauser, R.L. OA0302

Rwabasira, G.N. OA1706

### S

Sáez‐Cirión, A. OA0702

Sacdalan, C. OA0703, OA2204

Sacha, J. OA1802

Saetun, P. OA2204

Safa, H. OA0507LB

Safrit, J. OA1806

Saha, P. OA2503

Saidi, F. OA1302

Saina, M. OA1307LB

Sajjaweerawan, C. OA2204

Salazar, V. OA1303

Sales, J.M. OA0505, OA1605

Sallusso, D. OA1303

Sam‐Agudu, N. OA1606

Sam‐Urom, L. OA2505

Sambai, B. OA2103

Samer, S. OA0706LB

Sanchez Thomas, D. OA1107LB

Sanchez, J. OA0605

Sanchez, T. OA1705

Sanders, R.W. OA1803, OA1805

Sandoval, M.M. OA1303

Sango, A. OA2206

Sangwayire, B. OA1706

Santoro, M.M. OA0205

Sanzone, A. OA1202

Sassaman, K. OA0503

Satsangi, A. OA1902

Saucedo Mérida, B. OA0403

Saunders, K. OA1202, OA1804

Sawant, S.S. OA0306

Sawe, F. OA0807LB

Scarsi, K. OA2306

Schaafsma, T. OA1307LB

Scheckter, R. OA2302

Schief, W. OA1205

Schleich, F. OA2603, OA2605

Schmitzberger, L. OA0307LB

Schuetz, A. OA0305, OA1103, OA2204

Schuitemaker, H. OA1105

Schuster, D. OA0307LB

Schwartz, S. OA1302, OA1403, OA2502

Scott, H. OA0607LB, OA1204

Seaton, K. OA0606

Seaton, K.E. OA0306, OA0607LB

Seese, A. OA1205

Segawa, I. OA2403

Seid Ebrahim, E. OA1405

Sein, Y. OA1106

Seleme, J. OA0103

Sendaula, E. OA2006

Serantes, D. OA1002

Serebryannyy, L. OA1206

Serti Chrisos, E. OA0305

Serti, E. OA1203

Serwanga, J. OA1505, OA1506

Seth, K. OA2503

Severini, G. OA2204

Sewall, L.M. OA1803, OA1805

Shaaban, M.A. OA0706LB

Shah, A. OA1206

Shahin, L. OA0604

Shakery, T. OA2204

Shang, X. OA0303

Shao, D. OA0807LB

Shao, Z. OA2205

Shapiro, L. OA2602

Sharma, A. OA1806, OA2105

Sharma, M. OA2404

Sharma, N. OA1504

Sharma, S. OA2606

Shastri, J. OA1504

Shaw, G. OA0604, OA2607LB

Shelton, K. OA1607LB

Shen, M. OA1207LB

Shen, X. OA1803, OA1804, OA1805

Shetty, S. OA1605

Shiels, M. OA0503

Shih, J. OA2206

Shipp, L. OA1403, OA2502

Shook‐Sa, B. OA1606

Shroff, S. OA0705

Shukla, P. OA0507LB

Siegel, M. OA0607LB, OA1107LB

Siegler, A.J. OA0505

Sigcu, N. OA0904, OA1905

Sikwese, S. OA1302

Silhol, R. OA0106

Siliciano, J. OA1806

Siliciano, R. OA1806

Silva De Assis, T. OA0604

Silva, A.Q. OA0402

Silva‐Santisteban, A. OA0405, OA0406, OA1704

Silvestri, G. OA1806, OA2603, OA2605

Simeone, A. OA1607LB

Sinchai, K. OA1004

Sincomb, T. OA0604

Singh, B. OA2407LB

Singh, C. OA1403, OA2502

Singh, M.K. OA1902

Singh, N. OA2302, OA2306

Singh, S. OA2606

Singh, V. OA1806

Singh, Y. OA1807LB

Sinkele, W. OA2103

Sirili, N. OA1402

Sisel, H. OA2403

Sitikornvorakul, K. OA1004

Siva, S. OA2302

Sleesman, J. OA1203

Slike, B. OA0305, OA0704, OA1203

Smith Jr, M. OA1705

Smith, N. OA0603

Smith, P. OA1306

Sobieszcyzk, M. OA1204

Sobieszczyk, M.E. OA0606, OA1107LB

Soe, T. OA1906

Soita, D. OA1406

Sok, D. OA0604, OA1504, OA1505

Sokhela, Z. OA0903

Solai, L. OA0802LB

Solari, K. OA0406

Soothoane, R. OA2402

Soto‐Torres, L. OA0605, OA2302, OA2306

Sowers, K. OA2607LB

Spiegel, H. OA0607LB, OA1107LB, OA2002

Spinelli, M.A. OA0503

Spooner, E. OA2306

Srikrishnan, A.K. OA1504

Srinivasan, S. OA1807LB

Srivas, S. OA1504

Srivatsan, V. OA2105

Ssemaganda, A. OA2204

Ssetaala, A. OA2205

Stalls, V. OA1202

Stamatakis, C.E. OA1706

Stamatatos, L. OA1204, OA2604

Standfield‐Oakley, S. OA1503

Stanfield‐Oakley, S. OA0307LB

Stannah, J. OA0106

Stassek, L. OA0502

Stegmueller, D. OA0505

Steingo, J. OA2502

Stephenson, K.E. OA0606, OA1107LB

Stern, J. OA2303

Stevens, O. OA0106

Stevenson, M. OA1304

Steytler, J. OA0802LB

Stieh, D.J. OA1105

Stone, E.M. OA2106

Stone, J. OA0106

Stover, J. OA0106

Stranix‐Chibanda, L. OA0606, OA1606

Strauss, M. OA2304

Stuurman, R. OA1905

Subedar, H. OA2402

Sukhumvittaya, S. OA0305, OA2204

Sullivan, P. OA0905

Sullivan, P.S. OA0505, OA1602

Sun, L. OA2004

Sunguya, B. OA1402

Sutar, J. OA1504, OA1505

Swafford, I. OA0704

Swan, E. OA1105

Swygard, H. OA0502

Symul, L. OA2206

Szydlo, D.W. OA2302

### T

Tachedjian, G. OA2203

Tailor, J. OA0104

Takalani, A. OA0607LB

Takuva, S. OA0607LB, OA1107LB

Tam, Y. OA1202

Tanser, F. OA0806LB

Tao, L. OA2405

Tarrés‐Freixas, F. OA0304

Tauras, M. OA2102

Tavelli, A. OA1006

Taylor, O. OA1103

Tesoro, D. OA1006

Thaung, Y.M. OA1906

Thigpen, M. OA1203

Thomas, R. OA0703, OA2204

Thomas, Y. OA0603, OA0706LB

Thomson, S. OA1807LB

Thu, L.A. OA1906

Thuo, N. OA2003

Thuruthiyil, C.T. OA0706LB

Tian, M. OA1202

Tindale, I. OA0607LB

Tip, S.L. OA1906

Tobian, A. OA2205

Toledo, C. OA0103

Tomai, M. OA1202

Tomaka, F. OA1105

Tomaras, G. OA0307LB, OA1204, OA1205, OA1207LB, OA1503

Tomaras, G.D. OA0306, OA0606, OA0607LB, OA1105

Torjesen, K. OA2402

Torti, C. OA1006

Tragonlugsana, N. OA0305

Trahey, M. OA0607LB

Trautmann, L. OA2204

Travill, D. OA0903

Tressler, R. OA0605

Tsegaye, D.A. OA1405

Tyssen, D. OA2203

Tzindoli, H. OA2403

### U

Ul Hadi, S. OA2503

Umuhoza, N. OA1706

Unger, J. OA2303

Usachenko, J. OA1802

Uwineza, J. OA1706

### V

Valencia, R. OA1602

Valentine‐Graves, M. OA1705

Van der Westhuizen, A. OA2505

van Dorsten, R. OA1505

van Duijn, J. OA1105

Van Pelt, A. OA2507LB

Van Rompay, K. OA1802

Van Rompay, K.K. OA1803

Van Rompay, K.K.A. OA1804

Vannappagari, V. OA1304

Vanto, O. OA0805LB

Vasan, S. OA0305, OA0306, OA0703, OA1103, OA1203, OA2204

Vekatayogi, S. OA1803

Velloza, J. OA0904

Venkatayogi, S. OA1202

Verde Hashim, C. OA0104

Vermund, S. OA0107LB

Vickerman, P. OA0106

Villinger, F. OA0706LB

Villinger, F.J. OA0603

Villon, A. OA0405

Vimonpatranon, S. OA0305, OA2204

Visser, J. OA0802LB

Voillet, V. OA1207LB

Volcic, M. OA0703

Vu, V.H. OA0905

Vundhla, P. OA2304

### W

Wafula, R. OA2402

Wairimu, N. OA2003

Walker, B.D. OA0807LB

Wallace, M. OA1003

Walmsley, S. OA1303

Walsh, S.R. OA0606, OA1107LB, OA1204

Walworth, C. OA0507LB

Wandera, M.G. OA1706

Wang, H. OA2602

Wang, S. OA0604, OA2602

Wara, N. OA0105

Ward, A. OA1205, OA2605

Ward, A.B. OA1803, OA1805, OA2603

Warren, D. OA0502

Warren, M. OA0104

Watanabe, J. OA1802

Watoyi, S. OA2303, OA2305

Watson, D.L. OA2002

Webster, J. OA0204

Weiner, D. OA0302, OA2607LB

Weiner, J. OA0607LB

Weir, B.W. OA0905

Weisgrau, K. OA0604

Weissman, D. OA1202, OA1804

Wellington, T. OA1203

West, R. OA1302

Westfall, D.H. OA0807LB

Weyer Johnson, L. OA0403

Wiehe, K. OA1202, OA1803, OA1804

Wilcher, R. OA2402

Willems, W. OA1105

Williams, L.D. OA0306

Williams, W. OA1202

Williams, W.B. OA1803

Williamson, C. OA0807LB, OA1105, OA1502

Wilson, D.P. OA0106

Wilson, I. OA2603, OA2605

Wilson, J. OA2203

Wilson, R. OA2603, OA2605

Wilson‐Barthes, M. OA0506

Win, T.T. OA1906

Wirtz, A. OA1304

Wirtz, A.L. OA0905

Wong, B. OA2502

Wood, T. OA1306

Woodward‐Davis, A. OA1205

Wu, L. OA1307LB

Wyatt, R. OA0303, OA2605

Wyatt, R.T. OA2603

### X

Xia, S.‐M. OA1202

Xu, J. OA2206, OA2602

### Y

Yac, J. OA0403

Yacovone, M. OA0606, OA0607LB, OA1107LB, OA1204

Yamauchi, S. OA1102

Yang, J. OA2405

Yang, X. OA0705

Yegorov, S. OA2205

Yen, C. OA0606, OA1107LB

Yoon, A. OA1103

Young, A. OA1606

Yssel, A. OA0807LB

Yu, C. OA0606, OA0607LB, OA1107LB

Yu, F. OA0505

Yum, L. OA0703

### Z

Zachry, W. OA2405

Zalaquett, A. OA1503

Zangeneh, S. OA2002

Zemil, M. OA1203

Zhang, L. OA0306, OA0606, OA0607LB

Zhang, S. OA1803, OA1805

Zhao, X. OA0303

Zhao, Y. OA0207

Zheng, Z. OA1205

Zhou, T. OA2602

Zhu, L. OA1203

Zou, Y. OA1606

Zulu, S. OA0803LB

Zwane, P. OA0903

